# Resilience as recovery: stakeholder perspectives on Uganda's health system response to infectious disease shocks

**DOI:** 10.3389/frhs.2026.1888814

**Published:** 2026-07-22

**Authors:** Denis Okethwangu, Mahima Venkateswaran, Alex R. Ario, Sandra Nabatanzi, Evelyn Asio, Felix Ocom, Charles L. Okot, Anna Nyisomeh, Victoria Nankabirwa, Elizeus Rutebemberwa, Roy W. Mayega, Suzanne N. Kiwanuka

**Affiliations:** 1 Department of Health Policy, Planning and Management, Makerere University School of Public Health, Kampala, Uganda; 2 Uganda National Institute of Public Health, Ministry of Health, Kampala, Uganda; 3 Department of Global Health, Norwegian Institute of Public Health, Oslo, Norway; 4 Department of Epidemiology and Biostatistics, Makerere University School of Public Health, Kampala, Uganda; 5 National Public Health Emergency Operation Center, Ministry of Health, Kampala, Uganda; 6 Epidemic Preparedness and Response Cluster, World Health Organization Regional Office for Africa, Brazzaville, Republic of Congo; 7 Global Health Security Project, Baylor College of Medicine, Kampala, Uganda; 8 Resilience Africa Network, Makerere University School of Public Health, Kampala, Uganda

**Keywords:** disease outbreaks, One Health, pandemics, resilience, psychological, Uganda

## Abstract

**Background:**

The conceptualization, definition, and measurement of health system resilience remain contested, hindering policy integration. Country-specific empirical evidence from sub-Saharan Africa, where infectious disease shocks recur, is limited. This study explored how national-level Ugandan stakeholders conceptualize health system resilience to infectious disease shocks and its attributes.

**Methods:**

We conducted a descriptive exploratory qualitative study using semi-structured key informant interviews with 15 purposively selected national-level stakeholders from the Ministry of Health, academia, and development and implementing partners between May and July 2025. Data were analyzed using a hybrid deductive–inductive (abductive) thematic analysis approach, drawing on Kruk et al.'s and the WHO's resilience frameworks while remaining open to emergent themes. Reporting follows the Consolidated Criteria for Reporting Qualitative Research (COREQ).

**Results:**

Participants conceptualized resilience across five capacities: preparedness, absorptive, adaptive, transformative, and recovery. Recovery-dominated narratives prevailed, while transformative capacity was least articulated, indicating a system oriented toward “bouncing back” rather than reform: A resilient system in Uganda is one that anticipates and prepares for infectious disease shocks; absorbs them while sustaining essential services; adapts through innovation and operational adjustment; recovers core functioning; and institutionalizes learning to transform structures, financing, and service delivery models in preparation for future shocks. Seven interconnected attributes were identified: (1) governance and leadership, (2) preparedness, (3) service delivery, (4) community involvement, (5) adaptability, (6) collaboration and partnerships, and (7) soft skills. Governance and leadership emerged as foundational; preparedness, service delivery, community involvement, adaptability, and collaboration as structural; and soft skills (trust, communication, cultural sensitivity) as relational enablers.

**Conclusions:**

From the perspective of national-level actors, Uganda's health system is predominantly reactive, with recovery prioritized over transformation. Strengthening resilience requires institutionalizing learning, anticipatory financing, empowered subnational structures, and multisectoral partnerships. Future research should triangulate these perspectives with subnational, frontline, and community voices.

## Introduction

The COVID-19 pandemic, occurring within 5 years of the prolonged Ebola Virus Disease crisis in West Africa, ravaged health systems worldwide, including those in high-income countries (1, 2). These infectious disease shocks highlighted the need for health systems to be resilient beyond just their preparedness to respond to emergencies. Consequently, health system researchers began to explore the concept of resilience in the health sector. Among several framings of health system resilience, one influential view conceptualizes a resilient health system as a learning system (3, 4). Priority research areas include measuring and managing the dynamic system performance, and areas such as, linkages between societal and health system resilience; the impact of governance on resilience capacity; establishing legitimacy; and the influence of the private sector on health system resilience (5). Preparedness tools and indices used to assess system readiness were deemed inadequate for measuring resilience due to incongruence between measurement scores and the actual impact during shocks (6, 7). Joint External Evaluation (JEE) and the Global Health Security Index (GHSI) findings have been used as proxy measures for resilience but COVID-19 exposed discrepancies between scores and observed performance (8, 9). The WHO's Health System Resilience Indicators were introduced as improved measurement strategies where the JEE and GHSI proved insufficient (10, 11). The Epidemic Preparedness Index (EPI) had been introduced earlier, before the COVID-19 pandemic, to counter inadequacies in the development of tools that had been used to assess health systems (10). Definitional ambiguity compounds the measurement challenge. The more commonly applied definition emphasizes absorptive, adaptive, and transformative capacities (8, 12, 13). The World Health Organization (WHO) defines health system resilience as “the ability of all actors and functions related to health to collectively mitigate, prepare, respond, and recover from disruptive events with public health implications, while maintaining the provision of essential functions and services and using experiences to adapt and transform the system for improvement” (14). However, such definitions may not fully capture all unique perspectives worldwide, given the heterogeneity of national and subnational perspectives (11). The concept could benefit from further refinement in terms of definition, measurement, and scope (15, 16). Witter et al. describes it as a process shaped by relationships and power structures (15). Africa CDC and the Global Health Security Agenda emphasize measurable capacities, such as workforce, surveillance or coordination (17). Emerging voices call for the inclusion of equity in the definition of resilience (18). The absence of a universally binding definition allows national conceptualization but complicates cross-country comparison and may misalign global standards and local practice. Empirical research on health system resilience in sub-Saharan Africa reveals context-specific challenges and capacities that differ substantially from those of high-income health systems. Recent qualitative research in East Africa has identified several tensions, including the disconnect between decentralization policies and actual fiscal capacity at the local level, weak institutional structures for capturing and formalizing lessons from outbreaks, and a reliance on donor-funded preparedness programs rather than locally-designed initiatives (19, 20). These patterns have been documented in qualitative research following Ebola and COVID-19 responses across the region and continent (21, 22). In 2022, Uganda responded to an outbreak of the Ebola Virus Disease, which was first confirmed in a patient in the central district of Mubende (23). Although this was the country's seventh Ebola outbreak since the first in 2000, it was the first since the last in 2012 (24). The response to the outbreak was plagued by similar challenges, as documented in other low- and middle-income countries (25, 26). Following the 2022 outbreak, Uganda has responded to two other Ebola outbreaks, in 2025 and a current outbreak in 2026 (27, 28). Moreover, very few empirical studies have examined how national-level stakeholders in East Africa conceptualize and operationalize health system resilience in response to infectious disease shocks. Understanding local stakeholder definitions is critical for translating global frameworks into context-specific policies and initiatives. To contribute to this evidence base, we explored how Uganda's national-level health system stakeholders define resilience to infectious disease shocks and its attributes, with the aim of proposing a contextualized definition relevant to Uganda and comparable settings.

## Methods

### Study setting

**Figure 1:**
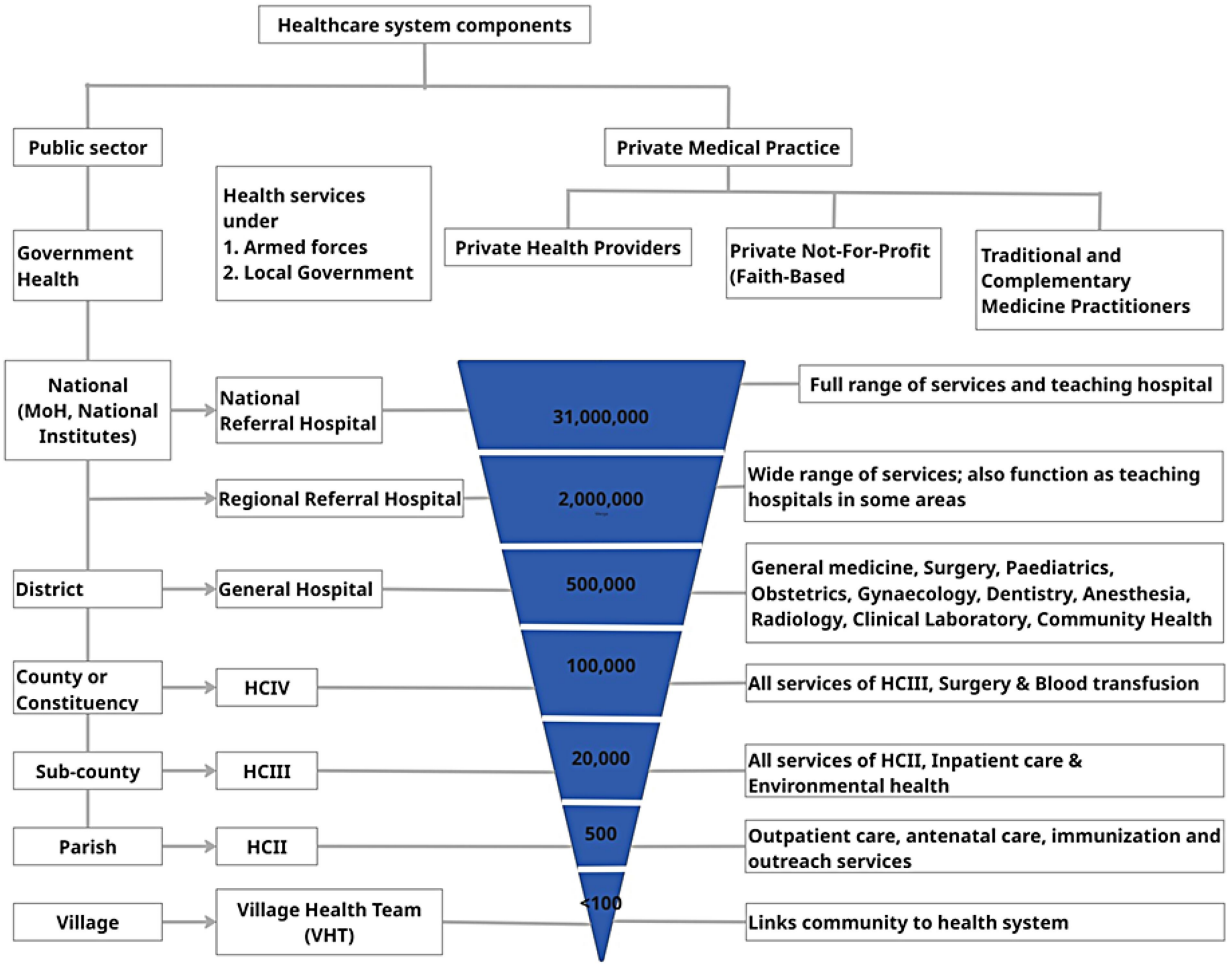
Hierarchical structure of the decentralized Ugandan health system (30).

This study was conducted within Uganda's healthcare system, structured into 16 health regions and operating under devolved governance, in which authority is transferred from the central Ministry of Health (MoH) to districts. Governance of national, regional, and general hospitals remains under MoH (29). Service delivery is hierarchical; a national referral hospital, regional referral hospitals (one in each region, each comprising an average of nine districts), 23 general hospitals across 145 districts, Health Centres IV (highest level in districts without a general hospital), Health Centres III at the sub-county level, and Health Centres II at the lowest tier. Community health structures, including Village Health Teams (VHTs) and Community Health Extension Workers (CHEWs), provide primary care at the community level. Detection, investigation, and management of infectious disease outbreaks fall under the mandate of districts, with national-level technical and financial mobilization for major outbreaks. Figure 1 details the hierarchy and services provided.

### Study design

We conducted a descriptive exploratory qualitative study examining key national and subnational stakeholders' perspectives on the definition and attributes of health system resilience to infectious disease shocks. We focused on shocks caused by infectious disease outbreaks, given their recurrent nature and impact on health system performance. Data collection was carried out between May and July, 2025.

### Study participants

To capture strategic and policy-level perspectives, we purposely selected national and subnational stakeholders based on their formal roles within the health system and substantive experience in Uganda's health system (31). We identified 16 prospective participants, including: Directors, Commissioners, and Assistant Commissioners of Health Services at the Ministry of Health; leading implementing partners (Baylor Uganda and the Makerere University Infectious Diseases Institute); development partners (WHO and CDC); and academics and researchers (Makerere University, Busitema University, and Kabale University). All participants were informed of their enrollment in the study via email. One participant was unavailable and interviews were conducted with 15 participants.

### Sample size justification

Rather than relying on saturation alone, we drew on Malterud et al.'s (2016) concept of information power to justify our sample. The aim is narrow and well-specified (perspectives on definition and attributes of resilience); participants are highly information-dense by virtue of their senior policy and technical roles; the study is theoretically informed; interview dialogue was rich; and the analytic strategy is in-depth case-oriented rather than cross-case generalizing. These factors support adequate information power at *n* = 15. We complemented this with attention to thematic adequacy during analysis (26–28); no substantively new attributes were identified in the final three transcripts.

### Data collection

We conducted key informant interviews through semi-structured, open-ended interview guides, which were administered to participants by the first author (DO), assisted by a team of experienced research assistants. There were six research assistants, half of whom were females. All the research assistants were highly experienced in qualitative data collection; five had a Master's degree and one had a Bachelor's degree. Prior to data collection, the research assistants underwent training in conducting key informant interviews. All interviews were conducted in English.

The interview guide (Supplementary File 1) covered: (1) participants' organization role, and years of experience in health systems; (2) descriptions of health system resilience to infectious disease shocks; (3) perceptions of what makes a health system resilient to infectious disease shocks, generally and specifically in Uganda; (4) assessment of Uganda's current resilience; (5) actions implemented that have strengthened the system; (6) recommendations for further strengthening, and (7) challenges of building a resilient health system in Uganda. Interviews were conducted face-to-face at the participants' individual offices. Two participants unavailable locally were interviewed virtually, via Zoom® (32). Each interview was facilitated by two research assistants, one guiding the interview and the other taking notes. Interviews lasted approximately an hour and were audio-recorded following informed consent.

### Data analysis

Audio recordings were transcribed verbatim by experienced research assistants led by the first author (DO). Transcripts were returned to participants for their review and concurrence with the contents (member checking at the transcript level); 11 of 15 participants responded, three suggested minor clarifications (e.g., correcting role titles), and no substantive content changes were made. Transcripts were imported into NVivo 15. We adopted a hybrid deductive–inductive (abductive) thematic analysis approach (33, 34), iterating between *a priori* sensitizing constructs and themes emerging from participant accounts. We applied the six-step process of thematic analysis, including: (1) data familiarization, (2) code generation, (3) integration of codes into themes, (4) revising themes, (5) determining the importance of the themes, and (6) reporting the results, as suggested by Braun and Clark (34). Thematic analysis helped us to identify, analyze, and report patterns of themes within collected data, sometimes extending to interpreting various aspects of the research topic (34, 35). Initial codes were informed by the *a priori* resilience capacities described in the literature, but coders remained responsive to novel patterns and codes that freely emerged from the data (12, 14, 36). This enabled us to assess whether Uganda's national stakeholders' conceptualizations were aligned to established frameworks. Following transcription of the data, the analysis team read the transcripts to familiarize themselves with the data that had been collected. From each transcript, codes, which are pieces of relevant information, were extracted. Codes were extracted based on the research questions being answered. Therefore, extracted codes were related to the definition of health system resilience to infectious disease shocks and the attributes of a resilient health system. Following the initial coding process, sub-themes were developed from related codes. Finally, broad themes were generated to describe the health system's resilience to infectious disease shocks and its associated attributes.

This study faces a significant risk of social desirability bias. Participants, especially government officials, may have been reluctant to articulate critical assessments of Uganda's health system or their own institutions, instead offering retrospectively constructed narratives of success or justified reactive approaches. Additionally, some participants had prior professional relationships with the research team, particularly the first author (DO), who has engaged in health systems work in Uganda for extended periods. This familiarity may have influenced the depth and candor of participants' responses, and respondents may have avoided sensitive critiques or emphasized particular achievements known to align with the research team's or their institution's interests. To mitigate these biases, several strategies were employed. First, we deliberately included respondents from diverse organizations (Ministry of Health, academia, WHO, CDC, implementing partners) to surface divergent perspectives and reduce institutional consensus bias. Indeed, notable tensions did emerge in the data (e.g., between Ministry of Health narratives of “successful withstanding” of crises and partner observations of healthcare worker burnout and demoralization). Second, interview guides used open-ended prompts, rather than leading questions, and the interviewing team included two research assistants per interview, one guiding and one recording notes independently, to create a check on interviewer-driven framing. Third, member checking was conducted at the transcript level (participants reviewed and endorsed their transcripts), allowing respondents to flag misrepresentations or context that had been missed. To minimize additional biases and risks associated with a single coder (e.g., subjectivity or confirmation bias), two coders were involved. Any discrepancies between the coders were resolved by consensus after discussions incorporating different perspectives. The researcher did not compute an inter-rater reliability score; however, the consensus-building process ensured the credibility of the data interpretation. The principle of thematic saturation was used to determine the number of interviews (37–39). We report the study in accordance with the consolidated criteria for reporting qualitative research (COREQ).

### Ethical considerations

All participants provided written informed consent prior to the key informant interviews. No names were recorded during the transcription. Every participant was assigned a study number, which was used to refer to them during the analysis step. The study was approved by the Makerere University School of Public Health Research Ethics Committee (REC) (approval Number: SPH-2024-609).

### Findings

#### Description of respondents

We interviewed 15 key informants. Thirteen were male, and seven were from the Ministry of Health, four from academia, two from implementing partners, and two from development partners. Eleven had over 10 years' experience in health systems (Table 1).

**Table 1:** Characteristics of the key informants.

Characteristic	Detail	*n*; (*N* = 15)
Sex	Male	13
	Female	2
Occupation	Academia	4
	Ministry of health	7
	Implementing partner	2
	Development partner	2
Number of years in health systems	<5	01
	5–10	03
	>10	11

Findings are presented in two sections: (1) the conceptualization of health system resilience and (2) attributes of a resilient health system.

#### Conceptualization of health system resilience

**Figure 2:**
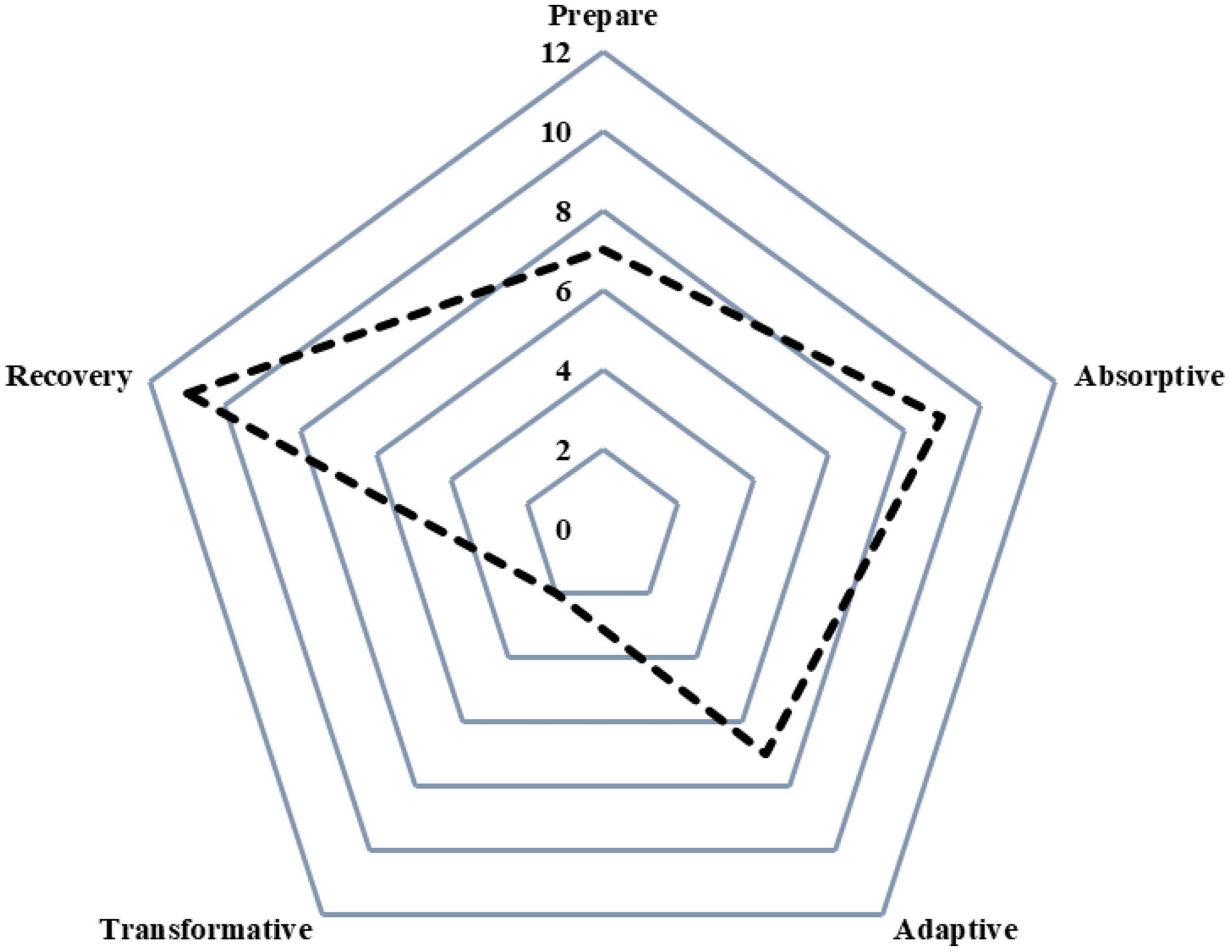
Relative prominence of five resilience capacities in informant narratives (proportion of coded references).

Five capacities emerged: preparedness, absorptive capacity, adaptive capacity, transformative capacity, and recovery. Recovery was the most dominant theme associated with health system resilience, while transformative capacity was less dominant (Figure 2).

#### Preparedness

Participants described preparedness as a multifaceted capacity encompassing human capital development, financing, and supply chain readiness:

“A resilient health system is one with critical number of trained health workers to quickly respond to an outbreak whenever it arises…” (KI07, Development Partner, Global Health Security).

“A resilient system is a prepared one…when we talk about commodities and supplies, in Mbale, I could tell you from my experience that the personal protective equipment (PPEs) and other supplies were available where they had to be” (KI05, Academia, Researcher).

A senior MoH respondent linked preparedness explicitly to financing dependency:

“…our health system has been largely on external support, so this does influence our preparedness capacities, especially when funds are cut like we have been experiencing in the recent past” (KI01, Ministry of Health, Policy Maker).

This account reveals an important tension: preparedness is operationally framed (supplies, workers) but financially fragile. Notably, no informant articulated a domestic financing pathway for routine preparedness investment, a silence that itself signals limited institutional foresight.

#### Absorptive capacity (ability to withstand crises)

Absorptive capacity was framed through emergency workforce surges, community participation, and institutional memory:

“The Ministry of Health has also made emergency recruitment of healthcare workers during emergencies, which has significantly contributed to the ability of the health system to absorb shocks” (KI02, Academia, Senior Academic).

“The Ugandan health system may be considered resilient because we have successfully responded to, and withstood several disease outbreaks. A resilient system can successfully withstand an outbreak. There is extensive human resource experience to handle outbreaks like these effectively” (KI07, Implementing Partner).

“There is no resilience when the health system cannot effectively respond to a crisis, and in Uganda, the community plays a big role in that” (KI15, Ministry of Health, Senior Director).

A counter-note came from a partner respondent:

“Crises strain human resources… it is essential to have an adequate pool of staff to prevent burnout and ensure that there is continuation of provision of essential services during outbreaks” (KI6, Development Partner, WHO).

The latter introduces a tension absent from MoH accounts: absorption through repeated emergency mobilization may erode rather than reinforce capacity over time. This divergence between official (“we have successfully withstood”) and partner (“burnout threatens absorption”) framings suggests resilience claims may be retrospectively constructed rather than structurally grounded.

#### Adaptive capacity

Adaptive capacity was illustrated through technological integration and creative service delivery models:

“…to allow for continuity of essential services we should be embracing things like mobile technology in access of care, to complement the health workers doing their job, so even when the healthcare workers engage in the response, some of the automated systems ensure there is no interruption” (KI05, Academia, Researcher)

“From my understanding of resilience of health systems… when we had the lockdown, there were essential services, what they call mission-critical services, which were left to run, giving our health system a semblance of resilience” (KI09, Ministry of Health, Commissioner of Health Services).

The phrase “a semblance of resilience” is analytically telling: even senior MoH actors register adaptive capacity as partial and performative rather than systemic.

“…if your health system cannot adapt to changes it becomes a problem. I will give an example, if you saw how we started with COVID-19, which no one knew much about. We needed the ability to streamline effective care pathways in the healthcare system and provide identification of isolation cases, and then streamline triage and referral systems to minimize impact on the routine services that need to continue in a way implementing differentiated service delivery models” (KI13, Implementing Partner, Global Health Security Team Lead).

“…the integral parts of the system can deal with the outbreak but also continue to… deal with the other issues” (KI06, Development Partner, WHO).

These responses pit adaptation as the capacity to introduce measures, technologies, or necessary adjustments to ensure the continued provision of health services during crises.

#### Recovery

Recovery dominated participant accounts, framed as return to pre-shock functionality rather than transformation:

“…a resilient health system should be able to come out of that shock and proceed with the provision of regular and routine services” (KI14, Implementing Partner B, Senior Programs Officer).

This description of recovery speaks through the lens of a health system that is not keen on making an overhaul of its structures in response to learnings from the response. This view was further reinforced by a respondent who said:

“Then after it is gone, everybody forgets… very few of the good things achieved during COVID-19 are being maintained.” (KI13, Implementing Partner, Global Health Security Lead).

This quote reveals the post-recovery mindset of the Ugandan health system, which is premised on bouncing back rather than making the necessary fortifications to withstand future shocks more effectively. This mindset seems driven by limited funding, as mentioned by a respondent:

“…limited funding causes us to focus mainly on health system recovery” (KI14, Implementing Partner, Senior Programs Officer).

These accounts converge on a recovery orientation that is reactive, donor-cycle dependent, and institutionally amnesic. The “everybody forgets” account is particularly significant: it points not merely to weak learning systems but to the absence of formal institutional mechanisms (after-action reviews routinized into policy revision; budget protections; structured documentation pipelines) that would convert experience into reform.

#### Transformative capacity

Transformative capacity, the least articulated, was framed primarily as aspiration:

“Therefore, a resilient health system is a learning one… needs to be a learning system that can study what is happening and implement appropriate reforms. In terms of infrastructure and service delivery models, we need structures that can accommodate changes without excessive difficulty if modifications become necessary” (KI02, Academia, Senior Academic).

The view of this participant suggests a system that is able to make significant changes in terms of infrastructure and service delivery models based on lessons learned from previous response activities. Based on the participant’s response, this capacity speaks more to system actors' desire to learn during emergencies, yet the current system struggles to institutionalize the lessons learned.

#### Proposed contextualized definition

On the basis of the five capacities (preparedness, absorptive, adaptive, transformative, recovery) and seven attributes that emerged from participant accounts, and informed by the iterative application of abductive thematic analysis, we synthesize the following definition of health system resilience contextualized to Uganda:

A resilient system in Uganda is one that anticipates and prepares for infectious disease shocks; absorbs them while sustaining essential services; adapts through innovation and operational adjustment; recovers core functioning; and institutionalizes learning to transform structures, financing, and service delivery models in preparation for future shocks.

This definition reflects the participants' emphasis on resilience capacities while highlighting the Uganda-specific tensions that recurred in the data: the gap between anticipatory policy and reactive funding; the need for transformative capacity but the system’s orientation toward recovery; and the critical role of institutional learning mechanisms that remain underdeveloped. The definition is not prescriptive in the abstract sense, but rather descriptive of what resilience would require given Uganda’s current constraints and system strengths.

The system should then, by learning lessons both during and after the outbreak make the necessary transformations to build stronger structures capable of withstanding future shocks. A health system that can rebuild its structures after a devastating shock to provide quality services afterward may also be considered resilient.

#### Attributes of a resilient health system

**Figure 3:**
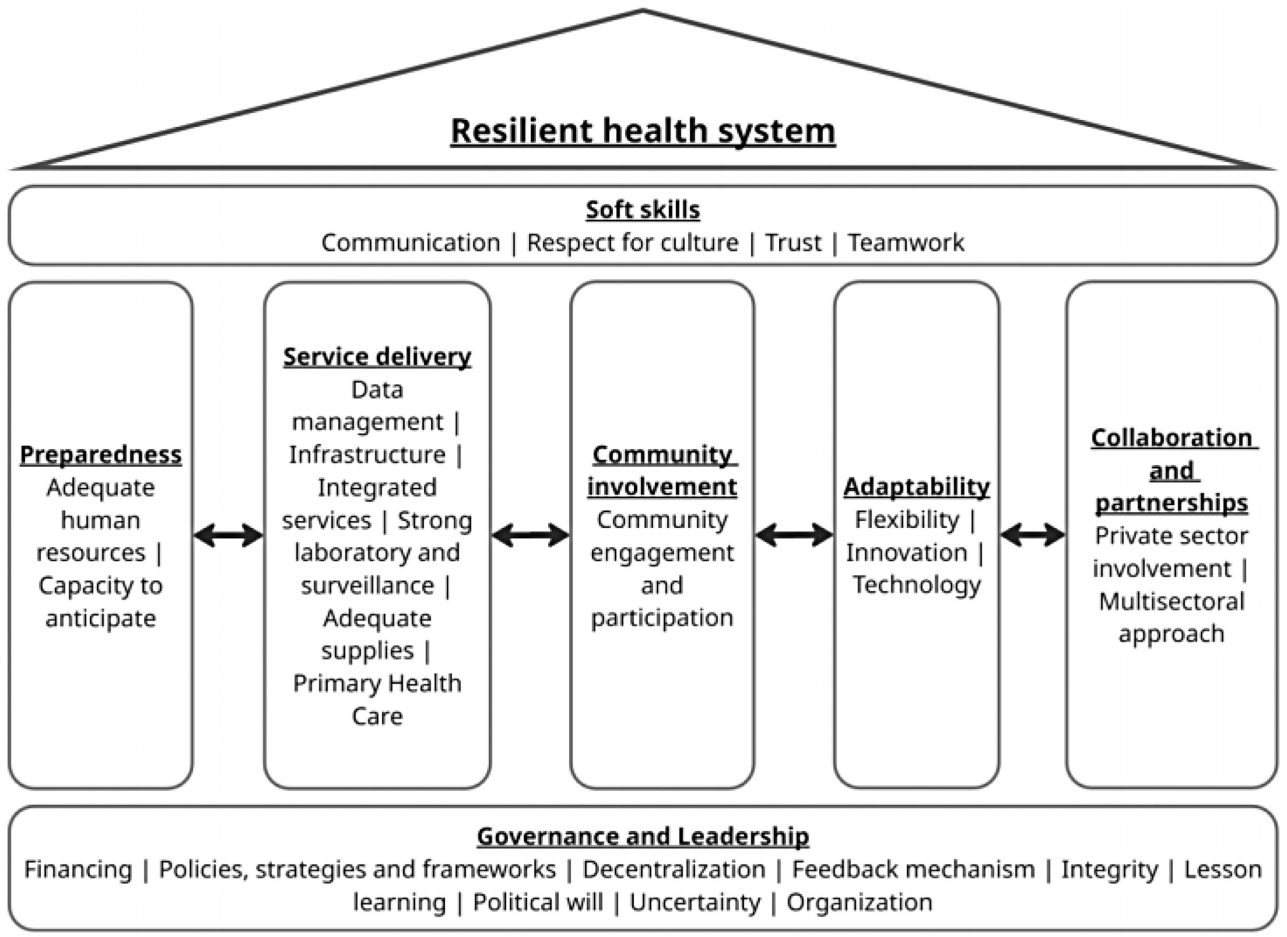
Layered architecture of attributes of a resilient health system.

Seven interrelated attributes, which represent both structural and relational elements emerged, namely: (1) governance and leadership; (2) preparedness; (3) service delivery; (4) community involvement; (5) adaptability; (6) collaboration and partnerships; and (7) soft skills. Their relationships are summarized in Figure 3.

#### Governance and leadership

Governance and leadership encompassed strategic guidance, financing, coordination, contingency planning, and policy frameworks:

“During the COVID-19 pandemic, we had a contingency fund… For a health system to be resilient, it needs to plan and recognize that these types of shocks may occur” (KI02, Academia, Senior Academic).

“The leadership and governance have a role to ensure that there are policies, guidelines, and adequate funding to make the system resilient” (KI13, Implementing Partner, Global health Security Lead).

These aspirational statements reflected participants' sense that governance should be forward-looking, but also revealed their perception that current governance is reactive rather than anticipatory—a gap between what resilience requires and what exists in practice. Decentralization, as a function of governance and leadership, was also framed as a resilience builder but a constrained feature:

“…decentralizing health in sub-regions is one of the features of our system. It can make us more resilient” (KI12, Ministry of Health, Technical Lead).

While there appears to be an alignment between this view and the government's decentralization policy, there remain fiscal difficulties that subnational structures face in independent decision making.

The lack of institutionalized learning was highlighted, framed as a failure:

“Then after the crisis is gone, everybody forgets. I think we need to focus on sustaining the learnings” (KII3, Academia, Senior Lecturer).

This account points not to weak governance alone, but to the absence of formal institutional mechanisms (after-action reviews tied to policy revision, structured documentation systems, budget protections for learning investments) that would convert crisis experience into governance reform. Resilience is built through institutional memory, yet Uganda’s governance structures have not yet created the infrastructure to sustain it. A countervailing positive account noted:

“…the issue of reviewing the human resource structure to meet current needs… and (the use of) technology are efforts our government has tried to undertake from lessons learned” (KI11, Ministry of Health, Commissioner).

Together, these accounts portray governance as the foundational—but contested—layer of resilience: simultaneously credited with credibility built across multiple outbreak responses and indicted for failing to institutionalize learning:

“We have credibility that has been built over time… and that has been a major source of inspiration” (KII3, Implementing Partner, Global Health Security Lead).

#### Preparedness

Preparedness was framed in the context of both pre- and post-occurrence of a shock, namely, the ability to foresee shocks and mobilize resources. It was described as a proactive approach based on prediction and planning.

“The ability to anticipate and prepare for shocks is a crucial factor… preparation means the ability to predict what is coming and to put in place what is necessary” (K13, Implementing Partner, Global Health Security Lead).

This response postures preparedness beyond stockpiling and increasing capacity to include strategic foresight, but includes anticipation. Detection was repeatedly mentioned as a marker of preparedness. Early detection emerged as a contested marker:

“We often take a long time to identify outbreaks… by the time you realize what is happening, many people have already started dying” (KI01, Ministry of Health, Policy Maker).

“We are using a system that provides quality data to ensure that this information is readily available for decision-makers” (KI05, Academia, Researcher).

The juxtaposition reveals an internal tension: informants simultaneously describe weak early detection and improved data systems, suggesting that progress in information infrastructure has not yet translated into early-warning operational performance. There was a response that provided a reason why improved data systems did not translate to strong early warning systems:

“…I think the country is doing quite well in the area of health information systems… I may not comment on the timeliness or quality of the data” (KI05, Academia, Researcher).

#### Service delivery

The attribute of service delivery was framed as the operational core of resilience, comprising the availability of appropriate infrastructure, personnel, and supplies, and service delivered in an integrated model. Notably, participants emphasized the importance of primary healthcare (PHC) in building resilience:

“…a key component is strengthening primary health care services for emergency readiness, and to strengthen response capacities, so that any potential outbreak can be managed at source without it spreading” (KI13, Implementing Partner, Global Health Security Lead).

The PHC practices that may contribute to building resilience, including community-based surveillance; health education and risk communication; and provision of integrated services. However, while there seems to be a call for strengthening PHC, both as a delivery model and containment mechanism, our health system still falls short in some aspects.

#### Community involvement

Participants described community participation as a critical enabler of resilience. Engaging communities, according to respondents, builds ownership, trust, and a sense of shared responsibilities:

“The extent to which the health system integrates and engages the community significantly helps in making the health system resilient.” KI14, Implementing Partner, Senior Programs Officer).

On ownership, another participant said:

“A sense of ownership is something that should be fostered” (KI05, Academia, Researcher).

These participants seem to point out the fact that communities participate to the extent of the level of ownership they feel towards the response. Community participation forms a feedback loop between the larger health system and end-users. Participants repeatedly mentioned the role VHTs play in that feedback loop:

“We built their capacity to support community mobilization and engagement… the community works as a link” (KI11, Ministry of Health, Assistant Commissioner).

This response shows a commitment to ensuring that the community plays a role in strengthening the health system.

#### Adaptability

This attribute was understood in the context of flexibility and creativity, enabling a response to shocks. Participants described it through examples of innovation and technology use. Technological innovations were cited as critical elements of this attribute:

“The use of phones for data collection… has significantly contributed to informed decision-making” (KI14, Implementing Partner, Senior Programs Officer).

These tools important as they raise sharp questions about sustainability. During recent outbreaks, including COVID-19 and Ebola, an application called Go.Data was very useful in contact tracing; the downside was that they required smartphones that responders were assumed to have. Innovations were not limited to technology; as participants mentioned vaccines and medical countermeasures:

“We need to develop our capacities in them” (KI14, Implementing Partner, Senior Programs Officer).

This response gives the interpretation that resilience depends on continuous innovation, both technological and organizational.

#### Collaboration and partnerships

This attribute was framed in a dual dimension; partnerships with the private sector and multisectoral coordination. Participants emphasized the critical role that private providers play in the delivery of health services, but also have historically been associated with disease outbreaks:

“Most of the outbreaks we have experienced in the country, including Ebola and other diseases, have been identified in the private sector. Therefore, enhancing PPPs and increasing the private sector's contribution to the healthcare system response are very important. Most patients access care through the private sector before seeking care in public facilities, and I believe this is crucial for building resilience” (KI13, Implementing Partner, Global Health Security Lead).

This underscores the importance of integrating private actors into surveillance and response systems. Multisectoral collaboration was also mentioned as being critical to building resilient health systems:

“We should consider issues such as cross-sector mandates and interdependencies” (KI03, Academia, Senior Academic).

This highlights the government's role in promoting resilience, extending beyond the health sector. These responses draw directly from the involvement of political players, including the President, who was actively involved during response to the COVID-19 pandemic:

“The involvement of the Prime Minister and other ministers… proves this point” (KI06, Development Partner, WHO).

This is an indication that political commitment in response operations is very important. The additional benefits that political involvement offers, include resource mobilization and risk communication.

#### Soft skills

Participants repeatedly emphasized the importance of soft skills, particularly trust, effective communication, and cultural sensitivity, in building resilience. Use the examples to describe how these soft skills have come in handy. For example; communities will respond faster if their VHT or local leader is part of a response team because they have trust in the person etc.:

“How is the trust between the government and the people?” (KI03, Implementing Partner, Senior Programs Officer).

This response in the form of a rhetorical question raises a concern about legitimacy and relational accountability. Respondents highlighted that communication through trusted community and political structures fostered acceptance and compliance:

“We need to reach the community… through structures that are trusted by the community” (KI11, Academia, Senior Academic).

This participant recognizes that resilience depends on aligning public health messaging with existing social fabrics:

“The community does have that little sense of trust in the system… regarding community engagement, I think it is critical for resilience” (KI5, Research Associate).

This is a reminder that vehicles of messages perhaps play a bigger role than the messages themselves. Messengers, when credible and legitimate, are carriers of trust.

#### Integrated architecture of attributes

The seven attributes interact dynamically rather than additively. Governance and leadership form a foundational layer providing structure, foresight, and legitimacy. Preparedness, service delivery, community involvement, adaptability, and collaboration form a structural scaffolding that operationalizes response and continuity. Soft skills function as a relational enabler that binds the architecture through trust and communication. Together, these elements constitute a context-sensitive resilience architecture (Figure 3).

## Discussion

This study aimed to explore how key informants conceptualize health system resilience to infectious disease shocks and the attributes of a resilient health system. Findings revealed that resilience was perceived as a composite comprising: preparedness, absorptive and adaptive capacities, recovery, and transformation.

Our proposed contextualized definition, namely: “A resilient system in Uganda is one that anticipates and prepares for infectious disease shocks; absorbs them while sustaining essential services; adapts through innovation and operational adjustment; recovers core functioning; and institutionalizes learning to transform structures, financing, and service delivery models in preparation for future shocks”, aligns with widely acknowledged definitions of health system resilience (8, 12, 16), while emphasizing Uganda-sepcific mechanisms and tensions. Specifically, whereas generic definitions of resilience often separate recovery from transformation as discrete capacities, our definition integrates them, reflecting participants’ insight that recovery without learning and institutional change perpetuates vulnerability—a tension that emerged repeatedly in accounts of Uganda's post-Ebola and post-COVID-19 experiences where “everybody forgets.”

The definition also aligns with that of the World Health Organization: (1) the capacity to forecast, prevent, and prepare for public health needs (such as emergencies, shocks and routine stressors); (2) the capacity to maintain the delivery of routine and essential health services in all contexts (such as emergencies, shocks, and routine stressors); (3) the capacity to absorb, adapt, and respond to changes in demand and the need for health services; and (4) the capacity to learn and improve, as required, based on experience, maintaining the course towards long-term objectives” (11). Recovery emerged as a dominant theme, framed as a return to pre-shock functionality rather than a building process informed by lessons learned from the response. This suggests a reactive orientation, which not only stifles growth of the health system but also risks perpetuating vulnerability. As our findings showed, underrepresentation of transformative capacities suggests slowness in institutionalizing learnings toward an overhaul. Findings from other LMICs have shown resilience as reactive and short-term (12, 40). For the Ugandan health system to move towards resilience, a shift towards a dynamic, learning-oriented process is necessary.

The understanding of preparedness, viewed through the availability of well-trained human resources, financing, and supply chain availability, resonates with the hardware of resilience, as coined by other scholars (41). However, based on my reflections, the overreliance on external financing and weak domestic financing threaten to undermine the preparedness capacity of the Ugandan health system. This aligns with critics of preparedness models that neglect local ownership, or where donor-driven preparedness results lack systemic strengthening (42, 43). Incorporating preparedness into routine planning could enhance efforts towards resilience in Uganda.

This study identified seven key attributes that interlink to form a suggested resilience architecture. These attributes are: (1) leadership and governance, (2) adaptability, (3) community engagement, (4) partnerships and collaboration, (5) service delivery, (6) preparedness, and (7) soft skills. These attributes align with widely recognized attributes of a resilient health system, based on prior scholarly work: (1) awareness, (2) diversity, (3) self-regulation, (4) mobilization, and (5) transformation (12). Leadership and governance, regarded as “the most complex but also the most important function of government in relation to its health system,” was considered foundational attributes upon which the other attributes are based (44). According to the World Health Organization, the responsibilities of leadership and governance encompass developing inclusive policies, enhancing regulatory and accountability frameworks, promoting research and intelligence, establishing cross-sector coalitions, and coordinating efforts with external partners to enhance health systems in a manner specific to the context (36). These roles of leadership and governance, which is also a building block of the health system, sets it up to be the bedrock on which all the other attributes are built. According to Kruk et al., adaptability “enhances the performance in the short term and, ideally, contributes to building long-term resilience” (12). However, our analysis acknowledges the tension between decentralization in policy and fiscal dependency at subnational levels, a fact noted in literature (29). The lack of institutionalization and sustaining learning may be seen both as an indictment on governance structures, and a factor that works to weaken leadership. The findings suggest that resilience requires technical capacity, strengthened by political will, organizational credibility and commitment by leadership. From the findings, service delivery emerged as the operational core of resilience, delivered through a strengthened PHC. This emphasizes the role of PHC as both the first line of defense and the foundation for continuity of care during a crisis, as suggested in the Alma Ata Declaration (45). From our analysis, it is apparent that integration of services, which appears in policy documents, remains distant in practice, hindering any effective delivery of PHC. Adaptive capacities incorporated adaptability and deployment of innovation. These capacities manifest as technological, logistical, and organizational creativity in times of crisis. Reference to the use of digital tools and innovations was mentioned in a positive light, reflecting the growing use of technology in epidemic preparedness and response. Yet, concerns about sustainability and equity persist, echoing the critique that resilience innovations in LMICs are donor-dependent and short-lived (41). Cooperation, both within the private sector and across multiple sectors, underscores the importance of collaboration and partnerships in building resilience. The recognition from the Ugandan health system experience is that many outbreaks are first detected in private facilities is a testament to the cross-operational collaboration between actors. This highlights the relevance of the whole-of-society approaches in building resilience (14). Community participation emerged as a key attribute of a resilient health system expressed through local ownership, trust, and feedback mechanisms. Community engagement has long been referred to as the “software” that runs resilience-building efforts, facilitating “legitimacy, trust and collective action” (46). Reflections on the role of VHTs further amplify the impact of these frontline players on the strength and fragilities of community engagement. Yet, their unclear functionality limits their potential impact. Soft skills emerged as strong relational assets. This study found that these soft skills underpin system legitimacy during crises. Trust and legitimacy enhance local ownership, which in turn leads to cooperation and adherence to recommendations from public health leaders; conversely, their absence can negatively impact response effectiveness (8). The seven attributes of a resilient health system identified exist interactively. Governance and leadership at the foundation; preparedness, service delivery, community involvement, adaptability and collaboration and partnerships forming the structural scaffolding; and soft skills functioning as relational enablers. Together, they create an ecosystem of resilience, shaped by the socio-political contexts in which they operate. The findings indicate that enhancing the resilience of Uganda's health system will require strategic investments in both structural and relational capacities. It is essential to institutionalize learning mechanisms post-crises, advocate funding at subnational levels, incorporate community participation and adaptive capacities into planning cycles. Cultivating a culture of foresight, rather than relying solely on external triggers, will empower the system not only to recover from shocks but also to undergo transformation through them. Ultimately, resilience must develop from a reactive attribute to an intrinsic system characteristic, rooted in governance legitimacy, strengthened local structures, and enduring community collaborations.

The seven attributes identified overlap substantially with existing frameworks. Governance, service delivery, and preparedness map directly onto the WHO building blocks (36); adaptability and community engagement align with Kruk et al.'s (12) characteristics; collaboration and partnerships reflect whole-of-society framings (14).

The genuine analytic contributions of this study are threefold: One, the empirical prominence of soft skills as a distinct relational enabler. While “software of health systems” framings exist in the literature (47), our findings position relational attributes, trust, communication, and cultural sensitivity at three nested levels (individual, organizational, system), suggesting that resilience cannot be reduced to hardware (workforce, supplies, financing) or even to governance arrangements alone. Second, the articulation of a layered architecture (governance foundation; structural scaffolding; relational enabler) that integrates rather than lists attributes. Third, the naming of Uganda-specific tensions: decentralization without fiscal devolution; data systems without operational early warning; community engagement without community voice in resilience discourse; and political mobilization without institutional accountability.

### Strengths and limitations

This study offers valuable insights into health system resilience in Uganda by involving diverse policy-level stakeholders from government, academia, and development partners. The qualitative design, supported by memoing and consensus coding, enhanced interpretation and yielded a contextualized definition and attributes of resilience that can inform policy and practice in similar settings. However, the relatively small sample size, and the short study timeframe may limit generalizability and overlook changing dynamics. Another weakness we point out is that our proposed definition of a resilient health system was not validated by participants. First, the definition reflects the research team's interpretation of participants' views on resilience, rather than an explicitly endorsed participant consensus. Presenting the proposed definition to participants for feedback might have prompted revisions, refinements, or disagreement, particularly from those in subnational structures (e.g., district health officers) or implementation roles (e.g., health facility managers), whose perspectives are underrepresented in this national-level sample. Second, the definition has not been validated against quantitative system-level indicators or longitudinal health system data. A definition may be conceptually coherent and locally grounded without being predictive of system performance. To address these limitations and extend this work, we recommend three directions for future research. First, conduct a validation study in which the proposed definition and attributes are presented to the original participants and to additional stakeholders, including subnational health managers, health facility leadership, community health workers, and community members) for feedback, refinement, and consensus-building. Second, conduct qualitative implementation research to examine whether and how these seven attributes manifest in practice across different health facilities, community settings, and governance levels in Uganda, using methods such as observational data collection and document review. Third, undertake exploratory quantitative analysis to assess whether variation in these attributes (measured through surveys or health system performance data) correlates with variation in resilience outcomes during health emergencies (e.g., outbreak detection time, case fatality rates, service continuity metrics, workforce stability). Additionally, we limited our KIIs to national-level participants, with little representation from subnational-level actors. While we carefully selected our participants, taking into account their experience in health services programming and service delivery, we acknowledge that participation by frontline and community healthcare workers was limited. Our study was designed to capture system-level perspectives, requiring participants with national or multi-district oversight rather than frontline service delivery. Expanding to community and frontline cadres would have significantly broadened the scope and logistical demands, while existing literature already documents their experiences of resilience. By focusing on key informants with specialized knowledge of financing, governance, and preparedness, we ensured methodological alignment and generated findings directly relevant to high-level policy and system reform.

These strengths and limitations together highlight the study's contribution to understanding resilience within context, while emphasizing the need for future research that includes broader stakeholder perspectives and longitudinal methods. Gender representation also requires acknowledgment. The sample is predominantly male (13 of 15, 87%), reflecting broader underrepresentation of women in Uganda's senior health system leadership positions. This imbalance may shape the findings in several ways. First, male-dominated perspectives on resilience may prioritize certain capacities (e.g., emergency workforce mobilization, technical preparedness, data systems) over those that women in health systems often emphasize (e.g., community health worker support systems, informal networks, continuity-of-care work during crises). Second, the predominantly male leadership perspective does not capture the views of female health workers, community mobilizers, or women-led organizations, who may conceptualize resilience differently based on their frontline experiences. Future studies should intentionally oversample women in leadership positions and include frontline female health workers to understand whether and how gender shapes the conceptualization and operationalization of resilience.

### Implications for policy and research

For policy in Uganda and comparable settings, the findings suggest four high-priority directions. Firstly, to institutionalize learning through formal after-action mechanisms tied to policy and budget revision. To achieve this, we suggest mandating structured after-action reviews for all outbreaks, with the Ministry of Health leading and the WHO and other implementing partners supporting. Outputs could include short policy briefs and, where feasible, peer-reviewed publications. Establishing a national repository of outbreak reports and instituting a biennial review of priority diseases in the surveillance system would go a long way in institutionalizing learning. Secondly, shift from reactive stockpiling to anticipatory financing, workforce planning, and early-warning systems with operational, not merely informational, capacity. This would mean taking operational steps, which include district-level contingency funds with clear disbursement triggers, quarterly audits of stockpiles, and pre-negotiated MoUs with partners for surge support. Workforce planning should include maintaining rosters of trained rapid-response teams. Thirdly, decentralize decision-making with commensurate fiscal devolution and empower subnational structures and community health workers as trusted feedback loops. We propose fiscal devolution mechanisms that allow districts to access emergency funds directly, coupled with quarterly dashboard reporting. Empowering Village Health Teams as feedback loops can be formalized through community health strategy guidelines and fourthly, cultivate multisectoral and One Health partnerships grounded in institutional accountability. This entails establishing inter-ministerial coordination committees with defined accountability frameworks, annual simulation exercises that include animal health and environmental sectors, and embedding One Health indicators into MoH performance reviews.

## Conclusion

This study offers a contextualized understanding of health system resilience to infectious disease shocks in Uganda, conceptualizing resilience as a dynamic capacity that spans preparedness, absorption, adaptation, recovery, and transformation. The prominence of recovery-oriented practices over transformative action suggests that Uganda's health system remains largely reactive rather than anticipatory, with limited investment in the structural reforms needed to pre-empt future shocks. The seven interconnected attributes identified, namely governance and leadership, preparedness, service delivery, community involvement, adaptability, collaboration and partnerships, and soft skills, constitute a layered resilience architecture in which structural and relational dimensions interact to sustain essential services under stress. Across these attributes, institutional memory, community trust, and strategic foresight emerged as critical yet underdeveloped foundations, underscoring that resilience is not merely a technical capacity but a socially embedded, context-sensitive process.

To strengthen resilience in Uganda and comparable settings, we recommend four high-level priorities: (1) institutionalizing learning through formal mechanisms to capture and apply lessons from past outbreaks; (2) shifting from reactive stockpiling toward anticipatory financing, workforce planning, and early warning systems; (3) decentralizing decision-making and empowering subnational structures, including community healthcare workers, to function as trusted community feedback loops; and (4) fostering multisectoral partnerships grounded in whole-of-government and One Health approaches. Future research should examine frontline and community perspectives and test these priorities longitudinally to inform policy and practice across resource-constrained health systems.

## Data Availability

The original contributions presented in the study are included in the article/Supplementary Material, further inquiries can be directed to the corresponding author.
