# Supplementary material for: Resilience as recovery: stakeholder perspectives on Uganda's health system response to infectious disease shocks
Source: Front Health Serv. 2026 Jul 22;6:1888814. doi: 10.3389/frhs.2026.1888814 (PMC13437620; doi:10.3389/frhs.2026.1888814)
Supplement: Supplementary file 1 [file Datasheet1.zip › Fig 2.pdf]

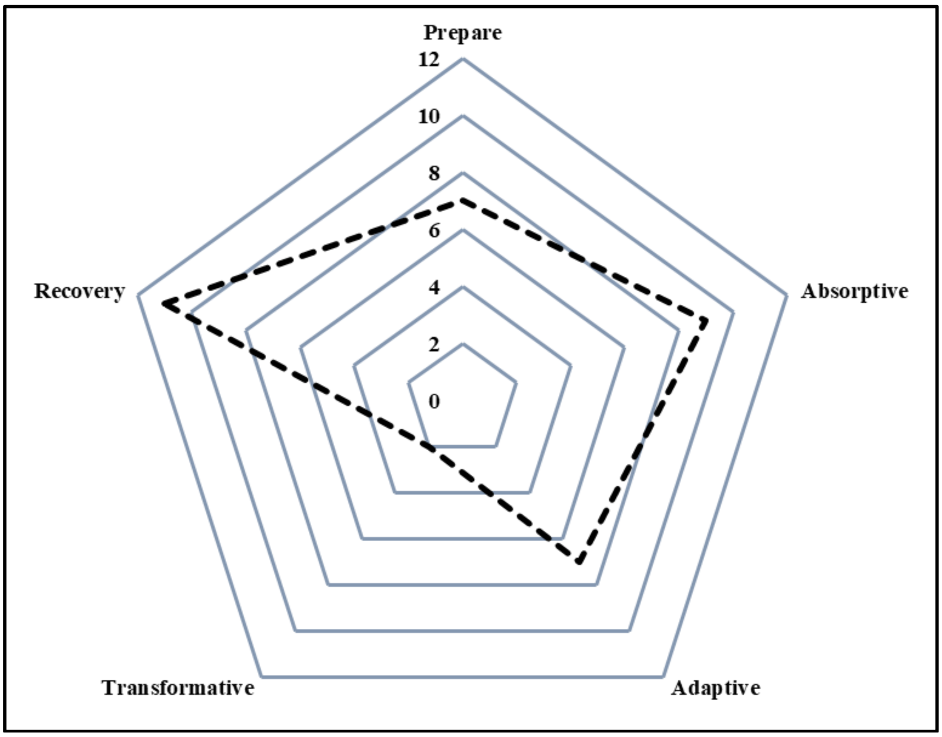

Prepare
12
10
8
6
4
2
0
Absorptive
Adaptive
Transformative
Recovery
