# Supplementary material for: Resilience as recovery: stakeholder perspectives on Uganda's health system response to infectious disease shocks
Source: Front Health Serv. 2026 Jul 22;6:1888814. doi: 10.3389/frhs.2026.1888814 (PMC13437620; doi:10.3389/frhs.2026.1888814)
Supplement: Supplementary file 1 [file Datasheet1.zip › Fig 3.pdf]

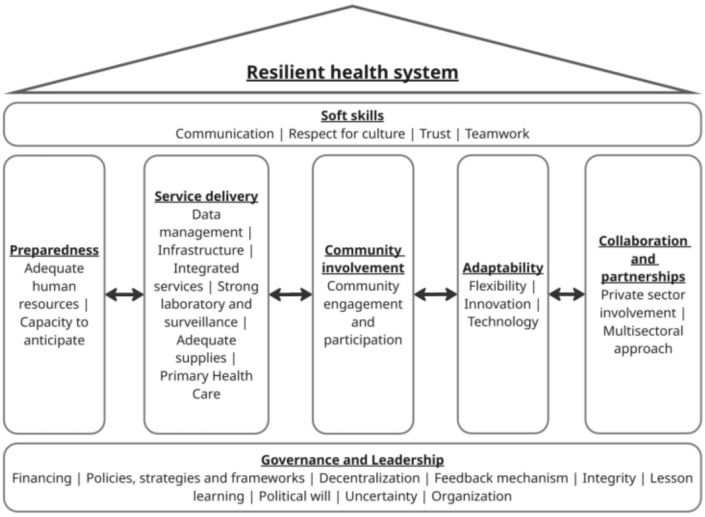
Resilient health system
Soft skills
Communication | Respect for culture | Trust | Teamwork
Preparedness
Adequate human resources | Capacity to anticipate
Service delivery
Data management | Infrastructure | Integrated services | Strong laboratory and surveillance | Adequate supplies | Primary Health Care
Community involvement
Community engagement and participation
Adaptability
Flexibility | Innovation | Technology
Collaboration and partnerships
Private sector involvement | Multisectoral approach
Governance and Leadership
Financing | Policies, strategies and frameworks | Decentralization | Feedback mechanism | Integrity | Lesson learning | Political will | Uncertainty | Organization
