# Supplementary material for: Resilience as recovery: stakeholder perspectives on Uganda's health system response to infectious disease shocks
Source: Front Health Serv. 2026 Jul 22;6:1888814. doi: 10.3389/frhs.2026.1888814 (PMC13437620; doi:10.3389/frhs.2026.1888814)
Supplement: Supplementary file 1 [file Datasheet1.zip › Fig 1.pdf]

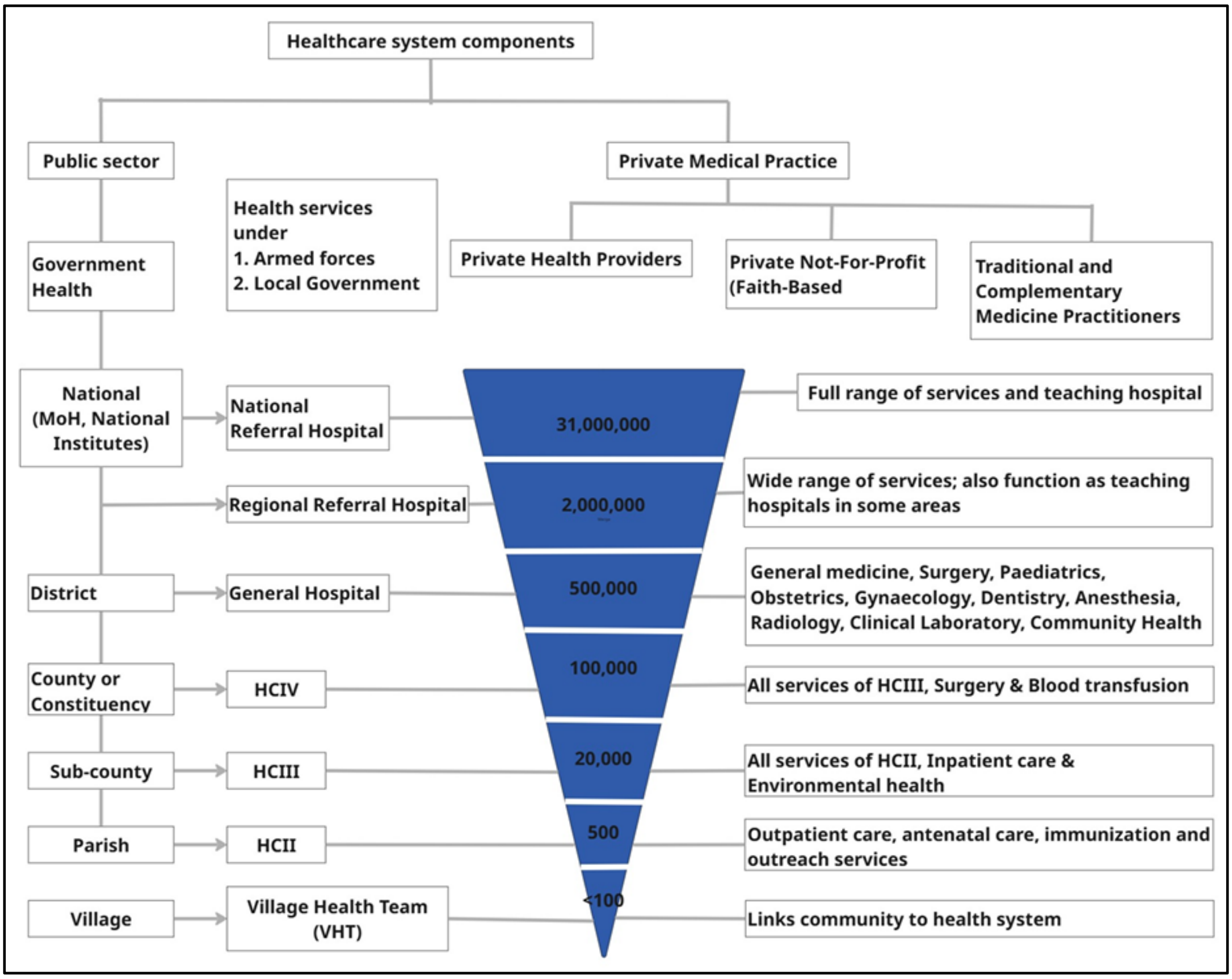

Healthcare system components
Public sector
Private Medical Practice
Health services under
1. Armed forces
2. Local Government
Government Health
Private Health Providers
Private Not-For-Profit (Faith-Based
Traditional and Complementary Medicine Practitioners
National (MoH, National Institutes)
National Referral Hospital
31,000,000
Full range of services and teaching hospital
Regional Referral Hospital
2,000,000
Wide range of services; also function as teaching hospitals in some areas
District
General Hospital
500,000
General medicine, Surgery, Paediatrics, Obstetrics, Gynaecology, Dentistry, Anesthesia, Radiology, Clinical Laboratory, Community Health
County or Constituency
HCIV
100,000
All services of HCIII, Surgery & Blood transfusion
Sub-county
HCIII
20,000
All services of HCII, Inpatient care & Environmental health
Parish
HCII
500
Outpatient care, antenatal care, immunization and outreach services
Village
Village Health Team (VHT)
<100
Links community to health system
