# Supplementary material for: Resilience as recovery: stakeholder perspectives on Uganda's health system response to infectious disease shocks
Source: Front Health Serv. 2026 Jul 22;6:1888814. doi: 10.3389/frhs.2026.1888814 (PMC13437620; doi:10.3389/frhs.2026.1888814)
Supplement: Supplementary file 2 [file Datasheet2.zip › KII09.docx]

**TRANSCRIPT OF KEY INFORMANT INTERVIEW**

**Respondent:** If you are looking at COVID response from now, today, there is the risk of underestimating why the measures were the way they were. First of all, you are dealing with a disease you don't know. They say the disease is spreading and it has spread to countries where people are dying and the health system is overwhelmed, yet those systems are stronger than yours and you don't even have the minimum they have to support life, to manage the uncertainty. What you know is it is a disease that is infectious. So what you are sure about is how to break the chain of transmission through isolation. Now, the isolation, now that it is a pandemic, the scale of isolation is what you saw in the country, where you had to go for lockdown, which by the time they were instituted, apparently we were aware of the likely consequences. The question was how long you could lock and what preconditions are required. I think that's the conversation, the lessons we can learn for the future. First of all, non-medical, when you lock people, food is a precondition. If you are able to meet people's needs for food, you can lock them longer. But if you don't meet that condition for those who survive hand to mouth, the starvation becomes more of an emergency than what you are trying to fight. Then number two, when we did the lockdown, there were essential services, what they call mission critical services, which were left to run. What we saw was mismanagement of that provision because you had health services as one of them. It was not locked to guarantee continuity of services. You also had transport services, particularly ambulances were allowed to move. That was also another one. The other was, I think the fear of health workers was not managed.

**Interviewer:** **XXXXXX, I'm going to cut you a little bit. If you could just, maybe we can be a little systematic and start in your view, your personal view as a professional, as a health system stakeholder, what would you describe as a resilient health system?**

**Respondent:** So a resilient health system is a system that can absorb shocks. So the shock in this case I'm describing is the shock of pandemic and the resultant measures. That's what shocked the system. So the resilience of a system will depend on a number of things. One is the preparedness of the system to manage the incident effectively, to reduce spread, to reduce fatalities. That's the incident. But also its ability to manage other essential services at the same time.

**Interviewer:** **So to continue providing services?**

**Respondent:** Yes, continue services while responding to this so that you minimize the adverse outcomes. This would be a resilient system. So if you want to remain resilient and minimize the impact, it boils down to your capacity. The impact is a function of the incident minus capacity. If the incident value, if the arbitrary you said is X, and your composite capacity to respond, if it is X, that incident will have no impact, but if you have incident value 100 and your capacity value is 80, then your impact will be 20. So there are determinants of that incident, like in a disease, there are issues to do with the dosages, there are issues to do with the virulence, there are issues to do with the risky behavior, risk-taking behavior of people to expose themselves. That will determine the incident. But in terms of capacity, then the health system capacity will require you to have clarity on your service package, which is the package of intervention and also the package of continuity of care. You need to be clear on the service package. It's a capacity issue, because if you don't run the vulnerabilities properly, you will not know how to put the contingency measures for response. The second would be your people.

**Interviewer: Human resource?**

**Respondent:** Human resource**,** both in terms of numbers and competencies. Now, if you are numbers, if you don't have a surge capacity, if your staffing is borderline, you cannot stretch the human resource in order to achieve the resilience, the system capacity. Then you also need resources, like financial resources, the basal financing of the health system to do its ordinary things, and your ability to mobilize additional resources to respond on time. You also move to your capacity to determine an outbreak quickly, and you detect, you investigate, and you take this action. That ability to do that work also makes a difference. Otherwise, how quickly you detect a disease also has bearing on how far it will spread, and therefore your ability to cope. So if you, and this capacity has to be before, to determine before an outbreak, to monitor during, and also to be able to manage the after effect, to measure that. Then more importantly, your ability to also, as part of the service package, your ability to communicate.

**Interviewer: Is that leadership?**

**Respondent:** No, no, that is the health promotion function, and disease prevention function, the social communication in response to managing the outbreak.

**Interviewer: That's social mobilization?**

**Respondent:** Social mobilization, because you are mobilizing people to communicate risk, so that they influence their behavior. You remember I said the disease incident weight will come from the virulence and the behavior of people, so it's a factor, so that they don't expose themselves. But your capacity must, you must have the capacity to communicate credibly to people, so that they are aware, they do the right things, and besides communication, your ability to enforce. Enforcement is a serious issue. If you are dealing with a population which has overwhelmed the state, I just look at the boda bodas. Boda bodas don't think that the traffic lights are for them.

**Interviewer: For everybody else except them.**

**Respondent:** For everybody else they must use traffic light, but for them it doesn't matter. Even an educated person, middle class person sitting on the boda boda is looking at you who is in the car with bad eyes. (laughs). It's a risky behavior. This is very risky behavior. So we cannot reduce the accidents, the head injuries, and our health system resilience investment must deal with that issue, because our systems are not coping.

**Interviewer: Very important.**

**Respondent:** So communication and enforcement is preventing behavior measures. It's a part of a response, part of a resilience. Just imagine if we did not, you saw the police, the army, they came to enforce, and if you have a population that does not see that as normal, then there's a problem. That's a public good which has to be protected. So communication is key, and you can see in our case here, the communication had to go at the level of the president. Just assume that the president was resisting interventions, nothing would have happened. Keeping Magufuli, we would not do much, because for him he said there's nothing, and the rest is history. Now this is the interface between communication as a health function, but also communication as part of governance, and leadership and governance. By the way, the communications which the president was making was our requirement, as health, that the source of communication information must be credible, and must carry authority. So the technical messaging, which we normally do here on newspapers, on the walls, and so on, this is the usual stuff. But when the updates start coming from the president, people are waiting to listen. So that is it now, to tie all these pieces together, you require also leadership and governance, both at the sector level, which should be able to coordinate partnerships, which should be able to mobilize resources, and we should be able to redefine the intervention strategy properly, and we should be at all levels of the health system. So from the national level, you go to the regional, you go to the district, and you can sit here, even going up to the village level, where the village health teams and LCs have to. So the resilience of a health system depends on how these different facets are prepared to deal with a shock.

**Interviewer: Okay. So you've already touched on my next question, but I wanted to look at the building blocks of the health system, and I noticed in your responses you're trying to pull them out, leadership, social mobilization, human resources for health. So maybe what we haven't talked about is the medicines and technologies, and what impact they can have on perhaps the health information, as well as the actual delivery of services. I know you already mentioned something about that.**

**Respondent:** Yeah, because the service delivery, which is directly linked to that incident, but the other one is continuity of care. So for you to cope with the services, for the health system to be able to cope, I'd say you need to be clear on your incidents management strategy. This is the incident we're dealing with. What are the health promotional interventions we must take? What are the health disease prevention interventions? What are the treatment elements? And what are the rehabilitative elements? We also need to be very clear on those elements, both for the disease outbreak and the essential services. You see, in other countries, when COVID came, they took their eyes off other services, for example, we saw immunization coverage in other countries falling, for routine immunization. But the Gavi CEO came to Uganda here. He came because Uganda was the only country whose routine immunization remained up. And when it happened, many people thought it happened by accident. It wasn't.

Because once COVID came, and we saw the issue of cancellation of flights and so on and so on, I got my teams here. I said, COVID is coming. How is it likely to affect immunization services, number one, vaccines were going to be affected. So we called the national medical stores, what is your stock level? The stocks were able to take us, I think, for one or two months. Then we said, we don't know how long we're going to be locked. Can we make an emergency order for Geneva to release the Copenhagen to give us our consignments? We found we had debts which would not allow us to receive vaccines. We had to clear our debts and order the stock for six months. It was deliberate. So when we did that, they brought, they chartered a plane and delivered our vaccines.

**Interviewer: Wow.**

**Respondent:** When the other countries, by the way, that vaccine was delivered when there was even lockdown. So it took very clear. Then we also said, in case of a lockdown, what will happen? Health workers will also be at risk, how do they give services under those circumstances? We produced the SOPs for continuity of immunization services. We also looked at the, we also looked at the, at what? We looked at communication of our strategy to the health workforce. We organized the Zoom meeting to prepare them. So when we did the first, the lockdown in April, immunization services went down in that month. The following month, it just rebounded quickly. But other countries which did not do this, and I think we even got an award, by the way, we got an Africa award. Yeah, it's either in the PS's office or with Jimmy. Because we, they had wanted me to go to Senegal to go and make a presentation. I said, what am I going to present, I was just doing my job. The head of supplies, go and make that presentation. And he came with an award. So it is, it is being deliberate. And again, these things also don't come from nowhere. They come from your background, how exposed have you been in management of epidemics? And it also came from trainings. We did a course in, which was organized by WHO, called Whole of Society Preparedness and Response. One of the principles of lockdown are well laid out in the training. And then the role of leadership in coordinating outbreaks, because you see, there are people who take these things as health matter. The determinants of health are outside. And if you want your health system to absorb shock, you have to deal with the other actors outside. And now, if that is clear in your head, then you know that you as health, you do not have power over Ministry of Agriculture. You don't have power over security and economics. You saw a Minister was resisting directive of the president. I'm not sure if she's a minister still. (Laughs). So you now need to realize that an outbreak or a shock or a disaster of that nature needs to be escalated to the presidency. So office of the president, office of the prime minister, which have a coordinating role. They are the ones who are better positioned to deal. So your system is being protected by preventive actions and collaborative actions outside the health system itself. So it cushions. If you do not have such a mechanism, because like our national task force would be chaired from office of the prime minister. It's like you're building a firewall. So that the health system itself with its building blocks will not be able to do this. But you need to hang on the governance mechanisms in the country to make a firewall outside you.

**Interviewer: What about maybe, we can talk about technologies, medicines and technologies, I donot know how the reach of telemedicine and such technology. You mentioned Zoom..**.

**Respondent:** Yes, we did zoom because it was a lockdown, so we could not congregate. We could not congregate. We could not also overexpose ourselves. So and if at all you do that, you need to do trainings which involve minimum movement. So now the role for, by the way most people came to know about zoom because of COVID.

**Interviewer: That's true.**

**Respondent:** The use of technology for training is key for instructing, also for resource mobilization because during lockdown I was here. I was participating in resource mobilization efforts globally for COVID vaccination. The global council for COVID, I think it was called like that, chaired by the Director of World Trade Organization. You find those big around seven or so people were the ones at the top driving the technology's agenda for COVID. I used to represent Africa on topical issues when we were discussing decisions. Oh, vaccines are coming, how do we deploy if we don't give money, what happens and so on. So technology for communication, capacity building and receiving technical support will become increasingly very important. Technology is also now, you could see with the genetic engineering, development of vaccines now is faster than before. You can also use technology for modeling disease trajectory and put out. Actually, the National Planning Authority kept modeling COVID trends and they were at variance with us. They were ahead of us. They would model and say, this is what we are seeing, it's likely it's not going to go beyond this, we can open schools. Then for us, we're saying, yes, this is good. But if we're not seeing in actual numbers a decline, it will still be a risky decision. So let's monitor trends against your modeling while we are counting numbers, but knowing that they are supposed to move the way you are doing. So at a certain point, we will decide. So technology has helped in that respect.

**Interviewer: And of course, for surveillance, right?**

**Respondent:** Yeah, for surveillance. Surveillance, now you can use platforms, ODK, for surveillance, you can now geolocate.

**Interviewer: What do you say about some of our internal capacities for manufacture of therapeutics? So I'll just, you know, we had Covidex. I don't know if you think Covidex had any impact at all, or any local remedies that were internally produced.**

**Respondent:** You see, when observation is in conflict with science, you go with observation. (Laughters)

**Interviewer:** That's a nice quote. I'm going to keep it here. I'm going to keep it here. It's important.

**Respondent:** You see, Covidex, the feedback came from people. This is a disease which produces mucoid stuff, which blocks your airway and people are saying when they drop it, the airway opens. So what is science doing about around this? And then number two, Covidex came out of locally known remedies people have used for ages. It was a matter of packaging and mass production. You see where we got ourselves? If it is a mucolytic substance, it breaks the mucus and allows it to flow with ease, where is the problem. So as long as it doesnot have its own side effects which kill, but I told you observation, people have been using it for generations. So for me, I think that is where I found the President appeared to be ahead of us scientist, because now he was saying, this man is a pharmacist, he says he understands the ingredients of these things, it is a local remedy people have been using for years, why dont you the scientists say you man, come here. I was in that meeting, it was a hot meeting, why don’t you say, you Prof, Ogwang, you are making a claim, you bring those things and I come and test in hospital in a scientific way. Then here you are big, you are being…

**Interviewer:** Resisting.

**Respondent:** But where do you want him to get the sick patients to test this thing on, does he own a hospital, he doesn’t. So where is our input as a scientific committee to prove or disprove scientifically this claim. You see, so it is like we are just sleeping, we are not looking for a solution to address this problem, we wake up like administrators who come to query your requisition. I just laugh at some people. If you sit here and make no requisition, they are also staying. Now when you say, I think my department needs to be doing A,B,C, I need E,F,G, that is when they will wake up to work. (Laughter). Query, quoting standing order. So I think we had Covidex and we also had another one from Gulu. Yes, there is a... Ola?

**Interviewer:** Yes I remember.

**Respondent:** Is she called Ola? There is a pharmacist there. She also had a meeting with the communities. Of course she comes from there, so she was more grounded. She collected the local herbs, which the communities are using for flu. And she also did a similar... Now, you can see the system is waking up and putting itself on a solution-finding mode.

**Interviewer:** Exactly.

**Respondent:** A solution-finding mode. And I think this potential is here. It is big. Just the... You see the... the... Our people also went on the vaccine. I think today we are not the same as we were before, because what, through that effort, they have been able to do is they have gotten a vector. A vector of an adenovirus of chimpanzees, from chimpanzees, which... which we can tolerate. And it is ready to be used as a vector to carry any genetically modified particle from any other virus which will come. So, we have a virus. We have platform to engineer. So, we have moved. Then, the issues which will be remaining to be done will be... Because next pandemics, the target is a vaccine must be available in 100 days. So, what it means is the genetic profile of that virus will be made public. We will be part of that race. The only things remaining will be mass production. Mass production which will require good manufacturing practice, certificates, and so on. But it is progress. So, I think the fact that our... our scientists are thinking in that direction, it is a plus. You know, in agriculture, we have one of the world-class technology centers for genetic engineering. Did you know that?

**Interviewer:** I did not.

**Respondent:** The research center for rice for Africa, is here. The groundnut species, the cassava, these people can make a potato yield from cassava and so on. So, in agriculture, NARO is more advanced in these technologies than us in the health sector. So, we just woke up. So, I think the country is in the right direction. But of course, people's expectation was the vaccine should come out and we should have used it in COVID. Development doesn't take place that way.

**Interviewer: Dr, we are falling behind time. But I will just ask about the information systems around data sharing, around data analysis. Do you think it played a little role in whatever resilience that you see? I know now we have the DHI division that is trying to make data as available as possible in the most real time. The issue maybe is around do we use the data for this?**

**Respondent:** I think we used this COVID. This COVID response was guided by science. It was guided by science. Even the evidence we generated for prioritizing target groups for vaccination, we generated it from here. And when we were engaging and then you said, this is our own data, you say the number, if you distribute deaths by age, this is the trend. So most of them they are very young, they are few old people. But they are dying more than many young people, so the risk is high. So people, because for me I don't know which part of this country I didn't reach. Whether you're educated, highly educated or not educated, I believe people understand graphs. Whether it is a pie chart, whether it is a line graph. It tells, it makes people visualize and you explain. So I think we used the data all around. The strategic meetings were evidence based which will give you information on stock, to give you information on cases, investigations, the outcomes from the laboratory. So it was data intensive.

**Interviewer:** Do you think, maybe we can, if we step a little bit away from COVID-19 since COVID-19, we've had two outbreaks of Ebola and other minor outbreaks. Do you think we are I know yes, they mentioned how we responded to Ebola in record time. But also I think what they don't mention is that we took close to one or two months before detecting that it is Ebola from the first case. I don't know if you recognize that there are weaknesses that we still have as a health system that might affect our…… (Some interruption).

**Respondent:** So you see earlier I said some of the resilience issues we have are related to how well or not well we implement now it becomes administrative or management issues. So in terms of the policies to have a strong health system or a resilient health system I think we have policies in place. So the issue is that policy frameworks from your policies your strategies, guidelines, standards I think that's not where our weaknesses are. If you see the structure of the health system it is, for me the health system we have is one of the best could be, well decentralized where all the administrative levels have got responsibility and a health system which doesn't have the normal administrative backup will falter, that is the headache we had with the health sub-districts. It had no political ownership, it was dangling in the air, even the regional hospitals as of now, the intention is good but because there is no local government structure that buttresses it, its mandate will remain questionable.

**Interviewer: Hhhmm, it is not anchored.**

**Respondent:** Yes, there is no political structure that drives, whereas if you go to a district you'll find an L.C chairman, the RDC, the Member of Parliament. You hear them asking for a general hospital, you hardly hear them asking for improvement of the regional hospital with the same energy. In fact, they attack it they condemn it because it is external to them by attacking it, they are attacking central government, so it is hanging. So if you see our district health offices, they have power to do things because there is a government there, a sub-county is a government even it has an enforcement arm, it is just that the health sector is not using the sub-county for enforcement of lawful health activities the public health act. Now if you go to the parish, the parish structure there is the parish chief but we do not have a strong presence there as a health system, now the community health extension workers are coming to fill that gap. If you go to the village, the VHTs are strong because they have also a political structure next to them. So if the VHT hits a snag, he has where to report and if the community feels that the VHT is overstepping his limit, they have where to report. So the health system at the village is strong, so our resilience comes from this structure. The policies are there it gives us our resilience, the structure which is highly decentralized and owned by communities and political arrangement, administrative arrangement gives it strength. Our resilience comes from there.

Now the last bit, the last tenet of resilience is about influencing behavior of actors, the behavior of those who support the health system, the behavior of those who provide service and the behavior of those who consume service. This is where the problem of our resilience comes from, at times when you have a big outbreak, the behavior of the partners who come is inappropriate in terms of not defining where they can stop, actually they distort the command structure, they parachute people from where they are. They are already in the field they are collecting blood, patients are complaining, the other time the Minister went to Mubende, patients were the ones who told the minister our blood is finished because everyone is going with the vacutainer taking blood. So this is just an example of inappropriate action by some actors, they can intervene badly there are people who have been ever found in these responses with blood samples in their pockets. This is inappropriate behavior. Then I went to another country there was a big cholera outbreak, there is a partner whose major intervention was with the blankets. So can blankets interrupt transmissions, so inappropriate behavior of actors is what erodes resilience in some instances.

**Interviewer: Clearly that is a leadership problem.**

**Respondent:** Yes we are not dealing with…You see if you want to streamline behavior of actors those are people, so you will provide leadership. so leadership cannot be underestimated and that's why the resilience of the system when I talked about structure, that's where the leadership is very important. So again, alignment of partnerships to one response plan is very difficult for some people, someone gets his money in a certain way he wants to spend it his way yet effectiveness comes from aligning, harmonizing agreeing on common results, holding each other accountable, then also you see inappropriate behavior I can talk about it until cows come back. You see if someone comes and says we are going to give you 20 million US dollars, it includes money which is left in their headquarters it includes money which is going to be hiring an NGO to support a district health officer, how much of that money actually goes to response?

**Interviewer: Very little.**

**Respondent:** Yes, but other people will say now already 20 million has come I think you have received too much. Now the system is going to crumble not because of anything but because of this inappropriate behavior that is indeed inappropriate behavior and it becomes burdensome let me tell you coordination of partnerships in response is one of the headaches of our systems. Everyone is exhausted occasionally some leaders become angry, they become angry my only story if I tell you I have ever chased some international NGOs from my district when I was a district health officer, I said you cannot use 7 lorries, no 3 lorries 7 pickups, 12 motorcycles to vaccinate in my district in 2 sub-counties in one week yet me with 1 vehicle and 7 motorcycles in 2 days I can vaccinate everybody in my district, what are you doing, …aah you know you see… then you bring 20 expatriates. Yes, I told them no you pack your things go away, you are causing me problems, they went and accused me they accused me here when I was brought here I explained myself, I think people said I think this young man was doing his job. The accusation went up to Geneva, some of us have lived dangerously, people who have power doing wrong things in a jurisdiction where you have a mandate, they want to trump you down. So when I just say inappropriate behavior in our partnerships, it is a huge thing. So behavior in our partnerships, behavior of the health workers themselves and then behavior of the public, the issues of trust, the issues of legitimacy when they think you are not worth taking the message, then they resist, religion comes in, culture comes in, so who is handling this issue, who is in better position to work on these issues. Now you can see resilience comes to support the health system, comes from outside the health so when you see Sheikh Mubajje talking, you see the bishops talking, these are all things which contribute to our resilience the way we respond, the way we defeat these diseases quickly, it is coming from those efforts outside the health system. So because now that is effective engagement of partnerships beyond the traditional realm of the health system.

**Interviewer: Now XXXX, I know you touched on some of them but in your opinion what would you highlight as the attributes of a resilient health system, I would have asked in a very abstract but I will just try and talk to the Ugandan context, what would be the attribute of a resilient health system in the Ugandan context?**

**Respondent:** A competent health workforce, strong leadership, decentralized governance and then the responsiveness of our partnerships because the partners are always there, they come to give a hand. So these for me would be the quick things.

**Interviewer: Okay, I know up to this point we haven't spoken much about the role of the community but also the community health workforce the VHT that we have, their unique role I was in Norway and I was talking to a small team and they were very intrigued about the community model that we have maybe that is something you can highlight about the Ugandan context.**

**Respondent:** You see the point is if you want a disease… to detect disease quickly, detecting the incident case can take long but once you have confirmed the disease, your effort now of course communities have a role in surveillance, if you leave it to them it will be the Mubende type. Indeed it was a person from the community who said there are many people with these symptoms in the community and they have died. The village health teams have a role in monitoring unusual health events, you see this is for me, the problem I have when you have no outbreak just prime your communities to report unusual health events, don't bring symptoms like water stool, no, just unusual health events, clustered unusual health events. In a community where everyone is having a red eye is an unusual health event, a community where more than 7 people have died in one week is unusual, a symptom which appears which used not to be there and it is circulating should be picked, you need a very sensitive surveillance system just to pick unusual health events including death. That is so sensitive it will bring every rumor to be investigated and then you sieve. That is what communities need in fact for me when I am managing diarrhea when I am the overall commander, during cholera every diarrhea is cholera until proven otherwise. So the clinicians will be the ones to say this is normal and in that way some of my experiences are very interesting I think I will write a book one of these days. I sat like this and I saw two young men energetic because I used to be on radio during this period any diarrhea must be reported to the health facility. So this was a young lady, I donot know what she had, I think she had food poisoning as soon as those young men saw their sister had diarrhea, they picked her ran with her when they reached my office they knocked the door opened they came and dropped her in front of me, I said hey what is this, then one of them said cholera (Laughter). You see this is a response in communities, it is a responsive system because the community structures are fitting, they are initiating referral using a vague case definition for an unusual health event and they brought this girl, you see the point. So that is all you want the communities to do, any unusual health event should be reported so that you can investigate to confirm or eliminate.

**Interviewer: Then what are your recommendations?**

**Respondent:** My recommendation is that health systems are designed to deal with health issues, they are designed to deal with issues now the resilience comes from proper implementation of those designs for example the current structure of human resources for health in the Ministry for the Ministry if it is implemented, we have enough staff to respond to the condition and enough staff to continue services, you see but the fact that we have financial constraints we are not implementing, that is the minus. Second our health systems are designed to be integrated but you also find we are torn between vertical and integrated services, our health system at this state is weak it cannot run on a vertical thing, when you hear the word focal person just know it is that is a way of mutilating the health system, so you can now comfortably have a clinical officer who is trained missing to pick a condition which is common because he is verticalised in a corner. He is a TB focal person, so he is going to miss cholera because he is not attending workshops on cholera, you may find the other day I was in Soroti and in Arua if a medical officer is working in HIV for the last 20 years, what has become of him when you take him to the maternity ward, you have lost that one because he is not all round but our trainings are all round to be able to move, you only become specialist at masters level but now a clinical officer becomes a specialist on malaria. So our services are integrated, our services are decentralized and our services are integrated and the services the structures which are the staffing structure is trained and deployed in line with the prevailing health conditions which are common. So with this for me I think judicious implementation in Uganda, the judicious implementation of the current health policy structure is all we need.

**Interviewer: I know you also mentioned in bits and pieces the challenges that we as a country have led us to where we are, I know you mentioned a few things on suboptimal implementation of policies and frameworks, our decentralization is not anchored on a political structure…**

**Respondent:** That was a shortfall otherwise in principle by the way our health services are decentralized from the national to the district to the health sub districts to subcounty to villages, but what I was trying to say is where you find there is no mirror in the political structure, it becomes weak, it struggles. The regional referral hospital is supposed to have a mandate over the region but you see it becomes somehow optional depending on the charisma of the Director. (Some disruption).

**Interviewer: Then another thing I wanted to ask about the models that we have, already talked about the VHTs, but we also have this WHO model of hubs. I don’t know if you think that is helpful or any other model that you think could work.**

**Respondent:** The one from regional referral.

**Interviewer: Yes.**

**Respondent:** Hubs and [Inaudible] they use for maternity services.

**Interviewer: What I understand is that WHO does have hubs in certain regions, from which they support the districts in that region.**

**Respondent:** Regional EOC.

**Interviewer**: **I know where the EOCs, they've been springing from the EOCs. But where there are no EOCs, they either work in the regional referral hospital and reach out to the constituent districts.**

**Respondent:** The EOCs are within the region, isn't it? Within the regional referral hospitals.

**Interviewer**: **Yes, but not all of them have, as it is now. Now, there are, I think, seven or eight EOCs. Some regions still do not have.**

**Respondent:** But they have created a structure that does function.

**Interviewer**: **At least they have a hub coordinator in most regions, where they provide surveillance support, general health system strengthening activities. But they are not well streamlined, that I should say.**

**Respondent:** You see... What I'm submitting to you is both experience and also scholarly theory. Where you don't have an established source of authority, those initiatives, people will not take them seriously. Where do you derive your authority? You see, what we are struggling with now is the political and administrative decentralization with real power and authority has moved ahead of our technical decentralization. It is a sheering effect. You see, if the Ministry was also expanding as more districts were created, and you can deploy people, then there would be no need to be talking about a regional arrangement, because the prominence for regional arrangement is because the centers are getting overstretched. Uganda had 28 districts at that time, but now we're talking about 150. So, if this is the number... By the way, I'm not against creation of districts. Let me tell you, a district which is created new, even in its most clumsy form, gives better output than the bigger, large one. That one, it doesn't matter how people look at it, that is it, it gives power, it creates platform for people to participate, for more people to participate. Now, this issue is not moving in tandem with the capacity of the center to be in touch with those districts. So, other Ministries have moved, they have created regional centers so that they now increase staffing to look after the districts. I think one major constraint we're having is pegging it to the curative facility instead of having a full-blown division or section of the Ministry of Health, which is a resident down, properly staffed to align itself with the functions we're asking them to do. So, this is a thing where we are having some challenge.

So, it carries with it some voluntary arrangement. But a structural adjustment, a structural modification to the way of doing business has not taken off with the budget, with the power, with the authority. It's hanging. So, you don't get non-actors, non-health actors talking for that arrangement. There's no non-health actor talking for that arrangement.

**Interviewer**: **And yet, their voice would have been very...**

**Respondent:** Nyeko Filbert had a problem in Arua, he was fighting with the local administration there. His actions were, there was no political structure there in the region to bless his decisions. So, his decisions would come as technical decisions and knock itself against the political decisions which are at that level and real people were power and authority. You cannot deal in times of disasters, epidemics, technically, because it affects the people. So, you require politics to deal with that.

**Interviewer**: **It is very important.**

**Respondent:** So, if the politics is not well aligned with you for some reason, you will struggle. That was a build-up of his problems. Either the outbreak found him with some problems which are related to... You see, if you don't melt into the local politics, you struggle. Those politicians, there is what they need. By the way, some of the things they need is just information. You go with the information ahead of everybody else. But if they hear from outside, then they call you, then it is an uphill task because you don't know how the other people packaged their information. But we, I think we are not prepared to deal with this.

**Interviewer**: **But that's a very, very strong recommendation. It's a very strong one, which will help the health sub-districts structure with regional referrals.**

**Respondent:** Yeah, a health sub-district that is moved to sub-counties will be stronger than a constituency-based health sub-district. And that's, by the way, the vision of the president. For him, his health sub-districts are at sub-county level. That's why now medical officers are recommended to be employed in health centre III. Yes, he is consistent. He is consistent in his things. That man, for all his problems, he is very forward-looking. Very forward-looking and he sees it afar. Yes, he sees it very far.
