# Supplementary material for: Resilience as recovery: stakeholder perspectives on Uganda's health system response to infectious disease shocks
Source: Front Health Serv. 2026 Jul 22;6:1888814. doi: 10.3389/frhs.2026.1888814 (PMC13437620; doi:10.3389/frhs.2026.1888814)
Supplement: Supplementary file 2 [file Datasheet2.zip › KII10.docx]

**TRANSCRIPT OF KEY INFORMANT INTERVIEW**

**Interviewer:** Good morning Sir! Thank you for accepting to participate in this interview. As discussed, we're focusing on health system resilience in Africa, particularly exploring experiences, enhancing capacities, and tools. To begin, based on your experience and knowledge, how do you define the term "Health System Resilience?’’

**Respondent:** I believe it's about a health system capable of withstanding and responding to various shocks and emergencies without significant disruptions or the need for extensive new arrangements. That's the simplest definition.

**Interviewer:** Hmmm…. Different stakeholders often perceive health system resilience as a state of crisis. You've provided your understanding. What do you consider the key attributes that make up a resilient health system in Uganda?

**Respondent:** The key attributes are workforce capacities, infrastructure, and finances.

**Interviewer:** Ok, please elaborate on workforce capacities, infrastructure, and finances.

**Respondent:** For the workforce, you need a database of individuals trained in various capacities. This ensures that during a crisis, you know exactly who to call upon, as some are specifically trained for such situations. Regarding infrastructure, we need adequate facilities, including equipment, tools, and physical structures. For example, during COVID-19, if our Intensive Care Units (ICUs) or Regional Referral Centers had been fully prepared, we might have averted a severe crisis. Unfortunately, ICUs weren't ready, and many lives were lost due to this lack of infrastructure. If the infrastructure had been in place, we would have been more resilient. ICUs are a prime example of essential physical infrastructure. And, of course, finances are indispensable. You cannot transport a person from one place to another without financial resources. You need funds to procure facilities, support human resources, acquire logistics, and pay allowances. So, finances are absolutely essential. I believe these are the primary areas defining a resilient system.

**Interviewer:** Now, if we consider these factors and divide them between the National and Sub-National levels, which do you believe would be most effective at each level?

**Respondent:** I think all of them are required at all levels. What I mentioned are strategic components applicable at every level. The capacities needed at the National level primarily focus on coordination and leadership. At the Sub-National level, these capacities are more operational and tactical. You need people with diverse skills in areas like surveillance and contact tracing, more technical capacities at the sub-national levels. While finances originate from the National level, they're crucial for various activities at the sub-national level. If you were to ask me specifically, I would say leadership and governance are paramount at the National level, while technical and tactical capacities are key at the Sub-National level.

**Interviewer:** Thank you very much. Just as you've highlighted strengths like leadership and governance, what factors contribute to a weaker health system?

**Respondent:** Weaknesses are essentially the opposites of the strengths I mentioned. This includes a lack of workforce capacities in certain areas, insufficient funding (especially readily deployable funds), and inadequate infrastructure. These factors create weakness because they mean that in times of crisis, you'd either have to hire consultants from organizations like the World Health Organization or from other countries, which takes time. A lack of finances also means you'd have to start applying for funding from pandemic funds, emergency funds from WHO, or from partners. So, weaknesses are the direct opposite of strengths, indicating where we lack capacities. Of course, you might not lack all capacities, but shortages at any level constitute a weakness.

**Interviewer:** Okay….. could you elaborate on community engagement regarding health resilience? What's your perspective on that?

**Respondent:** Community engagement is one of the essential capacities. When a community is properly engaged, using existing community structures, it significantly eases the response to diseases and other crises because these issues often begin and end within communities. Therefore, if communities are properly engaged, meaning they can identify the source of a crisis and understand what needs to be done to resolve it, then we have a resilient community capable of ending a crisis through their own means. This includes providing information, monitoring interventions at the community level, and sharing information, especially during an outbreak. If the community is well-sensitized, they can also participate in community-level interventions. In such a scenario, a well-sensitized community is a resilient community, forming a vital part of a resilient health system.

**Interviewer:** I'd like us to look at interventions or actions taken within the Ugandan health system from your perspective as a technical person in surveillance that have contributed to a resilient health system, particularly in terms of disruptions.

**Respondent:** The interventions have been quite numerous. The first one is capacity building across several areas or pillars. We've built capacities in outbreak investigations, surveillance, community engagement, risk communication, case management for various conditions, and data management and reporting. So, across all preparedness and response pillars, capacities have been well-developed through what we call Rapid Response Team (RRT) training. Furthermore, through Evoke Search, a WHO initiative, we've created a reserve of highly capable individuals in different response pillars. We also have that reserve to train people, and I haven't overlooked capacity in leadership and governance under the same arrangement, specifically leadership and coordination.

Another broad intervention has been the ability to mobilize resources. We've developed capacities for rapidly deploying resources by establishing a number of supporting projects. These include a World Bank project, a Korean project, the Global Fund, and a COVID-19 grant under the HIV grant. This has allowed us to mobilize resources both domestically and from other partners concerning financing.

Additionally, we've established several infrastructure projects, including two national facilities—Entebbe and Mulago, I hope you're aware of them. We've also managed to set up public health operational centers within national mobilization facilities in the regions. These are crucial structures for resilience. Other structures include intensive care units in national and regional referral facilities and equipment for installing oxygen plants. We now have very large oxygen plants installed in national referral facilities; this was a direct result of COVID-19, as the need for oxygen became evident. I've just mentioned a few examples covering financing, human workforce, and infrastructure.

**Interviewer:** That's quite broad. But as you've mentioned, which of these interventions do you think have had the most noticeable impact in strengthening health resilience? Others might exist but haven't had the same impact. Like the oxygen plants you’ve discussed, could you provide examples that have truly strengthened the health system?

**Respondent:** For me, it's been workforce development across all pillars. When you have workforce development spanning coordination, surveillance, community engagement, risk management, case management, and vaccination, when you broadly consider this workforce development and capacity building, it is the most important and most impactful. All these pillars are essential. Issues related to funding and infrastructure are also important, but in terms of impact, workforce development stands out. Perhaps we should call it technical capacities.

**Interviewer:** In your opinion, do you feel some of these attributes are new to the Ugandan health system, especially considering the challenges that accompany them?

**Respondent:** They are common to all countries; there's nothing particularly unique about Uganda. The only unique aspect is that we've experienced numerous outbreaks over time, which has given us extensive experience and a clearer understanding of where to improve. This is similar to other countries like the Democratic Republic of Congo; we share similar qualities.

**Interviewer:** Ok, earlier, you discussed community engagement, stating that it makes it easier for communities to respond to and manage such cases. Now, focusing again on the community……

**Respondent:** By the way, the community is one of the pillars of response; it's one of the technical capacities I mentioned, but it cannot function alone. There are other crucial pillars, such as Risk Communication and Community Engagement (RCCE) and case management.

**Interviewer:** So is community engagement less prioritized when discussing these pillars?

**Respondent:** Personally, I don't assign an order of importance because all pillars are crucial. That's why they're called "pillars", the response to emergencies relies on them. The reason they're called pillars is that no single pillar can stand alone. If you remove one pillar, the entire response will crumble. It's like pillars supporting a house: if one is weak, the rest will weaken, or the whole response will collapse. Therefore, I cannot declare one more important than another, unless it's specifically relevant to your particular interest.

**Interviewer:** No, as you said, they all work together. I'm focusing on the community as a theme in this study. We want to understand the role of the community in health system resilience. Could you elaborate on the role the community plays in building resilient health systems? You've already mentioned some aspects, but let's broaden it.

**Respondent:** Everything depends on them, making them highly responsive and capable of monitoring crises. They can intervene in various ways, especially with highly infectious diseases. If they are provided with tools to give people for treatment, they are excellent at ensuring community access to care. In cases like malaria, where they distribute antimalarial drugs and implement other community initiatives, their ability to report is crucial. So, they play a very important role as a community structure. I believe we now have resilient communities because they've been involved in every crisis and every program. They have a significant role in being responsive to required activities. They can also provide monitoring and actual interventions, and they can report. Furthermore, they can be a source of new information. For instance, if an intervention is being rejected by the community, they will provide reasons, such as adverse effects after vaccination. They will tell you if people are accepting it, if there are many people, if it will work or be rejected. They provide firsthand information about how the community feels, and this information influences policy. So, the community is a source of new information regarding any intervention you want to implement, including outcomes or adverse effects. They will give you that information.

**Interviewer:** Looking at barriers...

**Respondent:** Well, of course, the community is basically a voluntary group, and voluntarism has always been a barrier. There's no single organization, even the government, that can provide satisfactory incentives to the community because it's quite resource-intensive. So, community engagement has largely relied on voluntary efforts. Secondly, community resource persons are not always highly educated, except in cities. You might find a community person who is a retired commissioner, and you'd be very lucky to find that, but it's rare to find a highly educated community member. Therefore, this acts as a barrier for some interventions due to the level of education. If you tell people that eating diseased meat will make them sick and possibly die, people with some schooling and a bit of science will understand, saying, "Yes, we've learned about certain bacteria that can be transmitted from animals to human beings." However, ordinary people might disagree, saying, "No way; you cannot bury meat. As long as you cook it, it will be okay." That's their level of knowledge, and it acts as a barrier. Other barriers include the ability to access certain infrastructure like the internet for digital reporting. Some areas are also hard to reach due to geographical barriers, and others are related to education, creating a barrier to using certain tools due to language differences.

**Interviewer:** In the face of such challenges, what do you think different stakeholders should do to come together and overcome these challenges, and also strengthen health system resilience?

**Respondent**: It requires an "all-government" approach. If there are no roads, the responsible ministry should address it. If there's no education, the Ministry of Education should provide Universal Primary Education (UPE). If there's no network, the Uganda Communications Commission (UCC) and the Ministry of ICT should take action. What I'm trying to say is that to overcome community barriers, all government departments should be deliberate in ensuring all communities are mobilized and also have access to basic services like education, good roads, and reliable internet, including satellite internet, even in remote areas where a physical mast isn't needed. You shouldn't need a mast to have internet; it should be accessible directly from satellites. This is the approach. Additionally, the Ministry of ICT should ensure we have substantial, if not 100%, coverage in terms of service providers for things like the internet. So, it's an all-government effort across all community initiatives.

**Interviewer:** To my understanding, you are trying to say these services have always existed. Perhaps we need to consider innovations?

**Respondent:** No, even coverage is an issue. Where is your village?

**Interviewer:** My village is Busoga.

**Respondent:** Do you have tarmac roads there?

**Interviewer:** No.

**Respondent:** That's what I'm saying. If you need good transport access, you need good roads so that you can not only travel well but also reach a good school that might be 10 kilometers away, at least by bicycle. So that's education. But now, what about ICT, internet coverage, and telephone networks? That's coverage. If you don't have this kind of coverage, there's no way you can access communication gadgets. When I was a District Health Officer (DHO) many years ago, I couldn't communicate with different district health departments. I couldn't call a District Health Director (DHD) to find out about operations. Whenever I met him, I would tell him I might call at 11 AM on Sunday, and he would be there waiting for my call on Sunday. But now, things have changed. Airtel came, MTN came. I moved away from these and could directly call his phone, from the District Health Service Delivery (DHSD) to different health centers. We were then able to communicate, for example, on vaccination days or child health days. That's how the community gets involved. But if you don't have access to roads, education, and communication, you remain backward. That's why I'm saying all these barriers can be strategically reduced by mobilizing all government institutions. When I talk about "all government," it doesn't mean you have to go to the Prime Minister. All government functions are coordinated at the district level. A district has a Chief Administrative Officer who has an ICT officer, an educational officer, an engineer for works, an engineer for water, and a health officer for health. All these. And now the government has extended to the parish level with the Parish Development Model. The same model provides for improvement in all these areas. At the parish level, someone will be responsible for providing information about education, and so on. So, when I mention "all government," it's beyond health; it's the mobilization of different sectors of government for holistic community development.

**Interviewer:** Thank you so much Doctor. Besides that, do you think there's any other intervention that could work better?

**Respondent:** Well, if there are innovations—though innovations are not common, they are like miracles. If I knew a number of them, maybe it could help but innovations simply make life easier at any level. So, it's innovations, digital systems, and others.

**Interviewer:** As we wind up, let's discuss our Village Health Teams. We often talk about their voluntary work. How do you think they can be supported to contribute to developing a more resilient health system in Uganda?

**Respondent:** The best way is to provide some form of numerical compensation, perhaps not monthly, but quarterly or regularly, and other incentives like bicycles, bags, and essential items that would encourage them to continue working. Mobile phones for calling and reporting are crucial, as we rely on their phones for these tasks. So, direct incentives that aid their transport and communication would be beneficial.

**Interviewer:** How about training?

**Respondent:** Of course, training is very important, but the most important part of training isn't an incentive; it's fundamentally about doing our work properly. The incentives that truly work are those things that help them do their work, but also benefit their personal lives. They can use that bicycle to take their wife to the hospital or ride it to town. Training, however, primarily serves our work.

**Interviewer:** Lastly, I'd like you to tell me about other successful models in other countries that you think could be adapted to enhance our health resilience system.

**Respondent:** Uganda is a leading country, so I don't know which country we could copy from. In terms of disease surveillance and epidemics, Uganda is top, even higher than many advanced countries. We don't look to other countries; they look to us, honestly. As I speak now, I am the chairman of the Continental Surveillance Advisory Committee of Africa CDC, and on that basis, they come and benchmark us, perhaps because we have so much experience. Countries look to us; we don't look to them. Tell me which country has dealt with an Ebola outbreak in less than three months? Not even the Democratic Republic of Congo. Countries look to us. There's no country in this world that I look to and say "wow." No. For us, we have been tested and have performed. Countries might have their systems, but if they haven't been tested, I can't admire them. There are countries I would admire for having good systems, but they haven't been tested.

**Interviewer:** As a technical person, is there a country you admire?

**Respondent:** No, there isn't. Perhaps in terms of financing, some countries like South Africa have excellent funding for outbreaks. The last time I checked, their budget for COVID-19 was larger than the budget of our ministries combined. Maybe that has a lot to do with their level of development. But in that sense, South Korea did very well in responding to COVID-19, but I have no details on how they managed it, so I can't tell you that they performed better in that specific area.

**Interviewer:** Thank you for sharing these insights. Is there anything else you'd like to add?

**Respondent:** (Laughs) You've taken up my time, and I can't wait to see you off! (Laughs)

**Interviewer:** Ok, thank you once again!
