# Supplementary material for: Resilience as recovery: stakeholder perspectives on Uganda's health system response to infectious disease shocks
Source: Front Health Serv. 2026 Jul 22;6:1888814. doi: 10.3389/frhs.2026.1888814 (PMC13437620; doi:10.3389/frhs.2026.1888814)
Supplement: Supplementary file 2 [file Datasheet2.zip › KII11.docx]

**TRANSCRIPT OF KEY INFORMANT INTERVIEW**

**Int: Thank you doctor for accepting to participate in this interview. How would you define health system resilience?**

Res: this is the ability of a health system to withstand, adapt and recover from a given shock - it could be any challenges that may come into play or disruptions into routine service delivery.

**Int: okay. could also share with us some factors that contribute to the ability of the Ugandan health system to withstand and recover from the major disruptions?**

Res: some of the factors that we know that have enabled us to withstand these shocks is the design or the structure of our health system; the structure of our system is such that it has been developed with a strong primary health care foundation and this was largely before going to the community level with our policy of having primary health care facilities in terms of infrastructure and public facilities at all levels of care starting from initially where we had health Centers 2, 3 ,4 up to general hospital , referral - So the structure of the system was such that we have a strong foundation, regardless of the gaps - but the points of care and access in terms of primary care or first contact, we have a strong foundation. I hope that is clear - and on that we built a community health system. By the time when we started with Ebola, we were largely using the village health teams; so involving the community in the delivery mobilization for health and participation in health through the village health teams was also another important area of foundation that has enabled our system to be resilient and ensuring that these primary health care facilities are managed by qualified staff. As I said, there are gaps but at least I know that you expect to find a nurse at the Health Center 2; clinical officer, midwives at Health center 3 going upwards.

And another thing that has helped us to have a resilient health system - but - so by that policy where we were driving at having a public health facility starting from initially parish level as a health center 2, sub-county 3 county 4, we've been able to increase geographical access; at least the National Household Survey results showed at least 86% access within 5 kilometer radius of health service delivery - and this also includes the public private partnership, because we also promote private facilities of providers to provide care. So that also is another thing that is another factor that also contributed in terms of access to services.

The second thing I would say is integration. We started with the issue of integrating services into primary health care a long time ago; for example, some of the key public health services on interventions like immunization were integrated at all levels of care, so that people can readily access them. So there is some level of practicalization but the way that we had the delivery of a minimum health care package which was the best health care package starting from primary level, that also has helped build a resilient health system in that services are more or less the same - If you go to a Health Center 2, 3, or 4, the packages that are being provided should be more or less the same. So that aspect of also having a minimum healthcare package rolled out across the country though with different service delivery packages at different levels, are also factors that have contributed to a resilient health system.

In the area of governance and regulation, I would like to acknowledge that having the ministry taking lead in developing of standards and guidelines - because whether you are in the public or private sector, it is by policy that we try to adhere to the same standards - service delivery standards and guidelines. So that is also another aspect which is nationally led.

Another thing as a factor that has contributed or made our system more or less resilient is that I think around 2010 or so, the ministry of health emphasized the issue of having one M and E plan and through that one M and E plan, we made sure that we try as much as possible to have the data - health related data more or less harmonized or uniform and having a national health management information system that is government led. So with that, it helped us to have the data - but the data may not be a hundred percent accurate because of various challenges - lack of tools, capacity, and all that - but at least a government led M and E system with a strong surveillance system - a quickly surveillance system built helped us to ensure that we are able to to capture information and have reporting in through one channel - it may not be perfect but at least a government-led M&E system, is also another factor that I would say has contributed to that.

Another factor that I would look at is the issue of partnerships - strong partnerships- I talked about the public private partnership, but also partnerships with the development partners. If I compare with other countries, you'd find that a partner has come and takes on implementation of some of the program areas there. But here in government of Uganda, our focus has been on issues of implementation- we need to collaborate to coordinate and in enable partners support government. So the issue of partners directly implementing is something that is maybe there like I said is not a hundred percent but to a large extent, would ensure that withclear partnership, there is collaboration, there is coordination, there is a memorandum of understanding that are developed to be able to get partners to implement and also have structures through which governance structures through which we operate as different partners. For example we have the Health Department Partners Advisory Committee at the Ministry of Health; that's a structure through which government development partners, civil society organizations, private sector representatives, at least, are able to sit and we discuss and that's a very good foundation that also has helped our system to be able to withstand some of these shocks - having a strong, a relatively setup structure for collaboration, communication, engagement. I can stop there for now.

**Int: Thank you so much, Doctor, for sharing that. You've also talked about community engagement. could you tell us more about the role of the community towards resilient health systems?**

Res: You know the community are the users of health services - and as users if there's any outbreak or new case, they are the ones who feel the impact. So, for us as a sector, as a ministry, we are service providers and they are the users. So, the users must be involved and know and they are the ones actually who raise the issues which come up and we respond to the issues; so, as clients, as users, they are the ones who actually make us act because we are responding to issues that are affecting their health. Did I get your question clearly?

**Int: Yeah, because you had talked about community engagement. The community is involved in the resilient health systems. So, I just wanted to know their role. And I think you have tried to explain.**

Res: No, no, there are different roles, but we also have the community as users but we have also engaged communities to promote health and this is through like village health teams where you pick people, build their capacity so that they can support in community mobilization and engagement. So as a link, they are the users but there's also using the community as a link to community members or community structures as a link to the community.

**Int: Okay - briefly going back to the factors, is there any factor in relation to resources, in relation to the equipment?**

Res: Pardon?

**Int: You have shared factors that contribute to the ability of Ugandan health system to withstand the major disruptions. So, I'm just inquiring - are there any factors in relation to resources that could be contributing to that ability?**

Res: of being resilient?

**Int: yes**

Res: Well in terms of resources - obviously we don't have adequate resources and that is a challenge especially when it comes to major - major [outbreaks] like we had the pandemic - the COVID-19 pandemic; that one- as you saw, we had to run here and there- the impact was global and so it wasn’t for only Uganda. But I would like to state that much as we had that, because of the way we use our little resources especially the primary health care grant, we did not have much disruption in service delivery and this is because of decentralization. Our budget is centralized such that once such a budget is passed and resources have been allocated for example to health facilities, It's not easy to come and say ‘now, because of the pandemic we are not going to provide those resources’; so much as we had COVID, we had Ebola, resources for primary health care service delivery have not been cut to address those epidemics because the budget has already been passed and you cannot now go and stop that budget. So, what happens is that government has to look for resources elsewhere to be able to address [those challenges]. So, the structure of our financing for primary health care services modality is and the funds being transferred directly to health facilities is a class on that side. But the challenge is the financing- availability of funds to respond; we don't have an earmarked budget to respond for example to an outbreak or an emergency; we always have to again sit and think through ‘Where does this money come from?’ - and therefore, there are delays there. There is no earmarked funding - that's the big challenge for responding to emergencies. You have to wait either partners to come in or again for Parliament to sit and re-appropriate resources. So that is one of the biggest challenges we have.

**Int: okay - moving forward, you have talked about some interventions that have been taken that have contributed to the resilience of the health system; any other interventions or actions that you would share with us?**

Res: Interventions that are being taken?

**Int: Yeah, interventions that have been taken which have contributed to the resilience of the health system.**

Res: I thought I had exhausted them. Unless you have something that you want me to mention.

**Int: You have shared a strong partnership with the development partners; you've talked about the M &E Plan. I'm just inquiring about any other interventions on ground that have been taken.**

Res: No, some issues are like capacity building - I mean human resource. I think one which I would say was a reaction to COVID-19 - although it was not only for health workers, was for government to appreciate that remuneration of the human resource is very, very important and now there was enhancement for scientists and also health workers who have been benefited which kind of is a motivation. But to know that monetary motivation is not enough and as people get more money, then their demands increase. So we appreciate that also the issue of data remuneration of those workers can make people stay because even now, when you advertise for those posts, there are very, very many people now who are interested in coming because of the slightly better pay. But still, we have very, very big challenges.

Also, for more resilience as Ministry of Health we are glad that Ministry of Public Service went ahead and approved the new structure - because we are operating on a very, very thin structure. So if our structure is tough - if we get wage - the problem is wage - if government is to provide wage and we recruit more health workers, that will make our system more resilient because we already have gaps that we realized were already existing in our structure; for example there were no epidemiologists, intensivists and all those things and because of the lessons we learned, we have been able to rethink of what kind of structure we require looking at now, the trends - the global arena trends - so the issue of human resource structure being reviewed to take care of the current needs, disease trends and technology is also another thing that our government has tried to do; but we are still not yet there because wage is not yet provided to recruit or implement that new structure.

**Int: okay. And then according to you, what are the characteristics that make a resilient health system? You have shared what is on ground, but to you what characteristics make a resilient health system?**

Res: A resilient health system is a health system that has been built and strengthened along the health system’s building blocks. It must have strong evidence or what I would say Information system for evidence generation - okay? - so, it should be able to promote research and if there's generation, to inform policy and design of the health system.

A resilient health system must have the appropriate technology in place. You saw we suffered a lot because we didn't have ICUs in place, oxygen not there. So, you must ensure that the inputs that are required to provide services and equipment are in place and available across not only in limited areas. So you must ensure that whatever is needed - the inputs- invest in those inputs and technology that is required.

**Int: okay, thank you so much for sharing that.** **In relation to the characteristics that make a resilient health system - to you, what do you think about the different characteristics about the Ugandan health system? What are some unique characteristics about the Ugandan health system that work towards making the system more resilient?**

Res: We require an efficient system. So, issue of embracing technology digitization which can facilitate issues like communication, information - timely information sharing - that one has to be embraced.

Also, for a resilient health system you need also leadership and governance - so strong leadership and governance including political support. They are very key in ensuring that you have a resilient health system. So, the issue of leadership - strong leadership and governance, political support is very key for investments to come to the health system.

**Int: Okay - so how about in the Ugandan health system context? What are some characteristics about it towards making the system more resilient?**

**Network is an issue. So, you have been sharing challenges, obstacles. You've talked of challenge on capacity building, resources, financing. Are there any other challenges that affect the health system’s ability to withstand and recover from the shocks?**

Res: We need some arrangement because I don’t know whats happening to my internet.

**Int: okay, let's summarize this. You had talked of some challenges, and I was asking, what are the other challenges that affect the health system's ability to recover from infectious disease shocks?**

Res: You are repeating yourself; I think I've talked a lot and some of those things you need to address them from what I have said because the Internet is not good at all. I beg your pardon; ask your question again.

**Int:: Any other challenges that affect the health system's ability to withstand and recover from the infectious disease shocks apart from those that you shared? you talked of resources, financing, human resource - in case there are any other challenges Doctor.**

Res: Okay. So, you are asking what? I don't know what is wrong with this internet; it's going on and off.

**Int: I was saying that you have shared some of the challenges and you talked of the resources, finances. Then you talked of the challenges in human resource and I was asking if there are any other challenges that affect the health system's ability to withstand and recover from infectious disease shocks apart from those that you had earlier shared? hello?**

(no response from the interviewee due to network failure)

**Int: Yes, how can stakeholders work together to overcome the challenges that you earlier shared?**

Res: How can stakeholders work together?

**Int: Yes, how can they do that?**

Res: There must be forums - there must be stakeholder engagement - forums for stakeholder engagement. There must be a lead in all this and here it is government led. You must know all the stakeholders; so, you must have various fora for stakeholder engagement either through meetings, through dialogue, through guidelines.

**Int: As we come to the end of the interview Doctor, let's look at recommendations. What strategies would you propose to strengthen the health system and improve resilience to infectious disease shocks?**

Res: What?

**Int: What strategies or initiatives would you propose to strengthen the health system and improve resilience to infectious disease Shocks?**

Res: Okay; so one of them- the strategies, is first of all to ensure awareness - Yeah - there must be a strong system to create awareness about infectious diseases and respond to them; a strong surveillance system to be able to detect and report so that response is in time. Okay - so there must be a clear resource mobilization strategy to be able to respond to these shocks - there must be a clear system of regulation; who does what and where, and when? There must be also a strategy of integration into the existing services for us to be able to utilize the little resources we have optimally including capacity building of the human resource so that they are able to also detect and report.

We must have a strategy or another strategy of also community. first of all, also a a strategy for strengthening multi-sectoral collaboration and coordination not only between sectors but also with the partners. So, a strong multisectoral collaboration and engagement strategy should be in place.

**Int: And how can VHTs be better supported or empowered to develop a more resilient health system?**

Res: You know VHTs are volunteers and the rate at which we are getting these public health shocks- infectious disease outbreaks, we are tiring them a lot! So, much as we are talking of strengthening the community health structure - yes in our strategy, even the National Development Plan for that is going to be implemented for the next 5 years. The issue is to continue with their capacity building in terms of training because you never know what type of infectious disease comes up next. We have these which are on but there may be others that are not; so you have to continue with capacity building.

And also providing them with the necessary tools to do the work. We are talking of infectious diseases yet these VHTs may not be trained. So, you have to make sure as you delegate or do task shifting, they have the appropriate PPEs to protect them because if the VHT died in the in the line of fire, they are not protected - there is no compensation because for them as workers they are not formally employed by government. So, you cannot even go to the Ministry of General and say, ‘Oh, a VHT died in the line of fire’; how can we compensate them under the Occupational Health and Safety Act?’ So those are some of the things we even need to think through. As you say, VHTs coming into the front line - the line of fire when we are talking about infectious diseases. Their initial involvement - initial strategy for VHTs was largely for community mobilization, sensitization and awareness but now we see that even when it came to COVID-19, to some extent we are saying that can VHTs now go - they take temperature, they take measures thermometers, and they do this, do that- putting them in the real line of care. So, we need to have a proper review of our policies. If we are putting VHTs in the care and treatment of the arena of infectious diseases, we must have ways of how they are going to be protected. There's no policy on that. So, we need to think through that as well. Task shifting to what extent? How are they covered in case of this and that? To what extent can we task shift?

**Int: okay. And then, lastly, doctor, when you look at the successful models, are there any that you think would suit the Ugandan context better?**

Res: Which successful models?

**Int: The models available if they are there in relation to the health system resilience.**

Res: I am not aware of any models because that's not my area of specialty. Because when you ask that question, I don't know in what context you are asking, but what I know that even the developed world where they had health systems which are said to be strong health systems, the effects of COVID were felt across the world. So, when you say which model, I don't know in what perspective you are talking about because each country has to adapt within its context in terms of your developmental capacity and in terms of other factors. So maybe you could share with me some of the models that I can go and read about them that you're aware of by the way, which worked?

**Int: I think Doctor, I will tell XXXX to elaborate more about this question. If you don't mind, he could get back to you.**

Res: Where did you send the consent? I don't see that consent form on my email.

**Int: Let me send it now Doctor. Thank you so much for your time.**

END
