# Supplementary material for: Resilience as recovery: stakeholder perspectives on Uganda's health system response to infectious disease shocks
Source: Front Health Serv. 2026 Jul 22;6:1888814. doi: 10.3389/frhs.2026.1888814 (PMC13437620; doi:10.3389/frhs.2026.1888814)
Supplement: Supplementary file 2 [file Datasheet2.zip › KII12.docx]

TRANSCRIPT OF KEY INFORMANT INTERVIEW

Key

Interviewer: I

Respondent: R

I:
Good afternoon, my name is Geoffrey Mutole, and I'm conducting a key informant interview with one of the stakeholders at the Ministry of Health, and we are trying to understand the concept of health system resilience, determinants that influence building a health system resilience to infectious disease epidemics in Uganda. So, we're starting the interview around 1:32 pm, I think?

R:
Yes, yes, 1.32.

I:
Yeah, so we have different themes to discuss about, and one of them is health system resilience definition. So different stakeholders have come up with different definitions, they have been able to define the health system resilience. So, to your knowledge and experience, how would you define the health system resilience?

R:
Okay, resilience to me is absorb shock and provide routine services without being interrupted by things like outbreaks or public threats. Okay. But that is the simple thing I have. Simple. Yeah, you can absorb shock in an atmosphere where there is a threat or there is an emergency, so that the other services don't stop. You continue providing the routine services. Those people who need to be immunised, those who need to deliver can deliver properly. Those who need to go to surgery, they can also go to surgery. If somebody has an acute abdominal injury. You cannot say, ah, we are in crisis. So that's what I mean by you can absorb shock while continuing with the normal. But in detail, it is a big issue. Because resilience, it doesn't just come like that. You have to build it, and usually we build it through preparedness. When you prepare, and there are many ways of preparing. You prepare in terms of skills, in terms of competences, in terms of capacity, in terms of logistics, in terms of policies, in terms of laws, in terms of infrastructure. So, this preparedness is very, very important. To prepare for any shock which will come, but will not deter you from providing the normal services. But also, that preparedness means that you can respond very quickly, so that you don't hurt people. Or those people who are affected can be treated so that they don't die from that problem.

I:
So maybe you could have mentioned some of the key things I wanted to enquire more, but you can still re-echo. So according to you, what makes a health system more resilient?

R:
Okay, as I had said that we need to be really prepared in advance. First of all, we must know what are the common public threats. If you know them, then you can prepare better. If you don't know them, they find your pants down. But if they come when you know them, and you are prepared for them, that is a very important thing. You can even model and see what are the likely threats, so that you prepare in advance. You can also put the necessary logistics in place, the necessary countermeasures in place, the necessary competence in place, and of course the human resource. So human resource is very important, especially competent human resource can make your system very resilient. And of course, also the systems which you put in place, guidelines, protocols, procedures, plans, all those make your system very resilient, because you don't panic. For example, now for us here in Uganda, you bring even Ebola now, we can't panic. We have the plan, we have the people, so we are ready. So that's really resilience.

I:
Okay, thank you so much. Try to reflect back to what Uganda has been going through, those outbreaks. So, do you think Uganda was or is still ready to, just in case those outbreaks reappear?

R:
 Unfortunately, every outbreak is different, and every outbreak is a new thing to learn from.

So, we cannot say that really, we are ready, but at least if you scale 1 to 10, I can say we are over 70% ready.

I:
So we have some systems.

R:
 Yeah, we have systems in place, or we have strength in place. Like we have the National Task Force, which is a strategic level, it can give guidance. We have pillars, which are at the operational level. We have even decentralised data system to the district. We are even now enhancing regional capacity. We have put the regional EOCs to really build capacity at that level. And in any case, at that regional level, there is capacity there. So, we have to utilise it. We have to utilise it. So those are some of the things we are building to improve. But of course, we have a long way. We have a lot of data, which we have really to use and make decisions, programming, planning, so that we can do better. And that's why now here, there is a track called informatics. People can handle big data and come out with information which is actionable. So, all of this is really building. So, I cannot say that we are 100% resilient.

The first of the other things is that we are not resilient. Especially we are so vulnerable because we depend on outside funding. We depend on outside funding. And outside funding, or partner funding, as you know, is very vital. Anytime it can disappear, as it has disappeared. So now, we are now very vulnerable. Although we have just done, we have just kicked out Ebola now. But I think that was because we were, already we had some basic systems. And of course, the other thing which also makes us very resilient, that we have good working relations with the partner. So, partnership is also very important. Because we alone, we may not manage. We may not have all the capacity. We may not have all the technical knowledge. We may not have all the resources. We may not have all the money.

So really, partnership is very, very important. So, but as I say, we have a lot to do. And even our information, sometimes the early warning system is not very good. Because we are not well digitalised. We need to digitalise all our health system, e-health. We are also trying to build that. For example, our surveillance officer, we don't have sub-national surveillance officers. We have people who are assigned. So, for us to be more resilient, we need to have district health surveillance officers as an established position. Because it's so important. As you know, these public health problems start in the district. But if there's nobody there to really look at them, these people, they have their primary duties. They send you, now you're the surveillance officer, but you have your primary duty. So, you can see that what you give to surveillance is minimal. So that makes us a bit vulnerable at that level.

I:
Now that takes me to my next question, which I feel you have answered part of it. So, my second question looks at factors contributing to health system resilience. So, to your perspective, how would you describe Uganda's health system resilience? And what factors do you think contribute to the ability of Uganda's health system to be resilient? Both at national and sub-national level.

R:
Okay, let me say finance is one of them. As a factor. Financing health systems. Specially to build primary care, secondary, and tertiary. Those are really, we need finance. And I think that's one, Of course we have the Ministry of Health. That's a very big strength for us to make the country because they are dedicated to the health of the population.

I:
So having a Ministry is one of the key factors.

R:
Yeah, one of the key factors. And then within the ministry, we have a department of... It's called the Integrated Epidemiology Service and Public Emergency. That also is a big strength for us. So, it is important that we have such structures in the country. Because they can dedicate their time. Make sure that they can monitor the health status of the country. Because we need to monitor it and see what are our biggest problems. We have an approach called the Integrated Disease Surveillance and Response. That's an approach where we really look at the country's health problems. The emerging and the re-emerging and the endemic. And so that we can put a very big plan for them to control them. And of course, ...

I:
 So in terms of human resource, building a competent human resource Is a critical factor in the resilience?

R:
 As I told you, if we bought Ebola now, we can't run away. Because we have the competence. So that means we are resilient. But before we did that, you would see everybody fleeing. People passing through the wind. But now, I think we have that resilience.

I:
So when you mentioned building a resilient human resource, how would you build it? Or what do you mean by building?

R:
That's what I'm saying. We need to impart competences, skills to manage some of these common emergencies. Or common public health threats. So that you know... Even those which we don't know. Because sometimes we used to have... Before our surveillance improved, we used to call them strange diseases. Because at that time we didn't know what we were dealing with. Then the strange disease is here. People had no idea about them. But as our surveillance improved, and our laboratory... So, the surveillance system is very, very important to create a resistance. And that includes laboratory. Competent laboratories, of course with competent people in the lab. And that one we are very strong. Because we have the Butabika lab. We have a network of labs. In the regional hospitals. Even in the private. So really, all those are very, very critical in resilience. And maybe the other thing which is also very important is the community. The community is so critical. If you don't involve the community, you have missed the bus. The bus will leave with you. Because, as you know, the communities have two major functions. They can trigger an outbreak when it emerges.

Because of lack of empowerment. Or they can help you to control the outbreak. Because of their empowerment. And for us here, we are very strong in the community here. Because we have what we call village health teams. These teams are helping us in maybe three things. First of, they can educate the community. Two, they can report emergencies. Three, they can also help in the response. So really, they are very useful. But what we need to build more now, to make us more resilient, is to catch up with this idea of CHEWs. Community Extension Workers. These are paid workers. But that's our challenge. Because this one day, they can account for the action. But these village teams are volunteers. You cannot take them on action. You cannot, you know, because they are volunteers. They have their own responsibility. They have families. So, you cannot really take them on that account for this.

But if there is somebody who is being paid, like the CHEWs, Community Extension Workers, which is again something we are trying to build, it can really help the resistance in the community level. That will be very, very important. So, community is very, very important.

And also, we need to build our early warning system, including the information system, so that we can share across sectors, build what we call One Health platform. We have already One Health platform where we work because one sector is not strong enough to, because when an outbreak is manifested, it can affect all sectors. So, we want to work with all sectors. So, One Health is the way we use to, is also very important. One Health is a very, very critical issue for resilience, so that your preparedness and response is inclusive. Nobody is excluded. The private is there. The private is there. The sectors are there. The non-state actors are there, like the NGOs, so that this becomes, you know, those pieces, you put them together, they form a solid resilience team or group. And so, the One Health is very, very critical. The information is very critical in early warning system so that you can catch these public health emergencies when they are just beginning. And this helps us also comply with international regulations because we are signatory to IHR, International Health Regulation 2005. One of the provisions is that we are obliged to build and sustain core capacities for response of diseases so that we don't see that diseases spread across our international boundaries. So, we prevent international spread of disease by controlling the outbreaks at source. And here we have been very good, controlling outbreak at source so that they don't cross our boundaries, our international boundaries. And that means we are trying to fulfil the provision of our IHR, international where we are well subscribed. So, you can see that all of those make us very, very strong.

I:
Okay. Thank you so much, doctor. I don't know if, have you heard about the six building blocks of health systems?

R:
 Yes.

I:
So I know you mentioned some of them as factors that contribute to building a resilient system. So, what is your comment on leadership and governance?

R:
 Oh, yeah, I think I don't know why. That one is really, today, is so critical. Leadership is very, very important. And one of our lukewarm problem is that some of us, we attain leadership position without being orientated. And that's terrible. They just push me from district, or collect me from district X, and they put me here to lead the national leadership programmes. And yet, I don't have competence in leadership. You know, leadership is made up of two major things. The hard skills and the soft skills. And most of the time, we may have the hard skills, but if you don't have the soft skills, it's very difficult to be a good leader. So that leadership is very, very critical. Very critical. And of course, leadership coupled with coordination. Because if you don't coordinate the stakeholders, your kind of even waste the resources. You even don't shoot in the same goal. So, it is very, very important. So, I think one of the things which we have realised, I think they won't realise it in a lab. Because now there's a track here called laboratory leadership. And these people are doing really, equipped with the skills to be leaders in the lab. But also, we have a programme here called PHEM. Public Health Emergency Management Programme. Where we train public health officers in, again, leadership. And leadership is very, very critical. Because, as you know, the legs can take you anywhere if you don't have a head. So, head is leadership. So, the leadership will tell you, the legs, where to go. But if you don't have legs, you can be a chicken which has been slaughtered and has no head. So, leadership is one of the critical things and thank you for pointing it out. It's very, very, very, very important. And to me, I think I can say that, I think that's, to me, that's my skill. Leadership in public health is critical. Because a leader is the one who creates the future. If you don't have leaders, you can't create a future. So, it's very, very, very critical.

I:
How about the medical products, vaccines and technologies?

R:
 Of course, those are the countermeasures I talked about. And here, I want to bring the countermeasures or the products in a form of what I call transformative capacity. That means that you learn. You learn your weakness in the previous outbreak. And so that when the next one comes, it doesn't find you in the same position. You are better off. That's what I call transformative capacity. And here, we have done it in Uganda. What we have learned from COVID, we were importing PPEs. But nowadays, we manufacture them. That is transformative. We are importing all vaccines. We are starting to make vaccines. That is transformative capacity. So that again makes us more resilient. So that is, so the products, the countermeasure products are very important. And health supplies are very important.

And also, of course, this goes with a proper chain, a supply and a chain management system, which really helps us to make sure that the products are in the right place, the right amount, and the right time. So really, the countermeasures are very, very important. But I wanted to bring them into the concept of transformative capacity. Do you have that capacity? If you don't have that capacity, that is, we build it. Like now, we are trying to build the vaccine capacity. We are borrowing, you know. We are asking people who have the capacity to transfer technology so that we can also build it. Although some of that, and others, gloves we are making. So that is what we, so that now, next time, it doesn't find us in the same position that we didn’t have.

I:
Okay. Thank you so much, Doctor. Our next theme of discussion looks at interventions contributing to resilience. And my question is, what interventions or actions have been taken within Uganda's health systems that you believe have contributed to its resilience, particularly in areas of disruptions, like when there is an outbreak?

R:
 Okay. I think one of the Interventions we have taken is really to build systems and structures. And here, what I mean by structures, structures, response structures. Response structures. Structures like National Task Force, District Task Force, and, of course, the village health team. The structures here for response, and maybe prevent, they span from the office of the president to the village. So that really is making us very, very strong. Because, as you know, whenever there's a problem, you can see the office of the president, how it comes out to give risk communication to the public, the do's and the don'ts. So that makes the population very resilient. And, of course, the other thing, again, which makes us very, very resilient is, as I already said, the community-based surveillance, which is really driven by the VHTs. That also makes us very, very resilient. And the other thing which makes us is we have a critical mass of human resource. Human resource. We have a critical mass. Of course, it depends on the problem. We are also building such capacity in human resource. So, all those have made us very resilient here. We have, and also for the products, we have mechanism of acquiring health products very quickly through arrangements which don't have to go into red tapes. So, all those really make us very, very strong in Uganda. And, of course, we have also the office of the prime minister, which, of course, as you know, is a leader of government business. They also have what they call a national reduction plan. Where they really map disasters and emergencies where they are likely to happen. And that helps us also to make sure that we focus there very much. And, of course, again, I want to say that our emergency operation centres also have contributed. Because now they can ferry information quickly for quick response. As we say that, I always tell my colleagues that driving at high speed is very dangerous, but driving at high, moderate speed, which is regulated in emergency, saves life. And, by the way, one of the things is to really say you must respond quickly. Respond quickly. Quickly or speedily. We have seen countries which are well prepared, but because they didn't respond quickly, COVID showed them, they have more deaths than we have.

I:
The likes of?

R:
Those European countries. They are better prepared. But you see their death rate and look at ours.

I:
So, in your point, you're saying their response was a bit delayed.

R:
Yes, delayed response. Although you are prepared, you can be well prepared, but you must respond spontaneously. As soon as possible. So that's why these structures, I'm telling you, the EOCs, they can be activated within two hours. And that's quick response. That's quick response.

I:
So there's a team handling that?

R:
 Yes, there's a team handling that as well. So that really can help countries to respond. And you prevent preventable deaths if you respond quickly. So, I think those are some of the factors.

I:
 Okay, thank you so much for all that information. I did not hear any policy that highlights any policy that has impacted in strengthening energy health systems. You've been mentioning about strategies, what's the programme address. Is there any policy?

R:
 Yes, we have just amended our public health act. And this has really also strengthened our management of infectious diseases in the country. Because one of the important things there, which I like, is that everybody is supposed to report infectious diseases. So, if you, and I find that there's an infectious disease in your homestead, and you don't report it, I can take you to the police. According to the law, according to that act, I can take you to the police.

I:
So that is its mandate now?

R:
Yes. That you know all of us are surveillance officers. All of us. You and me. It starts with us. So that is also something as far as the policy is concerned. Of course, we have our health policy. And the threat there is health promotion, really, and disease prevention. That is the threat. Although our politicians, they want to build health facilities. No, first of all, they can't. They don't contribute much to our health status. But it's the prevention and the promotion which really help. And as you know, they're saying that health starts at home. And it's only been reared in the garages of hospitals. So really that still holds. And that's what the policy is saying. If we could really improve our nutrition, our hygiene, our homestead health, I think the whole country can be resilient. So those are some of it. So those are some of the weaknesses highlighted.

I:
So any weaknesses in those policies and interventions that you could identify.

R:
Yes, you know, again in Uganda as you know, we are very good at making policies but we are very weak in enforcing them, that's the biggest weakness, that's the biggest weakness, and also the other thing maybe is integration, we are weak.

I:
Integration is weak.

R:
We are weak, we need to integrate and even integration is very good because it saves some resources and also it ensures ownership, so if you don't integrate you really, you become a bit weak because in integration you kind of, your strength can leap, make a big leap forward to integrate because you bring all your strength and look at that. So, in integration we are still very weak, we are very, very weak and of course again like you are seeing that our finance, financing is so weak because as you know there is this Abuja declaration where the African states committed to fund finance of about 15% of their GDP. But for us here in Uganda we are only about 3% of our GDP and WHO recommends about 6%.

I:
So for us we are below the average.

R:
We are below, far too below, far too below and then this again, it has a repercussion, we are one of the poor countries in the world. One of the poor countries, but the expenditure out of it is very high, over 30% of what people spend on health and that aggravates, that weakens our resilience, weakens our resilience, so we want to work on some of the factors, especially as you know health is made of many factors, so we need to look at those socio-economic factors to make us, you know, educate people, people should go to school, people should have incomes, people should have jobs, you know, all those socio-economic, but the good thing here, one of the things is that our family, our family models are very good because usually if the government cannot come, at least our family can bring food, they can even go and buy the medicine, the doctor can prescribe. Because of the family, we don't have nuclear family, we have extended family, the uncles, the jajas, can respond. So that is a factor for us which is natural here, which can also make us a bit resilient. But of course, it depends on who is, you may not have a jaja or an uncle, you are on your own. So really those are some of the things, and of course access, access is not very good, we are weak in access, because access to health services is not uniform, access in rural areas is compromised. One, the facilities are not up to standard, some of them are dilapidated. Two, the health workers are mildly disrupted, the good ones are here, so if you are in Kapchorwa, my friend, you don't have a good doctor. So that is a weakness we have.

I:
 So that means if an outbreak happens, it can spread in the whole of place.

R:
So this really, to me, is very, very important. But the issue of financing there is very bad. And by the way, it may not be here only, most of the means of financing of the ministry of health the whole world, world over, they are always underfunded. Because they see it as an expenditure, not as an investment. That's the bad thing about it. Many politicians think that you are just spending, but health is an investment. So really, to me, that should be a very big priority.

I:
Thank you so much, doctor. You are welcome. Our next theme is going to look at the attributes or characteristics of a resilient system. So, could you paint a picture for us, what a resilient system looks like, particularly in response to infectious disease outbreaks?

R:
 Okay. One, I think I would say, you must be preparing all the time, continuous preparedness. And of course, knowing what are your problems, building a competent human resource, engaging the community, increasing access to good care. Like we should build universal health care or primary care. We should have a strong primary care or universal health care, so that that can make us resilient. Of course, finance, I've already said, we need to increase our domestic financing, so we don't depend on that. Also, we need to, of course, to digitise our health system so that we can make informed decisions according to the data we have. And of course, I talked about transformative capacity, and also, of course, decentralising health in sub-regions is one of the characteristics of our system. It can make us more resilient. Okay.

I:
 So maybe I'll also want to enquire more. Are there any specific examples from Uganda or any other country that you think demonstrate these attributes very well, as you've just mentioned?

R:
 Yeah, like, let me see. Like in Ethiopia, the community components are very good.

I:
How?

R:
Because they have what they call the CHEWs, and in fact, that's where we borrowed this idea from. For them, their community surveillance is very good. And to me, I would take that one. But for us here, I think decentralising health system is also a strong point for us here. And especially now, we are enhancing regional capacity. Because we realised everything was on the centre. But in the regional area, the capacity is there, but it was being underutilised. We have consultants there. We should use them. So, we want to make sure that we kind of build capacity in the regions. And of course, the district. So, to me, those are very, very good examples.

I:
Okay. I still want to hear that from Uganda. If it be specific of those building blocks in Uganda, which one do you think are working better? And which one do you think have some loopholes that we could, that need support?

R:
In Uganda, for example, if I tell you about the human resource, we are not retaining many of our health workers. So that's why we are always training, training because they are new people. So, the best way is to recruit and retain these health workers and of course motivate them. Motivate them. Although the government is trying, you know, now I think the health workers are being paid better. But again, the pay is not really up to the mark. But at least they are aware of the problem. So, motivate them. But there are other ways of motivating. Like during Amin's time, people used to get cars without, you know, you don't pay tax, but also, they give you the car and you pay slowly, slowly. Its motivated people. So, we must find a way of motivating so that people don't leave. The good people are leaving. They go to WHO, they go to Africa's CDC. So, they are leaving. When there was Ebola in West Africa, do you know that we lost many people from here? And some of them have been offered citizenship there. Their citizens of those countries now. So, it's because we don't retain our people. We don't motivate our people. So that is also we need to look at that. Of course, as I said, the government is trying to pay the scientists more salaries as a motivating thing. But there are many other things. I think the health sector itself can think of other innovative ways of motivating, you know. I think really, we need to motivate, especially the distribution. The distribution. During my time, when you agreed to work in Karamoja, the chances of getting a scholarship for postgraduate were very high. So that was a motivator. So, if I work there for two years, I want to get a free postgraduate for nothing. So, we have to find ways how we can motivate people so that they can go out there to stay here. So that's really some of the things we need to look at. So, we need to motivate our health workers.

I:
So in your perspective, you're saying the element of human resource in Uganda has not been to the standards. So which ones do you think that we can be proud of? Among these components or these building blocks, which one stands out in Uganda.

R:

There's nothing that really stands out here in Uganda. Everywhere we need really improvement.

I:

There's still support needed in each of them.

R:
Yeah, everywhere we need support.

I:
Okay. That takes me to my other enquiry or question. So apart from the building blocks, how does the structure, culture or other features of the Ugandan health system set it apart from other health systems when it comes to resilience?

R:
 Okay. One of the things which Uganda is the national health service, which means a free health service. That really is very different from other countries like America, where for you, if you don't have money, you just die. But here, you can go and get help. So, the insurance of the population getting free health services is really quite different from other countries. Of course, many other countries do have that. But that is something, again, which I think the government takes on, which it cannot afford. So, we looked at that and we kind of developed what we call essential health care packages.

I:
 Essential health care packages?

R:
Yes, which is a must. That if you go, at least that package should be free at the point of delivery. You can put in transport, you can do what, but when you reach there, it should be a free service. But you remember we also had the service for free, or fees for service, which was abolished because people could not afford to pay fee for service. So, I think that free insurance for people to get free services is quite different from other countries. And the other thing also, the National Task Force is something, again, which is quite... Many people have, by the way, adopted that. It's quite different from many other countries. Because for us, even now, we have added there a scientific committee as part of the National Task Force, so that when we respond, we are responding scientifically, using science.

I:
What does this committee do?

R:
It guides us so that what we do is most scientifically sound. They look at things... The problem, it's manifested, maybe. So that is also something which is very unique. The other one is responding in pillars. Because we have about 11 pillars. Surveillance, lab, case management, risk communication, community engagement, and social protection. All those pillars. Mental health and social psych support. All of those had a pass to really be a bit more different from other countries. And it is very, very good, because you have a dedicated team on specific themes. Like if surveillance, there's a team dedicated. If it is case management, there's a team dedicated for that. If it's the lab, there are many things in the lab. There's specimen transportation, there's work. So, all those, you find that we have dedicated teams, which have the capacity and the experience and the competence to do that.

I:
So I want you to highlight an example of a country that you think you're knowledgeable of their health systems, and we compare with our health systems here in Uganda. Which country would you highlight? Should we use our neighbours here? Rwanda? Kenya?

R:
 Well, Rwanda, for them, the e-health is very good.

They have e-health. They have digitalised the health system so well, that maybe they are better than us there. But other countries……

I:
 I want that country to really understand their health systems, how it is structured. Then we do some bit of comparison with our country. We see where we are performing better and where we are underperforming. And maybe we'll see what can be done to improve these other pillars.

R:
Unfortunately, many countries come here to benchmark.

I:
Really?

R:
 Yeah, as far as West Africa, they do come here to benchmark. But, as I said, for example, maybe we can take......

I:
 I was told Rwanda is using the community VHTs is a strong pillar, I was told.

R:
Yes. I don't know whether they pay them. But as I told you, this is a very strong pillar for health. But I cannot tell you, I don't know any country in our neighbourhood here. We do better, like for example, in Kenya, we are better than us in managing antimicrobial resistance. We are better in managing public health emergencies. So, each country has Yeah, has its own strength. But you cannot judge.

I:
Right. That takes me to my next section. Which I think you had highlighted, but you could still re-echo for clarification. What role do communities play in building a resilient health system?

R:
As I said, they can only play a role if you empower them. That is, if you give them the necessary equipment and knowledge. So that they can themselves take care of their own health. So that you know that health is the responsibility of an individual. So that is the main starting point. And then of course, collectively, to make sure that the communities can build their own structures. I know communities which say, we want to build a health centre here. Or we want to build a well here. So that we can get clean water. So that is community action. And also, as we said, the community can report incidents or events which are beyond their control. And they can report them so that they can get support. Either from the district or from the centre. So, reporting, recognising and reporting events is very critical. And also, to have community structures like the VHT. Which can pick up, which can play a part in mobilising communities. In educating them. In helping us to respond. In spreading, even policy issues. Even strategy issues and planning issues. So really, that is really to me, very critical.

I:
Thank you so much, doctor. We have had outbreaks in Nakasongola. Was it Mpox or Marbarg?

R:
Yes, Nakasongola.

I:
Then we had Mubende, we had Ebola. All those outbreaks. But I think even in Kampala also. Could you describe an example where a community involvement or engagement Helped the health system to recover or respond effectively to this crisis? Could you give an example of Nakasongola, Mubende or even within here?

R:
Okay, let me use the Nakasongola. You know, in Nakasongola for example, a fishing community mobilised themselves. To make sure that they try to control Mpox. Because it was a big issue there. So, they mobilised themselves and that was a good example of self-mobilisation. Now I think Mpox there now has gone down, especially in the fishing community. And by the also, I heard these people, the people who sell themselves.

I:
 The prostitutes, sex workers.

R:
They also said they wanted to suspend. So that is also a good community.

I:
They wanted to do what?

R:
To suspend.

I:
Suspend, what do you mean?

R:
 Their business for maybe two, three months. I read one of their leaders wanted to suspend. So that is also a good community.

I:
Because I actually heard how this Mpox outbreak started, it was through a prostitute who had an engagement with someone.

R:
 So those are some of the good examples of community.

I:
Thank you so much doctor. Our next theme looks at challenges to health system resilience. To your knowledge, what challenges or obstacles do you think affect Uganda's health system's ability to withstand and recover from infectious disease outbreaks or shocks?

R:
One of the things which is disturbing us, there are many emerging and emerging infectious diseases. That is a big challenge for us. And there are new ones also. That is a big challenge to us. Of course, the other one was, of course our communities are not well prepared. We need to prepare them more. So that we build a very strong community surveillance system.

Also, of course, funding. As I said, depending on the donors, it is very volatile. We need to really increase our domestic funding. Our domestic funding, there are many ways we can do it. We have been talking about health systems for years, for decades. This thing has refused to materialise. But that's one of the ways we can increase our funding for the health system. And there are many other, community financing. There are also those initiatives which we can use for funding. And of course, as I told you, the human resource. We need to distribute human resource so that we increase access to our people. We also need to build a strong primary health care or universal health care. We need to adapt technology to increase efficiency in our health system. Those are some of the things which we need. We are still very weak. We need to strengthen there.

I:
Thank you. You mentioned about the emerging and re-emerging infections or diseases.

Do you have an example of a re-emerging infection that we have had recently? A re-emerging one?

R:
Yes, of course, Mpox. Mpox is re-emerging. It's a new thing. And by the way, there are others which we hear are coming. Avian influenza, they are coming. But to hear them, they are still at a distance. But any time, as you know, these infections, they don't know boundaries. Any time, they will be here. So, those are some of the things.

I:
So, what is your comment on, for example, I just mentioned which keeps on re-emerging. What is your comment on that, regarding our health system's resilience?

R:
Of course, we need to make sure that our system can quickly detect unusual events. And then we can quickly investigate them and confirm them. And that means we have to build capacity in our confirmation system that we have. We must know how to do sequencing so that you can know really what is this. Where is it from? Then you can take appropriate measures.

I:
Okay. Thank you so much, doctor. Our second last theme looks at stakeholder collaboration in overcoming these challenges.

R:
I think that one I already told you about partnership and stakeholder engagement. It is very, very critical because everybody comes with a strength, which when you put together, you leap forward very quickly, or a big leap forward. So, it is very, very important that we have partnership and stakeholder engagement. And that is what we do through One Health. And also, we have one of the things we call whole of government approach, whole of society approach. Those are things, protocols we are trying to build, but they are not yet very strong. But these are things which will make us very resilient and strong because of stakeholder engagement and coordination.

I:
Yeah. So, who would these stakeholders be regarding building a resilient system?

R:
 Well, I think the Office of the Prime Minister and the Office of Gender and Community Development, those are the real people who can really do this for us because they deal with the communities, they deal with the public, those can help us. But of course, each of the sectors can build and empower their own jurisdiction so that all of us are. But one of the things we need to do is to what I call to align all our priorities together. So, we need to involve the partners, the stakeholders to build a plan. What do they call one plan, one budget, one report. That is very, very critical because we align all our efforts towards one plan, one budget and one report. That way we are monitoring. We monitor at the same time when we are supervising, we have an integrated supervision. So, that can also help us to make sure that we have a very strong resilient system.

I:
Okay. Thank you so much, Doctor. The last section looks at recommendations for strengthening the health system. So, the question is, what strategies or initiatives would you propose to strengthen the Ugandan health system and improve its resilience to infectious disease outbreaks or shocks?

R:
 Of course, the first one is again human resource. We need to build a critical mass of responders and we have what we call rapid response teams in the country. But also, we need to build them at the district level and maybe at community level. We need to make sure that these health workers are well distributed in the country so that they are not mal-distributed as it is now. We need to run away from donor funding so that we can increase our domestic funding and also make sure that we have other innovative funding. Some countries, I don't know which country, they put tax on tobacco, on alcohol and that goes to health. So, that is an innovative financing. We need to have good governance and leadership. Those are very critical. We should train especially the managers and the leaders in skills of leadership so that they can lead us very well and competently. Of course, as I said, we need to tap into the emerging technologies so we can increase our efficiency. Those are some of the things we need to look at.

I:
Okay. You have not mentioned about VHTs, but let me ask you a question about VHTs.

How can the VHTs be better supported or empowered to contribute to developing a more resilient power system in Uganda?

R:
No, I think we should run away from VHTs. Okay, they could be there, but we are more inclined to have the CHEWs, community extension workers, because those ones, they can account for their action because they are paid. But as I said, these are volunteers. And one of the things, me, I personally want to advocate that everybody should be a VHT member. The problem we have now is that you are a VHT member year in, year out. When will you do your work? So, we should say, I'm a VHT member for two years, now you take over. And then you hand over to another one. But to be year in, year out, sometimes, it loses and then people eventually think that this should be a job, a paid job. That's why they say, give us a reason. Are you motivated then? But I think everybody should be a VHT member in your village. You say, okay, every year, every two years, every five years, but you can't be a VHT member year in, year out.

I:
For 30 years.

R:
For 30 years you are a VHT member. No, that's not fair.

I:
Doctor, regardless still, what support do you think, whether people are changing from one to the other, what support do you think these people are needed to build a resilient health system?

R:
 I think we need, first of all, to facilitate their work. For example, if you want real-time information, buy them telephones, buy them phones, give them phones for reporting. And also, for sending messages, they can send to their communities, policy matters, communication messages. So, to me, facilitate them to do their job. Give them transport. Some villages are big. Give them a bicycle or a motorcycle. Give them gumboots. Give them raincoats. Give them umbrellas so that they can move. Give them a bag where they can put their notebooks. You know, that kind of facilitation can enable them to do their work more efficiently. But, you know, and then we can also increase their home visits. Because they have the means to move. But you can't walk from here to go to Kireka. But if I have a bicycle, I can ride there.

I:
So, meaning, if we empower them with all these finances, then they will be able to increase their home visits.

R:
No, you don't give them, but you facilitate them. Like for us, when we are going to the district, they facilitate us. They give us money for the hotel. They give us money for the food. That's a facilitation. So, they need to be facilitated.

I:
How about training? Do you think they need it?

R:
Of course, that's empowerment. By the way, that one, we do it. We do it every day. Every time there is a new thing, we train them or we orient them. For example, if there is a new disease, we train them. What are the signs and symptoms? Where do you refer them? Where do you report? Things like that. We are trained.

I:
 Doctor, our last question looks at, are there any success models from other countries or other contexts that could be adapted to the Ugandan context to enhance the health system resilience?

R:
I think one could look at Malawi and Zambia in the way they learn their resilience context.

Even Sierra Leone and Rwanda.

I:
So how do they do it?

R:
Well, they have some unique way of bringing some of these thematic areas and makes them strong. Like you said, Rwanda with the community, with technology. There they are very strong. Like Sierra Leone in resilience as far as human resources is concerned, they are very strong. So is even Ghana. Ghana is very strong in health products. So, these are some of the things you maybe can look at. You can take on.

I:
So those are the ones you have mentioned. Which one do you think could be effective and why?

R:
 Some of these things, by the way, depend on the stability of the country. Like Ghana has been very stable for a long time and therefore their systems have had very minimal disruption. So, they build it. You know, when you are unstable, you build a system and then you move five miles backwards. And so, you have to start again. But Ghana's system has been very stable for some time. So that's one. Actually, we should look at that.

I:
So I think that is it for our discussion, Doctor. Maybe if you have anything that you think is important for our discussion, I hope our PhD candidate comes up with a good report.

R:
Okay, yeah. I think we wish him luck. But I think that's a good topic. And his recommendation will be very important. And I want him to present it to the authorities concerned so that they can act. Because as you said, we are not yet there. We need to go there. And in the view of the funding modality infrastructure in the world, we need to be more resilient. We need to be more resilient. Otherwise, we shall perish. So, I think I'll be very happy to look at those recommendations from various respondents and then we put them into action. We want to turn the recommendation into action, to plans, budgets, and move forward so that we're more resilient. But resilience is not an easy thing. No country really is resilient.

I:
 So it's something that you build over time.

R:
Yeah, it's over time.

I:
Thank you so much, Doctor, for your time. You're welcome. And God bless you.
