# Supplementary material for: Resilience as recovery: stakeholder perspectives on Uganda's health system response to infectious disease shocks
Source: Front Health Serv. 2026 Jul 22;6:1888814. doi: 10.3389/frhs.2026.1888814 (PMC13437620; doi:10.3389/frhs.2026.1888814)
Supplement: Supplementary file 2 [file Datasheet2.zip › KII13_.docx]

**TRANSCRIPT OF KEY INFORMANT INTERVIEW**

**I:** Okay, thank you doctor for taking time to participate with us in this study, my name is XXX together with my colleague XXX. Yeah, the reason why we asked you to participate is because we are conducting a research as you had earlier read in the consent from entitled ‘Health Systems Resilience in Africa Harnessing Experiences and Enhancing Capacity Frameworks and Tools, so as part of the study we intend to explore the different perspectives of different stakeholders on their understanding of the concept of the health systems resilience in the local context. So yeah, that is the reason why you are a participant who is going to be participating and so we value the time that you have taken off and we really appreciate the willingness to give us this information

**R:** I lost you a little bit there, can you hear me

**I:** Ooh sorry someone had called me on my phone. So, what we are saying is that Doctor we really appreciate your time and the willingness to contribute, I had earlier one said that I want to stop here and invite my colleague XXX if she has anything else to add on and then we can dive directly into the topic our discussion today

**I_1_:** Nothing to add from me XXX, we can start the interview

**I:** Thank you so much doctor for accepting we had earlier explained that we are trying to understand the different perspectives of the different stakeholders regarding issues of health system resilience

**R:** I didn’t read the consent; do you want to walk me through it [consent]

**I:** Okay, so I can maybe briefly summarize it for you, so the purpose of this study is aimed at generating an understanding of the Ugandan health system that can enable us to recommend targeted interventions and strategies towards the development of resilient health systems against infectious disease outbreaks. In order to avoid unfavorable health outcomes during emergencies, it is imperative that we strengthen our health systems to withstand shocks when they occur. Access to, and ability to provide, quality healthcare should not be interrupted at all times, including during the occurrence of a shock. We also want to explore the status of preparedness of the Ugandan health system to respond to major infectious disease shocks when they occur. Our objectives in this study are to explore the understanding of health system resilience from various stakeholders in Uganda; critically review tools that have been used in assessing health system resilience to infectious disease outbreaks, and adapt a contextualized health system resilience assessment instrument; evaluate the resilience of health systems to infectious disease outbreaks in Mubende and Kassanda Districts, and recommend strategies and interventions for building resilient systems; and identify barriers and enablers to implementing recommended strategies and interventions. We will employ various study designs to achieve these objectives. The insights from these various sources are valuable in helping us answer our research questions. This study has been approved by the Makerere University School of Public Health Research and Ethics Committee. What we will do? If you agree to participate in this study, I would like to ask you to participate in a one hour to one and a half-hour interview to share your perspectives on the resilience of health systems to infectious disease outbreaks in Uganda. The interviewer shall take notes and, with your consent, the process shall be audio-recorded. We shall request you to share your expert views and opinions on these challenges from both a theoretical and a practical perspective, if any. After the interviews, we will make transcripts off the audio recording and review them alongside the scribbled notes, and summarize key findings. We will share with you the transcripts for your concurrence with the content. Your name will not be indicated anywhere during our write-up. During the course of this study, our team shall avail you any new information about the topic that is important for you to know.

Privacy and confidentiality; During the course of the study, there is a risk that some of the information may get out before we are ready to disseminate. However, we will protect your privacy. To do this we will use a unique study identification number and not your full name or personal information. At no time during the study shall we use real names or personal information for identification. Even so, all notes, forms and audio recordings shall be securely kept in lockable cabinets and electronic information stored in password-protected files. On completion of the study, those recordings and forms shall be destroyed.

Benefits: There are no direct benefits to being part of this study. The indirect benefit, however, is that the information we get from these interviews will strengthen efforts towards building health systems resilient to infectious disease outbreaks.

For the time compensation: There is a modest amount which is 50,000 UGX at the end of the interview

Cost: There is no cost to you associated with taking part in this study.

And of course, there is contact information for the PI Denis Okethwangu here in the consent form. So that is it briefly from the consent form maybe if you have any questions before we can proceed.

**R:** Sorry no specific questions from me

**I:** So, doctor just append our signature on that part where there is participant consent you just append the signature and you share back later on

R: Sure

**I:** So, uuh

**R**: Let me find a table here, a convenient place to sit and maybe better internet this one is giving me hard time

**I:** Okay

**R:** Is XXX still on?

**I:** Yes, XXX is still on and he will be the main person asking the questions and I will chip in here and there but he is the main person asking the interviews

**I_1_:** I was just projecting the consent form for doctor to look at it. I think doctor has got where to sit and a convenient place. So, doctor this is the part which I was talking about where you are going to append your signature and then sharing it late on. So, I think without wasting time doctor we can dive in to the topic of our discussion today like we had earlier explained to you doctor that we want to get your views or perspectives regarding health systems resilience in Uganda in the local context. So, maybe before we continue we would like you to share with us your understanding of the term health systems resilience?

**R:** Health systems resilience is your ability to prepare for, manage and adapt to shocks that come into the health system broadly can you prepare for, can you manage and adapt to any shocks that come into the system and ensure that you are able to have continuity of all essential healthcare services same as what we had when we had, when we have COVID Ebola, so all those things can you be able to sustain and continue to implement essential health services in the amidst of those shocks. Or it may a place where floods hit the place and you get a shock of refugees unusual number more than the usual expected numbers of people. So, that is what I understand

**I:** Okay, thank you so much doctor, so in your own view what factors contribute to the ability of the Ugandan health system to withstand and recover from major disruptions in this case outbreaks both at national and sub national level

**R:** I think when you look at our system and health systems in general the capacity to anticipate and prepare for shock is a very important part in determining whether the health systems can be able to withstand the shocks that comes. And preparation here means that can you be able to predict what is to come and put in what is required in that preparation. So yesterday I was reading through, I was reading through a WHO article about Kenya, Ministry of Health Kenya, they have gotten rains and they have started preparing for Cholera in other places. In other places they already have the cholera but they have started preparations in other places and they doing drills and so on in the communities to prepare the health care systems for the shocks that they expect to have. So, the key factors is how prepared is the health systems for these shocks and this preparation is determined by ability to predict and then put in the necessary requirements for those shocks. In the Ugandan health care system, one of the major things is the capacity among the work force to be able to deal with the different types of the shocks without necessarily letting go of all the other services. We have seen a quick example is the recent Ebola when we got a case in Fort Portal City; Virika hospital, all the healthcare workers approximately two thirds (2/3) were exposed. And because of that it wasn’t really two thirds but a significant number got exposed to Ebola and the healthcare services were affected but the ability of the district together with the Ministry of Health (MOH) to mobilize resources; and other healthcare service providers t go and cover up the gap while those ones were in isolation was very critical and that is still part of preparations; having a contingency plan.

A bigger challenge that we normally have as a country is around the commodities; commodities are a big component in being able to prepare for shocks and being able to respond to them appropriately. And when you look at the Ebola outbreak which we had in 2022 compared to this year, 2025. There were key differences that were marked by the availability of counter measurers that helped us to manage and mitigate deaths among Ebola suspects, I mean patients and then the contacts. In 2022, we got a vaccine trial that couldn’t be implemented because the outbreak short-lived but these vaccines prepositioned and kept in NMS and then we had also gotten the what? Remdesivir for compassionate use that was also prepositioned in the country that could not be used in the previous outbreak. That was also prepositioned and these came in handy in managing the cases that we had in 2025 outbreak to minimize death and also the vaccine came in handy all the initial contacts were vaccinated and then we went into the trial mode. We believe prepositioning that is very helpful, right now the county has gotten, we have again prepositioned the monoclonal antibodies which we got from the US government in case there got another outbreak. So, I am really focused on preparation. A big factor that affects resilience is the number of the healthcare workers that are available within the healthcare system that can be able to run multiple services and what commonly affects continuity of health care services is that the availability of the healthcare workforce to deliver all the essential services. So, if you want to I don’t know whether I am still answering whatever you asked me but if you want to have a healthcare system that is resilient you have to locally prepare and own the risk assessments and preparedness plans, once people have preparedness plans they have preparedness plans they will be able to respond better and minimize the impact of the outbreaks or disasters in the health care system

And the other key component that is important is strengthening primary health care services as well as community as well as private and public facilities to be ready to deliver all the required services and having comprehensive services within the primary health care function ensuring that all those facilities have both epidemiologist, surveillance, contact tracing, emergency readiness and response capacities in the primary health care system so that any potential outbreak can be managed at source without it spreading

And the other key thing is ensuring that the health care system can be adaptable. Adaptability is very important, if your health system cannot adapt to certain changes it becomes a problem I will give an example that once you have an outbreak what is the capacity to be able to rapidly disseminate changing guidelines. If you saw how we started with the COVID, no one knew COVID and then and at that time the guidelines kept changing so the capacity to be able to rapidly disseminate the guidelines as they change and the ability to be able to streamline effective care pathways in the healthcare system and providing identification of isolation cases, and then streamline triage and referral systems to minimize impact on the routine services that need to continue in a way implementing differentiated service delivery models. Understanding that if I have a patient who is a suspect within the system how do I manage that patient. If I have one who is normal how do you manage that, so adaptability of the health care system is really very important. Being able to create like we saw during COVID, Ebola and so on how fast can people establish screening spaces within the facility and be able to isolate potential suspects from the others who are not suspects and deliver services for those other suspects. And in the community can you quickly establish home-based care systems, so that you don’t overwhelm the healthcare facilities and we are seeing this with Mpox right now, many cases, Entebbe is full and all the units that are providing care are small or they have small spaces but how adaptable are our health care systems to be able to provide this care. So, we need to prepare, we need to have the ability to manage, stocking counter measures if its needed. You need to ensure that each of the levels have risk and preparedness plans as well as ensure that your system is adaptable. That is why during outbreaks the ministry of health will quickly setup and ensure that we do Zoom calls and invite people on zoom calls and disseminate guidelines quickly, send out people to the field quickly its all to minimize damage. If I have not answered your questions you can rephrase the question

**I:** *Laughs*, thank you so much Dr, in the same line you did mention keys aspects about human resources, and then also medicines and supplies, vaccines and what have you. In the same line talk about the leadership and governance. What are the strengths of this building block and how do they contribute to the ability of the health system to withstand health crisis?

**R:** So, leadership and governance is the centre piece that holds together everything else and leadership governance and coordination. I will give you an example, let me first give an example from the lower level and then we can talk about the higher level. I am right now in Mbale and we are doing supervision in the health facilities evaluating integration of health care services, the shock that hit the health care system when Trump realized the stop work order cut grants from partners and because of that most of the health care facilities were relying on Pepfar funded health care workers to run HIV services. In response to that shock the MOH issued a memo to all districts to integrate health care services. A key component of leadership is one being able to be decisive and offer guidance in the middle of chaos. The MOH came out and said, we may not have a clear plan now but we must be able to issue a decision. When the permanent Secretary came out and said that they have cut funds but we are going to manage the services creating confidence in the community that there is ownership and there is ability to take over the program. Leadership creates that confidence and reduces panic when there is a problem.

And the strategy to instruct all the district s to integrate services was key. You issue the guidance and you let the teams deal with implementing it. I have gone to a couple of facilities some that have clearly implemented this guidance and they have started implementing integration and others that have done nothing. The difference the biggest difference that I have seen is the leadership within the facility and ownership. You find a facility that has integrated and you talk to the manager, he clearly owned the guidance from the PS, brought the team together and told them that it is possible and they started it may not be ay 100% but they are very enthusiastic, leadership has a key role in providing guidance and being able to direct action when it is needed.

The leadership and governance at national level, we may have outbreaks, we have the IMS; the incident management system within structures but at strategic level, we have the other strategic levels directives that come from the top management. Before an outbreak and so on like right now Ebola has ended the guidance that ought to come from the leadership about strategic issues like how do we plan response to any other outbreak. There may be people responding to outbreaks but if you look at Mpox right now, the key strategic decisions that are coming is that we have sufficient vaccines in the country. There are partners who are responding, running around looking for people who are sick and providing them with care which is a component but the big decision is that can we make sure we have sufficient vaccines to close off the tap. So, leadership is very important in providing such guidance when you have IMT decisions that are made in the IMT at strategic level are the decisions that guide response and these are very critical decisions/ And the decisions made at strategic level for example when in 2022 when Ebola entered into Kampala the decision to quarantine all contacts was very key to mitigating deaths and spread of Ebola within Kampala city which should have been very nasty if that decision was not made and it is a similar decision that helped us to stop this outbreak quickly. So, leadership and governance are very important, the components of governance, ensuring that there is money available for the response. Mobilization of funds for the response, monitoring what is being implemented in the entire health care system and ensuring that policies and guidelines are adhered to at national level and of course also managing up. There is managing things down but also managing up. And by managing up meaning that the executive in the country is informed, parliament is informed and minimizing the impact of the outbreak on the other sectors.

One of the key challenges we have had in the outbreaks is the impact on the economy, impact on schools. The leadership and governance provide that role to ensure that there is balanced management of the outbreak and, to save lives and minimize damage to livelihoods. Ensure there are policies, ensure there are guidelines, ensure that people have the materials that are required and money. XXX over to you

**I:** Okay thank you so much doctor briefly can you talk about the health information management system. The role of the HMIS to build a resilient health system.

**R:** Yes, if I may summarize the HMIS, for you to…you need data to understand what is happening to you and understand what to do. One of the key indicators that has been set out to understand how well a system is in responding to outbreaks is 717 matrix. The 717 matrix is so much dependent on systems, the first 7 is the ability to detect an outbreak within 7 days and that is dependent on the ability of communities and facilities to identify alerts and be able to notify through the HMIS and trigger a team to investigate and maybe declare an outbreak and maybe respond on time. So, in terms of preparedness and identification; early identification there is a lot that lies in the HMIS in identifying as I have mentioned but also within the facilities the surveillance systems and the reporting that is required and the ability to be able to track trends of notifiable diseases all that us reliant on the HMIS, the capacity within the facility to be able to report and the ability of the district and the region or the MOH to be able to look at that data and understand it. There is a component of the availability of the infrastructure for HMIS, the infrastructure to be able to capture and report outbreak data urgently and quickly that is very key. And in being able to ensure that you detect and respond to an outbreak quickly and minimize its damage in the health care system and of course promote resilience within those facilities. So, there is more and more urge to ensure that the HMIS are digitized including for lab to be able to easily track samples, to be able to report in time, to be able to provide data that can be analyzed quickly and inform the response to an outbreak and changes its course when managing outbreaks. And uh as you may know information is power, without information you will not be able to achieve as fast as u can. If you look at all the decisions that are made that leadership makes involving outbreaks is based on information and this information is data that is coming through the HMIS. A few gaps there is still a need to digitize our information systems and ensure that there is stability of internet and power within the different facilities to be able to deliver whatever we want and of course this will eventually reduce the need for paper and reduce the burden on human resource to do paperwork and then later on transfer to the electronic for reporting. If we get to that level and there is a systematic approach to be able to analyze and understand what is happening at that level you can be able to minimize damage or harm that is caused by an outbreak within a system. The ministry is trying to put in initiatives like digitizing some of the reports and setting analytics in there so that you can be able to quickly identify any emerging trends for example the HMIS 33b that monitors ah outbreak prone diseases. They are digitizing the stems to ensure that you can quickly and easily identify any trends and be able to respond in time to control outbreaks and minimize the effects, I mean the damage that is caused. Minimize loss of life and minimize the loss to the economy. So, the health information management is very critical, all our outbreaks run based on data.

**I:** Doctor, can you hear me?

**R:** Yes, I can hear you

**I:** Okay you did hint on earlier about the community in your previous submission, I want you to expound more how you view community engagement as a factor in this resilience

**R:** So, outbreaks begin in communities, and they spread via the social and economic links between people and outbreaks end in the community through successful implementation of public health and social measures by and with the affected communities. I don’t know of any outbreak that has been successfully controlled without the community. And a number of time the trust or lack of trust and transparency creates resistance to public measures within the communities. People often trust the people they know, people that they listen to and who act on their concerns. They trust what they understand and they trust measures that they feel ownership for and are consulted on. I will give you two examples, number one is the COVID vaccination drive, the COVID vaccination drive initially got the first lot of vaccines and when they were taken upcountry there was a lot of resistance. We went to Kasese to do a dialogue meeting and what we got from the leaders was that the ministry of health got their vaccines, they did their things and they brought here to vaccinate us so we left them to do their own thing. But when we talked to them including answering their questions including giving experiences of how we ourselves had been vaccinated and how we felt after vaccination immediately during the dialogue meetings people asked whether we had come with the vaccines so that they could receive as well. So, community dialogues ensure that there is community ownership and it must be done from the point of preparedness up to the point of response, so it promotes ownership and there must be active listening and we must provide access to services to the community and ensure that we have stronger community health systems. When we had Ebola in 2022, I asked the RDC of Kassanda that if you were to do something different, if we have another Ebola outbreak what would you do differently. And she told I would have not wasted time with you; medical people I would have gone to mobilize the community and talk to them and ensure that they understand that this is a danger we would have controlled the outbreak faster.

**I:** Okay, yes doctor

**R:** I am done

**I:** Okay thank you so much, let us talk about the interventions that are contributing to resilience of our health system. What is specific programs, or strategies or policies have had a noticeable impact in strengthening the systems’ resilience in Ugandan context?

**R:** I think one of the key factors that has contributed to resilience is the capacity building that has we had less mortality part of that is that we have built capacity across the healthcare work force to be able to respond to outbreaks as such creating a lot more resilience. If COVID came now it wouldn’t affect us as it did before.

The other that has promoted resilience is actually the availability of life saving safe and scalable health interventions; these have been very important in promoting resilience being able to have the intervention that are required. More importantly even our laboratory systems you saw how we moved from DNA PCR diagnostic for COVID to rapid diagnostic tests and a robust sample transportation network that could quickly pick samples from one place to another and transport them for testing. Having that infrastructure in place and that system has promoted a lot of resilience with the lab testing system and we are even being to see ourselves have mobile labs. When we had Ebola, the mobile lab was deployed in Mbale and when we had Mpox the mobile lab was deployed in Bwera. So being able to have that laboratory capacity to be able to test quickly that has really contributed to promoting resilience.

But also, there has been a lot of improved coordination and management of outbreaks. We have had incident management teams that have been active since we got COVID and they have hardly gone off and they have gained a lot of experience in coordinating outbreak response within the country.

And of course, not to forget one of the key factors the funding. We have also had a lot of funding support from government as well as from bilateral donors within the country who have contributed in a timely manner to the management of outbreaks as well building systems. Right now, we are doing recovery plans and being able to prepare better for the next outbreak. And of course, for us in Uganda the presence of implementing partners who have been working closely at district and community level has also played a very critical role in being able to quickly respond and control outbreaks.

And I think as a country we have also had a very robust approach to health promotion. If it is not politically affecting the economy the health promotion kit is a very key component in drawing communities and populations. And we have a very robust community health care system that gets easily mobilized. When we had Ebola in Mubende, the VHTs contributed heavily to the control of that outbreak. I remember we trained close to a thousand VHTs in one week and set up a system to quickly follow up all contacts. Once the VHTs were trained we started getting alerts and identifying new cases quickly and rapidly. I think our robust community healthcare system once facilitated well also contributes to the resilience of the health care system. During COVID we had to manage people at home; home based care this is part of the system that was built to manage people. Not 100% in terms of delivering what is supposed to but it alleviates a lot of burden from the main health care system.

**I:** Wow, wow, thank you so much doctor, now we would like to look at the characteristics of a resilient health system. You maybe just paint for us a picture of what a resilient health system looks like particularly in response to infectious outbreaks

**R:** Yeah so for me a resilient health care system must have core public health functions built in there. So, you must be able to have surveillance; passive and active surveillance, you must be able to have the capacity for epidemiology meaning that you are able to utilize the data that is coming from surveillance to be able to quickly identify threats, the health care system that is resilient must have health emergency readiness and response capacity at primary health care levels as well as contact tracing capacity. And the system should be able to have locally prepared and owned risk assessments and preparedness plans. There is what we call in districts disaster preparedness plans where districts do star risk assessments, strategic risk assessments and they develop plans to be able to respond to any outbreaks but those must be locally prepared and owned by the facility and they should be tested routinely to ensure that the plans can be implemented. Tested either through simulation, different types of simulation exercises. For us to say that this healthcare system is resilient there must be preparation as well as in the community level. So, the community healthcare systems must be functional and there should be surveillance systems within the community to quickly identify but also to be able to support response to outbreaks. And when we say the system this should cut across between private and public facilities within the health care systems and there should be capacity to be adaptable but still be able to deliver services. We have seen in a couple of places during COVID and so on where facilities had to create isolation units and create wards within to be able to mange these cases, and I think one of the key things for us as a country that will help with ensuring resilience and what is to be able to develop, is to have a fund for outbreak response. So, there must be also availability of money and availability of supplies and there should be a system that can be able to deliver all those things to the right place at the right time and of course the capacity to diagnose any infectious outbreak in a timely manner and we train people.

**I:** Okay, thank you so much doctor, in that same view lets talk about the unique attributes of the Ugandan health system, the uniqueness. In your opinion doctor what are some of the unique attributes about the Ugandan health system that works towards making the system more resilient

**R:** Uum, there are a few key things to look at, if you look at the healthcare system and the structure of the health care system in the country, it has been designed in such a way that we have service delivery at all levels starting from the community, health Centre II, III, IVs and hospitals and the referral hospitals as well as the private sector. This has potential to provide alternatives for service delivery across and minimizing impact on delivery of health care services in case you have a shock. We had a time when the health facility was submerged in Ntoroko but the system was able to quickly provide access to health care services in the neighboring facility. So, there is a structure that is robust and I believe if operationalized fully provides an opportunity to promote resilience

Number two (2) is the leadership and governance at different levels, whether this leadership and governance is effective at all levels is debatable but there is a clearly instituted structure to promote resilience right from the ministry level the different departments, divisions and so on that have been created to be able to ensure that there is continuity of these services and these structures are reflected downwards at regional level as well as at district level to be able to ensure that there is monitoring of the delivery of the health care services and ensuring and instituting countermeasures when needed. In addition to of course identifying outbreaks on time. So, there is capacity for surveillance, we trained people on eIDSR there is both capacity that has been instituted in the community and the facility that provide a recipe for better resilience of the facilities although it might be sub-optimally used but it is available. You know that in the community we have the 6767 and then in the facilities we have all the channels reporting including eIDSR that can be used.

We have a robust surveillance structure that is probably better any of the many of the places. I think this has been key for us as a country and that is why you find that probably in a quarter you have 25 or so outbreaks that the country has activated for because of the capacity to identify.

The other big thing is our laboratory structure, CPHL, UVRI and all the regional labs and the network for sample transportation and results delivery. I think that is a unique attribute to Uganda that doesn’t exist in many countries that makes us more resilience to these outbreaks

The other key thing that has been formed that has been very helpful is the presence of the EOCs, as part of the systems to promote regional and national surveillance and coordination of response work. And then when you talk about the response it self we have relatively knowledgeable healthcare workforce on outbreak response across the country that whenever there is an outbreak there are rapid response teams that can be mobilized quickly and that can be deployed. I think that is a plus for the country and well established and easy to mobilize IMS, IMS systems that can be mobilized quickly. As part of the preparedness every time we have big outbreaks the EOCs is activated in less than 24 hours and teams are mobilized and in one day there are teams in the field to start response and I think that makes us unique as a country.

And the other one that is very key and important in our country is the political leadership we saw during COVID the president leading during the COVID outbreak, in giving guidance. Communities would listen better to what the president says but of course they started grumbling when it started affecting their pockets. So, those some of the things that come off head.

What we need to do a little better is the financing for these systems, local financing for these systems during outbreaks because most of our financing has been dependent on donor funds. I think that is the downside and with now most donors turning off the taps we are likely going to face an uphill task in trying to make sure that we control outbreaks on time and have a resilient health system.

**I:** Uum, wow, wow and thank you so much doctor. I would want to take you back a little bit in the role of the community in the health system resilience. Earlier on you did point it out but could you further explain how can community structures such as the local leaders, or health volunteers further support the health system resilience apart from training that you had earlier on mentioned

**R:** So, we talked about a few things, our communities are organized from villages, you go to parishes and there are sub counties, you have counties and districts. Within those communities we have health structures, we have VHTs that support households and we have health assistants that work within the communities. The VHTs currently are supporting with surveillance, early identification of suspects and communicating to the health care system. That is on way that they can support. During outbreaks, we have what we call the parish task forces, the sub county task forces, district task forces. Within the task forces at those levels they have supported coordination of the outbreaks within the communities and mobilizing the communities for the outbreaks. The other arm of the community that we can’t ignore are the influencers within the community and the local leaders; the LCs, the religious leaders, the cultural leaders very critical in health promotion, creating confidence in the community about countermeasures that are coming up. Those are the key gatekeepers in the community if you are to succeed with making the health care system resilient as well as the political leaders, once these people buy in then you will get action within the community level where the outbreaks are happening and you will be able to have resilience within the health care system. We have had examples during COVID outbreaks we identified hotspots and mobilized community leaders to go and enforce the control measures within their community

And reduce the spread of the outbreak, we saw during Ebola when we wanted to do mortality surveillance where we worked with agencies within the parishes to be able to report all deaths and enable us to investigate all deaths within the community and control the damage. So, the community is very critical but also, I think what we underestimate is the role of the households and individuals within the community to take these measures. If people accept and implement the measures within the community the diseases will not spread within that community and that helps us a lot in controlling the outbreak, any outbreak and making the system more resilient. If they say put on masks and people put on masks, so their ability to be able to cooperate with the health care system is very important.

Right now, there is a move to be able to train community health extension workers who can be able to manage conditions at home. Those if they become effective can be the group that can support things like home-based care. For a couple of conditions within the facility if I am having outbreaks and it’s risky to bring people to the health care facilities, I can deliver ARVs through the community structures that is one of the things which helped us during COVID to ensure that ARVs were delivered to different people when they stopped movement, community structures were used to deliver ARVs to people and ensure continuity of health care services for people on chronic care medication. So, the community is extremely important in such circumstances. [*You have finished your time do you still have more questions?*]

**I:** Yes, we just want to look at the few questions those are challenges and recommendations and without wasting time, lets talk about the challenges to health systems resilience. Earlier on you had mentioned about financing, what are some of the other obstacles that affect Uganda’s health systems’ ability to withstand and recover from infectious diseases.

**R:** Finance is one of the biggest you need the ability to finance the health structure within the country looking at both human resource and commodities for delivery health services. A number of our health facilities are having less than the required capacity to deliver the services required for them. It means in such instances when you have an outbreak it is atomically going to affect the delivery of all the other essential health services if these health workers are directed to managing outbreaks. So, financing is the center piece

The other key component that is very important to look at it is leadership I gave and example of the ones were visiting here [Mbale] the ones that were making progress were the ones that have leadership and those that didn’t had significant gaps in leadership. We need to be able to strengthen leadership from the community level upwards to the level of the ministry of health and the leadership to some of the structures that we have contributes to less reliance within the facilities. This morning we were meeting with the DHO and one of the things that he mentioned is organized absenteeism from work. If you have organized absenteeism from work you cannot have a health system that is resilient when you are hit disaster. So, that leadership and governance at sub national level still needs a lot to be strengthened.

We need to reduce a lot of course on donor dependency to having country ownership. One of the other challenges to the resilience of the health care system is the available infrastructure for delivery of all that is required. In a couple of countries when you have an outbreak they have specific places where the outbreaks are managed. We had a COVID outbreak I remember in Fort Portal Regional hospital, they displaced people from the male ward and turned the male ward into a treatment center and what does that mean? There was an effect on essential health services for the men within that region because then they were pushed to a smaller place. So, we need a lot of infrastructure as a gap.

The other biggest challenge that we have that we should be able to address is the ability to get counter measures. You saw what happened when we were getting the first dose of AstraZeneca in Uganda some people in the US were having the second or the third dose. So, our capacity to be able to have counter measures on time. And counter measures that are scalable. A case in point s not we know for sure that the US and others were able to control Mpox through vaccination but right now we are just vaccinating small populations because we don’t have what? We don’t sufficient vaccines for Mpox. The same thing happened with COVID, having sufficient counter measures on time. We need to embrace the bigger move by the Africa CDC to strengthen local production of countermeasures and local her may mean within the African Continent and being able to work collaboratively to ensure that we have a force that can advocate for and get these measures at the time when we need them.

The other portion that is not so well developed in our system is the research especially research about outbreaks where we don’t have capacity to do independent research but also at the same time research is not in tandem and doesn’t not happen at the right time when outbreaks are happening. We need to be able to strengthen capacity for research, prioritize research that will inform how we can be able to have systems that strengthen the resilience of our health care system. So, we need that research and research on the counter measures and how they best perform within the community in the country. Key gaps of course key things that need to be done is bringing on board for example regulatory authorities to ensure that the regulatory authorities understand the urgency of initiating research quickly and being able to review these documents and providing approval on time so that we can be able to do this research on time to be able to understand the counter measures. As a country of course we need a blue print for research in health systems strengthening.

We have local manufacturing here we need to see how to as a country, how to be able to leverage that to begin to produce some local counter measures that are required. We know that there are robust global and regional mechanisms for distribution of emergency products however, they may not be equitable and there is a greater need for us to begin to discuss around transfer of technology to places where this can be accessed easily this is the move that Africa CDC is currently negotiating across. Can we have local manufacturing of vaccines and dealing with the people who have the intellectual property rights for these, to transfer that technology to within, Africa.

Global political and the geopolitical environment that you see right now of course is a major challenge and this challenge comes up right now because most of our funding for health systems work is coming from; not really most but a significant component is coming from donors. This a challenge that we are going to face the current geopolitical situation with different countries cutting down funds and the norm has become reduced funding for health care. The solution for this is for the government to own more of these services and allocate more funding. Uganda is not very close to the Abuja declaration agreement on the percentage of funding that should be for health as a percentage total budget for the country. So, there is need for more advocacy, advocacy for more funding and to be able to have advocacy groups that target increasing funding to the health sector. And advocacy groups who know where to go and not going to the minister of health but going to finance and going to parliament to advocate for funding for health in the country and being bale to make pitches that make sense to the non-medical professionals. Because when you get doctors to pitch for more many for health they will only think about health but you need to learn to advocate in such a way that you learn to talk to the people and tell them how this will affect the economy, how it will affect other sectors and why it is important. So, that is the advocacy for more funding is very critical for us as a country.

The leadership component there is just a need to strengthen governance at district level and we have tried this previously at a lower level at the facilities. We had a training that we developed as Baylor for leadership at health facility level, where we were training these people on their roles and responsibilities and providing them with tools where they can have effective like for example district or health facility meetings, tracking their action points, tracking the attendance within the facility and monitoring their performance within those facilities. So, some of those measures at that lower level can help us to improve issues of governance and leadership within the country.

Of course, part of what affects, I don’t know whether to call it resilience but that has continuously affected the ability to communicate outbreaks openly across different countries is the way that the international community implemented the IHR. You remember during COVID when they discovered a strain in South Africa and they were put in a travel ban in South Africa. Such measures have caused the countries to become less open in communicating outbreaks because then they put you on a no-fly list and then you don’t get tourists coming to your country and it affects the economy in one way or the other. And when you are not open in communicating outbreaks then you are also likely to have more impact in your health care system, if you are not having support to be able to manage the outbreak quickly and control it.

The key challenge especially for the HMIS is the infrastructure; infrastructure is not to the lowest level and of course the internet bandwidth and its spread within the whole country still remains a bottleneck. But there have been movements in that area with government working with key providers to take internet close to the district but someone still has to pay that cost and of course the strength of the internet is not that fast to enable people to work very quickly. So, we need to work to promote this IT infrastructure within our facilities to ensure that reporting is seamless and promotes identification of outbreaks and making us respond on time to control outbreaks quickly.

The other big component we have a community structure but they are not remunerated and we need to be a little bit innovative about this. VHTS are there but they are not paid. I have seen places, I was in a conference and I was chatting with a colleague from Rwanda on how Rwanda is motivating their community health workers and they said you know the community health workers are not paid but government has supported them to form SACCOs and in those SACCOS they have given them money to start investments and then they have formed them into groups and they have little companies and such that when there is need to prequalify a vendor for example to clean up health facilities, those tenders are given to the community health care workers. And that is how they have been able to sustain the structure of the community health care workers without paying a lot of money for it. Some of those innovations are required for our community health care work force.

My battery is warning, it is going to run in less than 5 minutes

**I:** Okay thank you doctor, you have really elaborated and since we have the battery problem there, maybe are there any successful models from other countries or contexts that you think can be adapted to the Ugandan Context to enhance health system resilience

R: So that is homework for Denis, tell him part of the PhD is to read up those models, anywhere I am tired

I: And then maybe lastly doctor, uh apart from the strategies that you have mentioned are there any other initiatives that you could propose to strengthen the health care system and improve resilience?

R: Getting money for response on time, financing, you have seen the pandemic being started globally I think Uganda needs to put its own pandemic fund t be able t implement such things. Countries have funded such initiatives in different ways; people like Zimbabwe at one point when they had cut off money for ARVs put a tax on cigarettes and they were able to get money to fund their HIV program. So, we need to think about innovative ways of financing. We need to strengthen private public partnerships (PPPs), most of the outbreaks we have had in the country have been identified in the private sector including Ebola and other outbreaks. So, strengthen the PPPs and the contribution of the private sector into the health care system response is very critical. Most of our patients access care in the private sector before they go to the public facility and I think that is very critical in creating resilience if we are going to have resilience we are going to have resilience both in private and public facilities alike. So, PPP is very important

Other in terms of capacity building is we need to move away from the component of responding by running and starting to train people when there is an outbreak and so on. We need to mainstream our capacity building into our pre-service trainings in the country. Such that at the point when someone is coming out of the university or out of the nursing training school, or school of clinical officers the person should be able to respond to an outbreak in one way or the other. For example, infection prevention and control (IPC), when the MOH of passed IPC guidelines can we be able to integrate those into the trainings such that if you produce nurses they already understand what it means to implement IPC. We need to have different initiatives and also capacity building including virtual communities of practice and creating training such that people can attend without gathering in places. So, that we make our capacity building approaches cheaper and continuous where people can have where to find the information that we need and we reduce on the dependence on donor funding.

And the other key thing is strengthening regional structures for coordination of response and preparedness work in the country, we will need to strengthen the coordination at the lower level and regional structures; regional EOCs and regional referral hospitals of the place to promote resilience but also to ensure that response is managed well. Alright I have to go now

I: Yes, thank so much doctor for your time, think we have also come to the end of this interview. We want to thank you so much I don’t know if my colleagues XXX, XXX do you have one word or two for doctor before he drops off.

I_1_: Nothing from me

R: She has dosed off

I: I think XXX is off, so I think doctor that is it and I think we will be waiting for that signed consent from you and then also Denis will reach out to you

R: Thank you

I_1_: I had not dozed off I was struggling to unmute but thank very much doctor, you were very elaborative, so I don’t have any questions for you. So, thank you for your time

R: Have a good evening

I_1_: Good evening to you too

I: Have a good evening doctor
