# Supplementary material for: Resilience as recovery: stakeholder perspectives on Uganda's health system response to infectious disease shocks
Source: Front Health Serv. 2026 Jul 22;6:1888814. doi: 10.3389/frhs.2026.1888814 (PMC13437620; doi:10.3389/frhs.2026.1888814)
Supplement: Supplementary file 2 [file Datasheet2.zip › KII14.docx]

**TRANSCRIPT OF KEY INFORMANT INTERVIEW**

**Interviewer: So basically, we are exploring the understanding of the concept of health systems resilience and determinants that influence the health system resilience to infectious disease epidemics in Uganda. So, we could start by you briefly describing a health system resilience.**

**Respondent:** Okay, so my understanding of health systems resilience is the ability of the system to rebound back to normal operations after any outbreak-related disruptions in service delivery. So, for example, we recently had COVID and a lot of the operations of the health system changed. And there were a lot of disruption in the delivery of other services, non-COVID services. For example, maternal child health services, TB and HIV programming. So, the ability of the health care system to rebound from that kind of effect, pandemic-related effect, to normal delivery of all essential health services is what I would consider a health system resilience.

**Interviewer: Okay, you could also share key characteristics that make up a resilient health system.**

**Respondent:** So, I would take a resilient system as a system which is able to respond to the needs, the health needs of a given population. So, the system should be well-financed. It should address all the major health challenges in a given country. For example, here in Uganda, the biggest challenges remain HIV, TB, malaria, hypertension, heart diseases, and other maternal child health. So, the system should be able to address that. The system should have all the technology that is required. So, that feeds into, for example, the health information systems, the diagnostics and laboratory equipment that are needed. Then the other one is the system should have stable governance. So, even when there is an outbreak, the governance system should remain stable to provide leadership for other essential health services. So, that is what I would take to be the characteristics of a resilient system.

**Interviewer: Okay, and if we are to paint a picture of a resilient health system here in Uganda, based on your characteristics, to you, looking at Uganda, what are the characteristics that make a resilient health system in the Ugandan context and also share some examples?**

**Respondent:** I thought these are some of the things that I mentioned.

**Interviewer: Yes, these were general, but which ones could you relate to Uganda’s context?**

**Respondent:** In Uganda's context. So, in Uganda, the characteristics that I would consider key is the ability of the system to still provide essential medical services for our major health issues. Our major health issues in Uganda are childhood diseases, HIV, TB, malaria, maternal child health, and NCD services. So, the system should be able to provide those services, but the other key, most important thing is funding, financing. So, even during outbreaks, for example, the funding towards those critical essential services must not be interrupted because of an outbreak.

**Interviewer: Okay, and when we look at the factors, in your view, what factors contribute to the ability of the Ugandan health system to withstand and recover from the major disruptions, we are looking at national and sub-national levels.**

**Respondent:** So, it's political leadership and governance, first of all. So, you know, issues like healthcare financing and policy and decision making are all political decisions. So, there has to be strong governance and political will at national level to make sure that we design policies that ensure resilient health systems. And at sub-national level, for example, when you go to district level, there has to be strong political will of the LC5, but there also has to be the district health committees, for example, to make sure that they oversight all those services. And indirectly, almost all of those now lead to the other issues that I talked about, the financing, the health management information systems, the laboratory systems and equipment, all of those literally depend on governance and leadership. So, for me, that is the most important issue. And then the other one which ensures governance is community engagement. So, the extent to which the health system integrates and engages the community will greatly help to make the health system resilient. For example, you know, we have our community structures, for example, the VHTs. If the health system does not strongly integrate the contribution of those community structures, it literally means it cannot offer healthcare services for the people.

**Interviewer: So, looking back at the political leadership and governance that you talked about, what are some of the weaknesses that you think may contribute to a weaker system?**

**Respondent:** So, first of all, the challenge that I see is that decision-making tends to be made to score political interests. So, for example, we definitely know that we need a national health insurance system, but the government will not enact that law because the President or the parliament knows that the majority of the people will complain against healthcare financing, and therefore, they'd rather not do it than do it, even when it would be the best decision to make. Okay? And then the other thing is that in Uganda, of late, all most important policy decisions have been so centralized, so the President almost has the last say on everything for instance you remember what happened during COVID, when the President got involved to communicate to the public about what the current state was and the issues of lock down.

And yet, when we talk about healthcare delivery, we are talking about we want to be as close to the community as possible. So, the community leaders should have a bigger say on these policies, rather than it should be a bottom-up approach in terms of decision-making, but in Uganda, it tends to be up-bottom.

**Interviewer: Okay, thank you for sharing all these views, and still on factors, any factors in relation to human resource for health?**

**Respondent:** Yeah, there should be considerations for human resource that fits the peculiar needs of a given society for example, most of the conditions that Uganda deals with are really primary healthcare issues. The diarrhea, the pneumonia, malaria, you're getting it? So, the human resource and staffing should ideally reflect the needs of the community. Are we together? So, a lot of, for example, the human resource staffing in Uganda tends to be in the clinical setting, okay? So, we have so many nurses, we have so many doctors, and so on, but we almost have no community healthcare workers. The community healthcare workers, the VHTs are there, but they are not remunerated, their terms of reference are not very clear, and that curtails the whole approach to human resource for a resilient system. So, we ideally need to make a policy shift and move away from the current looking at human resource in clinical setting, but also incorporate human resource for community health.

**Interviewer: Okay. Yeah. So, you have talked about community engagement and the community health workers. Elaborate more about this and the roles of the community towards resilient health systems.**

**Respondent:** The community, first of all, should be involved in all decision making, okay? For example, they should be the ones to map what are their priority health needs, for example. And they should also be engaged in the delivery of services, okay? For example, they can mobilize themselves to dig up pit latrines, to ensure hygiene and cleanliness at community level. But also, they could as well support the health system in reporting disease outbreaks, which is very critical for outbreaks. So, the VHTs and other resourceful people could pick these disease signals and report them to the mainstream surveillance systems. So, when you look at it that way, you literally realize that you can't have a resilient health system that does not have strong community engagement mechanisms.

**Interviewer: Okay. Let's talk about the interventions or actions that have been taken that have contributed to the resilience of the Uganda health system.**

**Respondent:** So, we make decisions quite fast, okay? So, for example, when it comes to declaring an outbreak, the Minister is normally very swift to declare the outbreak. And Uganda also has a strong partnership and engagement with other players, other stakeholders, For example, the donor community, I think we do very well with engaging the donor community to support these outbreaks, okay? So, they tend to supplement with the finances. But also, we tend to do a very good job when it comes to providing clinical care for those who are affected, because by doing so, we build confidence in the population to come out and report themselves when they have signs and symptoms. So, the population now has strong confidence in the health system. And therefore, they will come up and report if they have any signals of disease outbreaks, rather than just sitting on them, like what happens in other countries.

**Interviewer: Okay. Any other interventions or policies that have had a noticeable impact?**

**Respondent:** No, the policies are there. We have, for example, the Integrated Disease Surveillance System, IDSR, which helps to pick outbreaks from the peripheral health facilities and ensure that they are reported very fast. But one thing that I realize which helps Uganda in the outbreaks, we have strongly implemented the 717 framework. And you know the 717 is the framework for measuring the timeliness of outbreak response. So, an outbreak must be detected within seven days, must be notified to the national level within one day, and the government or the agents should take seven days to amount an immediate response. Now, the other thing is that, as part of this framework, there are normally intra-action reviews. So, when there is an outbreak, there is a mid-action review, there is an end-action review, where we identify the barriers to addressing a given challenge and devise strategies for addressing those barriers. So, the use of such frameworks have also been very helpful. These are frameworks that are designed by multilateral agencies like Resolve to Save Lives, WHO, and other entities. But they help countries, they give a clear vision to countries on how to measure their response to outbreaks.

**Interviewer: Okay, I know you briefly shared some of the challenges, but any other challenges that affect the health system's ability to withstand and recover from the infectious disease?**

**Respondent:** The biggest problem is we don't have funding for outbreaks. There is no dedicated budget for these outbreaks. So, the government normally plans for the usual health services. And whenever there is an outbreak, resources are normally channeled from other critical health sector towards outbreak response and that ends up disrupting the services, which shouldn't have been disrupted. So, we need to have an emergency fund. An emergency fund for outbreaks, so that when there is an outbreak, that fund is utilized. And the government can levy taxes to gather that money for example, tax the beers and whatever, all those kinds of drinks. The other issue is that because of limited funding, we normally tend to focus on the health system recovery. But you know these diseases affect the livelihood. They affect the livelihood of people, they affect the social setup of communities, for example, COVID. Now, we normally tend to look at recovery in terms of putting out of action the outbreak. But these communities sometimes need to recover. And there is no funding for those recovery mechanisms outside health, you know. That is, I think, the major, major issues I can speak to.

**Interviewer: Okay. And how can stakeholders work together to overcome these challenges?**

**Respondent:** So, the stakeholders, first of all, have to be organized. And that is a role of the government. The government needs to do a thorough stakeholder mapping and make sure they know who are the stakeholders, what do they do. And sometimes they need to revise their terms of reference and engagement so that their actions are coordinated.

You know, during these outbreaks, there is sometimes a lack of coordination. But the role of coordination is supposed to be government. So, once that is done, you literally make sure that every partner or every stakeholder does what they are best at. Okay. So, for example, if we say that WHO, for you, are very good at drafting guidelines, focus on the guidelines. If we say that USAID and CDC, for you, are very good at funding, bring in the money. And then if we know that there is a group that is very good at risk communication and education, they focus on that. Then if the academia is good at research, they also do research. You get it? But that can only happen if there is very nice coordination, which in Uganda has tended to be very good, because there are normally partner meetings to make sure that they are coordinated in response. And these meetings normally happen at national and subnational levels.

**Interviewer: Any strategies or initiatives that you would propose to strengthen the health system and improve resilience? You talked about need for emergency fund…**

Respondent: Yeah, but I also talked about health care financing, insurance system, which has never taken place. But it would be nice to have an insurance system in place.

**Interviewer: Elaborate more about the insurance system.**

Respondent: So, right now the government shoulders all the costs of health care delivery directly. But we know that indirectly also people share the costs. Now, it is increasingly not sustainable for government to do that because the population is growing. But also a significant proportion of Ugandans now get care from private health services. Now, what happens is that whenever there is an outbreak, people are not working. And therefore their finances are limited. Okay, So, if you don't have an insurance system, people cannot access the health services. So, an insurance system would ensure that people could still access the critical health services, even when the government does not have sufficient funding for those critical services. Okay? So, the insurance mechanism would supplement the available government funding to help the citizenry to access the services which are critically needed.

**Interviewer: Any other initiatives you would propose?**

Respondent: The other initiative I already talked about, it is a multi-sectoral approach. So, we have to start looking at resilience beyond the health sector alone, But also there has to be economic resilience. And therefore, during outbreak preparedness and response, we need to bring other partners on the board. For example, the education minister to make sure children are not dropping out of school, Ministries of Finance to make sure that people's livelihood are not being affected, trade to make sure that business interruptions are minimized. You sometimes also need the transport sector to see how you can safely ensure transport systems are maintained, even during an outbreak. And then you literally also have to, and this is a different point, it is making sure that you ensure quality health services. So, you literally have to make sure that everything that you do measures up to the quality or the standards of care. Because if you don't do that, people will lose trust in the health services. Yeah, I'm trying to see if there is any other issue I've not spoken to.

**Interviewer: Okay, then in relation to community, let's look at the VHTs that you earlier talked about. How can VHTs be better supported or empowered to develop a more resilient health system?**

I think I spoke to that actually. I said, number one, they need to have clear terms of reference. You know, VHTs should have like a formal, even a formal appointment but they should be remunerated. VHTs need, they need supervisory systems. Right now, VHTs are supervised by health center II In-charges. But we know that is not working. There are also health assistants who are supposed to work with them. But that whole thing is not working very well. Now, when it comes to outbreaks, it's a disaster because the community healthcare workers are supposed to be working very closely with their colleagues in the animal sector in terms of the One Health approach because these diseases begin from animals, before they attack the people. But I don't think we have community animal health workers in Uganda. Maybe they are there, but I'm not aware of them. And if I'm not aware of them, in my role, it literally means they are non-functional, even if they are there. So, there's a need for coordination. We have done very well with the One Health approach. But that is currently missing at community level. Yeah.

**Interviewer: Are there any successful models that you think would suit Uganda's context better?**

**Respondent:** You need to break down that question. Which models? What do you mean models?

**Interviewer: Okay, how I understand it. Any interventions that have worked in some districts or other countries that have contributed to the resilience?**

**Respondent:** You asked me some of them. Like a multi-sectoral approach, that one I mentioned. Having strong disease surveillance systems, I talked about IDSR. All those.

**Interviewer: But have they been successful elsewhere?**

Respondent: Yeah, even in Uganda they have been successful.

Interviewer: Okay, so as we come to the end, Doctor, this question of the factors. I would like us to discuss more about it. Factors that contribute to the ability of the Ugandan health system to withstand. We talked about leadership and governance…. Elaborate more.

**Respondent:** And I talked about financing. I talked about human resources. I talked about information health systems. I talked about the laboratory systems,

**Interviewer: Anything to talk about medical products, vaccines, technology…**

Respondent: Yeah, those ones should be there. Absolutely, absolutely. So, the vaccines and medical counter measures stroke innovations. There are also other innovations which are not in terms of vaccines. There could be diagnostic innovations. There could be financing innovations. All those are equally important.

**Interviewer: So how do all those work together to enhance the capacity of the health system to withstand crisis?**

**Respondent:** They are supplementary. They are synergistic. They work together basically. So, unless you ensure that each of those pillars is strong, the rest will not function. For example, if you look at human resources, even if you fund and build hospitals, but you don't have the human resources, you can't deliver the health services. But among all these ones, one of the typical WHO framework, I added community engagement.

**Interviewer: Yes.**

**Respondent:** So, community stroke stakeholder engagements. If that is missing, then you literally can plan the best health system there is on earth, but people will not use it and that practically won't help. Okay. Yeah.

**Interviewer: Anything else you'd like to share in relation to health system resilience?**

**Respondent:** No, that is it.
