# Supplementary material for: Resilience as recovery: stakeholder perspectives on Uganda's health system response to infectious disease shocks
Source: Front Health Serv. 2026 Jul 22;6:1888814. doi: 10.3389/frhs.2026.1888814 (PMC13437620; doi:10.3389/frhs.2026.1888814)
Supplement: Supplementary file 2 [file Datasheet2.zip › KII15.docx]

**TRANSCRIPTION OF KEY INFORMANT INTERVIEW.**

**I:** Briefly tell us about yourself.

**R:** I am called XXXX. There is another famous name that I have, is XXXX

**I: [**Laughs**]** XXXX

**R:** I am a medical doctor, Epidemiologist, Public Health specialist. I am an Associate Professor of infectious diseases. I am the Director of the National Institute of Public Health. What else do you need?

**I:** For how long have you been here director (Position)

**R:**  Ah, since 2017, since 2017, yeah.

**I:** Ok thank you very much, that is a wealth of experience of portfolios that you have. Ah, could you share with us your view in relation to resilience of health systems and looking at infectious diseases?

**R:** Yeah, I think ahm, ah, if I.. If I recall, ahh, going back too…, to Ebola especially, but also looking at ah, [coughs] emerging and re-emerging infectious in general. Starting with HIV when we were still young, ahh, actually HIV came when we were still, yeah, when we were learning how to have sex.

**I:** ok,

**R:** Yeah, then you can imagine, we grew up with it, with all the devastating effects of HIV.

Now when HIV came, you know what HIV did to the health care system. Aah, it messed up the health care system so badly and aah, then when you look at ah ah Malaria, ah when you look at Tuberculosis. Now, some of those conditions, what it did to the health system, is actually what to the establishment of.. of vertical systems.

**I:** OK

**R:** Now, the establishment of vertical systems was meant to kind of buffer the gaps that the health system had in such a way that of course it is also true that most of it was driven by funding.

**I:** Hmmn

**R:** Driven by funding to them MoH. So, but nonetheless, ah, that funding system helped support the health system and actually, strengthened the health system. Because if you really look at ah many of those programs did, they did not only support HIV programing for instance, they actually ended up strengthening the whole system.

**I:** Hmnn

**R:** Now, when it comes to these other emerging infections like Ebola.

**I:** Hmmn

**R:** Ebola came in 2000 that is the first time we had Ebola. Actually, it took my mentor the one who picked me from the medical school, XXXX. XXXX is the one who picked me from the medical school. Actually, I entered XXX’s vehicle when the war was, that was peak of the war in 1989.

**I:** Hmmn..

**R:** And I remember my sister saying this young boy is very stupid. He thinks he is adventurous, now he is going to the peak of the war, what is wrong with this boy. He was in customs, because I am fairly stubborn, OK. So, he said, he thinks he is stubborn but he is going to die. So, it is XXXX who picked me from there, I left everything. In fact she is the one who even bought for me my first bag. Can you imagine we lost him! Imagine how I felt?

**I:** Hmmn.

**R:** So, if you look at the response at that time and that is why we lost so many stakeholders in that outbreak. Because out of 400, I think 443, or something, 145 died. It was disastrous. So, you can imagine the effect ah that Ebola had on the health system. Everything, was firefighting, was not streamlined, everything wasn’t correct and all that and then we had the Bundibujo outbreak,

**I:** Ahm

**R:** Which killed XXXX. That one actually I was already in the MoH. When that, because by that time I was much into HIV. So, I was not part of that response, though we eventually ended up training, I think I was meant to train in Luwero and other people when they sent to many places. But I was not so much in response yet.

**I:** OK

**R:** Now. So, the effect on the health systems was enormous, because the response was not streamlined. And so, when you look back and see was our system capable of absorbing the shocks of the coming infections, the emerging infections. Probably not! But at time, but now with the changing systems, then you could say that we have probably managed to ah, despite the fact that we still have the disease’s but there is a big difference between then and now.

**I:** OK.

**R:** When you look at in terms of health system resilience.

**I:** OK

**R:** Yeah.

**I:** Ah, you talked about the vertical systems could you expound more on that?

**R:** Now, the vertical systems that were establish to mainly were disease focused. So, you find that Malaria control program, that is actually when they created the position for program manager in the MoH. So, you find that malaria control program has a program manager.

**I:** OK.

**R:** Under the commissioner, National disease control, you find the commissioner is in charge of TB, in charge of Malaria, now, under him he has very powerful program managers.

**I:** Hmmn

**R:** You see that? So, these program managers are very different from the other principle medical officers. They are very powerful. You see that? Because they control a huge, whole program with all its, so, you can imagine the MoH ended up actually setting systems around those disease programs. You find that they have their own surveillance systems, their own administration system, you see that? So, there are many things that ended up becoming parallel. Now, in essence these were meant to weaken the health system but because they were heavily funded programs, they ended up actually, strengthening the health system indirectly because that is what it was meant to do, but they ended up. So, you realize that setting up vertical systems weakens the health system, but, because of the heavy investments, they ended up actually strengthening the health systems indirectly. You find ah they are sending gloves for HIV programs, those gloves actually end up in the surgical ward. [loughs]

**I:** Wow!

**R:** [greens] yeah, so, because I mean the in charge of the health facility is not going to allow doctors walk out of the theatre because there are no gloves and yet we have gloves packed somewhere for HIV programs. So, indirectly they ended up ah, strengthening the health system. Yeah.

**I:** OK. So, you have talked about this resilience, health system resilience, ah, in your own words, how would you define it. How would you make a lay person understand it?

**R:** I think is, Me I would define it to a lay person, I would define it is the ability of the health system to withstand ah, the effect of disease occurrence. So, if a disease occurs, is the system able to withstand it in terms of first of all, I mean there are four series, because if the disease comes, for the system to withstand it, the system should be able to detect it. So, that means the surveillance system must be perfect.

Now, for that surveillance system also to function well, yeah? The condition must be diagnosed. So that means that the Laboratory system must work.

**I**: Hmmn

**R**: OK?

**I**: Hmmn

**R**: Now, for that alone to come, and reach the district official, and then eventually the MoH, they must be a system to relay that information and then when the information is relayed, what happens?

For you to be able to control the outbreak at source, there must be a team that actually goes within the stipulated time, in our case it is actually, 24 to 48hrs. So, that kind of ability to withstand the effect of the disease for me is what I will call resilience.

The system is able to pick, control, and then also manage the people who eventually fall sick. And then of course, the most important thing actually about health system resilience for me in infectious diseases is being able to first of all to stop transmission. Cut transmission. Now then when you cut transmission, then manage the sick.

**I**: OK.

**R**: Yeah, so, the health system should be able to do that and for me once the health system does that, I will call that a resilient health system.

**I**: Ok, ok, ok.

**R**: So, that is how I will define it to a lay person.

**I**: So, I was following up on that, what factors contribute to the ability of Uganda’s health system to be resilient.

**R**: I think the ability if the health system, Uganda health system to be resilience normally one is ah, is ah, is the infrastructure.

**I**: OK.

**R**: Yeah, the infrastructure, then two, ah because the infrastructure should be able to first of all to ah, there must be shelter to start with. Because you are not going to run a system where you have nothing. I know we are going to talk about the community set up. But even in the community set up, that community man there you need to give him a bag. That is why we have,.. ah no, I think I took all of them to, I would have given you the ones we gave ah, ah, the Village Health team members. Very beautiful bags, you see that?

**I**: Hmmn..

**R**: Because we do not want rain to enter into his things.

**I**: Hmmn

**R**: So, there must be first of all where you keep things safe. Then two,

**I**: [Interjects]Like an office?

**R**: Yeah, like a shelter. Like an office, you can call it an office, call it a clinic an OPD, so, [Interjection by interviewer, ‘’a shelter’’] Yeah, and equip it from where you sit, to where they examine the patient to where you do the laboratory, to where you store medicines.

So, there must be that infrastructure. And then two, there must be people to run that infrastructure. There must be personnel.

**I**: Hmmm

**R**: Trained personnel to actually run that system. Starting with the Askari. Because if the askari is not there at the gate things can turn upside. You start with the askari up to the in charge. So, the whole of that, set up of the health workers. Because in whatever, in quality improvement they say behind every step, there is a human being, behind every step. So, there must be a human being, a useful human being who is trained and capable of managing things well and effectively.

**I**: Hmmn..

**R**: And then of course the other one is the money.

**I**: Financial resources.

**R**: Exactly! If the money is not there, [laughs] you won’t manage. It goes back to when I was telling you about the vertical systems.

**I**: Hahahaha [loughs]

**R**: They ended up being, you can imagine actually right now, if I take you back to financial resources. Do you know because of Trump, the MoH said we are integrating services?

**I**: Yes, and it is the talk now.

**R**: Yeah, I don’t want to contradict my PS.

**I**: [Loughs]

**R**: She said “Those guys give us Peanuts” Ahaa, you delete this on record.

**I**: Hahaha. No, we won’t capture that.

**R**: They give us peanuts, Yeah, “we are going to integrate service”

Now, you say you are going to integrate systems, you still left the program manager malaria control program, the program manager TB control program is still there! You see that? I mean that is where you start from! Hahaha [laughs]

**I**: Hahahah [laughs]

**R**: Yeah, so anyway you need money. Ahn, so if you want to see, the effect of this whatever, the implementing partners pulling out, you are going to see our system that we have been proud of that it is getting resilient, you will see how porous it becomes.

**I**: For real?

**R**: For real, I have told you. For real in this country, this one is now real, you can even record e, I don’t care.

**I**: Hahaha [ Loughs]

**R**: Its going to be so bad that you won’t believe it. So, funding must be streamlined. So, if funding is not there, it is a problem.

**I**: Hmmn

**R**: Yeah, it is a problem.

**I**: OK. What about in terms of leadership how does that attribute to the ability of the system to be resilient.

**R**: Yeah, it does. First of all, for all these things I am taking about, it requires very strong leadership. It requires leadership from the highest level. Okay?

**I**: Hmmn..

**R**: to the lowest. To the health facility.

**I**: Highest level what do you mean, the MoH?

**R**: in this case we can put the MoH, because the minister operates on behalf of the president. Let us put it at the Ministry level.

**I**: OK

**R**: It needs very strong leadership, if you don’t have strong leadership, then there is a problem. Because the minister must be able to present her issues in parliament and attract money. You see that?

**I**: hmn,

**R**: so, that one should be there. And then you should have that strong leadership to set up systems which work and it takes me actually to why we set up the National Institute of Public health.

**I**: OK

**R**: It goes back to the way we were doing things, we were doing things in haphazard manner as I gave you the examples of Bundibujjo Ebola and all that. The reason why we had this meeting in 2013, I was still in HIV when we had the first meeting when Jane was still the director general, when we had that meeting.

**I**: OK

**R**: And in that meeting that is when we conceptualized the National Public Health Institute and eventually, we streamlined everything and that time we did not have the National task force.

**I**: You didn’t have?

**R**: No! we didn’t have. We didn’t have the organized National rapid response team that we have. We did not have the emergency operation services, we didn’t have a frame work for instance.

**I**: So, when it came?

**R**: Yeah, it is actually how we set up this system and yet some funny fellows are down playing it and think things just happen. You wait after 30^th^ of September, I am going to ask them fundamental questions. I will be in my farm.

**I**: hahahah [laughs]

**R**: Yeah,

**I**: Ok, what about community engagement, how do they contribute to the ability of the health system to be resilient?

**R**: They do a lot. Actually, ahm you know, communities … [sighs]

**I**: Hmmnn.

**R**: All diseases, start and end in the community. That is the truth of the matter. All these outbreaks we struggle to control start in the community! Even it starts here in Kampala, you start with Kisenyi. You remember Typhoid, I don’t know whether you recall typhoid? [Hmmn, hmmn] the one we controlled, that is when we had just opened up the first group of the field epidemiology program. The first time that we responded to whatever, happened in the heart of the city.! But it was among the community. You see the point?

**I**: Hmmnn.

**R**: so, the community it I where all these things end.

So, number one, you need the community that is informed. You need the community that ah, that are able, ah.. to say this is wrong, not to hide things.

**I**: Hmmnn..

**R**: yeah? So, the community must also be cooperative. The community must also be able to report whatever comes and the community must be able to mobilize itself to educate, but also mobilize themselves to help the ministry. There are many things that we do at community level when you.. When you hear that we are doing community education, number one we are giving them information, but two we want them to pass on that information and three we want them to participate in the control.

**I**: Hmmnn

**R**: Because if we say that don’t drink that water, the community must say that water there, there is no drinking it until they say we drink them and nobody should go there. For you the experts you have already packed your bags.

**I**: And gone back.

**R**: You are no there. So, if the community does not help you to say no, we have been told until we are told open this water please don’t drink, you will find everyone is dead. So, the community plays a very fundamental role in control.

**I**: OK

**R**: They are the ones that are going to mobilize. You look at vaccinations, Vaccination program, if there is vaccine hesitancy, where does it start from?

**I**: In the community.

**R**: The only one actually where we found vaccine hesitancy amongst health workers, is COVID-19.

**I**: Hmmnn.

**R**: It COVID-19 in all the studies we did. It was COVID. The health workers were the biggest problem and because the health works have a lot of mistrust in government. [laughs] So, but the rest is always community. So, the community is a very fundamental thing in health systems.

**I**: Ok.

**R**: Yeah, and they are the ones who understand themselves. Because you know each of those communities are unique. There are people they listen to, when they speak, they keep quiet. There is respect that they give to people in that community. You go there with your whatever, even when you have put on your medical uniform or coat or whatever, they can sit like this and look at you and then they go behind and say look at that fool there. Hahaha… [laughs]

**I**: Hahaha… [laughs]

**R**: [laughs], because they know their culture. They know those things very well.

**I**: Hmm...

**R**: And they certain messages that they even tell you that aah, that one doesn’t talk about it here.

**I**: Hmm...

**R**: Yeah, they know what to say.

**I**: Hmm…

**R**: So, you need to be cautious of the whole community. So, the community is a very powerful stakeholder in maintaining, you know, what we call a good health system at that level, you need the community.

**I**: Ok, ok,

**R**: Yeah.

**I**: OK! I thought we take the health facility as high up there and not the community,

**R**: No! everything starts in the community. The health facility you see there, if the community says that those health workers there, we don’t understand them anymore, you are closed. *[health center]*

**I**: They will not come.

**R**: They will not come! They can even cross there and go 5 miles away to another health facility. Hahahaha *[loughs], they do that. Hahaha [Loughs]*

**I**: Ok, Alright. so, we need to understand the community when doing this?

**R**: Yes, very well.

**I**: Ok, ahn. What do you see as the interventions or actions that contributed to the Uganda’s health system to be resilient.

**R**: For me I think ah, I think what has contributed majorly is the heavy investment in health with of course support from especially partners that is the truth of the matter. We need to give credit.

**I**: Hmnn

**R**: You know because I have been part of this health system from my leaving the medical school. Hmnn?

**I**: Hmmnn.

**R**: To todate. I have seen it evolve.

**I**: OK

**R**: the evolution over time, because, all my life I has been under Museveni. So, I am not comparing with the so-called good system of previous government of Obote, or whatever, I am just talking about what I am seeing on. The building up of the resilient health system was majorly, heavy investments, you know with support of partners. That is the truth of the matter.

**I**: Hmnn

**R**: So, that heavy investment, actually has built up the system. So the major system which we have of course with contribution from government through MoH. One is by putting favorable policies, so those favorable policies are good and then of course plans.

**I**: Hmmn..

**R**: Development of guidelines.

**I**: Hmmn.

**R**: Much as it was with the help of the partners, or whatever, the partners were the brains behind it.

**I**: Hmmnn.

**R**: So, all those, the favorable environments, the policies, the plans.

**I**: Hmn,

**R**: Okay, helped build a resilient system. And then of course the capital investments. The investments of the infrastructure, we talked about which has been put up, you know hospitals built, health centers built, equipped with laboratories, diagnostic capacities, very OK.

**I**: Hmnnn.

**R**: You can imagine when, as I told you that when we actually narrow it down response of public health emergencies.

**I**: Hmnnn..

**R**: When we did not have all these systems, then we started setting up these systems that is actually when we built up that laboratory. That laboratory is actually the campus of the public health institute. That laboratory you see there is the campus.

**I**: OK

**R**: Yeah, so, when we set up that laboratory there, when we were still in HIV, we started the early hand diagnosis program. But eventually it was now expanded when started using dry blood spots. And we expanded it, that is when we opened up that laboratory, remember we started, and all that money, was CDC money. From construction to completion. When we started public health there was no people, here we had about only two people.

**I**: Hmmnn

**R**: Analytical lab, XXX and XXX, two of them were there We pulled them and took them to there is a building on Buganda Road.

**I**: CPS?

**R**: Yeah, that is where we started. There is a building on Buganda Road where there is GT Bank.

**I**: Yes,

**R**: The next one. That is where we started. I used to sit on level 4.

**I**: Oh! OK.

**R**: When we were in Aids control program, we were the ones and then we started building the laboratory. And that is how National Institute of Public Health came about and we go and opened up in Butabika. It has been evolving whatever, The Gen-Z came later, they do not even know some of these things.

**I**: Gen-Z?

**R**: Yeah, they come in later. You can imagine, we built up the National Lab sample and results transportation system which is now of the best examples you can give in Africa.

**I**: The hub system?

**R**: Yeah, the hub system. It wasn’t there!

**I**: OK

**R**: It wasn’t there! We started it on, and I saw it evolve like this. Now we added on transportation of outbreak samples, still through the hub system., we only added courier systems because you can’t you can’t work without them.

**I**: OK. True, yeah.

**R**: You see that? So, all those ones were heavy investments.

**I**: Hmmnn.

**R**: Heavy investments that were done. Which has made that possible now, so you can test. In the Ebola of 2000.

**I**: Hmnn.

**R**: It took us 86 days to get the results from south Africa. When you tell people here they don’t, you feel like ah ah no, no. 86 days for us to get the results for confirmation.

**I**: Hmmnnn

**R**: Now, when you get the samples like this, you will see it pop into my phone, the moment we sent it through here like this, because we can now even do the tests here in Butabika. You see that?

**I**: Hmmnn..

**R**: Within 8 hours you get the results and we know what is going on, and we are sending teams to the field. You see that?

**I**: Hmmnn.

**R**: So, those are all changes which have happened because of heavy investments.

**I**: Hmmnn…!

**R**: The National Institute of Public Health itself. The national response team led by fellows, is the best thing that happened to this country in terms of ah, you know ah, chang in response.

**I**: Hmnn..

**R**: The trajectory of response has totally changed because of that. So, this was a heavy investment in capacity building. Hmnn. Now we have graduated so many that are actually everywhere. They are here in the MoH, they are in WHO, they are in CDC, some are in the school. You see that?

**I**: Hmmnn.

**R**: They are in many of these places, but most importantly is the response that they lead. Now, if you add on the laboratory leadership group, which are all here.

**I**: Hmnn.

**R**: You add on the modeling group which are all here. Add on the health informatics group which are all here. Now, that heavy investment has done a lot in terms of building resilience of the system.

**I**: Hmmnn..

**R**: Because we are able to pick, to pick.. then also the other thing is also building up the system of the surveillance system. The robust surveillance system that we built.

**I**: OK.

**R**: Has also, helped a lot. Because we are able to pick these things and also of course setting up the emergency operation centers which was also part of the institute. It has transformed response in many ways.

**I**: Hmmnn

**R**: In many, many ways that is the truth of the matter. So that heavy investment has really really done and then of course the other thing also is the change in technology.

**I**: Hmmnn!

**R**: Yeah, there is also better technology. Now, so, you can also say that you are able to do some of these things here because of the changes in technology, we are able to do it faster than we could have done before.

**I**: Hmnn

**R**: And that is OK. That has also helped in one way or the other.

**I**: Hmnnn..

**R**: So, when you add on all those things from policies, planning, and all that and then of course budgeting, the funds and support from partners, have been actually critical.

**I**: Ok

**R**: Yeah,

**I**: Ah, what about preparedness. I heard you talk about trainings, fellows, and the money, how has that led to preparedness and also link it to the health systems.

**R**: Yeah, I think the good thing about preparedness is about one, first of all one we have learnt lessons. We have learnt a lot of lessons along the way. So, those lessons along the way has helped us plan and prepare.

**I**: Hmnn.

**R**: Then also we have the National rapid, rather ahmn.. response plan. National response plan which we have is now actually by pathogen. Is by disease, we don’t have like general we have it by disease.

**I**: OK

**R**: So, we just change and go through.

**I**: Hmmnn.

**R**: The two, we have the emergency operation center which is in watch mode all the time. It is only activated to another level. So, it is in watch mode all the time.

**I**: Watch mode.. haha [ loughs]

**R**: Yes, and then the other thing is that because of the fellows which we have, because they have universal access to DHIS2 system. So, they analyze data all the time periodically and even they have sent me some of them there, I am going to look at them. They analyze data all the time, so, we are able to track patterns of disease.

**I**: Hmnn.

**R**: People there saying whatever, but we are doing all that. So, because of tracking those patterns, that is part of preparedness.

**I**: Hmmnn.

**R**: Because we can actually detect and say, hmm! Hmm, there is something here which is going we don’t understand it.

**I**: Hmnn.

**R**: yeah, so we receive alerts all time, because the EoC is in watch mode.

**I**: Hmnn.

**R**: We have people who are analyzing this data, looking at changes in patterns. Like malaria channel, we actually detect these things much early and whatever, because we monitor, what is going on. Yeah, so all that is part of preparedness.

**I**: Hmnn..!

**R**: And then now, when it comes to response plans it is all part of preparedness.

**I**: Hmnn…!

**R**: And if you see by the way, some of our responses which have been so quick. Ahh, we had Ebola 2019 which we imported from Congo, we lost 4 people, none of them was a Ugandan, all were Congolese, they died in Uganda. We never got any community transmission. We had prepared the communities through, from Bwera, from whatever actually the whole of Kasese, they were through.

We had just transitioned, and then when we had done all that, yeah?

**I**: Hmmnn..

**R**: We were, still even winding up even with that imported Ebola, we had even revised our response plans and all those kinds of things, then COVID strikes. You remember COVID?

**I**: Not the Marburg of 2019 in Kween?

**R**: It was earlier, that was actually not 2019, it was 2017.

**I**: Oh!

**R**: That Marburg, I have the paper. That was 2017 when we went up to Kenya?

**I**: Hmnn.

**R**: It was 2017, yeah, so, 2019 was Ebola.

**I**: Hmmnn.

**R**: That was the serious one. But it was imported and we lost only 4 people, because we were prepared.

**I**: Hmmnn.

**R**: Then we transitioned actually to COVID, with that level of preparedness.

**I**: Hmnnn

**R**: So, that is why actually, we were among the best countries to control it apart from when they opened up and we started having community transmissions.

Our first rapid assessment, we didn’t have any community transmission.

The second rapid assessment then we had, when they opened up.

**I**: Hmnn

**R**: The second wave. When they opened, that is when we started getting community transmission which overwhelmed us and we couldn’t do control any more, hahaha [loughs]. We had actually done very well, and all that because of preparedness.

**I**: Yeah,

**R**: And then also in preparedness we had, things like we now begin giving them out and whatever. Like now when you go to the EoC or whatever, supplies, yeah, we have them but all these actually because support from partners.

**I**: Hmnn..

**R**: Yeah,

**I**: So, paint for us a picture of what a resilient health system would look like? What are those characteristics?

**R**: Ah, I think, for me a resilient health system will be, a, ah.. would be.. [takes time], a system which ah, which addresses ah, which addresses the needs of the population, ah, from the perspective of ah, of ah preventing illness. If the system can prevent illness, that system should be able to provide proper hygiene, proper sanitation. So, first of all the system must prevent disease. That is, to me that is a resilient system. Prevent disease! Now, if you cannot prevent this disease 100% which is, you can prevent 100% it is difficult.

**I**: Hmmnn.

**R**: OK? A few that get infected. OK. First of all, In the system, you must detect it.

**I**: OK.

**R**: In the record time. Then when you detect it in record time, those who get sick must be treated. The system should be able to treat. SO, that means that you have the medicine, you have the diagnostics, eh!

**I**: Yeah,

**R**: Yeah! You have the people who are able to treat, eh!, if it requires surgery, you have the surgery. You the ambulance system, you have the transportation systems.

**I**: Hmmn

**R**: So, the referral system must be perfect at that level. That is a system.

**I**: The referral system

**R**: Exactly! The referral system. So, you have all that. Now, once you have all that whatever, then there are a few, that definitely may end up having some bad effects, hmm! you must be able to rehabilitate them.

**I**: OK

**R**: So, if you cannot rehabilitate the people, that is not yet a good system. Because not everybody is going to recover from whatever. You have seen people come out and whatever they say this one is walking like this, so you must have physiotherapy, so that rehabilitation system that must be OK.

**I**: Hmnn

**R**: And then of course the.. the other bit of it is now the something that we rarely, although now incorporate it in is the psycho-social support.

**I**: Oh!

**R**: Yeah, the whole thing is. You know sometimes when we are discussing health, what is the definition of health?

**I**: Hmmnn [Loughs], I can’t put it together.

**R**: Hmn, so, health is a state of complete, physical, mental, social, spiritual wellbeing.

**I**: And spiritual?

**R**: Yes!

**I**: Hahaha [Loughs]

**R**: I have told you, [Lughs] spiritual well-being and not merely the absence of deformity.

**I**: Yes,

**R**: That is the definition of health. So, you need to

**I**: interjects [ Spiritual wellbeing?]

**R**: Yes, you see the problem when you say spiritual, for you are thinking religious?

**I**: Yes. Hahaha [Lough]

**R**: No, spiritual is not religious. It is to connect with your ancestors.

**I**: Hmnn! OK.

**R**: That is a good thing. You know religion has made us become funny.

**I**: Western religion?

**R**: Yeah, and the whole idea let me tell you. Do you pray?

**I**: Yes, I pray to the above.

**R**: If we don’t change our attitudes to religion. Do you know what these guys did?

**I**: Hmmnn..

**R**: Do you believe that spirits don’t die?

**I**: Yeah!

**R**: Spirits don’t die!

**I**: Yeah!

**R**: Now, do you know what these guys did? And then we find ourselves in situations of perpetual bad luck. Do you know what? They made us disconnect ourselves with ancestors we think that our ancestors are evils.

**I**: Are evil. Correct!

**R**: That is the first thing these guys did!

**I**: Hmmnn

**R**: So, you find you are in perpetual problems you don’t know. You detached yourself from your ancestors.

**I**: Hmnn.

**R**: And they are very unhappy with us. That religion oh! Bla, bla. Nothing! Just making you foolish, because you don’t understand.

**I**: Hahaha [Loughs]

**R**: So, when you are talking about spiritual,

**I**: You be like sheep.

**R**: Yeah, you be like you end up being like sheep. They can just take you and slaughter you there. Yeah? We need to rediscover ourselves Africans.

**I**: OK. The spiritual one.

**R**: Yeah,

**I**: OK, OK, Hmnn, Hmnn.

**R**: So, so, the whole thing is about really that completeness.

**I**: Hmnn..

**R**: So, the resilient system must look at that kind of system. But then of course eventually during whatever, is ah, is now eventually, as all those things, is basically education. It is community Education.

**I**: Hmnn.

**R**: So, that system now, once you whatever, set up systems well, the community education, first of all you there must be education about all those services we are talking about.

**I**: Hmnn,

**R**: The prevention, yeah. If we say that we eventually have all these vaccines available, educate the community that vaccines are available. If there is testing of women, the screening, services are available, make the community know. So, education of the population.

**I**: Hmnn.

**R**: hnn, Eh, if you see the way we, when we were growing up, we had these, ah, these, ah, I don’t know how you call it in ah, in Teso. The health inspectors. We called them, ah you know, it is like ah, like ahaaa.., like a doctor of hygiene if you translate it in my language in Lango.

**I**: Ok.

**R**: Is like a doctor of hygiene. So, and these guys when they are coming, you could see how the homes are clean.

**I**: Aha!

**R**: Yeah.

**I**: And then places for putting plates [Plate stand], water somewhere near the toilet latrine. Yeah, I remember.

**R**: Yes, the granaries, clean. Toilet clean! everything, cassava gardens, shining.

**I**: I remember that time.

**R**: Casava gardens which was never touched whatever, [weeded] and culturally, they were granaries, and culturally for us, it is only women who climbed in the granary. You climb the granary, the clan will whip you! Aman, climbing a granary?

**I**: Hahaha

**R**: Now those cultures are gone, the granaries are not there.

**I**: The granaries?

**R**: Yeah, you see how powerful it was in the community.

**I**: Yeah the granaries are not there.

**R**: Yeah, in the communities are not there. Now that kind of education now when people are whatever, you find that you have chased away the disease very far away.

**I**: Yeah.

**R**: The questions of diet. What kind of food do you cook? You see that?

**I**: Hmnn.

**R**: So that kind of community education, so now you have averted a lot which is going to stress the health system at facility level.

**I**: Hmnn.

**R**: You have averted many things. So, for me a resilient health system must have those complete set up.

**I**: OK.

**R**: And then of course even the treatment whatever, it goes back to response. The way we set up response system. That is a resilient health system. Because now, we are able to respond within between 24hrs to 48 hrs.

**I**: Hmnn.

**R**: That is why, I am saying that we are proud of this, we set up a system which works, some people are trying to dismantle it unfortunately, but HE. Trump has dismantled it better for them. They were trying to disorganize us, dismantle systems, but Trump is now doing it better for them.

**I**: Hmmnn.

**R**: I told you wait after 30^th^ September, you will see for yourself. You will see what will happen.

**I**: Hmnn.

**R**: So, that is a system that you be proud of as a resilient system. You control public perception, you have everything that you need, that is necessary for you to respond.

**I**: Hmnn

**R**: You have vehicles, like for us our vehicle now is down. The drivers are not whatever, the other one I went with in Bombo, others are there, but let something happen like this, we are already on the ground. Even when the DG [Director General] is till calling everyone, even before the National task force sits, the team is already going.

**I**: You are already gone.

**R**: The team is already gone!

**I**: Wow.

**R**: So, that is the kind of system you will be proud of.

**I**: Ok.

**R**: Yeah.

**I**: So, what do you think are some of the Unique attributes about the Ugandan health system that make it more resilient.

**R**: I think ah, Ok. What I would say is the.. ahm, is the ability to adapt, to changes,

**I**: OK

**R**: Ahn, two, is the, ahn.. is the adoption of tools,

**I**: OK.

**R**: Ah, of policies, guidelines, response. Tools generally.

**I**: Hmmnn

**R**: And guidelines, which has also helped.

**I**: Hmnn.

**R**: Ahn, is the ability to also to, to attract technical assistance in terms of ahhn expertise.

**I**: Hmnn

**R**: Yeah, that exchange of expertise builds up systems,

**I**: Hmnn.

**R**: And then aah, aah, the other thing also, I think which I would ah, it is a dubious distinction but is it. It is that we have had many outbreaks.

**I**: Hmmn, I wanted to ask you about exposure

**R**: Yeah. Because we have had many outbreaks, it has given us the opportunity to learn while doing.

**I**: Hmmnn.

**R**: So, that is how we are actually, top notch in the whole health security, because we learnt it while doing.

**I**: Hmmnn. OK.

**R**: Yeah,

**I**: You had talked about the role of the community and said that they are very, very important. Could you expound more, in case there is anything that you left out. About the role of the community in health system strengthening.

**R**: I think for me, the only thing I can say is that the community should always be consulted.

**I**: Do we consult them always?

**R**: I am telling you. You should always consult the community.

**I**: Hmmnn.

**R**: We should, whenever we do anything. You see consulting the community, you can do it in many ways.

**I**: Hmmnn.

**R**: Aah, we have structures, that penetrate down to the community.

**I**: Hmmnn.

**R**: I will give you one example. I am a cultural leader, did you know that?

**I**: No, you are telling me now. Hahaha [Laughs]

**R**: Hahaha. So, you see as cultural leaders, Eh!

**I**: Yes..

**R**: Cultural leaders, we have our big meetings of cultural leaders. Now, you tell cultural leaders all these things here, do you know where it ends?

**I**: Yes, in the community?

**R**: Yes, that is it. So, you need to find ways of making sure that the community is consulted. Because the cultural leader will go to people and say, now, MoH came up with this.

**I**: Yeah

**R**: Hmn!, what are you views? You see that?

**I**: Hmnn,

**R**: Then say, OK, we were taught, we think it is a good thing what are you views. The response from people will be ah, that is very OK, that is fine. Because you consulted the community, because cultural leaders know what goes on in their community all of it.

**I**: Hmnn

**R**: So, I was giving you just an example. So, we need to find ways of consulting the community in whatever you do.

**I**: Hmnnn

**R**: If you don’t consult the community, it is just like if you want to go and construct a health facility. You need to gather the community and say, we think the other facility is far is actually on the other side. We think that we should construct a health facility here, and this one is going to be a level three, what do you think? They will say that is a good idea, we have no problem. So that is an example. So, consult the community.

**I**: Hmnn

**R**: Then number two, use community structure.

**I**: Hmmnn.

**R**: Use community structures.

**I**: Those are the LC’s, the VHT’s?

**R**: Yeah, you can use the VHTs, LCs, the Cultural leaders, the Opinion leaders.

**I**: OK. We also have opinion leaders?

**R**: Eh! Communities have opinion leaders. In fact they are people who

**I**: [INTERJECT] in politics or health?

**R**: Let me tell you, some of them may not even want to join politics. Let me tell you, they people who simply respected.

**I**: Hmmnn

**R**: Let me tell you there are retired head teachers.

**I**: Hmnn. Head teachers.

**R**: Retired head teachers, who don’t want to join politics. First of all, they are towns are very pity. If I tell you that I know now you understand what I mean.

**I**: Hahaha [Loughs]

**R**: They don’t want politics, they are just busy saying that man is hopeless.

**I**: Hahaha [Lough]

**R**: Now, those ones when they speak.

**I**: Yeah, yeah.

**R**: Eh! The community listens.

**I**: Oh! Ok

**R**: The community listens, there are such people, those ones never join politics. Such people.

**I**: Hmmnn.

**R**: But they are highly respected and they are intelligent.

**I**: Yeah.

**R**: And they have grown up in that community, they have institutional memory that the young people do not have. So, those ones are very instrumental if you want to penetrate the community.

**I**: Hmmnn.

**R**: Yeah, they are very important. They know a lot about their community. They can even tell you for example [“I am telling you don’t do that”] and he sits down. [sighs] now, there you have to change tactics, if you don’t change tactics, you are wasting your time. You will fail your program.

**I**: Hmnn.

**R**: So, you need to understand those things. so, communities are really, really good. Yeah.

**I**: Ok

**R**: Yeah.

**I**: Thank you very much. So, ah, much as you have said that we have a resilient health system, ah, I believe there are some challenges and I would like you to mention some of them some of the challenges or obstacles that affect the health systems ability to withstand or recover from any of these outbreaks.

**R**: Yeah, I think ah, one of the challenges is parallelism. Parallelism in the sense that, you see, ah, you see the way we operate, ah MoH, you know disease occurrence.

**I**: Hmmn

**R**: If you build a triangle of a disease, do you remember?

**I**: Hmmn

**R**: ahm, with the host, the parasite,

**I**: [Interject] And environment/

**R**: You see that. Now, if you look at the way diseases occur, right from when we are saying that the disease occurs from the community.

**I**: Hmmnn

**R**: You find that, MoH, has an aspect it controls, there are also aspects it doesn’t.

**I**: OK.

**R**: look at the zoonotic diseases.

**I**: Yes.

**R**: hmnn! Zoonotic diseases which occur. A huge chunk of it of course it comes from animals.

**I**: Hmnn.

**R**: 70%.

**I**: Hmnn

**R**: And not vise versa. We human rarely infect animals by the way.

**I**: OK.

**R**: It is rare. It is occasional when we infect gorillas, when we infect chimps.

**I**: Hmnn

**R**: It is occasional but the other way is true.

**I**: Hmmnn.

**R**: So, most other infections are from the other side.

**I**: Hmmnn

**R**: But we don’t control it. We don’t control what goes on in the sanctuary in the wild life, we don’t. You see the point.

**I**: Hmmnnn

**R**: Now that inter-relationship, so, that is what I am talking about parallelism.

**I**: Hmnn

**R**: We operate as if diseases occur from human, to human. And where diseases come from, we have no control,

**I**: Hmmn.

**R**: Look at the environment. When disease occur like in snails, in like whatever, we say those are NEMA guys, they are busy with their whatever and they have not done their part.

**I**: True.

**R**: So, that is where the problem comes from. So, we need systems that intersects.

**I**: Hmmnn

**R**: So, that parallelism is not good.

**I**: Hmmn,

**R**: For a good health system.

**I**: Hmmn

**R**: And two, is also the human resource, because it is the human resource that are necessary.

**I**: Hmn,

**R**: For managing that kind of system there, you must be trained to understand and also intersect with each other.

**I**: Hmmn,

**R**: But here, they don’t. That is actually, what they call the one health approach.

**I**: Hmnn,

**R**: Yeah. So, you find the vets are doing their own things,

**I**: Hmn,

**R**: Even at one health approach, they still think managing animals it is only theirs. One time, I challenged them and said, “You guys, you are the ones killing this one health” And one health actually we brought it to the lime light because of us. Had we left it to the vet, we would have buried it.

**I**: Hmmn,

**R**: And that is the problem.

**I**: Hmnn.

**R**: So, some of those things do happen, which of course, ah, it is not good for the health system. If you look at JEE. You know JEE?

**I**: No.

**R**: Joint External Evaluation. It one of the things we do to kind of measure how far we have gone through compliance to international health regulations.

**I**: Hmn,

**R**: We have 19 technical areas, that we gauge ourselves.

**I**: Hmmnn

**R**: It is a country engagement.

**I**: Hmn

**R**: And we judge the whole country. We do not judge the health sector.

**I**: Hmn

**R**: When you look at all the 19 areas, every time, you whatever, you know the people who bring us down?

**I**: No.

**R**: The environment, MAAIF, they bring us down. We are supposed to score 5, green,

**I**: Hmnn

**R**: Dark green, but you find us in red or in yellow. We have ever quarreled in meetings because of that. So, that is the way the system was set up. So, we need to ensure that if we want a strong health system, we need inter-linkages.

**I**: Hmnn,

**R**: But then the other one of course is ah, for me what I would also say is a problem, is poor prioritization.

**I**: From who?

**R**: That is now from the planning side. There is poor prioritization by government.

**I**: Hmnn

**R**: Yeah. We need to prioritize, we need to know which, what starts where. We don’t know where our money goes.

**I**: Hmnn.

**R**: Yeah. So, we need to prioritize well. Because if we do not prioritize well, there is a challenge. We are not going to put money where it matters less.

**I**: Hmnn,

**R**: So, one of our biggest challenges is poor prioritization then of course now, I will now narrow it down to lack of funding.

**I**: Hmnn,

**R**: But if you see it carefully, it is poor prioritization. We baptized it as lack of funding but if you go deep,

**I**: It is not lack of funding.

**R**: Not, look at these fools here who are telling us to hanged in military courts. They are there passing that bill into law, they have already passed it.

**I**: Are you sure?

**R**: They have already passed it. Because they were paid 100 million the other day, you remember?

**I**: Yeah,

**R**: And they were paid 50 million each yesterday again from state house.

**I**: [Sighs] Oh God!

**R**: Now you tell me all that amount of money. 150 million per MP.

**I**: Time 200 plus.

**R**: Calculate. Is that good prioritization? Then we say we don’t have money. So, we baptized it as lack of funding but it is poor prioritization.

**I**: Hmmnn

**R**: Then of course the other one is the ah, is still, the bit of it I will say, we also have gaps.

**I**: Hmnn,

**R**: In the, in human resource.

**I**: Hmn,

**R**: Gaps in terms of numbers and human resource.

**I**: Hmn,

**R**: Because, we are strong here, but we are not strong everywhere. Yeah.

**I**: OK.

**R**: Yeah, so, there are still gaps. Big gaps to attend to. Yeah. And then the other thing also is which are, ah, which takes me bact to the community we talked about a lot.

**I**: Hmn,

**R**: We need the group that understands the community. That should be part of the health system. You know, it is like in response, it is one of the systematic reviews I have just done which actually found out about, the, in response you find actually that we, rarely use social workers. We rarely use the Anthropologist, and that is a mistake,

**I**: In health?

**R**: Yeah, we need to understand the community and people who understand the community best are the anthropologists. The social workers, they understand the community very well. So, we need to be embedded in the health system because they understand communities very well.

**I**: Wow.

**R**: Yeah. These villages even if you send there these medical doctors, it is even worse, because a doctor is looking at things at a very high level and is not thinking about

**I**: [Interject] Hmn,

**R**: And is talking to people whatever in a hurry.

**I**: Hmn

**R**: If you find one, you get a medical doctor who has not done public health like some of us who have. He is talking to people anyhow and can even abuse the people if they ask him. Because they are taking too long to understand him.

**I**: Yeah,

**R**: Yeah. You see that?

**I**: Hmn,

**R**: We need people who can understand the community. For us we are fair, because we have done public health, we have worked with communities, we have interacted with patients in the community,

**I**: Hmn,

**R**: So, you need that that group. So, for me lack of incorporating that group in our structure, is also, a problem.

**I**: OK!

**R**: Yeah,

**I**: Alright. So, you talked about stake holders and the parallelism. How can we work better, to overcome that challenge. Or how can stakeholders work better to overcome this challenge.

**R**: I think we just need the ah, we need the, high level systems change. Because if you ah. And these things can be done, it is just a matter of building our systems that you, that intersect. Because if you have, MoH and this is the way MoH employs, if you a section in between, and make it strong and fund it. Let us say, ah, not necessarily calling it ministry of one health, you could call it ah, like you could make it, ah actually that is even what we had even proposed.

**I**: Hmn,

**R**: To make it one of the departments, directorate in the National Institute of Public Health.

**I**: Ahn,

**R**: Bring that group, on their own and fund it.

**I**: Hmn,

**R**: You will see a big change. It needs a deliberate effort of merging in a systematic way.

**I**: Hmn,

**R**: and putting in a system of government, which receives funding. You will see things change. With time you will find, if you are in the one health group, you even stop calling yourself a medical doctor or midwife, vet or whatever.

**I**: Hmn,

**R**: You, the name will merge.

**I**: surely?

**R**: It will merge somewhere. So, you will find a group there who understands the whole system.

**I**: Hmn,

**R**: Yeah, so, it needs a deliberate effort, if you are to promote it. The moment we start, planning, small, small, it won’t work, so, it needs a deliberate effort by government to address those sectors by actually bringing them together and creating the system which works.

**I**: Hmn,

**R**: Yeah. And funded.

**I**: Hmn, OK! Thank you very much. So, let’s look at the recommendations, ah, what would you propose as some of the strategies that would strengthen our health systems. Or resilience to infectious diseases.

**R**: Ahn, I think the strategies are is ah, number one,

**I**: Hmn,

**R**: We need to increase domestic funding.

**I**: OK

**R**: Domestic funding. Because you see, All these things you are talking about,

**I**: Hmn,

**R**: Now, we are going to wake up late.

**I**: Hmn,

**R**: But we shall wake up eventually.

**I**: Hmn,

**R**: We need to increase domestic funding.

**I**: Hmn,

**R**: That is number one.

**I**: Hmn,

**R**: Once you have domestic funding, you look at Traoré [President of the Transition of Burkina Faso] has done. Ibrahim Traore’.

**I**: Of Ivory coast?

**R**: No, Burkina Faso.

**I**: Burkina Faso, OK.

**R**: You know the man chased away the French.

**I**: Oh. Ok [loughs], I am behind that one.

**R**: He chased away the French, he has paid all the debts, of Burkina Faso, He is doing everything in, using Burkina money! He has said no more borrowing. World bank? I don’t want any World Bank, close your offices. IMF? Close!

**I**: Hmn! [WONDERS] That is good.

**R**: So, all of that can be done. So, we need to increase domestic financing. For me that is number one.

**I**: Hmn,

**R**: And then ah, number two, we need to adopt models that work.

**I**: Hmn,

**R**: Ah, I will give you an example. National institute of Public Health.

**I**: Hmn,

**R**: Is a tested model. It has worked everywhere.

**I**: OK.

**R**: Yeah, it has worked everywhere.

**I**: Hmn,

**R**: US CDC is a National Public Health Institute.

**I**: OK.

**R**: Yeah! That is the truth of the matter!

**I**: OK.

**R**: Look at Nigeria’s CDC. Ethiopian Public Health Institute.

**I**: Hmn,

**R**: These are institutions you can be proud of. They are models which have been tested and works. Look at China CDC,

**I**: Hmn

**R**: You can’t believe it. Go to Korean CDC. You can be proud of it.

**I**: Hmn,

**R**: It has done a lot for government, governments have injected the money.

**I**: Hmn,

**R**: And usually, they are domestic fundings. But also, even if you are getting foreign funding, is domestic funding injected?

**I**: Hmn,

**R**: Foreign funding actually, just comes in to substantiate a few things. So, we need models which work.

**I**: OK

**R**: And then, of course the other one which for me I think, is a recommendation, is, even if you are getting domestic funding, prioritization which is I said earlier.

**I**: Hmn,

**R**: Yeah, we need to prioritize.

**I**: Hmn,

**R**: Yeah. Put money where it really works and do things right. And then also we need to, we need to build ah, human resource. We need to build cadres.

**I**: Capacity building?

**R**: Capacity building of health workers. We need to the skills.

**I**: Hmn,

**R**: With skills that can transform the health system in this country.

**I**: OK.

**R**: Yeah, because you see, we have basic trainings, let me tell you, basic training, if you see carefully, basic training is good. But it prepares you for general things.

**I**: Ohn,

**R**: Yeah, it prepares you for general things. If you want to transform a country, you need to deliberately, do post basic. Post basic degree training like fellowships. I will give you an example, the fellowships that we are running here, if you inject money and have enough of this, you will see how you are going to transform your health system.

**I**: Hmn.

**R**: It will change! And when you are going out I am going to show you.

**I**: Specialization, right?

**R**: Yeah, because, these are fellowships that are competence based. If you have such people, in all categories that you planned, you can’t go wrong as a country.

**I**: Hmn,

**R**: You can’t! You can’t . It is highly skilled people.

**I**: Hmn,

**R**: Yeah.

**I**: OK, OK, OK, yeah. So, you have talked about models, ahn, are there any successful models that ah, you think about that suit our context.

**R**: I have given you the National Public health Institute.

**I**: Yes.

**R**: Yeah, that one is one model which there is no question about that, it will work.

**I**: Hmn,

**R**: An then of course, the, the ah, the other model, which I will talk about is ah, is ah, is ah, I think it is also the fellowships I talked about it is a model.

**I**: Hmn,

**R**: Yeah. Because fellowships are competence based.

**I**: OK.

**R**: Yeah. They are competence based. It is not just ordinary training, it is competence based.

**I**: Hmn,

**R**: And, you need, and when we were developing this fellowship program, we actually looked at the spiral progression of public health interventions.

**I**: Hmn

**R**: so when you look at spiral progression of public health interventions, you start from, where does the problem occur?

**I**: Hmn,

**R**: Now, when a problem occurs there, how do you detect it?

**I**: Hmn,

**R**: And then when you detect it what happens?

**I**: Hmn,

**R**: You make recommendations,

**I**: Hmn,

**R**: Evidence based recommendations, when you make evidence-based recommendations and then it is implemented, how do you monitor?

**I**: Hmn,

**R**: OK. Then how do you determine that they are cost effective? And what support systems do you give, to ensure that all these things work. That is actually, how we developed those fellowships.

**I**: Hmn,

**R**: So, when you build up your system around that and fund it, you will see how it works. You will see it.

**I**: OK. So, looking at other countries, that have maybe resilient health systems, is there any model that you thing we can adapt to our country?

**R**: Yeah, the other thing also, is ah, is for me I think, ah, reducing, I think more investments should be put in prevention that curative services.

**I**: OK.

**R**: Yeah, we need to do that. We need to deliberately, put more investments. You can even curative services even more expensive for a few people, because majority of the people will not need them.

**I**: Hmn

**R**: That is it. If you make your prevention perfect, very few people are going to climb there, then you just. You will spend little money there. Then, yeah, so, we need to do that.

**I**: OK. Why are we failing with prevention now, currently, yet we have a resilient health system. Ok, some how resilient health system.

**R**: Yeah, it is because, it is not fanciful.

**I**: [Loughs] Fanciful

**R**: Yeah. Because we think, when you build up a big building, then you go and open it then you come and report the president is cutting the ribbon, it is fanciful. Hahaah [Loughs]

**I**: Hahaha [Loughs]

**R**: Hahaha, so, why would someone take money to prevention. [laughs]

**I**: Hahaha [Loughs]

**R**: Look at that place where they are passing these bills, that building there. You saw that thing Roko is building.

**I**: The part of Parliament?

**R**: For what? For what! Why waste money?

**I**: Because they are hmn,

**R**: For what? [Hahah loughs]

**I**: Hahahaha [laughs] Oh my God! So, finally, ah, you talked about VHTs being part of the health system, yes they are part of the health system and they are at the lower level there. How best can we make them, or train them to build a more resilient health system?

**R**: You know VHTs, if you want VHTs to do their work,

**I**: Hmn,

**R**: We should stop making them mini doctors.

**I**: Hmn,

**R**: That is a mess we are making. Have you seen curriculum of VHTs?

**I**: No,

**R**: The training manuals?

**I**: Hmn,

**R**: It is like this [makes a hand gesture to mean very big] Even thicker than some of the medical books I read. Really?

**I**: Hahaha [Loughs]

**R**: That is being very stupid. Hahahaha[loughs]

**I**: Hihihi [Loughs]

**R**: You don’t need that thick manual to train a VHTs for God’s sake!

**I**: Hmn

**R**: VHTs are mobilisers at lower level.

**I**: Hmn,

**R**: Their back pack there is supposed to keep very few things for demonstrations.

**I**: Hmn,

**R**: But you find, they put there condoms, Oral contraceptives, you put antimalarials, mention them. You put there, why are you turning VHTs into mini doctors?

**I**: OK.

**R**: You don’t need that! VHTs would have been very effective if they are put there to mobilize communities at that level.

**I**: Hmn,

**R**: Tell the community that there is vaccination. They will go for vaccination. Tell the community when a child has fever, don’t say that it is malaria, please go for testing.

**I**: Hmnn

**R**: That is the only thing they need to do. But when they are called, and they say I think this child has malaria, and the VHT even goes with his/her bag. What are we doing? Hehehe [laughs]

**I**: Hahaha [Loughs]

**R**: These are mistakes that we make as a country. Yeah.

**I**: Yeah, I see, I hear you loud and clear, now I have seen the background.

**R**: Yeah. So, for me VHTs are mobilisers, it is very simple.

**I**: Hmm…

**R**: you will see the big difference. If you want to go to the community, you just equip them, give them their microphones, they mobilize the community, they whatever, you can even give them the other thing, they call it what?

**I**: Megaphones

**R**: Yeah, megaphones. That is much better for a VHT. If you give a VHT a megaphone, I will give you a tick. But you are giving them condoms, oral contraceptives, folic acid,

**I**: Self inject, Sayana. Yeah.

**R**: Eyii!, No,

**I**: OK. Thank you very much, this is wow! Anything else? Whatever you think that we have not talked about regarding health systems resilience?

**R**: I think for me, I think we, have challenges.

**I**: Hmn,

**R**: We have really tried to build up the health system.

**I**: Hmn,

**R**: And there is still hope, we have learnt a lot and I still believe that we are on the right path as long as we, utilize our funding well.

**I**: Hmn,

**R**: So, we are on the right path to build up a good health system in the country.

**I**: Ok. Thank you very much for your time.

**R**: You are welcome.

**N**: Thank you very much doctor.

**R**: You are welcome.

**I**: May be Joseph do you have any question for doctor?

**N:** No, I think doctor was very elaborative. He touched on every detail.

**ENDS.**
