# Supplementary material for: Resilience as recovery: stakeholder perspectives on Uganda's health system response to infectious disease shocks
Source: Front Health Serv. 2026 Jul 22;6:1888814. doi: 10.3389/frhs.2026.1888814 (PMC13437620; doi:10.3389/frhs.2026.1888814)
Supplement: Supplementary file 2 [file Datasheet2.zip › KII01.docx]

**TRANSCRIPTION FOR KEY INFORMANT INTERVIEW-1**

***Study title:*** *Health system resilience in the context of infectious diseases in Uganda: Exploring concepts and experiences, frameworks and tools*

**Date: 22^nd^ May, 2025.**

**Study investigator:** Denis Okethwangu

**About respondent:** The respondent was male

**KEY:**

**I: -**Interviewer

**R: -** Respondent

**VHF -** Viral hemorrhagic fever

**MoH -** Ministry of Health

**VHTs -** Village Health Teams

**Lab - L**aboratory

**UN -** United Nations

**CDC -** Center for Disease Control

**TRANSCRIPT:**

**I: Thank you so much XXXX and thank you too Professor for accepting to be part of this discussion, just to let you know that we shall be recording this conversation because we won’t be able to capture everything you are telling us.**

R: Its alright

**I: Ok, so prof could you begin by telling us a little bit about yourself ad how long you have been in the organisaion where you are and then your experience with Health System Resilience?**

R: Thank you so much XXX and XXX, am called XXXXX, am a Ugandan Medical Doctor and I completed medicine from Makerere in 1991 and I specialized in the public health and MPH from Makerere University in 1998 and I eventually did a PhD in Health Systems Research from The Royal College of Health Systems in Ireland and completed. So, my engagement with the Health System, well I trained from Mulago Hospital for my undergraduate under Makerere University, did internship in Mulago, eventually I worked in Mutolere Hospital which is located in Kisoro District, thereafter while doing my MPH, it was called Public Health without walls, Public Health Schools without walls, MPHSWOW, so we used to call it MPHS Wow. Yes so, XXXXXXX was my classmate at Masters, we joined together the MPH programme.

So during that programme I was deployed to Arua District, I worked in Arua District Health Office for one year and later I was in second year, I was deployed in Mpigi District, so we would spend 30% of our time in class and 70% of our time in the field, so I directly delt with Health System Programmes and one of the problems I had to deal with at that time in Arua was the time of Armed Conflict so there were Health System Resilience issues, because the Kony War had cut off Northern Uganda and West Nile, so we had to look for how to access the West Nile Region and continue with our work, so the partnership with the NGO international, NGO called, presently its called Doctors of Africa and this NGO they provided lecturers to the university and at the same time they also gave us some facilitation, so they would pay for air tickets for us to fly to West Nile, so we could connect West Nile by Air, and it continued like that.

Then in West Nile there was also the armed conflict of West Nile Bank Front which had cut off areas and they were not accessible to the government or could not receive all the required services because of the security situation. There after my PH I worked in Rwanda but I went to Rwanda I must say deployed to Kasese District as a District Health Officer, those days we were called DDHS but I didn’t go there because I Kasese also it was the peak of ADF War, there was so much insecurity, so I didn’t go, generally security you can see it had a lot impact on delivery of Health Services and on my acceptance and ability to move to different places within the health system. So I just went to work in Rwanda and I worked there for 4 years, it was post conflict, actually it was still ongoing, it was immediately after the genocide, I was still working in insecurity but we were working in emergency for four years from where I came back to Uganda and worked with Uganda Martyrs University and I left at the Rank of Associate Professor in 2017 and then joined an online University called the Virtual University of Uganda which has now closed and been transitioned into another organization, and in 2019, I joined Mbale University, so currently am working with Vervet University am a professor of Public Health and Associate Dean of the School of Medicine.

**I: Wow, what a wealthy experience. Prof am very happy that you have very wide experience in the health systems and most of it have been related to war insurgency, security, now we want to look at this Health Resilience in terms of Infectious of Diseases, so when we talk about Health systems Resilience what is your view, if we could take an example of the Marburg virus disease outbreak which was in Kweni, what is your view related to the resilience of health system of Uganda?**

R: Well, maybe we need to go back to the understanding and definition of resilience. Resilience is generally taken to be a system and it may not necessarily immunize itself against shocks but atleast it should be able to recover from shocks and be able to continue performing and ensuring equitable access to services for the nation. If you take it from that perspective, I think that Uganda’s health system has had important shocks, some local like Maburg, Ebola, Cholera, Acute filariasis, periodically but the system and ofcourse the global one was covid-19 where I would say we did not have strong abilities to rebound. Imagine our system, the ability for a health system to rebound, depends on a number of issues, we call them pillars of health systems, I was building blocks as presented by the DHO, we don’t have adequate staffing, we don’t have adequate, *uhm,* our health managers and leaders are not adequately. We don’t have enough trained managers and leaders in governance and leadership issues so we have had problems of corruption in the health sector at least perceptions of corruption if not real corruption, so there are a number of issues where our system wobbles when we get major shocks.

So, these emerging infectious diseases have really eat us significantly, a number of times we do take a long detecting the outbreaks like the outbreak which was in Kiboga Mubende area, by the time you realize what is happening, quite a number of people have already started dying, the disease has spread to a number of districts in a country. So, our system at the detection stage of sort of immunizing itself against these shocks is still lacking.

When it comes to rebounding, I think we have got a very strong ability as a system to rebound because we have seen that generally although we have been hit hard, but sort of the system picks up and starts moving again, there is a complex system, I do believe that Uganda’s health system is quite complex and is able to repair itself and get going again. There are a number of factors that have contributed to this, the training of health workers has improved over the years, we don’t have a critical ………….as yet but the training of health workers has improved, in particular I look at the post graduate training programmes, like there is our programme which we went through with XXXXX, it was called a Field Epidemiology programme, it was later succeeded by the AFENET programme actually am a direct defender of that Field Epidemiology Programme and it has trained a number of people who have eventually become global experts. When you see people like Dr. Monica Musenero, she went through the programme, if you see like, there is one of our classmates who is called XXXXX, she works now with WHO Geneva, she was at the programme and eventually she was involved in the first Ebola outbreak in Uganda, so many people have been trained and they have become sources of other countries which experience similar programmes.

So you ca see we are on the right track, we are not yet there in building the human resource capacity, I can say we are still weak but the rebounding is stronger. On the other pillars I think we still have some issues, our system has been largely on external support in terms of financial muscle, so this does influence our ability, God forbid now if we would get a major attack we would have a lot of problems because most of our funding for health action has been coming from Donors especially the US and they were managing it themselves through a side channel but its nolonger available, so if we are to get an attack at a time like this when US has withdrawn significant support for programmes funded through USAID and even scaled down CDC funding, I really worry as to how we would cope. First of all the National Budget Allocation for Health Care is small and has been dwindling over the years and the proportion to the to public health has been going down, so where would we get the resources to manage a major crisis? Would the IPs agree to open their passes again, would other donors step in at the right speed to be able to ensure access to adequate resources to manage a major crisis, that is a big question, I doubt because I have seen over the years, engaging with these development partners in a fora, they are good hearted but they don’t open their passes.

So, I remember when we used to attend the joint review missions at Munyonyo with quite a number of development partners, they pledge many things but when you come to a review the following year, they ay its not honoured, quite a big chuck of money that was pledged they say its not honoured. So there was an initial motivation when we had the Charismatic leadership at WHO country office, there used to be a WHO representative who was called Atib Jay, he had a way of mobilizing development partners and they were able to release whatever they had pledged because also Donors would come on board and they strengthened the Health Policy Advisory Committee at the National level and you could see they were speaking the same language with the Top Management of the Ministry. But over the years, I do not see the same convincingness at that level as wheat we used to experience at the beginning of 2000s, so like from 2000 to 2010, you can say those were the Golden years of the health sector in Uganda, but since then there has been a couple of challenges and these have affected our ability to respond at short notice to such kind of situation. So that’s what I would say.

**I: Alright, thank you very much, I think you have answered on my next question which goes to define the Health System Resilience, you have already answered that, you have also touched on some of the factors that are contributing to the Uganda Health System or being able to withstand or recover from the major distractions, but you talked about trainings, when we look at leadership, what is your comment on how it contributes to the Health System’s resilience? What about equipment, what about any other building block in that line?**

R: Yeah, first of all from leadership perspective, it could be that we deal with these emerging infectious diseases and re embark on infectious diseases, like TB for example, we have heard the development of multi Drug Resistant TB, we have risks of drug resistant HIV but we need to keep our fingers on the path of the health system and that is a question of leadership. Do our leaders in the health sector keep our fingers or the fingers of leadership on the path as far as infectious diseases are concerned? How strong is our IDSR System (Infectious Disease Surveillance and Response system). It does not cover, when you get a situation like the recent Ebola outbreak, like Marburg outbreak, you realise that the disease can take a couple of weeks before it is detected that means that our systems are not very strong as far as that aspect is concerned. And then, leadership from strong leadership, that area from the top, who heads the HMIS for example? Is he or she the resource centre? There was a time when we had XXXXXX he was the leader of resource centre at the Ministry of Health and he was an Integral part of the top management and people would listen to him and so he could get information, claim and pass it on.

We have improved in terms of technology, those days we were getting reports using paper based system but you get problems within one week but with the online HMIS, the DHIS 2, you would get problems, the problem takes 3 weeks, 6 weeks or it would take like the Ebola outbreak where the Tanzanian Doctor died. I thought we had improved in technology yet it would take longer in detection of an outbreak, I think we need strong leadership at the head of the resource centre, which receives the DHIS so that they can be able to screen the information coming in. we also need to influence, so the leadership of the disease surveillance mechanism, there is I think a department now, National Disease Surveillance, the likes of XXXXXX, I don’t remember whether that now is headed by XXXXXX but they need to also engage with the training institutions. How do we increase the, in clinical medicine there is what we call an Index of Suspicion, it is a situation where as health workers, clinicians, you are always suspicious that this could be the problem, so once you get high index of suspicions you are more likely to investigate looking for what you are suspecting.

Now, in Disease Surveillance Mechanism we need to develop a high Index of Suspicion for these hemorrhagic fevers, viral hemorrhagic fevers, malaria outbreaks, and insurgence of diseases like TB. So how do we increase our index of suspicion? I think its through training, I don’t know whether you have read some literature about first corona outbreak in Uganda which was detected by the late Dr. Mathew Lukwiya, he was the Medical Superintendent of Lacho Hospital and he was called as a student of Master of Public Health Makerere at the time when the outbreak occurred, so they had just gone through a crew of infectious diseases and he went back for a holiday that’s when he detected the disease at the hospital unfortunately he had already got in tough with the disease and it took him and killed many other people but atleast the index of suspicion because of the training he had had. So the leadership of the disease surveillance mechanisms in the country needs to engage with training issues to our health workers both at clinical preservice level and the nursing level. What is their index of suspicion for these infectious diseases? If they were interested can we for example introduce a course unit on infectious diseases for undergraduate medical students, or undergraduate nurses, preservice nurses at certificate level, diploma level, bachelors level, masters level, all of them can be introduced to a course unit called infectious diseases, how do they present, these hemorrhagic fevers how do you detect them and these other diseases of epidemic potential, how can we detect them? So if everybody is trained through all those I thin we would have a critical mass of heal workers with a high index of suspicion because these are the front line workers, they are the ones who deal directly with patients, they live out there in the community, if they are not trained to recognize and report promptly, then the system remain vulnerable so the speed of detection becomes weak. So we need to strengthen our ability to detect and make sure its stronger. The ability to detect needs to be acted upon so that we can detect within one week, within three days we should be able to announce the outbreak of an infectious disease within the population. Yeah, that’s what I can say.

**I: Thank you very much Prof, what about the community engagement, how have they contributed to the ability of the Ugandan System both at national and sub national level do we stand to recover from the major destructions of outbreaks, the community engagement or community level, what has been their contribution?**

R: Well I would say that most of my lesson could be from covid where we saw a community engagement, it entails leadership to participate in community engagement at the political level. So it came with scientists fortunately our health minister is a medical scientists so she could set the structures or the ministry set up the structures to advise the political leadership of the country specifically the president who mobilized the public to get involved. So in that you could see that most of the population that responded to the pandemic was through the political leadership and that was an excellent collaboration because believe it or not, the public trusts the political leadership on health matters more than they trust the technical health leaders. So if the health problem is presented to the community by a political leader, the people are more likely to listen and respond than when it is actually coming from the health positions, this is a paradox situation but it seems to be the case in many other countries not just Uganda. So we need to engage, at national level, the engagement has been good, at local level, the health workers still take matters in their own hands, they still run the talk shows.

If you go to other countries, the health talk shows are run by health workers, the district health assistants and DHO, they are the ones who run the talk shows yet preliminary evidence shows that people listen more to the political leaders than the technical personnel, so we need to make sure that at lower levels the technical people hardly struggle over the political leaders who are listened to more than the technical people, they can provide the technical information, provide the support but really the community mobilization and engagement is more effective if it is handled by the political structures.

The other issues depending on the country you are, is the traditional political leaders, lets say the social cultural structures, the religious leaders, those can also be empowered by the technical health personnel to make sure that they are actually mobilizing people to be on alert of these disease epidemics. So my view is that, we the technical people we need to reach the community, yes, but through the trusted structures that are trusted by the community and these are largely political and to some extent, the social cultural structures and the religious structures.

**I: Ok, thank you very much Prof, for that, uhmm, just to take you back to the leadership, you talked about keeping the fingers on the path of the system, what did you mean exactly if you could simplify this.**

R: Real what I mean is our ability to detect the occurrence of these diseases or pandemic potential should be very high, I mean ..as I mentioned I have lived in Uganda, I have lived in Rwanda, I have worked in Malawi and lived in Europe, for example, in case of heart attack in Uganda, the people who can handle heart attack just where I was living in Ireland, very few people die of heart attack, they have got an indicator called “call to needle time” when a person gets a heart attack either that person gets a phone or somebody in the house makes a phone call, they call the ambulance system, the ambulance system arrives and they inject the person with an appropriate drug within a maximum of 23 min, that is when I was in Ireland that was the maximum they were quoting, so the phone to receive a needle in his or her blood was 23 min. now if you compare to Uganda, “the call to needle time” for a heart attack it would be a couple of days, now, within through a similar indicator for our diseases or epidemic infectious diseases, how long do we have to relate as a health system that if patient develops a disease, how long should it take before the head quarters is notified and before action is taken. So the similar kind of indicator similar to a call to needle time as used in Europe especially in European Union I remember at that time it was 7 minute but the average was 23 minutes, they were not yet there but they were moving towards there, I should check how long they have now improved. So, there are quite evidence to ensure quick detection, quick reporting, quick response. So that’s what I meant by keeping our finger on the pals detecting the occurrence very quickly.

We have got the thresholds, they are set by the British, we know for different diseases, if one appears, some diseases you must report internationally, immediately, some other diseases you must report nationally immediately. Some others you wait until maybe a number of cases has reached a certain level that’s when you report. So we need to update these thresholds as I said we need to increase the index of suspicion for our case definition, must we first confirm before action is taken or we can start acting when we are still dealing with probable unstained cases, so all these call in order to increase our detection speed so that the system can be more responsive. So the Achilles heel of our system, the resilience of our system to me is at detection stage, the response is fairly still poor but it is stronger than the detection speed between diagnosis and action. Once the diagnosis is confirmed, then its generally appropriate but that gap is where we still have a problem.

**I: Ok, thank you very much Prof, now I would like you to maybe give us more interventions or actions that have been undertaken or that have contributed to the resilience of the Ugandan Health System, besides the training that you talked about, you said health workers have been trained, are there any other actions that have been taken or that you feel have contributed to the resilience of our Ugandan Health System?**

R: Well, I would say ofcourse there system changes reforms that took place years ago like centralization did contribute even that now with centralization local leaders get involved depending on how well the action is taken and also how the system is arranged. You recall that following the repeated Ebola outbreaks, I think the lessons learnt from the repeated Ebola outbreaks was that the mobilization is better done through political structures, so wherever we get a rapture, the health system reports to the political structures and the political structures take over, so the approach of the political structures is through directives, once the message is passed, then the RDC who is the head of security at the district, they act and mobilise all the local resources, UPDF, police and other local structures, the security structures come into place, so that has helped a lot and when there was an outbreak the west Africa Ebola outbreak, the first they deployed General Okello because from Uganda’s lessons we have learnt that situations like those you respond through directives, so the first person in human resources with deployment of medical resources and other resources, that’s how they were able to bring this disease under control. So definitely, we use an orthodox approach managing an infectious disease outbreak but it works so I can say we have adopted a pragmatic approach, what works is what we rely on, that is through directives of through political and military structures.

**I: Ok, what about preparedness, could you comment on how that could contribute to the resilience of the health system, what about infrastructure, you have talked about exposure when you talked about the repeated Ebola outbreaks but I would like to hear your comments on preparedness and then infrastructure and how it has contributed to the resilience of health system in Uganda?**

R: Yeah, now, on preparedness we are very bad because we don’t have the structures in place but our preparedness I would say has improved over the recent years and again from covid-19, we now have structures like actually I have heard of something like “the incident commander”, there is a doctor I have forgotten his name, but he is a Military Colonel based in the ministry of Health on standby, ready to activate this dormant mechanism of the directives…he is on standby, this is a new thing, whereas it was established during covid actually it acted during the Ebola outbreak that came after covid outbreak and it helped to respond quickly I think it was also activated during the Marburg outbreak in Kween, so it is now going to be a permanent structure of the resilience mechanism of the whole country, so we are innovating the response mechanisms that eventually maybe other countries will learn from and pick and make them even better. That’s what actually happens, Ugandans are usually known to be very innovative and then our innovations are improved upon by other countries, so we need improvement in our innovative area.

As of infrastructure we are still not doing very well because you recall for example during covid, our health system didn’t have adequate surge capacity, the surge capacity of our health system was over whelmed because of the number of cases, I recall we could not get hospital beds in all the government and private hospitals, until the decision was taken that lets manage covid cases at home, that was an unusual case but what is the surge capacity under non pandemic conditions, do we have the ability to expand our hospital admission capacity for these cases. We always see whenever there is an outbreak of cholera or typhoid in Kampala, there is a place in Mulago which is identified as during the covid-19 outbreak, we had to convert the mental units in the hospital in the regional referral hospitals as the covid hit peaks, can we arrange and have appropriate units for managing infectious cases in all these let’s say at regional level but eventually scale them down to regional hospitals and with time to the lower levels. So to me, we don’t have adequate infrastructure but that’s something we can look at. During covid, we just took over the mental health units but what about having infectious institute units, we have other departments what about having infectious disease treatment units in the hospital, that’s the area we can expand and have a new department the structure in the hospitals, so I would advocate for that line for future department, development of infectious disease treatment units, talk of isolation units, but you know that the hospital has 1 to 2 beds in the hospital and call it isolation unit which is not even appropriately isolated from the rest of the wards, can we have the structure, we can team with the structure division of the ministry of health, can they design for us a plan that can be rolled out to all hospitals something appropriate for each level for managing these infections.

**I: Ok, thank you very much, so I would like you to paint for us a picture of what a resilient heath system looks like most especially to infectious diseases, what are those characteristics that make a resilient health system to infectious diseases, paint for us that picture and share characteristics, and then after that you tell us some of the unique attributes of the Ugandan health system are that work towards making the system more resilient, one, tell us the characteristics of a resilient health system generally and then two, tell us some of the attributes of the Ugandan health system that work towards making the health system more resilient.**

R: Hmm, first, I think in terms of characteristics, yes the system should be able to immunize itself first and foremost, it should make sure that we know these things are going to come and prepare, see when they are likely to come, detect them when they come as soon as possible then be able to respond to them appropriately. Now, on detecting when they are likely to come I think lets strengthen our training and response levels, be able to …actually disease modeling, our classes including mine will not teach disease modeling, I have a colleague at the University who studied from south Africa, he studied mathematics and specialist graduates of masters and PhDs even I think in Makerere they don’t practice this model, then modeling, people are able to predict, but this gentleman studied it from South Africa, can we bring it here in Uganda, at undergraduate, at Masters level at PhD level for those who are doing epidemiology such that people get the skills and be able to predict when he epidemic is likely to come. We collaborate with Ministry of Health we should have a unit within this surveillance mechanism in the ministry of health which predicts their focus on prediction, when is it likely to occur, what are the features at individual, human, the likelihood this infectious disease have.

For example, when covid started, I had just joined Kabale University and it started in China, we set up a unit to analyse the possibility of this disease coming to Uganda, to Kabale in particular, we created that force, actually I was requested by the vice chancellor to study what is happening in China and inform the University Management on what is the likelihood of this outbreak reaching Kabale. So I ofcourse had heard about covid, I had read about Sas but not Sas cough, so I had to go back to read the literature, I had to watch news and see what is happening, eventually I developed a profile of the disease, I gave a presentation to the management and to my colleagues at the school of medicine and eventually we agreed that this disease is likely to come to Uganda and is likely to reach Kabale within a period of 45 days, the diease actually reached Kabale wthin a period of 60 days, so our model was not very strong, it was not mathematical, it was not based on epidemiological analysis, we were just seeing the travel patterns of people and see how long it would take to reach Kabale district. So, we need to build that capacity of modeling the projections of when is it likely, what is the likelihood that it is likely to come, so we build our preparedness along the way and once the model is firm then we put in place mechanisms, as we have had preparedness mechanism for malaria.

You might know that malaria was ……in the Kabale Area because Kabale is a hilly area, the place was cold and mosquito breeding was high, atleast it was in 1995 when we experienced climate change, people started getting a lot of cases of malaria and ofcourse the people who had very poor immunity, their mortality rate was very high but the ministry responded by predicting death outbreak and ensuring the drug supply of malaria was adequate in Kabale for a long time in Kigezi region in general, so they would make sure that season where they expected a large number of cases, they made sure the supplies of anti malarials were actually very good, so they had restocks and the supply mechanisms were all activated throughout those seasons and they were able to reduce mortality and this has continued up to now. So we need to do the same for the different diseases that we are experiencing, we know some which have been recurrent, Ebola, typhoid, Marburg, Cholera, they have been recurrent, polio, so we can establish those mechanisms and make sure that the ministry responds very quickly and reduce both morbidity and mortality.

**I: So, the part of unique attributes, did you talk about them?**

R: So, the unique attributes of Uganda’s health system that have improved our resilience is, I should say they are an orthodox approach, some people will say they may have a charitable precedence about the approach. First of all, you have a leadership that has been in power for a very long time, it has its weaknesses, it has its strong points, if we leave the weaknesses aside, the strength of having a system that has ben in leadership for along tie, it should include the fact that the political leaders understand the country very well and they know which approach works and which one doesn’t work, for example they should know that if you beat up a few people and put the pictures on TV, when it comes to mobilizing, to isolating people to enforce the law, people get scared when they see kiboko, when they hear somebody has been shot because he didn’t respond to…people will stay home, so those lessons are learnt from having been in power for a log time. So they know that this one works and that knowledge should be put to use, so its unique for Uganda especially in an era where many people are, *uhm,* which involves change of periodic reviews, our failure to change leadership results into those benefits for us as citizens because those long serving leaders know how to manage our society.

Then another uniqueness is the trained technical and political, that is one unique are in many other countries, the technical people want to take the things within their hands, the political wing doesn’t have a say, this is so because the government doesn’t have the capacity to allow the health sector handle matters on their own but when you look at government, there are usually enough resources to handle the disease outbreak, so if you access the position from the technical health sector from the political level, this is across the sectors so you get the resources coming not just health sector wise resources because its not the only sector, so that’s another unique feature that one can say Uganda has had that improves our resilience.

Then I should say the general nature of Ugandans, this is something we cannot discount, Ugandans are generally cooperative, when they hear of people dying in one phase and you are telling them please do this so that you don’t die like those others, they generally respond, that is within our social family as Ugandans, we listen to correction and we respond to it, so that gives us credit. Once a population is sensitive and responsive to instructions, I think that also calls for resilience.

**I: Thank you very much, earlier along you had talked about the role of the community towards the resilience of the health system, I don’t know if you have anything else to add on that, if you don’t have, I would like you to tell us some of the interventions to the challenges that you mentioned. You mentioned over dependency on donor fund, poor detention, poor infrastructure not using the politicians, as some of the challenges that affect the health system, but with all that and you keep saying that the health system is more resilient now, so what are some of the solutions that have been put in place to work on the challenges that you have talked about for us who have the resilient system?**

R: On community I think we need to continue educating the community but as I said, let the community ……….as I said we have had a constant problem of over reliance on funding, I think Trump has opened our eyes to start life more and more on our own resources, I know for example the Ministry of Health, when Trump cut off the funding in USAID, they opted for integration of whatever we are doing into a more nationalized system rather than vertical system, yes you can integrate the services but if you integrate the model of delivery, that was very heavily funded by donors into the poorly funded system, you will soon begin to deliver the services. So, I think we need to redesign the model of delivery of infectious disease control. So we should not absorb the models that were funded by USAID and integrate them into the existing without modifying them and customizing them, the ability of …..as we take the cone, I agree that integration is the best approach but it should be accompanied with modification or customization. One needs to analyse what mechanisms do we have, how are they funded, how can we continue with the same mechanisms but within our means.

The other question is, do we have the ability to increase the funding for these activities? I think we could have some if we look at the national budget as a resource envelope, we need to prioritize these occurrences more and add more resources from the National offers not on standard funded resources, so we need to reactivate more of our own resources, especially on infectious diseases and pandemics and the case of HIV has been very pathetic, how can we as a country hand over HIV control to donors, global fund, USAID and other support mechanisms, we cannot afford that treatment because we have got …1.4 billion being spent on ARVs in the country but if you put all of them at the mercy of foreign countries and yet you would not be able to sustain your own population. So we need to have, HIV yes its an infectious disease but not highly infectious though in the sense that the mode of spread is not a public health, its not a top model in the top mode because it involves sex transmission and exchange of blood and fluids other than other diseases which spread through physical contact, air, we have to make sure they are all funded through our own mechanisms.

So, I think we still have the capacity if we look at certain sectors on which we spend money, the public administration through the budget lines of government, public administration still consumes a very significant chuck, I think we could see if there are more resources for health care and this could be channeled into absorbing these approaches that were funded by donors, can we raise new sources I am not very good at that area, URA, can innovate, we can also have for example disease cards. In Kenya they have ear marked funding specifically for health conditions, health problems, I think we can also do the same in Uganda. And if we can ear marked funding for infectious disease control and raise them from different sources, we have ear marked tax, I think it can work for a couple of years to raise money, the units …………..people can tolerate that……….(1:20 not clear).channeled directly to infectious diseases units. So that’s one way. So, we can mobilize internal resources but also ear locate the existing resources in other areas and channel them to infectious diseases control.

**I: Ok, thank you very much on that, we are going to the part of the recommendations, what do you think are some of the strategies or initiatives that you would propose to strengthen our health system and improve its resilience to infectious diseases?**

R: Really, we have been touching most of these, for me the story begins with detection of infectious diseases, so how do we improve detection, it’s through improving the sharpness of our health workers, the front-line workers, so that they think of possibility of these infectious diseases at community level and health facility level, so we need to strengthen the curriculum, nowadays, the government is talking about competence based training in primary schools, secondary schools and its coming to senior level, in our health institutions can we also improve the competence of our health workers in detecting new and emerging and re-emerging infectious diseases. So competence training in these infections that is our own area to improve on our resilience.

Then once this is detected, information should flow, it should be very quick and the responses should be very quick and the rest training would be training of managers in the health system, how can we improve decision making as far as infectious diseases are concerned, its should be very quick, so the person in charge of HMIS should not wait for the Monday meeting to report and for the actions to take place, I think we have a weekly reporting mechanism maybe we need to shorten the reporting period for these infectious diseases such that we don’t have to wait until Monday 10:00am, if the case is detected, the report should be sent immediately, so we may have to make concessions to allow prompts for infectious diseases. So if you find 10 infectious diseases, you should report immediately without waiting for a Monday weekly report, I think that would also solve also go a long way in improving our quick infections.

And then the person at the help of surveillance system should have a hot line, the minister so that these things are reported immediately to the minister then eventually since we work through a political system, the president should be alerted immediately, not to have to wait. I know currently the president says he gave the minister of health a hotline to report the disease outbreak but the minister herself receives reports after 1 week, so there is still that delay of that weekly report, they extract information from the weekly surveillance report and its that information that they can meet the president very quickly but it already has a delay of one week, so we should make sure that we use the system of hotlines.

Then I don’t know how the funding was at the ministry of health level, I think that we have ….i don’t know the legal provision, whether the minister of health is allowed an emergency budget (missing words…1:25) cabinet meetings and is meant to declare the disease outbreak or things like that, I think we need to ease the speed with which funds can be accessed or used for response, checks and balances to avoid misuse and …….so that (un audible..).

We need to strengthen the disease surveillance and response unit at the ministry level because I know at one point ministry had created a standby team of affiliate graduates willing to be deployed immediately on the ground whenever there was susception of any outbreak and those days we were targeting the Ebola mainly from the DRC but I think the standby teams, I think affiliate I see they have an office in Lugogo along Lugogo bypass but I think we need to have standby teams of individuals who are willing to help whenever they hear, they can be disease focused for example we start with a team of Marburg, a team of Ebola, A team of Cholera something like that, so we need to develop that kind of mechanisms of those standby teams. Otherwise, I think we have tackled most of my other recommendations along the discussion.

**I: Ok, maybe I missed that out, on the VHTs how do you think we can support them, or how can they better empowered to develop a more resilient health system? If you already mentioned them maybe you could just ….**

R; I actually didn’t mention VHTs specifically, its an important structure but the problem the kind of diseases we are talking about, the infectious diseases are usually such a threat that you even get scared whether VHTs should be involved because that may even increase mortality, but there is a training VHTs, CHEWs we did offer them that they are also alerted that there are some conditions where we report that immediately and where even a VHT member should not even get in physical contact with a patient, so it takes..i have not seen a survey showing among the people who die of these lets say hemorrhagic fevers, how many were VHTs? We have seen reports on qualified health workers, if the VHTs are the first respondents at community level, how many got into contact with these people of infectious diseases and how many of them actually die, it could be a research question to find out, of the people of who died of these diseases, how many were VHTs we could be losing a lot of VHTs if we encourage them to respond quickly to whenever there are health problems in the community and we have not trained them about some of these infectious diseases and then they respond and get infected. So we need to improve the infectious diseases in the training of our VHTs. It’s also comprehensive.

**I: Ok, thank you very much, we are on the last question now, you talked about Kenya having a successful model where they ear mark some funds for health, am just wondering if there are any successful models that you think would suit our context in relation to our health system resilient that we could adapt or modify?**

R: I know for sure Kenya has ear marked health funds, they collect taxes on roads, they have a road toll system where they invest some money and that money although you pay it on the road its specifically for health, they do it periodically, its not a permanent thing, they do it when they want to address a particular issue, so we could adopt the same thing, but in Uganda the only road tolls we have are only on express ways but if we were to have a reliable mechanism where the funds are not being misappropriated we could institute even a road toll. We have I think now Uganda has a very high rate of importing vehicles, so if a road toll could work on major roads, or alternatively, the best example would be to turn the taxes into a daily expenditures so that its not ear marked, Uganda has been avoiding earmarked taxes, it has been generating money through VAT, PAYE, and a number of other taxes but I think if the government educates he public about the need for specific thing and they actually use the resources or that particular purpose I think that can be done.

Ugandans for a long time have specifically known the government intention and government ability, so if you propose an ear marked tax it will obviously face resistance but if they can demonstrate that whatever little they have collected has actually been used for the purpose its meant for, I think the public can improve on some funds for a year or two and can have these items funded for such a purpose if for example they were saying lets make a tax even if it’s a small amount lets say 2000/- for every vehicle that passes along Jinja road and the money we are collecting are for building an infectious disease unit at Mulago hospital or any other hospital, Jinja, Kawolo, whatever it is and actually they put it there and keep the public updated, we have raised this amount, we have contracted a contractor to build this and this is a facility and people actually see it, I think the public willingness to pay for such a cause would be improved. What the public fears is to be put into the general pull of government resources, the consolidated funds of government then hence people don’t trust them very well, so the willingness is not there.

**I: Ok, thank you very much Prof, for your time, I don’t know if my colleague Joseph to ask, Joseph is you have a question one or two you could ask Prof.**

**I2: I think I don’t have any question for Prof, he has been very elaborative and he has given us a big picture of all the perspectives that we are looking at.**

**I: Alright, thank you Prof, for your time, I don’t know whether you think there is something we have not talked about, you could say it, otherwise that would mark the end of our interview.**

R: Not really, I think at the standing of resilience I think we are on the same page so I don’t have to add, I thank you for the opportunity to discuss with you this topic, I have been kin to understand what resilient is over the years, now that Denis is focusing on it, I wish him all the best in his research and I look forward to the publications from his research.

**I: Definitely they will come out. So, Prof, the secretariat will reach out to you with some little airtime, as we wait for our consent form.**

R: Oh, I didn’t expect that. Alright I will do the consent.

**I: Ok, then, thank you and have a good evening.**
