# Supplementary material for: Resilience as recovery: stakeholder perspectives on Uganda's health system response to infectious disease shocks
Source: Front Health Serv. 2026 Jul 22;6:1888814. doi: 10.3389/frhs.2026.1888814 (PMC13437620; doi:10.3389/frhs.2026.1888814)
Supplement: Supplementary file 2 [file Datasheet2.zip › KII02.docx]

**TRANSCRIPT OF INTERVIEW WITH XXXXX**

**Health System Resilience in Uganda**

**Interviewer:** Good afternoon Prof. It's a pleasure to speak with you today. Thank you for accepting to have this interview with me. I understand you have many titles, (laughs) and we are honored to have you here. I won't take too much of your time, as I know you're a busy person. As you've read in the consent form, this study is titled "Health Systems Resilience in Uganda," and we are exploring experiences, enhancing capacities, frameworks, and tools. From your extensive experience and knowledge, how do you define "health system resilience?’’

**Respondent:** Hmm…. generally, when we discuss resilience, we're referring to the health system's ability to absorb shocks and adapt itself, sometimes even transforming entirely, so that it can continue providing its usual services while also offering any additional necessary services.

**Interviewer:** Hmmm….. given that definition, what are the key attributes that contribute to a resilient health system?

**Respondent:** For a resilient health system, you need to consider various aspects, often examined through the building blocks of the health system itself, as well as what is required for adaptation. When we talk about resilience, we're focusing on the health system's capacity to adapt. If you encounter a challenge, how can you modify your approach to suit the new situation? Sometimes, you might just need to slightly adjust the system's structure. Other times, a more significant reform is necessary, or even a complete transformation of the entire system. So, if we consider the components of the health system, the key elements must be structured to allow for adaptation. There should be a degree of flexibility. Across all the health system building blocks, consider the financing aspect. If you experience a shock that necessitates reallocating resources—like during the COVID-19 pandemic, when resources suddenly needed to be directed towards disaster management—are your systems flexible enough to allow this? Can you draw resources from other areas? In terms of adaptability, the bureaucracy within the health system should have mechanisms that permit such adjustments.

Sometimes, a complete transformation is required, demanding a change in the entire structure of your health system. Crucially, the health system needs to be a learning health system. It must be able to recognize new challenges. For example, with Non-Communicable Diseases (NCDs), sometimes referred to as a silent epidemic, we historically thought they primarily affected the wealthy. However, they've begun to impact those who are not affluent. The system needs to realize this and understand that we must change our approach to prevention and restructure how human resources are configured to respond to the high incidence of NCDs. So, the system must be able to accommodate such reforms. These reforms may be needed in various parts of the health system. Sometimes, you find that reforms are necessary in human resources, where the specialists you currently have can no longer handle the issues you're facing, or you might need more specialists. For instance, if you need more people working in prevention, the system must adapt to that.

Therefore, the health system needs to be a learning system that can study what's happening and implement appropriate reforms. In terms of infrastructure and service delivery models, we need structures that can accommodate changes without excessive difficulty if modifications become necessary.

*……… (Interruption as the respondent attends to a person who had come in her office) ….*

**Interviewer:** Welcome back from that short break. You've provided a lot of valuable information, especially regarding the key attributes of health system resilience. You also mentioned COVID-19 and how a health system can be flexible and adaptable. Using that same example, how would you best describe our Ugandan health system? Is it adaptable? Is it flexible or?

**Respondent:** Perhaps before we delve into that, there are a few important points I haven't yet addressed. During COVID-19, we had a **contingency fund** in the country for financing emergencies or disasters. For a health system to be resilient, it needs to **plan ahead and recognize that these types of shocks may occur**, requiring reserves it can draw upon. As a country, it's beneficial that we possess such funds.

Additionally, in a learning organization, the human resources within the system must also have the **capacity to effectively respond to challenges or problems when they arise.** Sometimes, human resources in management positions, even those expected to deliver services, aren't trained to react effectively when a problem occurs. Yet, in service delivery, when a problem emerges, you have to react and respond regardless of your position, often necessitating changes in how things are done. So, at all levels of the health system, human resources need this capacity.

Looking at the Ugandan health system, I believe there was a ranking done by WHO, and **we ranked highly in terms of resilience.** I would say our health system is highly resilient. When COVID-19 hit, we observed how the health system adapted to the new situation we faced. In facilities that used to experience significant crowding, we very quickly saw some facilities begin to use tents, introduce appointments, and offer services like lab tests in homes, among other changes. I believe this demonstrated the health system's capacity to adapt to different situations, and our human resources have proven their ability to respond to such demands.

However, where I think we don't perform exceptionally well is in **reforms**. Sometimes, simply adapting is not enough; you need to transform. As a health system, we don't excel at transformation. For instance, when we discuss prevention, health workers are largely focused on providing curative care. But for us to effectively prevent Non-Communicable Diseases (NCDs), we need to restructure and have more workers providing preventive services. This requires a fundamental reform in how health workers are organized in the country. We are taking steps regarding community health workers, but we're not moving very fast.

Furthermore, regarding the availability of funds, I mentioned the contingency fund. For example, the Public Finance Management (PFM) Act requires that when money is collected, you send it back to the central government, which then sends it back to you. During COVID-19, when we collected funds, the PFM required us to send that money to the Ministry of Finance, which would then send it back. Yet, in a situation demanding an immediate response, you don't have that kind of time to say, "We've collected this money, what should we do?" So, we need **reforms in the PFM that allow for immediate implementation.** Reforms usually take a very long time, so we need to implement reforms to enable us to respond to these shocks in real-time. We have a weakness there.

Again, during COVID-19, we saw various stakeholders come together to respond. Similarly, when we had the HIV epidemic in the 1990s, we saw different stakeholders coming together to respond. So, once we have a critical understanding of how to deal with a situation, we are able to coordinate different stakeholders.

However, when it comes to **providing resources, especially when facing a shock that requires additional resources** to handle the situation, that's an area where we don't perform as well. At times, our response isn't adequately resourced. When we talk about infectious diseases, you have to protect your health workers, and you need to increase drugs and diagnostics. At times, our resources are so constrained that funds are not readily available at the central level, and resources at the district level become conditional. Districts don't control a lot of resources. So, even as a District Health Officer, you might face a cholera outbreak but lack the immediate funds to allocate. Many of those resources are conditional and controlled by the center, which restricts the extent to which different managers at various levels can respond and provide the required resources in real-time. This is even reflected in infrastructure. It really revolves around the ability to provide adequate resources in a timely manner. We saw countries like China rapidly construct hospitals. We couldn't do that. We did what we could, like converting a stadium into a treatment center. It was the best we could do. So, it's a 50/50 situation, being resource-constrained. But again, when it came to providing things like sanitizers and gloves, we saw some local manufacturers increase production, indicating a capacity to respond to some of those needs.

**Interviewer:** You've discussed the health system's adaptability and flexibility. Regarding the recovery from disruptions at National and Sub-National levels, do you believe the recovery rate was consistent across both?

**Respondent:** You mean recovering from disruptions?

**Interviewer:** Yes.

**Respondent:** Of course, it depends on the specific disruption we're discussing. I believe there were attempts, but some responses were not as fast as needed. Again, using the COVID-19 example, some of the measures we implemented, like transport restrictions, greatly impacted people relying on public transport, especially those on medication for chronic diseases or HIV patients needing refills. These individuals faced significant challenges accessing facilities. We observed an increase, particularly in teenage pregnancies, due to the lockdown and transport difficulties. Some responses tried to adapt once it was realized that certain people couldn't travel; they attempted to respond accordingly. For instance, regarding teenage pregnancies, they tried to engage schools to allow pregnant girls to continue their education. When it came to distributing food to those who were unable to work, it's fair to say the system attempted to respond to the outcomes of COVID-19, though perhaps not as swiftly as ideal. We did see attempts. Also, in the subsequent financial year, the government increased the budget, indicating an attempt to respond, but that was more of a financial shock to us.

However, other types of responses we needed to implement, such as generally cutting down expenditures across the system, didn't really happen. We see the government stating they will allocate more money, but they often don't fully align with the need when we face a financial shock. So, the responses are often **half-hearted.**

Perhaps within the health sector itself, Ministry of Health workers usually respond faster than those outside the sector, such as political leaders or those providing financing. Their responses are typically slower than those of individuals within the system who provide services and oversight.

**Interviewer:** Ok, you mentioned building blocks, which was my next question, and you've thoroughly covered financing and human resources. Perhaps you could now discuss **health information systems** and **medical products, vaccines, and technologies**. Could you elaborate on their strengths and weaknesses within the health system's building blocks?

**Respondent:** For **health information systems**, I think the main challenge we used to have was that it was primarily a **paper-based system.** When certain shocks occur, the system should be able to immediately provide you with the information you need. For instance, during a cholera epidemic, you need to know how many people have been admitted, how many have died, and so on. So, one of our weaknesses was having a predominantly paper-based system.

However, we are currently in the process of **digitizing the system.** We now have **electronic medical records within facilities,** and we also utilize m-T**rac,** which allows health information to be provided to health teams and emergency centers. For example, during outbreaks, they can report the number of suspects, confirmed cases, deaths, etc. Especially concerning outbreaks, we're doing quite well because we're able to share that information efficiently. But when it comes to the overall **integration of the health information system, different parts are not yet well-integrated.** Our health system isn't well-integrated when it comes to linking human resources with the District Health Information System (DHIS) or connecting grants to the management system. These disparate parts aren't integrated, which creates challenges during outbreaks. For instance, if there's a cholera outbreak and particular drugs and fluids are being used excessively, it would be incredibly beneficial to be able to immediately inform the district level that a facility has run out of supplies.

Unfortunately, significant progress hasn't been made toward this kind of integration, though there's certainly interest and discussion about moving in that direction. As a result, we have "verticalized" systems—for example, one specifically for surveillance—that aren't well-integrated or interoperable. This means we lack comprehensive information, such as knowing exactly where two Doctors are located in case of a specific need. This is a weakness.

Furthermore, in terms of analytics, our capacity is improving but still somewhat constrained. We sometimes need to analyze incoming system data to take necessary actions. Even within our community engagement systems and the broader health system, we lack a robust community health information system that could help us capture the type of granular information we need. For example, if you need to know which households affected by cholera have latrines, or if a specific group is likely to be affected, our current information systems aren't sufficient to provide that. During COVID-19, we needed to identify individuals with certain pre-existing conditions who were more susceptible, but we didn't have that individual-level demographic information in our systems. Information about what's happening in the community that could be immediately accessed by relevant service providers is also often absent. This remains a significant challenge. We are trying to digitize this as well, but it hasn't been fully realized, representing a transition phase.

**Interviewer:** Hmmm…… so in your view, beyond these building blocks, is community engagement also a factor that contributes to health resilience?

**Respondent:** Yes, absolutely! In our communities, we have **indigenous methods of communication** that can be effectively utilized during outbreaks, and I believe we have used them. For instance, we have **public radios in communities**; individuals move around with these radios to disseminate any information they want to communicate. We also have various **community leaders**, including Local Council (LC) leaders, church leaders, and people in women's groups. These are all avenues we can utilize.

However, during outbreaks, we don't always fully leverage them. In more rural settings, it's generally easier to communicate with the community. In more urban settings, we face a challenge in communication because people are not as connected. Of course, what we primarily use now is **social media,** but it has a tendency to spread misinformation. Nevertheless, social media exists for communication in urban settings, and it's probably the most feasible way for us to communicate, along with traditional media like radios and TVs in urban areas. That's what we primarily rely on. So, when there's a need, I believe we can bring communities together; we have a number of channels available.

**Interviewer:** Ok, what I gather here is that community engagement works hand-in-hand to improve the health system's capacities.

**Respondent:** Yes, there are structures we can use if we choose to utilize them. So, sometimes the onus is on us to leverage the existing structures. The communication structures, especially in health, are indeed there.

**Interviewer:** Thank you so much. Now, I want us to look at interventions. As you mentioned earlier, our system is ranked highly, implying effective interventions. Particularly when considering outbreaks, which specific policies or programs have significantly impacted strengthening health system resilience?

**Respondent:** That's not entirely my area of expertise, so I might not be able to fully exhaust it. However, I believe what has been particularly helpful is the existence of a **national emergency operational center,** and within the Ministry of Health, we have a dedicated **Department for Disease Surveillance.** So, when an outbreak occurs, the country has been able to immediately investigate. For example, if they suspect Ebola, they can investigate, and once an outbreak is confirmed, they can activate the response structures. Their **guidelines are well-established at both national and district levels,** and they are also aligned with international regulations for managing outbreaks. So, the structures exist, along with the guidelines to follow.

There are different committees that typically swing into action during an outbreak: those concerned with the budget, with risk communication, and with infection prevention and control. All these committees and structures are in place, and I believe that could be the reason we were able to respond effectively and be ranked highly. We didn't have to scramble and figure out what to do because the structures already existed and immediately went into operation. We also have **capacity in terms of human resources.** We have field epidemiologists; we have training programs here at the School of Public Health and at the Ministry of Health. Several people have been trained through inter-intermediate programs, creating a pool of individuals capable of conducting the surveillance needed during an outbreak and identifying factors leading to exposure. So, I believe our human resource capacity is trained and already in place with appropriate guidelines. Furthermore, we've received **significant support from various partners during these outbreaks**, and they've been able to provide the necessary funding to support these initiatives. Some of this funding has come through vertical systems. While the government naturally has limited funding, as we mentioned, it has been able to provide a contingency fund. The government is also able to draw on funds, as demonstrated during the COVID-19 pandemic. I believe these financial aspects, coupled with strong leadership in relevant areas, have been crucial. For instance, the leadership within the Ministry of Health, including the Commissioner in charge of Disease Surveillance and the heads of the Emergency Operations Center (EOC), has been robust in directing the necessary actions. Furthermore, the country performs well in **risk communication.** Whenever there's an outbreak, information is widely disseminated through billboards for public awareness, such as explaining how diseases like Mpox present, and providing guidance to schools. In terms of risk communication, I believe we've been effective in ensuring people understand the risks and what actions they need to take. We have also taken necessary actions, including **quarantining individuals** when required. We know that even at entry points, such as airports, we have systems in place depending on the traveler's origin, like checking for yellow fever vaccinations. So, at border entry points, strict measures are maintained to prevent the entry of individuals who might expose the population to diseases. These are some of the specific aspects that have been functioning effectively.

**Interviewer:** Thank you so much, Professor. You've provided a comprehensive picture of our health system, including its current status and past actions. Before we conclude, you mentioned reforms, an area where we face challenges. What significant reforms do you believe are crucial for improvement?

**Respondent:** I previously mentioned **finance** as one critical area. The Public Finance Management (PFM) Act, which mandates that all collected money be sent to the central government before distribution, can truly hinder a quick, immediate response during an emergency. This regulation needs to have a **waiver clause for epidemics.** While it has its strengths, it should be revised to ensure flexibility in such situations.

I also discussed the **highly conditional grants allocated to Districts**. We need to provide districts with more funds and greater **decision-making autonomy** as managers, so they have more resources they can reallocate to meet their immediate needs during emergencies. Even before that, the overall funding needs to increase. Perhaps we also need a **contingency plan at the district level** so they don't have to wait for funds from the central government during an emergency. Reducing these conditionalities to give more decision space is crucial.

Lastly, for me, making health information systems **interoperable and integrated** is a critical reform. All major health systems should be able to communicate with each other. This would allow us to access necessary information, such as drug inventories, the number of available health workers, and suppliers. To achieve full access in terms of our health workforce, we need to restructure to have a larger **community workforce**. The community health workers we currently have, mostly Village Health Teams (VHTs), are volunteers. During an outbreak, a significant amount of work needs to be done in the community, and some tasks don't even require health workers. You talked about community engagement earlier, and to effectively do that work, you need that kind of community workforce.

We also need to reform the way we engage **inter-sectoral workers.** Currently, each ministry has its own workers. There are times during an outbreak when you need social workers to counsel people, for example; many situations require counseling. This highlights that when we are reforming, we also need a more **team-based approach** to our work. Right now, people often work in isolation—individuals in agriculture, sanitation, etc. We need an inter-sectoral, team-based approach to utilizing these resources.

Finally, as I mentioned, we need to be a **learning organization**. We need to do more in terms of **access to information, generating appropriate evidence, and ensuring we learn from that evidence to improve our practices** because things are constantly changing. For example, regarding how we handled the Ebola outbreak from the first time we experienced it, all health workers need access to that information. So, we need to do more in that area and learn from those who are performing better. We also need to learn when it comes to infection prevention; you find health workers sometimes continuing practices that expose people to infections. So, when we talk about reforms in the area of evidence, sharing best practices, and learning about the most updated methods we need to use, there's significant room for us to introduce reforms.

**Interviewer:** Thank you so much. You had discussed providing VHTs with incentives. Beyond that, what policy changes do you think can be implemented to improve their work?

**Respondent:** Incentives are good, but the kind of people we call VHTs—if they've only completed Primary Seven, they might not be able to manage all tasks. So, we need to pay attention to the **qualifications of individuals we bring in as VHTs**. Furthermore, to retain them, we need to establish **career progression** opportunities, because no one wants to remain at the same level indefinitely. Can we create a career path so that if someone starts as a VHT and works for some time, they can move forward? Of course, **training and continuously improving their capacity** as we train them are crucial. The training needs to be effective. Are we giving them certificates? How do we ensure they have continuing professional education? So, their **supervision and mentorship are equally important.**

**Interviewer:** Thank you so much Prof. You mentioned successful models and China building a hospital during COVID-19, and isolation centers. Are there any other successful models in other countries that you think could be adapted here, despite our high ranking?

**Respondent:** Not particularly. We just need to focus on doing what we need to do. I think **Rwanda often achieves a lot because they have strong political will.** They also demonstrate compliance with what they choose to do. People often say that we develop policies in Uganda, and they are then implemented elsewhere. We have the right ideas, which is why, for example, with Ebola, when we decide what to do, we do it. We know what needs to be done, but we sometimes **fail on implementation.** That's why countries that effectively implement policies progress and go further. So, perhaps that emphasis on **implementation and continuous adjustment** is necessary to get where we need to be.

**Interviewer:** Thank you so much for contributing these insights. I believe they will contribute significantly to understanding health system resilience in Uganda. Do you feel there's anything else you'd like to add that was left out?

**Respondent: No**

**Interviewer:** Ok, thank you so much.
