# Supplementary material for: Resilience as recovery: stakeholder perspectives on Uganda's health system response to infectious disease shocks
Source: Front Health Serv. 2026 Jul 22;6:1888814. doi: 10.3389/frhs.2026.1888814 (PMC13437620; doi:10.3389/frhs.2026.1888814)
Supplement: Supplementary file 2 [file Datasheet2.zip › KII03.docx]

**XXXX**

**Int: Thank you so much Doctor for accepting to participate in this interview.**

**So as someone who is grounded into the health systems architecture, could you please explain to me what you understand by the term ‘Health systems Resilience’?**

Res: Okay - thank you Deo for this: two things - one, is that it is now a uniform word - everyone is talking about health systems resilience and trying to understand what it means - it is very important; so, I want to start with appreciating the effort that the research is making to try to unpack systems resilience. Many of these concepts are used but understanding really defers - I think from the practical sense but also from an academic sense. Health systems resilience brings together 3 words but I could say we have health systems and we have resilience; and for me when I hear the word ‘health systems’, I always imagine a set of inter related components that interact and have to work together to reach certain results - and when we look at systems, they are by design complex and for most people we appreciate that systems are complex and health systems for that matter are complex because there are social systems that bring together different people different actors; there are different objectives to be achieved. So, before we talk about resilience, we need to first of all understand health systems and how they behave. So, from our understanding, complex systems are actually - in addition to having components interacting together, have properties that make them by design, hard to predict in terms of behaviors. So, uncertainty is one important aspect of health systems. What this means is that even a small change can trigger a very broad reaction within the system. Health systems also have another important component which is very important especially to the debate around resilience - we call it emergency. Emergency is that the property that the system develops - this whole piece that we are calling health system is greater than or has different greater attributes compared to the different components that make up the system. So, what that means is that the properties of the health system when looked as a whole would be different from how the different components would behave on their own. So, in that case we need to appreciate the importance of the health system and many people talk about the health systems’ building blocks but we admit that that is a very good attempt but it’s not sufficient to capture the complexity of the dynamics within the system. So, people will talk about financing, governance, service delivery, medicines - but that’s part of the story especially when you look at those components or those different building blocks or functions of the health system - we need to ask ourselves: what makes them or what makes their interaction of their combination to become a health system? And if you understand what requires us - we have what we call systems thinking - as we talk about health systems, we need to think about properties of this system; looking at health system from that perspective, helps us to appreciate resilience better. So really systems thinking requires us to look at this interaction, these dynamics, the unpredictability that is within this system. So, when we come from that perspective, the concept of resilience makes us to ask ourselves ‘does it apply sufficiently?’, ‘does it capture the properties of the system?’. Resilience is seen in terms of being able to absorb shocks, being able to maintain basic functions but also there are times when the system must transform; so, from that we have adaptive resilience, we have transformative resilience as part of the concepts that bring together the concept of resilience. So, I would say we appreciate the system has to be able to maintain some stability in terms of shocks but because by design, systems are complex and by design they show these properties of complex systems, I would imagine health systems resilience is also- I don’t know - a status or spectrum of state of affairs - but I would imagine it is something systems have to keep adjusting all these changes because by design, they have to have those attributes - they have to be able to adapt, they have to be able to self-organize and they have to be able to transform. So, Resilience in that case starts the debate but I think it may not capture the true extent of properties of complex systems especially from health systems perspective.

**Int: Thank you. So, what according to you Doctor, are the key attributes that constitute a resilient health system?**

Res: I think we need to appreciate like I have mentioned previously, that by design, complex systems have their behaviours and for me calling the system resilient, we need to appreciate resilient in terms of what? Or is resilience the desired attribute of the system? Okay? - so we are saying looking at the system, you have these shocks, external factors and we know that once you are faced with shocks- internal or external, the system has to re-organize itself to be able to adapt to that new state. So, I don’t know if they are the attributes of the resilient health system or they are the attributes of any complex system - because by design, any complex systems have to show certain properties whether you call it resilient or not but we know the systems have to show properties of uncertainty, feed-back loops that change in one direction can bring back change in another direction. You have the aspect of emergency that I talked about; so, from that perspective, I would imagine if we look at attributes of a complex system, then those should be able to align well with what I would call a resilient health system.

**Int: okay. So, lets switch to our context here in Uganda. In as far as resilience to major infectious disease disruptions are concerned, how would you best describe the Ugandan health system?**

Res: I have just completed some assignment looking at the global health security landscape in Uganda and for me there are strengths within the health system that really stand out; whether that constitutes a resilient system is a different story but I think we acknowledge that we have had a rich history with epidemics and this has got into our profile as a country and we have also built capacities in different areas.

From the policy side we have the frameworks - we have the National Health Plan for health security, we have the Public Health Act, we have ‘The One-Health’ strategies, we have the attempt to create structures to support preparedness, response as well as mitigation and recovery from these epidemics. We have the National Epidemics Operations Command Centre - these have been efforts to regionalize them so that they try to manage operations across the many districts because now we have so many districts - so they are now trying to create a regional structure so that you have stationed capacity at the regional level which can be mobilized quickly before even the national people come in.

We also see efforts within that same space, to use the good will of the donor community and for a big part the USAID, the CDC - who have been very active. We have had funding from the British government - UKAID FCDO- that is Foreign Commonwealth and Development Office of the UK and others that have been trying to support the health security agenda in the country. Of course, with the disruptions and re-prioritization of the whole of the US funding, we could have a different story around what gaps have been shown right now after the January 2025 changes by President Trump - but by and large, I think we have internal capacity.

There has also been an effort by government to get on board new partners - the Russians have been here; they had a conference last year about health security - the first one in Africa and for me it was an expression of their interest in partnering with us. They provided the new vision and for us they are providing aid with less conditionalities unlike the Americans- ‘we are not going to interfere in your politics’ and all those things.

So, government is also trying to diversify the donors. Like the Russians who brought here the mobile laboratories so that they can have that big foot in our context. But I want also to say that our resilience is contextualized as we especially talk about health systems - because within the health systems we have other systems, so when you look at Uganda, we are within a region that is inflicted by these challenges of epidemics and so in that regard, our resilience or our capacities or whatever you call it - preparedness, has to be seen in light of that regional preparedness and the capacities within the region. So, Uganda is as safe as the neighbors - so like the regional frameworks are very critical here. There have been efforts to create an established East African Public Health Laboratory System and apart from that, the cross-border discussions of how we can work with our neighbors especially for the vulnerable border points in western Uganda - like the border with Kasese and DRC or Busia and many others. So, we need to look at that as part of our resilience because really, we have vulnerabilities in these regions.

The other areas that I can think about now which also is very important, is that we adopted a new Pandemic Treaty last week but one at the 78^th^ World Health assembly - and this is a framework that WHO and member countries have adopted to try and create this cohesion and harmonized efforts to try and address these pandemics which I think is part of the broader picture of resilience in the country but also in the region and also globally because as I said, we are safe as our neighbors, we are safe as a region.

Also, we have to look at the roles of the different bodies especially at the regional level - Africa CDC, WHO AFRO I think are very strong in this debate of resilience or whatever departments of our health systems to accommodate these needs.

We have human resource capacities that have been built. We have the Field Epidemiology programs; we have AFENET supporting some; we have the National Institute of Public Health; we also have the students at the School of Public Health who engage in epidemics but the challenge there is always the coordination of these efforts across the different actors and the issues of mandates.

We can’t talk about resilience and fail to talk about One Health; so the One Health business is also very important to this - how does health work with the environment? How does One Health with Wildlife Authority, Agriculture, MAAIF, National water, NEMA issues of climate change come in - they are all part of the picture in my view. We are talking about climate-induced disasters and all these epidemics that are disaster related to climate change issues like cholera and even malaria itself can turn into an epidemic if not handled well like the water reservoirs. We cannot also leave out the discussion around that coordination and collaborative capacity as part of resilience. When you talk about the framework for health systems resilience, I think for me we should take into account issues of multi-sectorality, multisectoral contributions, cross sectoral mandates - interdependecies - these for me constitute the key aspects of this resilience architecture.

I would also want to consider the effort to create a One Health Platform where these different sectors come and talk about the current evidences that are really based on goodwill, donor funding. So there hasn’t been much appreciation in institutionalizing our systems; so, it is more or less ad hoc and goodwill of the actors.

Talking about financing I think it is also another dimension that we really need to think about. May be partly, the story has been that money is available for response but majorly from the donors and now that they have pulled out, I think that is a threat to our system capacities - much of our response has been supported by USAID and the CDC - and so for me that is very critical. We have seen some funding from the World Bank - the Pandemic Fund to help us boost that effort. There have also been regional efforts to create an insurance. There have also been some funds for regional epidemics. So, for me all these are part of the picture of what capacities we have - I am using capacities because may be resilience is a capacity, maybe it is a property, maybe it is an outcome, maybe it is a means to something.

But allow me to speak to the fact that when we talk about the resilience of a health system, we are reminded of the sector goal which is accelerating progress towards universal health coverage and issues of access to services are critical - which populations are benefiting from these services; issues of financial burden when people access care become very important. And so, may be as we look at the outcomes of this resilience, is it in terms of universal health coverage? Is it in terms of preventing epidemics? Or having residual capacity that you can mobilize any time in case of an emergency. So bottom line, I would think Uganda has strength in certain areas - surveillance, issues of human resource but these have to be also contextualized within the changing geo-politics and the donor efforts and also efforts around One Health approach to this.

**Int: Thank you Doctor for that elaborative explanation. Now, taking Ebola or COVID 19 as an example of some of the major infectious disease outbreaks we have had as a country, what are your thoughts on the roles of each of the building blocks on the resilience of the health system? You had earlier on mentioned something about this.**

Res: Oh yeah, I did that during the overview submission and you note that my biggest focus has been on issues of governance, issues of financing, issues of human resources - but I think if we start with the governance issues, we are working together. COVID-19 showed that the state can be mobilized- we had the president in charge of the whole response; they had to bring in Dr. Kyobe who is an army officer to command the Incidence Centre- and for me that kind of military approach- top down, can be very useful under certain circumstances like during that time. But then there is also need to be a lot of community engagement, there has to be a lot of participation, buy in; so one of the aspects I think is very critical in this is the state- citizen relationships - still under governance - how do people relate with the government? How is the trust between the government and the people? Do the people really listen to the government or willingly offer information? Are they going to run away? What is the role of the police? What’s the role of the Military? What’s the role of that relationship? I think for me as part of the governance question, on issues of human rights, at what point do we enforce, negotiate with the people? Are really part of the discussion around governance issues. With governance leadership and management, you can’t leave out the legal frame works - we talk about the Public Health Act, those policies like the National Health Plan - I think they are very strong in shaping our response. We also have the National Task Force headed by the Office of the Prime Minister and during COVID-19 you could vividly see that these were mobilized even getting additional resources from the community and other actors. So that is very important.

COVID-19 also demonstrated of course the multisectoral approach - you needed to get permits from the Ministry of Transport; we had people in trade managing the markets; we had those situations around school closures but how do we sustain learning in that context? - so the whole issues of multisectoral governance and collaborative governance is very important.

Also related within that domain, is the aspect of district task forces - DTFs - they had roles to play; what’s the role of the RDCs? What’s the role of LC5s? How do they work with the Department of Health? How do the different departments work at the different levels because that’s very important - a main decentralized system to have the local government and city authorities well prepared to respond to that. So we need to look at the multisectoral element of this; we need to look at the multilevel; we need to look at the state- citizen relationship and how these converge to doing the other aspects together - so for me, we could talk about all the building blocks but I think the governance issue is very critical, it also shapes on how financial resources are mobilized; it also shapes on how the human resources are mobilized- who is recruited to support this? What’s the set up to have different entities provide their respective support.

Then also you look at the levels above government- the East African level, the regional level, Africa CDC, WHO- as part of the governance component.

In terms of financing I think usually it can be considered - may be expected that much of the financing is going to be mobilized during the response phase and preparedness - these tend to have less financing recovery - but I think sometimes it is understandable because of competing priorities and you wouldn’t want to be spending the same amounts of money during preparedness as you would during the response - but then there has to be availability of resources to train these officials; we are getting into an election year for example a new set of people are going to come to parliament - the local governments - I don’t know about the president - but in change of leadership then there has to be resources to build capacity and to orient them but people need to understand the inter dependence that they have. And so, for me I would say governance - leadership management takes a central place and then the other building blocks can pick from there.

**Int: Thank you so much. How would you then view the community engagement as a factor in this aspect of the COVID 19 Resilience issue?**

Res: Yeah, I have emphasized the role of the state - citizen relationship and I think during COVID it was very apparent that how will the people perceive their government is very critical for the pandemic response. First of all, do the people trust the message that you are giving them? If you say we have had a surge of cases, would they really believe in you or they would think it is a ploy may be to get money or a plot to support corruption? So, during COVID it was apparent from the reports- at least from the Auditor General and the Finance Ministry had lots of background or behind-the-scenes concerns around procurement, around corruption, issues of where the tests are to be done; so for me all these speak to the picture of a community.

But then also the community could mean a lot of things; so, when we talk about the community - the wanainchi-, I think that plays a big role - do they understand what we are dealing with? Do they understand their roles? Are they supposed to be recognized by signs and symptoms and report? Are they supposed to hide their relatives? How do they reconcile the public health messages with their social norms and values? Especially how you handle the dead; how you manage these epidemics; social gatherings also are occasional opportunities to spread infections- you have for example imbalu that is a very big ceremony for the people in the East but also other cultural events, burials, church and all that; so all these have roles to play and I think community engagement becomes very important.

During COVID-19 also, we saw another community - which was a scientific community- and you had a Scientific Advisory Committee with sub-committees; providing technical advice to the state and I think that is a very important community as well - how they are able to synthesize experiences from other settings, provide guidance to the decision makers and that becomes a very important community.

The other aspect that I want to comment about is really around gender, equity and social inclusion. So usually there are some communities which are affected. We did some work in Kisenyi around COVID-19 lockdown; being a slum area people depend on informal jobs, so the issues of food distribution are very critical to them and these are places that are vulnerable to epidemic spread because of overcrowding and the living conditions - but then we also saw efforts of people to earn, people to mobilize new businesses and so communities are also trying to adapt to this new change brought about by COVID-19. So, I think that is what comes to mind - the role of community health workers has also been mentioned - how do they support the response? Are we putting them at risk? Have we prepared them? Do they have protective gears? Are we really compensating them for their time? Is there any compensation in case of getting diseases like Ebola? So, the community engagement and the role of this community and the players are very important.

The interaction with animal health people; how are we working with the extension workers for the people in Agriculture, in animal health? How are we looking at these farms as potential source of pathogens? How are we cascading down to the community level I think is very important. So those are some of the issues.

**Int: Thank you Doctor.**  **Given the array of points on what government has actually done in as far as confronting these epidemics is concerned, what specific programs have had a noticeable impact in strengthening the health system’s resilience, in your view?**

Res: Noticeable programs – well, I would say in terms of human resource, there have been programs to prepare a cadre who is able to respond to epidemics outbreak. We have had the Field Epidemiology Fellowship Program - and initially under the school here, which later on transformed into the National Institute of Public Health with support of some colleagues in the school here. I think that is a program where we can of course ask ourselves like ‘what opportunities are there for students who are doing Masters here to be part of (that)?’; how do we involve them in epidemics so that before they get out of here, they have those competencies. So, I think there have been some effort within the school here to engage the students as well to build the capacities which are required. But epidemics do not only need field epidemiologists - you need people with behavioral change, you need community development experts, you need anthropologists, you need finance people, you need modelers; so I think that skills set needs to be expanded - it may not be adequate. And now when you talk about noticeable change, I think there is always a challenge at these benefits or successes sustainable beyond the funding out lays that are available; most of these programs are heavily donor reliant especially the field epidemiology fellowship. When you hear the funds, the students are paid - like to maintain a student then may be there could be concerns around sustainability of such programs. So, in as much as we have had these efforts going on, there is always a threat of whether that success is sustainable.

Any other issues are really in terms of building capacity and I think One Health has tried; we have had the AFROHUN people - AFROHUN is Africa One Health University Network through their field experiential learning programs. They have engaged students here in One Health institute and so they have done a lot of contribution to one youth work development in the country and beyond. We have had tackling deadly diseases’ programs - I know they are starting a second phase TDDAP2 which is currently being rolled out by Palladium - but I think they also promise some kind of effort to support national and community level efforts - works around the National Action Plan for Public Health sector Security which has been very critical. The programs that IDI has led, they have what they call Security Program - I don’t have the details but at least they have had their name. BAYLOR has also been another player in this space managing some efforts around that. So, the school - National Institute of Public Health are also some of the critical players- Yeah.

**Int: Thank you Doctor. Now, with that rich explanation, could you briefly paint a picture for me of what a resilient health system looks like particularly in response to infectious disease outbreaks like the case of COVID 19 that we had about 4 years ago?**

Res: okay - in sciences we have what we call normative view - what should be and what is; I think for me when you talk about resilience, it means there is a desired state of affairs that we want to see and with that it means we are able to work towards some status - some expected state of affairs. So, within that line, people have tried to provide things like systems that are able to detect some shocks, should be able to mobilize necessary capacities and capabilities to respond and where necessary should be able to transform into more resilient or resistant modes of operation.

But in my view a resilient health system should have a clear sense of the actors who know their respective roles and have been cultured or socialized in a way to be able to play those roles in a coherent way and being able to do that in a way that enhances out comes like provision of services or continued basic social functionality. So that would be the case but from a system thinking perspective, a resilient system may be far from a reality because whatever external shock a system is faced with, it is going to demonstrate some properties; it is going to demonstrate feedback loops that one move step forward may create another - either a way forward which we call the balancing loops - in other words- rather - no - the reinforcing loops- for example improving the training of health workers - improving their privileges may be looked at as a positive state of affairs but if you don’t deploy them - may be if they can’t all be absorbed in the system then that creates a gap because now you have redundant staff which may create a whole set of issues- they may decide to disappear or they may decide to take on other tasks; which means that in as much as you try to push the system towards a certain direction of having qualified Epidemiologists or you may create unintended negative outcome where they are so many and are fighting each other. So, then a resilient system in that case should be able to continuously adapt; if things are moving in a given direction that is positive, at what point does this system have checks and balances so that we need to go slow on this. Like we have the challenge of interns which could be related to this; so, we have created so many training institutions, so many people are coming out, so many Doctors - so from the face value, you are having many interns many Doctors graduating and may be looked at as a positive attribute of a system. But at the point when they are having disruptions, people are talking about low quality, then that becomes a counter intuitive kind of progress. So indeed, a resilient system should have those capacities to sense the direction and acknowledge this feedback loops, acknowledge the unintended outcomes and be able to correct things.

For example, during COVID, there was a lot of uncertainty because so many things were new! And on a hindsight when you look back, you may look at certain actions as being bad! People have talked about lockdowns, people have criticized that whole process, people have criticized the effort to provide the social protection - the food subsidies and all those. But at that point in time, there were needed actions before the chaos came in and there was a time when they said enough is enough and we need to do away with these lock downs - we need to open up society, we need to open up the economy, we need to open up schools; for me that then is a resilient system. - that it has learnt and has been able to read the signals and has been able to acknowledge unintended out comes and is now scaling back on some actions which are created; but whatever you do, whatever investments you make from a sense of a complex health system, there will be unintended out comes and solutions of today may become problems of tomorrow. So, we need to be aware that there is no static measure of resilience from that point of review.

**Int: Thank you. In your opinion Doctor what are some unique attributes about the Ugandan health system that contributes to its resilience especially in the face of public health challenges still with COVID 19 as a reference?**

Res: I think I had hinted on some of these already - issues of governance was very prominent during COVID - the whole of government approach- the whole of society approach - everyone was concerned! Everyone was mobilized to some degree; even none health people, corporate entities started donating pickups- I don’t know what intentions they had but we can acknowledge that people saw this epidemic as a challenge to their interests and their business and their operations; and so in that respect, COVID 19 gave us a chance to see in real time how you can mobilize the whole community and I think for me, that good will is still there if the state justifies or is able to make a case to the people and for me that makes it a unique attribute. So, these experiences being mobilized, documenting them is another unique attribute of the Ugandan health system.

I would also say that Uganda is blessed to have Ugandans experts in the regional and global bodies - people in the Africa CDC, we have people in WHO Afro and all those others. So, for me these speak to the unique capacities Uganda has and has been able to export to benefit the wider regional and global communities.

I also think we have tried really to make One Health work - not yet there but we have tried and so that is one other important unique attribute for the Ugandan health system.

We also have the legal instruments - we have the constitution, we have these strategies, we have the public health Act which can be mobilized at any given time to support either action or enforce certain efforts that are ongoing. So having a good set of legal frameworks is very important.

I think we have credibility that has been built over time by our institutions to manage these epidemics and that has been a major source of inspiration for Uganda that we also have a story to tell that we have been able to successfully manage these different epidemics. So, this rich history is very important.

Also, donor support becomes another attribute because the funding has been provided over time and then of course the capacities - local experts, universities, institutions all contribute to our capabilities as a country - yeah.

**Int: Thank you Doctor. So what challenges do you think are affecting the Ugandan health system’s ability to with stand and recover from infectious disease shocks?**

Res: With the challenges I think I could divide them into 2; some are really structural - they are really who we are and what we are as a country - being in the Great Lakes Region is a big vulnerability we have and also having these big open borders - being land locked; so this is another important aspect of our context that we can’t change and so we have those vulnerabilities.

We also have another challenge regarding One Health still although we are making progress and I think much of this is being dominated by the health sector and less of environmental health people and getting these new charges that are coming that are permit related, also becomes another concern, threat that we need to watch out. And so, for me those stand out. and of course, I also talked about the citizen-state relationship; if you have a very rough issue with the state, then you are likely to see resistance to even unintended government initiatives and programs - once people lose trust in their government even when you declare an epidemic - may be if those guys want to ‘eat’ that money.

The other critical challenge we have and related to compliance to the global mandates- the international regulations 2005 and I think the Pandemic Treaty, all demand us to report once we see some notifiable conditions you have to report. This brings a lot of risks to the economy, a lot of risks to our tourism because as you try to be transparent, other countries close their borders, shut your businesses. At one time they declared a bad flu epidemic and I think it was Tanzania- people stopped taking our chicken thereby causing a very big challenge to the markets. So how do we communicate these epidemics but most importantly how we balance being compliant with these regional bodies also maintains some element of national control over that.

The other challenge which is not well spoken about or that is emerging is the issue of sovereignty and dependence on the donors - at what point does Uganda say that this is what we think to be important for our country. I would really relate this to the US and am aware that government is trying to scale back its interaction with the American government in reinforcing the Public Health Emergency Command Centre to become a government entity and the department of surveillance and epidemic something; so that is an effort. And there have been also efforts to transfer testing from UVRI to the public health laboratory - all these as efforts for government to control this story of how to communicate information and also to protect pathogens from being exploited. You have heard Dr. Musenero a lot talking about the pathogen economy and how we can harness this. There has been concerns during the Kassanda district Ebola response - the minister came out to openly complain about donors and how they were misusing data and how they were abusing the local system. So that is going to be an ongoing challenge really - how does Uganda relate with these donors’ interests. So we have talked about the structural things that we can’t change- the environment, the economy, the geography, the donor dependence but I also talked about the aspect of the actors and their interests- how do we work in a multisectoral way, how do we work in a coordinated way, how do we relate with the donors - and I think those are the two categories of forces that we need to contend with - yeah.

**Int: Okay - so, Doctor how do you think these challenges can be overcome at both national and local levels?**

Res: okay - one of them is to ‘relocate Uganda from the Great Lakes Region’ (Laughter)! - so if you may be transfer it to Iceland or elsewhere where the pathogens cant thrive, then we are in good hands, the pathogens will die in ice instead of having them in the tropics or in a weather that is favorable, we shall have sorted out the problem!!! - in other words, the point I am trying to make is that there are some things we cannot change by virtue of who we are and whatever as a country! But then there are things that we can try to influence - I don’t think whether we have full control over everything - issues of the donors, state-citizen relationship I think is something that is very central to the government and how it negotiates its actions with other donors is very important. So that has to be strengthened.

We also need to think about the politics and political economy of this resilience business. We are talking about framing- what does resilience mean? I am happy this study is interested in that. So, if you don’t have a common language that people appreciate, it creates a problem. So, we need to develop I think, shared understanding of these concepts of these issues - like what does resilience mean? Do we have measures? So, there is some aspect there of even fine tuning our analytical tools.

Then the other one is of course - I think Uganda - as it has been demonstrated by the Pandemic Treaty, I think needs to build stronger coalition with neighbors and the other countries in the region. Collective bargaining can be very important. I remember during COVID, we had the issue of vaccines and there was that story of COVAX and the rich countries that had monopoly over these vaccines until they got a bit ashamed and then decided to give something to us.

I know we have been having challenges with absorbing some of these vaccines some having expired and have had to be disposed of. But bottom line, I think Uganda needs to work with the other neighbors - with the region but also Africans to mobilize critical resources. That also speaks to other capacities like manufacturing of key ingredients, vaccines. We had people making soap, we had the hand - what is it called?- the one people put on the fingers? - disinfectants? [sanitizers]. I think those ones; so local manufacturing capacity is very important. I know something is going on in the African region to have a manufacturing plant in Rwanda and that is going to be very important.

And then of course these issues of Uganda being very vocal during the World health assembly 2 weeks ago making a statement of a need to adopt a treaty but also to support countries. So, engaging in that space is going to be a continued requirement for Uganda; we can’t go in alone - we can’t lay isolated - we need to mobilize other colleagues to join us in this journey.

And then of course the budgeting issues have got to be looked at. How are we prioritizing our resources to be able to address epidemic preparedness? To also be able to have a range of investments that would give us good results either in the short or in the long term. So that is going to be very important.

The analytics is very important - people are talking about disease modelling, people are talking about systems modelling - so these capacities have to be available to the country to be able to anticipate where they are likely to be. So, the analytics, artificial intelligence, machine learning - these capacities have to be developed and be used locally and also exported to support the other countries- Yeah.

**Int: Thank you Doctor and we are about to come to the end of our discussion. But allow me take you a little back where you talked about our VHTs where you were commenting that if we are to engage them, are we having the PPEs to give them so that they can work? Are we exposing them to danger? and so on. So how can these village health teams be better supported to contribute to developing a more resilient health system in our country?**

Res: I think the issues of working with VHTs do not only speak to the epidemics aspects but the issue of support to these VHTs has been of a concern; many of them work as ad hoc - that is okay- though there have been efforts to link them to facilities which is good. But then there are also distortions that have been brought in different programs working with VHTs to advance certain components of their work; so, then that has been a major concern I think for me. But I also know that government is piloting them to choose community health extension workers who should be stationed at parish level to offer support to the VHTs. These cadre have been used in Ethiopia where they have a lot of these VEQs and so we are also trying to pilot this. I am not very certain of the districts but I have heard of 4 districts where this is being piloted. So VHTs are very critical but the support for epidemics I think has to be integrated to other support otherwise the epidemics response health security may come off as another vertical program - that increases the burden to these issues - but I think we have also to look broadly at what we call critical local actors - you have the VHTs, you have the local councils - very critical, you have traditional healers; the Kassanda story is very important about the role of traditional healers to the extent that people went to these people for immediate care - may be they thought they were bewitched- and then that becomes an epicenter for the spread of this. So we need to broaden the discussion around VHTs who are the critical cadres to support response at the community level.

You are also talking about the parish development model arrangement by government bringing together the financing, social development at the community level. There is the parish development committee- how are we engaging with such? We have the extension workers from other sectors - from Agriculture - we have the community development officers - the CDOs- how are we working with them? We have the sub-county chiefs; we have the health inspectors - so how do we mobilize the community response? How do we mobilize a community system that is able to understand these issues and respond beyond VHTs? I think for me that is the central main story I would push forward instead of looking at VHTs as working in isolation.

**Int: Thank you. Are there any successful models from other countries or contexts that you think could be adapted to the Ugandan context in order to enhance the health systems’ resilience? Are you aware of any?**

Res: models in what aspect?

**Int: the approaches that other countries have used to bring down these epidemics that you think could be adapted here to enhance health systems resilience?**

Res: I am not very sure about that but what I know is that Uganda has been at the centre of exporting its expertise to other countries- I don’t know whether that means we are already at the promised land or we need additional support but I believe that is part of the story but we acknowledge that for a big part, the discussion is really about- how is Uganda exporting its story, its capacities to other countries? and not the other way round - this is something we need to look into. Some work from the AFRO HUN network could offer something here because they have had work in over 20 universities in over 10 countries in Africa and their platform is to share experiences and exchange localized information. For example, I am aware of a program in DRC which is about strengthening what we call territorial administrators - they are either below the districts or something like that. But we have similar arrangements- we have the District Task Forces, we have One Health - so I think we are doing well.

**Int: thank you so much Doctor for sharing those insights. Before we end this interview, do you have anything that you want to provide as a final thought on strengthening the Ugandan health system in the face of future health emergencies?**

Res: I think my points have been very clear; first of all I want to acknowledge that this is very important work - understanding how people frame and understanding issues - I think this is very important. Even this concept of health system resilience is a complex one and we continue to wrestle with which interpretation or understanding we need to advance - so that is very important.

Two, is to bring back systems thinking into this discussion of health systems resilience and I encourage the team to really look at how systems thinking can be useful to shape our understanding of health systems resilience - because once you understand the complexity in the system behaviour- issues of emergencies, issues of feedback loops, issues of interactions, issues of interdependecies, then your appreciation of what we call a system and what attributes you expect of it - whether you call it resilience or whatever, it might change well knowing that systems are in a state of flux in one way or another and movement in one direction may create balancing loops backwards and then that creates another chain of interactions and reactions. So bringing back systems thinking is going to be very critical in defining resilience.

I think the other issue in my view is strengthening multisectorality - this is not a health issue - it is all incumbent- the whole of society business- it has regional, it has local, it has supranational issues. So, we need to have that multidimensional approach in understanding the challenges and the roles and opportunities for change and then of course negotiating donor- country relationship to remain critical going forward- Yeah

**Int: Thank you so much Doctor for your time.**
