# Supplementary material for: Resilience as recovery: stakeholder perspectives on Uganda's health system response to infectious disease shocks
Source: Front Health Serv. 2026 Jul 22;6:1888814. doi: 10.3389/frhs.2026.1888814 (PMC13437620; doi:10.3389/frhs.2026.1888814)
Supplement: Supplementary file 2 [file Datasheet2.zip › KII04.docx]

**Transcript of interview with XXXX**

Interviewer: Today is 28th May 2025. So, the first question you could briefly first describe what a health system resilience is.

Respondent: So, why do you ask me what the definition is?

Interviewer: Basically, to understand your opinions about the health system resilience. Let us start the interview basically by describing the health system resilience Prof and then we also discuss the factors that also contribute to the ability of the Ugandan health system to withstand and recover from major disruptions.

Respondent: Anyway, so, a system resilience is the ability of a system to continue functioning or to adopt and continue functioning given shocks. Like the current shock of Trump, epidemics, climate change, strikes, all those things. So, in other words, it's a system that can adopt and continue functioning, providing services continuously but also with quality. And ideally, even reaching the most vulnerable so that the system works for them. The Ugandan system is actually quite resilient, surprisingly, despite poor financing. And because it sort of continues to function.

I think the main thing is that the people themselves provide most of the resources for care because most of the care is out of pocket. So, really, people don't depend so much on government. So, they sort of continue, even when they come to government, they know that they will still have to pay some minimal money. Number two is the fact that the government has also provided most of the basic things like infrastructure, although archaic, but it exists, human resources. And the third one is the availability of community health workers. And then also, because there is a big role of the private sector, maybe it aligns to my first point, that the government provides many more things, the people take care of themselves most of the time. So, the private sector continues to do a lot of the care.

Interviewer: What kind of private sector we are looking at, elaborate more?

Respondent: The clinics and stuff, they are part of the health system. And if you pay, you get service. And of course, there are also drug shops and stuff like that. But also, there is quite some governance and management of the sector, and it has data. You can see almost, the system is quite well connected, so you can get data. And then the role of NGOs that have been supporting, but some of them which continue, so they support the system. In some aspects, Uganda has taken a long time to learn from especially HIV/AIDS. They built a lot of capacity for system resilience, including HIV testing but hose platforms have been used to do like surveillance systems, to do laboratory care. And of late, a lot of the things have been taken up by the government. Donors have been going away. Donors are actually nowhere. Around 2000, when the sector-wide approach in swaps ended, the government started taking over a lot of the things and sometimes the role of donors is overrated, except of course in HIV.

Interviewer: So is it resilient?

Respondent: Well, fairly, it's not that resilient. I mean, too many people die. So you could also say that, even what we call system continuity function, it was already functioning at minimal functionality and we sort of used to that. But it is not resilient in doing high-quality things. That one is not.

Interviewer: Okay. So not being resilient in doing high-quality things, maybe you could tell me some of the characteristics that make a resilient health system.

Respondent: Well, a system has to provide services that are responsive to the needs of the people, and that are timely. I mean, you don't delay, that are high quality, that are appropriate, that really look so much into client needs. But we know that in general people, that the quality of services is poor. So I think the system is resilient in providing what it is used to. Yeah.

Interviewer: Any other characteristics?

Respondent: I said many things there, so many, many things there.

Interviewer: Okay, we can continue. So what are those unique characteristics about the Ugandan health system?

Respondent: I think I have answered that. I told you why it continues functioning. I mentioned so many when we were starting, I have answered all those questions.

Interviewer: Well, you discussed about the factors… Respondent: I answered that.

Interviewer: So still on community health workers, you could also tell me some of the roles of the community towards... You could tell me more.

Respondent: Well, the continuum. I think for me a starting point is the system is suboptimal already, even before shocks. So it has adapted to at least maintaining the minimum level of services and quality. Sort of, it has learned to survive, but it is not the system you would admire. Community health workers, of course, play a role in mobilizing community members, in educating and sometimes providing some services. Yeah.

Interviewer: How about the community itself?

Respondent: Community, I've already said that community... Provides the care by themselves, they dig into their pockets to get the care, most of the time. I think that's the main thing. Then there are these, of course, the elites who don't even use the public system. They find alternatives.

Interviewer: And about the challenges that affect the health system's ability, you talked of poor financing. Any other challenges that affect the health system's ability to withstand and recover from infectious disease shocks?

Respondent: Well, first of all, the system is so, what can I say, amorphous in a way, public, private, informal, too many levels, community, health center one, two, three, four, so many, NGOs and more. That makes it really an amorphous system. That becomes difficult to manage. Then, two, there is a lack of accreditation of the system. There are too many private clinics, for instance, and they have to be managed. But, like here in urban centers, people die in the city where they shouldn't be dying, maternal mortality, neonatal mortality, perinatal mortality are highest here in the city. And where people shouldn't be dying. Because the system is very bad at taking care of the poor. But also the system is, of course, there is the health care system and then the other parts which add to be called the system. For instance, too many external shocks like what you would call the social determinants are generally wrong. The governance, politics is weak in a way for the system. The environment is quite poor, I mean, so much pollution and other things. You have all these, in the cities, a lot of unregulated transport, like motorcycles, boda bodas causing too many accidents. And they put pressure on the system with those things. The private sector is unregulated. I mean, there is a law, but nobody cares really. They are being given a license to collect money. Then lack of proper referral system. People just walk into anywhere. And the human resources are limited, they are poorly managed, poorly distributed, they are a mess in the city, but even in the city, there is dual practice. But then, I already said, weak management in general. And the fact that we don't have health insurance, all those contribute to the system not really taking that resilience to another level. And then the verticalization, of course. These donors, sometimes they keep the system moving, which is artificial. I mean, like HIV/AIDS services, TB, malaria services have been disrupted because it's been so much, before, they were doing very well, because the donors, now the donors have withdrawn and the system is shaking. And of course, politically, it's not that prioritized. It gets very little money from the national profiles.

Interviewer: Okay. Thank you for sharing all that, and in your opinion, how can stakeholders work hand in hand to overcome some of these challenges?

Respondent: Well, the most is, I think the biggest problem is, there are three for me big problems, and maybe a fourth. One is, our community members do not demand so much.

They just accept what they get, because if people were to vote based on how they are satisfied with health care, these governments would not be in power. Or they would be more responsive. But, number two, is the political class doesn't, the ruling class, do not look at health as a very critical investment that contributes actually to the economy and they leave the health sector too. So we need to sensitize the public, we need to sensitize the politicians to allocate more resources, but also to appreciate the value of the sector.

The third one is, improving the management of the system, because you can save some money by being more efficient. Then finally, in the end, of course, we need increased, better economy, because without a better economy, then there is no money really to allocate.

Interviewer: Better economy, how can this be achieved?

Respondent: Well, if people are poor, they are not able to pay tax, and if there is no tax, there is no money for government to allocate. You have ten minutes, I have a meeting.

Interviewer: Yes, I will wind up shortly, share some of the strategies or initiatives that you propose to strengthen the health system and improve resilience to these shocks?

Respondent: I think those which I have said.

Interviewer: You have shared emphasizing the public, engaging politicians, improving management of the system, any other strategies?

Respondent: Yeah, and better use of data. Use a data-driven system to enhance decision making, resource allocation and respond to some health challenges, increasing health workers in the health facilities since we generally face a shortage in health workers and the health worker-population ratio is very low, blah, blah, blah. But all this comes from allocation of more resources.

Interviewer: So we earlier talked about the VHTs engagement, and to you, how can these VHTs be better supported or empowered to develop a more resilient health system?

Respondent: I didn't say VHTs.

Interviewer: Community health workers, VHTs, how can they better be supported?

Respondent: I think it is important to define better their roles, to get the right types of community health workers, and if we are going to depend on them, then we need to recruit them and pay them. Yeah, and also furnish them with the resources they need to carry out their activities.

Interviewer: Okay. And then, as we come to the end, are there any successful models that you think would suit the Ugandan context? Models in relation to how other areas, other countries or regions have been able to mitigate these shocks.

Respondent: I think that, number one, we are stressed with more resource investment. Countries more in the south of Africa have invested, they are giving a little bit more money to health. True countries like Ghana, Rwanda, Kenya have health insurance. Countries like Rwanda have community insurance. And there's need for more leadership from the government around what donors can do and what they can't do. Even the government needs to... Actually, the other problem factor which affects resilience is corruption, which is so rampant in the health sector. Finally, I think we need to do two other things. One, invest in family planning. There are too many births that we don't need, there are too many unplanned deliveries. Number two is advanced social determinants, things like ensuring the environment is safe and protected, the education and agriculture and stuff like that, road safety. All those things are important and drivers of good health and the resilience system. Because, for instance, educated people are more likely to take good care of themselves. Although many are also ignorant, they don't want to take in too much sugar.

Interviewer: Here in Uganda, could you share some of the successful models that have been able to support the system to recover from the shocks?

Respondent: Well, during COVID, the government allocated more resources across some key areas for instance in health systems response like provision of medical supplies and PPEs, communication, during enforcement and public safety, even social protection through food aid to the public. Then the use of data. The use of data, the strength of building on other systems, like using a lot of the HIV platform to make it as a platform to provide other services for instance during COVID, a lot of the testing for COVID was using platforms which were initially made for HIV. I think we've been also strengthening public health, which has been appreciated as an important part of the system. But in reality, most of our things are short term, because when you have a pandemic, everybody comes on board. Then after it's gone, everybody forgets. So I think we need to be sustaining the learning too. like all the good things which were done during COVID, very few of them are being sustained. Like bringing stakeholders together, involving the public, more use of data, increased resource mobilization and allocation, and more sensitization. All these are things that ideally should be routine. Of course, things like immunization have also helped because they address some of the diseases. So, yeah, as I said, Uganda is a bit, compared to other African countries, more resilient. But when you look at it in the whole, it is sustaining things which are already suboptimal. That is all I can share.

Interviewer: Just in case this wasn't talked about, the factors that are contributing to the ability of the Ugandan health system, I know you shared many but any factors in relation to areas of leadership, resources and other blocks of the health system?

I've talked about all those, so we need more, I don't need to mention or go by building blocks, blah, blah. I don't believe in the blocks. I've told you, governance and leadership has critical issues of accountability and more resources. Those go with, basically I've talked about every single block, without being so academic.

Interviewer: Okay, thank you so much for your time. And we would humbly request to contact you again incase of more enquiries in relation to the same.

Respondent: Yeah, yeah.
