# Supplementary material for: Resilience as recovery: stakeholder perspectives on Uganda's health system response to infectious disease shocks
Source: Front Health Serv. 2026 Jul 22;6:1888814. doi: 10.3389/frhs.2026.1888814 (PMC13437620; doi:10.3389/frhs.2026.1888814)
Supplement: Supplementary file 2 [file Datasheet2.zip › KII05.docx]

**TRANSCRIPT FOR KEY INFORMANT INTERVIEW.**

**Study title:** Health system resilience in the context of infectious diseases in Uganda: Exploring concepts and experiences, frameworks and tools.

**TRANSCRIPT:**

**I:** OK, thank you so much XXXX once again for accepting to be part of the study. As I had previously explained to you in the consent, without wasting time to re-explain, may be doctor if you don’t mind, kindly share with me your experience and views regarding health systems resilience specifically in relation to the Marburg outbreak in 2017 in Kween District.

**R:** OK ahmn, Thank you very much XXXX. First of all, thank you for giving me an opportunity to be part of it, I wasn’t really aware until my professor called me and told me about the need to be interviewed for this study. With that said, *uhm,* normally I don’t pass an opportunity to contribute to knowledge especially in regards to improving the response to infectious disease outbreak in our country. Reason being, as you noticed we are if not the first or maybe the second leading country in like getting many different out breaks over the years.

And talking about the health system, our resilience to some of it is a key component. Ah, something that we need to start looking at from time to time. Like for instance in my understanding of resilience, I think we are talking of things in line of preparing for some of these outbreaks a head of time and then also having the capacity to recover form the shock. Like the after activity like for instance, I am going to give you my experience from the recently concluded outbreaks. In 2017, I think I was still a young professional and I wasn’t so much into the outbreak. The contribution those was very minimal. So, these recently concluded outbreaks, I had a chance to be part of it, and I could see areas that we were strong, and areas where we were not. For instance I could give you an example, in terms of leadership, I think that the country has a very strong leadership, ahmn in terms of coordination, in terms of ah, quick mobilization to ensure that appropriate response is initiated and along that you realize that aside from having strong leadership, there are other parameter associated with having a strong system to respond like for instance If you look at the health work force. Ahmn, we talk about them being flexible, and well trained, but there are some core, core competence. During the outbreak, I could see that it was lacking in most of our health workers. More especially, I could see during my IPC activities, you realize that a healthcare worker could be, ah, he knows about what I supposed to do, but in practice they are not doing it. So, it is something, I saw as a gap, in that you know what to do, but I don’t know because of low interest, kind of poor perception regarding the pathogen we are dealing with. Some of them were very sluggish the way they were handling that IPC, more especially when it comes to time when we were supposed to handle patients, incubating contacts to be admitted in the quarantine centers. So, these are somethings that tell you that health workers need to be trained during certain outbreaks. In fact, if it is me, I think the country needs to have opportunities where health workers are trained on pathogens, for instance you mentioned Marburg, there is Ebola, there is may be viral hemorrhagic fever, some of these things are very life threatening like when it comes to mortality. So, we don’t want to be losing our health workers time and again in some of these things. So, I think that understanding that we need to protect them as our first respondents, because I saw, like in Mbale, we needed numbers to cover the response but the people who were there to respond, you could see that in terms of protection, they were below standards. In Kasanda it was different, a little bit of protection was there, you could see it but in Mbale I think it was lacking, but thank God the outbreak was not fatal as, like the case mortality was not as high as like in Mubende and Kasanda.

SO, I think that takes care of the health work force, then I also wanted to highlight something about service delivery. This is also another key area of health systems resilience. Now, my experience is that, I happen to be, I was in charge of many things, even in things like case management, ah, contact tracing and then going to investigate the suspected cases and that was around Mbale. One thing I noticed was that during that period, majority of the health workers who were mobilized were working on Ebola and when it comes to essential services, service delivery, like someone coming in for malaria, and coming in maybe for antenatal, and maybe coming in for OPD, there were no health workers to really help. There is point where some DHO’s which I may not mention their names, they were now not receptive because they had taken all the health workers they had and their health facilities literally empty and not having capacity to respond to these small, small emergencies of the population to the point that patients were supposed to be referred to the regional referral hospital but since there was issues of Ebola around, patients wouldn’t want to come. You get? So, some of these really contributed, can contribute to a health system that is not ready to deal with, let’s say an outbreak is taking a huge magnitude like what happened in Gulu with Ebola sometime back. So, I realized that the ability to make sure that we must continue providing essential health care services, that is still lacking in our setting more especially when there are outbreaks in a given area. You know we have to contain it in that particular region or something like that, but if they are to use all the health care workers available for that containment then there is a challenge, that means they are not ready in terms of having first responders or emergency responders who are ready to come in and give support. We had our partners but you could realize that they only do much financial support and then some that are involved in maybe global security directly, we would be able to give some epidemiological guidance but in terms of first responders, those ones were taken away from the mainstream health centres. Obviously, the common man down who has come to access services they were kind of denied that.

Then in terms of Health Information System, this one was something that I think that the country is really doing a lot bit well in this though I may not comment on the quality of the really timeliness of this data but what I know is that, we are utilizing a system quality data to try and make sure that this data has been made readily available for decision makers at the ministry level to be able to decide on the next cause of action and in that regard I realized that it was a lot of work for starters, imagine you are the quarantine site manager, you have to really ensure that you monitor contacts, after monitoring them, you come and enter information in the online register, there is an online register for the ministry, there is go-data, so this one at times I think that it still speaks inadequate work if we are to do some of these things. Basically that.

Then maybe in terms of community engagement, you know when there is an outbreak, one aspect that is very key, I think that we should always be trying to pay more attention to the community engagement. The way we engage community, somehow, they help us address the myth, help us address the fear, help us make the community feel that they are wanting what is happening. So for instance, I have realized that during the outbreak of Mbale, communicating the risk and communicating the situation was happening but at a high level but at the community level, you would rarely find people having information to the point where even in Mbale city even if you walk around they would tell you there was no Ebola, the risk was communicated at once I remember when the Minister came they communicated the risk through registration but then after that, I don’t know because sometimes as a health system we have to protect the information but somehow as basic as the information could be, we need to be telling our public that there is this risk in the community either through radio channels or through health education or using social workers to communicate this risk to people during this period when we are responding. I think it could be something that strengthen us because this operational one I felt like the community was not owning whatever it was, some people call it horn, you can imagine when we are dealing with the dangerous pathogen and the community which is at risk doesn’t believe in it. So those are some of the things I saw. Thank you so much, back to you.

I: Yes, thank you so much XXXXX, I really appreciate your understanding of the health system resilience, now still in line with that, as far as resilience to major infectious diseases disruption is concerned, how would you best describe the Ugandan health system from your own perspective bearing in mind that the partners that majorly contribute to our ability as a health system at national and subnational level to withstand and recover from major disruptions.

R: I kind of lost you at the beginning and at the end, but I think I have understood what you asked, just as I was saying, describing the Uganda health system ability to withstand the shock after infectious disease outbreak, I would say that we are not that resilient still, the reasoning is, this thing should be measured in terms of what you people are trying to do, I mean after an outbreak, are there people who are interested in going back there and assessing or evaluating the health system in that region, to find out when there was this outbreak? What was its impact on service delivery, what was its impact on health information system, what was its impact on health finance? What was its impact on things like the health workforce in that area?

Then in terms of essential service delivery, if we have not yet reached that level of always going back to assess ourselves after an outbreak and already the first thing that the government wants to do, or the ministry wants to do is first respond to the outbreak and then contain that, but after containing, that after part, I could say the ability to respond to it from my experience or from what we have been going through, we can respond to it up to 70%. The reason why am saying 70%, sometimes to do some of these things you need resources, for instance health financing, now we are 3^rd^ world country, some of these things we need to respond to this outbreak like PPEs and other essential commodities, we cannot readily afford them and if we can afford, they don’t come in term, like infections don’t tell us that today we are coming, be prepared. So that’s why I was saying, if we can reach a level where we have those early preparedness actions like for instance during simulation activities, once in a while I see it being done in some places but if we could do simulations of these outbreaks before it even comes in like also modeling the infectious disease, try and see the universal kind of correlation and see which places will potentially contribute to an outbreak in a country or where could we possibly see an outbreak.

Then using such simulation, we can be able to plan that you know what? Given what our data scientists or what our epidemiologists are saying, these areas have high potentials of outbreaks or maybe a certain condition. So, if we are not yet at that level of going back and assessing the impact of our response whether on mortality, whether on…and then another thing that we don’t look at is let’s say the psychological bit of it, there are also workers who respond. An outbreak of a very deadly hemorrhagic fever like Ebola or Marburg being placed with people mentally, you respond to that, come back to your family you don’t know whether you have carried the disease to your family, so do we have capacity to go and assess, what was the impact of this? The status of these health workers and their families, so I think some of these things we have not reached that level, so in terms of categorizing I would say 70%. To reach a level of maybe say 90 or 100% we are going to need a lot of basement in some of these areas that we have identified for instance it may require us to do something that we do through simulation, make sure that we plan, and also make sure that we are doing things like stock filing to understand the issues that are making us not transforming, so that’s what basically I can say. I think we are still at 70%.

**I: Thank you so much XXXXX and what do you think are those factors that are enabling us to reach 70%, if you could point them out quickly, the factors that contribute to our ability to respond to about 70%?**

R: This is actually very simple for instance, the number one factor is our history with infectious diseases as Uganda, we receive almost all types of emerging and remerging infections. In this small country we have outbreak ranging from Marburg, monkey pox, malaria, so these are experiences that shape our response everyday. So I could give you an example that now because of the history of how these infectious diseases, we now have structures like the institution of the institutions like the National Task Force coming down to the District Tsk Force, the District response team, going out to the community level, so using the structures, then political commitments through the office of the president, you remember during covid how the system of structures from the President to the National Task Force going down to the District Task Force and going down to community level. So, our history with some of these infections have put a scenario where we are able to respond to 70%.

Then also the contribution of partners, we have partners like for years I have been able to identify the contribution of organisations like say maybe IDI (Infectious Disease Institute) during the covid response, the contribution of WHO, the contribution of organisations like Baylor, then also another important area that has contributed to response and people don’t pay much attention to it, I think it’s something that starting from 2015-16 when we had the Uganda National Institute of Public Health, having a hemorrhagic training, the first building in epidemiology has really put us somewhere. Now of recent I have seen one, we have one with Busitema University which is also a teaching university. You realize that we have epidemiologists who are looking at these conditions and they are responding to the outbreak as the first responders, guiding the response in all aspects like determining epidemiological linkages, who are we calling to run that, lets say a situation where we have epidemics with different conditions based on the situation of the patient, you realize having the contribution of the fellowship programme, training in epidemiology and capacity, I think there are still gaps in things to do with modeling and handling of data to be able to predict some of these outbreaks help so much during the time when there is an outbreak.

So, me I could point towards our history with infections and then I could point towards the core capacity with training in epidemiology, though the number is still very small but I think it’s contributing a huge pool, then I could point to partners contributions, then I could point to things like let me say from time to time, our health care workers being able to come in and provide critical mass. Specifically that.

**I: What about in terms of community engagement and then some equipment?**

R: Yeah, let me talk about community engagement, one thing I notice with community engagement is that, I had an opportunity to benefit from both sciences, if you realise, community engagement really needs people who understand the community in their own setting, you are no going to go to community, I know, one thing that happens in communities is that people put too much ………on us medical workers, doctors and all that but sometimes when we are communicating to rescue the community we have to really communicate it in a language that they really understand. Now, the challenge that is coming for me in terms of community engagement is that now there is a mixture of fear I the communities, a mix about these infectious diseases which we don’t address. Whereas our primary goal is to respond, but we do always do comprehensively reason being we don’t address all the myth around all these, because sometimes they tell you, its not there, its witchcraft. So how we address that kind of myth, we are not yet ready for that because in Mbale, people whom we want to collect them as contact they will literally run away because there is information that their women want to kill them, there is no empowerment, there is no what. So if we cannot still address that fear in our population or that myth in our population, in terms of community engagement we are not doing much.

The only part I saw was during the time when we were releasing contacts back to the community because for us scientifically, we were sure that these contacts now no longer have any risk of Ebola, we demonstrated certain things where we would hug the respondents to show the community that these people are now free. So, the community do have that little sense of trust in the system that whatever is being done, is being done for their own good, so regarding community engagement I think that is still something that needs to be worked on.

**I: Thank you very much, can you briefly point out if what you see as interventions or actions that are out to contribute to the resilience of the Ugandan Health System, if there are interventions?**

R: Uhmm, the interventions that I would say have contributed to the resilience of the health system is that one thing I could say is partners coordination, am sorry to start from that higher level but partners coordination has always..i had experience to be near Nakasongola and I realized that partners sometimes come in to support the response, when we were in Nakasongola because we had partners like Baylor, WHO, we had other partners that I may not mention, you find that even our health workers were motivated to work because there was sort of facilitation, and I said that because I wanted to say that sometimes we are ready to respond but then we go in because we have not built a system that responds on its own that needs partners to come in with resources and make these response move on. Sometimes some of our health workers relax why am I saying that, when the partners left two months after, the thing went back to the hospital level but they left it, they were not getting any pay from…so that’s a sign that we are not yet at that level of say maybe we can sustain whatever we are doing on our own. So, I think partners coming in has been a very key output in our input in our response as a country.

Then I could say that at the National level political commitment as you know our president is always interested in science and then things that go around, if there is anything that certain population health is someone who is not afraid to rally a political commitment towards what is going on. Then to the Ministry of Health standing strong with us during those times when we respond to some of these but ideally, I can’t mention them explaining that this and this. Then also I have seen issues like multi hazard risk assessment that was done, these are some of the parameters that tell you that we are somehow prepared, and I talked of 70%, there was a multi risk assessment that was done, then there is a contribution of JEE- Joint External Evaluation, these are some of the parameters that have pushed us to a level we have had.

Then also I have seen issues where, you see sometimes in terms of boarder surveillance, you realize that we are very active even from the experience of covid, now when there was Marburg in Tanzania reported Teso Marburg in Tanzania, you realize that the Ministry had to strengthen boarder surveillance by orienting health workers telling them to have high index of suspicion for Marburg giving printing those health workers around us, so I think that’s it from me, but I can’t point to maybe a single, it has been a wild of interventions, some come in you know them some you cant notice depending on the level you work, if it was at the national level, I would have a chance to know, if it was at a regional level, it becomes a little bit wild, you may not notice and sometimes the problem would be that they put it in one region may not have known about that like for instance when you talk to Rwenzori and Tooro region, you realise that if you are to assess the understanding of this disease, you could find health workers having high knowledge or good knowledge of this…..as a health system.

But you go to other regions, depending on the way you are working because I realized that I went there one time, and you could see those case definition in OPD, maternity where there are doctors, it’s a kind of regional imbalance in a way services are given. Interventions are given, you realize some regions might be lacking some of these but at least they help in channeling their support to one side because it’s their project. Back to you.

I: Thank you so much XXXX for that elaboration. With specific examples from Uganda or from other countries, in regard to responding to health system resilience, I mean to responding to infectious diseases, what are some of the key attributes or characteristics that you think make our health system resilient during such crisis like if you would just paint a picture of what a resilient health system should look like?

R: Pardon me, come again?

**I: What am saying, paint for us a picture of a resilience health system especially to infectious diseases outbreak, drawing examples from Uganda but also other countries?**

R: What I would say in that regard is that a resilient health system like I mentioned in the opening session, this is a type of health system that have the capacity or is prepared to or prepared for these outbreaks from the very beginning and as for preparedness I think I talked of staffing, ability of response as they announce the outbreak, we have the system set in place to make sure that we respond to that. So then ability to withstand the shock that comes with this. So, for me why I even said 70% is because of some of these. Now if you realise that Uganda is one of the countries that are very good in ratifying some of the drylands or hemorrhage like the recent one that I know of is this 7-1-7 timelines we are using to report the outbreak, not finding the next level of the health system and then having an appropriate response initiated within 7 days. so am beginning to see it into place that if there is an outbreak in the country, we are trying to make our response and everything within that timeline so that’s one key thing, we are moving toward that though we are not yet there because I remember the rabies outbreak in Buko we missed some of those timelines.

Then there was measles outbreak I think in Kyenjojo, their outbreak a detection was delayed and in terms of response, the notification of the health teams that was done within one day, then response was also delayed because of some factors, so you realise that the view that I see, is that having a coordinated leadership team has supported our response to these outbreaks. We would do well if we had available financing, lets say house financing because these are some of the things that we need to rally the health workers, you need to have the PPEs in place, you need to have…and some of the resilience to (28…..)but I look at it from what type of outbreak are we dealing with, it is something that Uganda has been dealing with in the past, you realise that the response are initiated as quick as possible because we had maybe commodities remaining from the previous outbreaks, saved somewhere or we have experience. But maybe there is something new like we had covid- you realise that we struggle because we don’t have the laboratory capacity to be able to handle some of those things.

One thing I know is that sometimes we don’t do enough laboratory risk assessments, this is also a limitation because our healt workers are dealing with pathogen, they don’t understand much about its biology and what is the level of risk that we are dealing with as health workers, the only think we know is that we have an outbreak in the country, we have to respond but in terms of laboratory leadership, that is an area where …but leadership in terms of coordination we are ok, laboratory leadership in terms of maybe having a lab that can do for us those, I know there is work being done at UVRI and these other core labs like ADI, CPHL and other laboratories that we have currently but still we don’t do enough laboratory research. That is something, and sometimes I worry about our fellows because immediately they say there is an outbreak of A in a certain district, the ministry will react by sending our sending our fellows immediately there, but without a thorough laboratory research actually if in any case, it’s the lab people who are supposed to go there before we send our health workers because for them they are able to understand the level of risk they are dealing with.

So basically for me I would point to things to do with coordination, things to do with having the housework first and by that I mean in terms of case management, we do have a referral approach but in terms of capacity, in terms of epidemiologists, the numbers we have are still not at that high level, if am to maybe guess, I think the core epidemiologists we have in the country might not reach 70 over the years because their numbers started increasing in 2016 when there was a training of these cohort in Uganda.

So, when I look at issues of service delivery, this is an area where if we are responding to outbreaks in a region we realise that these essential service access reduce or service delivery has reduced because it’s the same health worker who is responding the other side, the same health worker who is supposed to treat these people. So basically that.

**I: Ok, thank you so much XXXXX for that good explanation. Let’s talk about still in details the unique attributes of the Ugandan Health System. Now, if you are to look at the structures, our health system structure or the culture and other features in the Ugandan health system, what are those unique attribute that set it apart, I know you had earlier mentioned the exposure in terms of many outbreaks that we have responded to but I would want you to add more if there could be any other unique attribute that has set us apart from other health systems in other countries?**

R: I think for me the unique thing about our health system that places us in a point where we can maybe respond to some of this is, I think its down to the health workers, the reason why am saying that it’s the health workers, I tend to be given a statistic that in this country we train a lot of health workers but our problem has always been to retain some of them in our country like for instance there are more Ugandan health workers in the US or UK than they are here because we don’t have what to pay them but one thing that is clear is that we train a lot of health workers but we fail to absorb them into our system. If we could absorb the people we train from Makerere University, Mbarara, Busitema, Lira, Gulu into our setup and there go and check their track record in some of these countries they are doing very well. So one key area is that we have enough labour force but its marked with regional imbalances. Some of the rural places we don’t get majority of these professionals being absorbed that side because they are required to work in a centralized place like Kampala or major cities in the country but we have the health work force who are willing and ready to help us respond to some of these.

Then another thing is that maybe the coordination at the political will level, I could talk about that because in public health, one key aspect is political will if the leadership of the country is not willing to support or to help in the coordination of response outbreak, there is no way you can come out and stand as a resilient health system who is willing and able to respond adequately.

Then another thing I could say is that, the contribution of partners, I mentioned this earlier, partners like WHO and then these other development partners being around always to support, they have also placed us in a stronger position. Then I could talk about Uganda’s ability to ratify some of these guidelines like for instance International Health Regulation which is a component of our priority disease surveillance in the country.

Then having a good surveillance structure in place, for instance the past surveillance history that we have at health facilities where health workers routinely through their ……likes that for surveillance then the way it is sent every week or maybe every month to the next level of the health system, this tends to be a national level provision of health information although one thing I can tell you is that we don’t have a lot coming out of those data because people start mentioning issues of quality, completeness, but for me my experience with that data is that any data is better than having no data. So why not start from the ones we have then through its analysis, how we can improve, how its quality could be in the future or something like that, because of ignoring that data doesn’t come through is not right, we are not able to do evaluation of our health system and yet this is a key …(silence..).

**I: XXXXXX…hello, I have lost you…**

R: E- component of …..

**I: Ok, so XXXXXXX I want us I think again to look at this other important aspect to discuss about it and that is ..did earlier mention about the role of community engagement in responding to these outbreaks, what are some of other roles of the community towards the resilient health system?**

R: Ok, first of all, thank you so much for that, the role of the community towards the resilient health system, first of all what I would say, the ability of the community to trust the system. If he community that is being served by the system doesn’t trust the system, there is no point in saying that you are resilient at that level. Now, this can be handled in several ways; for instance I can give you an example that when you enter the community you need to enter the community through that structure, by their structure when you have an elder being who has very high influence in the community, when you are going to communicate something to these people, communicate through the person, sometimes our people, like for instance I can give an example during the outbreak in Mbale, we had people who were from the WHO, going to the community to get people whom we are calling contacts, some of them reported that they were being scared. If you don’t come, am from state house I will arrest you, you get those kind of things, so that doesn’t support the resilience of the health system because they are responding because of fear.

But when the community respondents in such a way that they feel part of you, that means you involve them in the process, and how do you do that, communicate these risks clearly using their community key gate keepers, after communicating the risk, then you will begin to see them taking part of or being part of the response at that level. So and aside from that, these days we have the contribution of social media to some of these, before any news is out, already it has been announced on social media, how do you address that and how do you protect a community elderly man who doesn’t know how to tell that this is true, this is wrong. So until we can deal with some of those at the community level, it is very hard to say that we have a community that supports resilient system, I don’t know whether that answers the question but that’s what I can say.

**I: Yes, that answers it thank you very much, now let’s talk about the challenges to health system resilience. Now are there any particular barriers such as inadequate resources, weak leadership or other systematic issues that need to be addressed and if they are there how best do you think these challenges can be overcome at both national and sub national level?**

R: Thank you so much, you have asked a very good question, and I will just try to answer it this way; at the National level, maybe what I can talk about is that we need to start thinking of alternative ways of financing our response not making it donor led or partners led all the time because time will come when we shall have issues. We have these outbreaks and partners have not put money, the people who are responding are not motivated to work. Sometimes we deal with these zero lane conditions and people need to be motivated internally to be able to go and respond to that. So our relying on those funding other than setting aside resources in our national budget to cater for that, we will always make those responding ….I can give you an example during the Ebola outbreak in Gulu, the findings from Prof. Kwale and the rest, tells you that Ebola was already in the community almost three weeks to one month before ………..as respondents, so limited health financing will always make us detect some of these outbreaks late.

Another challenge is that we need to strengthen our surveillance system, though active surveillance is always very expensive and because of that we cannot always mount it from time to time, the existing health system levels goes national and that depends on the quality of the data being collected and then motivating the health workers to be providing those reports.

Then there is one area that came out strongly during these recent outbreaks, was the aspect of mortality surveillance, it’s a subsection within surveillance, it is an area we need to look at because of weak mortality surveillance system in the country, we can nolonger detect outbreaks through that angle of mortality surveillance because you could detect an outbreak because you are seeing classes of death being reported from a given health facility or community using the system but because that reporting is not adequate enough because the people involved in it like for instance you are supposed to do maybe confirming death is supposed to be done by a trained doctor which is not happening in community but maybe some facilities its happening because we have micro doctor who can tell you what is the cause of death. So that is in surveillance.

When you talk about coordination, coordination pillars are there through the national and then regions EOCs that we have. So I think all we need is to make sure that personnel are available in those public as emergency operation centres to make sure that there is coordination throughout some of these. Then also in the community I already mentioned challenges of making the community horn what we are doing as a ministry, that’s an area which is still lacking and we might have to apply what we are calling multi sectoral collaboration where we collaborate with people like the social service, they know how to understand a community better than some of us health workers because for us, if we know that Coartem treats malaria, that’s what we know, but a community, a social worker, would communicate that in a different way to a community member or a household who doesn’t know anything. So the communication bit of that, sometimes the Dr. will tell you the rates as it is, but the community social worker will tell it in a very ….i don’t know.. could say in a very soft way that they would understand. So that’s an area.

Then when we talk about commodities and supplies, in Mbale, I could tell you from my experience that the PPEs that we were using there, the personnel for instance their hygiene, we had requested emergency team but the people we were using at quarantine centre, these were not trained people and there was no initiative whatsoever to train them or equip them, give them a daily briefing that you know what, as you do your work, take note of ABC and D. So it was actually our responsibility as the leaders there to tell some of these people indirectly that the way you are doing this is not right, do it like this, this is supposed to be part of the bigger part of the whole training not an individual initiative from a person. So in terms of challenges, it could rain from one arm to lack of community involvement in terms of things like surveillance and alike. Thank you so much.

**I: Thank you so much for your submission, now this is our last section, we want to discuss some recommendations then we call it a day. Dr. what are some of the strategies or initiatives that you would propose to strengthen the health system and improve resilience to infectious disease shocks? Initiatives such as innovations that you think would do better?**

R: Thank you for the opportunity, you know the recommendation that I would suggest still bow down to what I was saying in adequate finances for instance I would imagine a Uganda where we have like a readily available Emergency Response Department that responds to all sorts of emergency, whether its infectious disease outbreak or its an accident emergency or a maternal health emergency, like creation of a department of that nature that deals with these emergencies and its their role. Remember some of these things become additional responsibility to the health worker, for instance in their day to day treating of patients or delivering of services, if there is an outbreak of any, they need to be called upon to come and respond to that, meaning that we don’t have a designated emergency team involving some of those things. They are supposed to wait for case management in the CTUs that have been created to be able to help there but they are now being called to come and help in contact tracing, come and help in case identification or you know case investigation, that is when you start thinking that there is a problem still.

So I would recommend the creation of an emergency unit department, I have seen this in some other countries, then I was like many things to allow for continuity of essential services we should be embracing things like mobile technology in access of care, very many things are stack like that, some of the things complement the health workers doing their job, so when they engage lets say in responding process some of the in service system is still in position to do that.

Then in terms of community engagement, lets involve the community more in these outbreak response other than tackling them to a position where they are now weak, they are dealing with the risk but the way they are dealing with it is that we are not making them part of it. So, the part of ownership is something that should come in.

Then in terms of surveillance let’s use more of the data that we are collecting through the available surveillance system. This could help us critique like they should be able to tell us when or which outbreak has happened. Then there are techniques like modeling mathematical modeling of infectious diseases, that should tell us how to assimilate some of these outbreaks, these help us adequately prepare for the response and then I would recommend that after every outbreak in a particular community, there is always a team that goes back to assess the impact or response of that outbreak to the health system or to the people in the region, that in itself would really make us a resilient system that is able to prepare, respond and then absorb the shock that comes with such kind of outbreak. For instance, if you are trying to assess what was the impact of the response to the health service delivery to mortality, morbidity in the region, you could find in terms of things, so basically for me that would be. Thank you so much back to you.

**I: Thank you so much XXXX and maybe briefly, talk about still in line with the recommendations, talk about how can VHTs be better supported or empowered to develop a more resilient health system?**

R: For instance, I can give an example, a VHT is our first respondent in the community and these structures, the people I was telling you, they are community gate keepers, when you want to communicate a thing, who is better than a VHT who should help us to do that, it’s the VHT and maybe the LC chairperson and these people have been known to work together in these activities, more especially on us programmers. So majority of training should be given to the village team, I know there is this section of community health work, which they are talking of rolling them, these kind of cadres have helped so much in countries like Ethiopia and other sub-Saharan countries but for us we have VHTs working on voluntary basis, the only thing we can do for them is to make sure that they are empowered, first of all knowledge wise, they are empowered through giving them resources that allow them to work, like for instance there is a system called ECIS, I don’t know if you are familiar with the E- Community Health Improvement System, we are using this to collect data, on certain parameters in the community and it’s the VHT using this, but you realise that some of them don’t have smart phones, some of them normally lack data to send some of these reports. So, if VHTs were to be empowered and given the necessary equipment to use they are the very, like a village would have like 2-3 VHTs depending on the size.

So these are our first respondents here in the village and I have worked with them on research, I have worked with them during outbreak, I have worked with them, so they are committed and people trust them and I think they should be empowered in a certain way to have at the community level. You know in terms of malaria we have been using them a lot, in terms of these test and treat, the referrals to the next level of the health system, we could use VHTs more in terms of community engagement. I think that’s it, thank you so much.

**I: Yes, Dr. are there any successful models from other countries that you think we can adapt in line with recommendations? Successful models in terms of health resilience?**

R: I think that if there are models to be adopted, you would think that the one that works in developed countries but if you look at the level of the health system, its at different kinds of levels, for me when it comes to infectious disease response in the world I still bounce back to our health system as it is somehow more resilient compared to other health systems, because I have never been other countries systems being tested the way ours has been tested on multiple occasions, yes there was covid-19 which tested a health system everywhere but as you can even see from mortality, it was highly worse in developed countries than it was in Uganda or sub Saharan African countries. This was a shock that was coming to every nation, so in terms of the best model for me I still think that if we strengthen our structures from community to sub national level, I think we can go from 70% to something like 90 or even 100% in basic care scenarios. So, I can’t tell you single handed because in Liberia during Ebola we realized that it was our health workers who went to Liberia to provide additional support to their system that side, when they were responding to Ebola you would realise the case return that was registered in those countries during the 2014 Ebola outbreak in Liberia. One thing I can say, if we strengthen our structure, make sure that our financing is enough, then we train our health care workers on what to do, case management, detection, we should be able to achieve some of the studies that will release evidence that will help us to strengthen some of these key areas of our system resilience in Uganda. So thank you very much.

**I: Thank you so much XXXX and I think this brings us to the close of this session. Maybe briefly to let you know that this transcript from this interview will be shared with you to verify the accuracy and then maybe incase we need any clarification we may be reaching out to you through phone. So thank you so much for your time, maybe if there is any other final question that you want us to address before we close.**

R: I don’t think there is any question from me, back to you.

**I: Ok, so I think this brings us to the close, thank you so much for this time, we are really humbled Dr. and safe travels.**

R: Thank you so much
