# Supplementary material for: Resilience as recovery: stakeholder perspectives on Uganda's health system response to infectious disease shocks
Source: Front Health Serv. 2026 Jul 22;6:1888814. doi: 10.3389/frhs.2026.1888814 (PMC13437620; doi:10.3389/frhs.2026.1888814)
Supplement: Supplementary file 2 [file Datasheet2.zip › KII06.docx]

**Transcript for a key informant interview**

**I:** Thank you so much XXXX and good morning team and XXXXX. My name is XXXX and together with my colleague we will be walking you through this discussion. I am glad you have accepted to be part of this study you are welcome.

**I_1_:** Thank you so much XXX, and thank you XXXX, good morning every one, and good morning XXXX, we are happy that we are going to have this conversation together so I am working with XXX and we will be conducting this interview, XXX will take lead and I will chip in once in a while thank you and over.

**I:** Yes, thank so much XXX, and I think without wasting time we should delve straight into our discussion this morning. Once again XXX my name is XXXX and uuh, we appreciate you being part of this study and maybe before we start. We would like to hear from you like you share with us your experience [*silence*]

**I_1_:** I can’t hear [*silence*]

**R:** …in the country including some other countries that we have supported but aah but to go straight to the subject matter which you have talked about which is systems resilience, my experience in this as I have mentioned I started off working with immunization program and then quickly transitioned to emergency program which has been dealing with mainly outbreaks but also focused on resilience of the systems towards these emergencies and how the systems remain functional after some of these outbreaks and emergencies. And then in the last three (3) years I have actually focused my work on district systems and build district resilience so that districts which are the implementing arms of every health program but also the part of building their systems and that is where my work and responsibility is right now.

So, regarding the Marburg outbreak during my time in the emergency program and again we did learn many experiences with Kween not unique to it but in the context of response so over to you

**I:** And yes, thank you so much XXXXXX, now we would like us to talk about health systems resilience in detail. A number of stakeholders have defined health systems resilience as the ability of a system to withstand stress or crisis. In your own words how would you define health systems resilience? Yes, XXXXXX, hullo, hullo XXXX can you hear me?

**I_1_:** Yes

**I:** Hullo, people, hullo

**R:** Now, I can hear you XXX

**I:** Ooh, you can hear you

**R:** Can you hear me?

**I:** I can hear you XXX

**R:** Sorry I lost you at some point, my network is behaving funny. I gave my background now back to you XXXX and colleague

**I:** XXXX, I was saying that a number of stakeholders have defined health system resilience as the ability of the system to withstand stress or crisis. In your own words how would you define health system resilience?

**R**: Thank you so much, my understanding of health system resilience, is the integral system that applies to an area of an issue is able to I would call it steward and also manage outbreak in a way that leave the system weaker but it leaves the system able to continue to deal with other emergency issues, so in a way it may be aligned to what you said but in detail I just want to say that when an epidemic happens or it is detected in a district, or an area, or a country the system is able, the integral parts of the system is able to deal with that but also continue to thrive and deal with the other issues. And I can always go into detail with what I mean by that, over to you

**I:** Okay, thank you so much XXXX lets talk about the factors contributing to health system resilience. In your view what factors contribute to the ability of the Ugandan Health system at national or sub national level to withstand and recover from the major disruptions. Talk about it in the perspective of leadership, resources, equipment and building blocks of the health systems as well as community engagement

**R:** Okay, thank you so much, definitely for a system for it to work efficiently and needs a very strong coordination and that coordination comes from the leadership both political but also technical leadership. Every outbreak will cut a cross one sector and you will never have an outbreak or emergency that needs only the health sector; it will need ministry of health, ministry of finance, then you will also have political leadership to pull and also work together to have this emergency or outbreak responded to.

The other part as you mentioned is the ability to have finances to respond, every outbreak will need resources from what is routinely available within the system so there is always need to have the resources mobilized and additional resources put towards the response and the continued resilience for that response.

The other area of focus is the ability for the supplies; the drugs to be made available on time and also be adequate; that is also another component that needs to be in place. The other one is the human resource, you always need human resource that are competent in terms of the quality but also quantity because every outbreak or emergency will pull your human resource and stretch them so you need to have an adequate pool so that people don’t burn out and also continue with what we call continuing the essential services in the emergencies or in the outbreaks. So, a resilient health system so as to have these various components well entrenched together in the context of an emergency or an outbreak. Over to you

**I:** Thank you so much XXXXX just still on the factors that contribute to the health system resilience, I would like you to shade more light especially in the recent outbreaks like the COVID-19 and Ebola. And I would like you to throw more light on how the leadership contributed to resilience as a country and maybe how that resources at that time contributed to the health system resilience and maybe how the leaders contributed to the health system resilience in particular using the recent outbreaks.

**R:** Okay number one (1) I would say that if you looked at the leadership, their contribution to the resilience of the system with regards to the Ebola outbreak both in Mubende and recently in Kampala is that we honestly have strong political will and that brings into play the multisectoral nature of handling these outbreaks. The ability to have the Prime Minister, and the other ministers engaged in the response I speak specifically in the previous one in Mubende. This particular one we also had multi ministerial engagement…including for example Kampala City Council and a minister.

And then in the finances I have to say in the negatives, this time round we had a challenge with our finances because of the time at which this outbreak happened and our finances were not adequate to be honest. And then had to use the available finances which by God’s grace at least was able to take us through. But I would really put a note that regarding the resilience on the financials it needs to be improved because I can’t comfortably say that we have a resilient system when it comes to the area around financing.

The other is supplies and drugs during this recent, we had enough supplies and drugs and those that were not available could be procured very quickly with a short turn a round time, I know for a fact that agencies WHO helped in that and we had adequate supplies at our disposal to be able to push through this response.

I also missed out on the last definition of a resilient health system is the community and the community engagement and involvement, we did have a very, very participatory community, responsive community that made the resilience towards these shocks, towards this outbreak very sustainable because they were supporting in containing this outbreak…

**I:** Yes, XXX I have lost you, XXX hello, XXXX can you hear me?

**I_1_:** Yes, I can hear you XXX but I can’t hear him as well

**R:** XXXX, did I miss something out?

**I:** Yeah, you dropped off at some point

**R:** Ooh sorry, at what point did I drop off if you can recall

**I:** You were expounding on the community involvement

**R:** Yes, I was mentioning that we have a very good community engagement that contributes to the resilient system because with all honesty I have had to go to different countries, I think the Ugandan community and the way they fully engage to participating in outbreaks and emergencies really ensures sustained and resilient system that is able to maintain and be able to overcome the shocks. So, I think I had gone through the other areas, the human resources in terms of how they were able to maintain and ensure resilience, we do have adequate human resources specifically in regards to this outbreak so, I think we have most of the components available to say that we have a resilient health system that ahs been able to withstand the shock of the two last outbreaks. Over to you XXXX

**I:** Okay, yes thank so much doctor, still on leadership and governance in regards to contributing to health systems resilience during crisis, doctor, what are some of the weaknesses that you think may contribute to a weaker system in particular areas of human resource, health information systems, medical products like vaccines and technology, financing and service delivery. Hello

**R:** Can you hear me, I am trying to speak but I don’t know whether you can hear me? Can you hear me now?

**I:** Yes, I can hear you

**R**: I was saying that with financing we have a big gap and a big challenge; it is actually a threat because we all know that the US government has been funding quite a big chunk of the health system, its withdrawal has caused a big fundamental shift and gap regarding the available resources that should be needed for a resilient health system so that one is honestly a big gap that we need to see how to address before a future outbreak that may shock us and find us in a weaker place.

The second one that I see that needs also to be continuously be worked on as a gap is the coordination because much as I have said that we have a multisectoral coordination we seem to be losing that ground because in the last outbreak we seemed to have an issue where seems to be a contradiction with regards on how to coordinate a response when it comes to the effects of an outbreak on tourism and trade and I think we need to resolve that contradiction otherwise it will spoil all the gains that country has built in terms of resilience. So, around coordination and leadership a lot of work needs to be done because here is a need to harmonize the expectations of the different stakeholders. I think in the last outbreak we found that the expectations of the other stakeholders in the outbreaks outside of health which tourism and trade overrode the expectations of the health sector that is dangerous in a particular way

When it comes to drugs and supplies we have a bit of a gap although for now it is still being filled by the available partners but I think it is important to stock pile. We are a country that faces outbreaks very often and we can’t talk of the next outbreak as if but when stock piling for example EVD kits need to be stockpiled in most of the regional referral hospitals so that when you get a confirmed outbreak you can have immediate response and not wait for, for example the national level coming to bring in from the international supplies. So, those are the few gaps I would raise regarding the resilient health system.

**I:** Thank you so much XXXX still on that I would want you to shade more light on how you view community engagement as a factor in this resilience

**R:** Yeah, I think for me If asked me the community is a very, very integral and a very important part of a resilient systems because we know that any outbreak or epidemic starts from the community and honestly only ends with the community. It doesn’t matter how much of the other components we have talked about for example financial resources, communities can decide to hide and not be responsive to the messages that are given in an outbreak setting and until they decide to cooperate and respond you can’t do much because even if you used force it will be difficult to know where every person is and who is hiding a sick person, so I can reiterate that community participation and full sensitization and all that goes with it; mobilization is very crucial to a resilient health system because they are the first to detect they are the first to be suspicious and say we have many deaths happening in the community, we need to inform the people that are responsible. They are the ones when the messages for control, seeking health services and all that if they respond early you have a quick turn around in containing the outbreak and if they are slow, you have a slow turn around to outbreak and the outbreak affects many so I can’t have enough words to say that community engagement and its involvement and the community is very, very crucial.

**I:** Yes, thank so much XXXX, lets talk about the interventions that are contributing to the health systems resilience in Uganda. XXXX, what do you see as the interventions or actions that have contributed to the development of the Uganda’s health system.

**R:** Well when you talk about the interventions and ensuring that the structures of the system are able to raise up and do what they are supposed to do. So, the intervention that I can speak of directly are interventions to do with issues around the capacity of the health system in terms of response and then we have interventions in terms of social mobilization and health promotion and community engagement. So, sending out the messages and appeal to the community to engage and participate in the system that is operational and it is so effective to pick, detect and to be able to transfer these suspects or contacts to where they are. So, all these interventions that go into a resilient health system. Over to you XXXX

**I:** Yes, doctor, yes XXXXXX can you hear me, hullo

**R:** Over to you

**I:** I lost you towards the end of your submission doctor

**R:** I can repeat but can you hear me now

**I:** I can hear you but the last

**R:** Yeah, so I was mentioning that we have interventions on promotion, we have interventions around case management and how to treat and mange patients. We have interventions around the lab and how testing is done and delivery of specimen when it reaches the lab, so those were the interventions in my earlier submission

**I:** Thank you so much XXXX, still on interventions are there any commo policies that have had an impact in strengthening health system resilience apart from what you have mentioned now some policies if any?

**R:** Come again XXXX, I didn’t hear that

**I:** Are there some policies that have had a noticeable impact in strengthening the health system resilience if any

**R:** Yes, quite a number of policies, I think you know that the immunization act which helps regarding how populations respond to immunization I think that is a very effective over ruling arm in terms of the policy area that helps to be able to; for example, in Ebola now we do have interventions that require vaccination and that comes in in terms of how a response entail. We have quite a number of policies and guidance around case management and these for example the health care protection and guidance around IPC all these are also policies and guidance that help in the resilience because we know that for example infection prevention and control are very key in ensuring that our health workers who are the frontline of these outbreaks are kept safe and also the population and we do have policies and guidelines within the case management pillars and that also work towards ensuring a resilient health system.

We also do have the policies and guidelines around international health regulations (IHR) but also which have been domesticated around country level with various policies that talk about what surveillance is, how it is undertaken, and the obligation of the key stakeholders. All these are very effective and efficient in mobilizing the systems and strengthening its resilience. Over to you

**I:** Wow, thank you so much doctor, now we like to discuss the characteristics of a resilient health system. XXXX, can you paint for us a picture of a resilient health system especially to infectious disease outbreaks. You could go ahead and discuss some of the attributes and characteristics that make a resilient health system

**R**: Okay, I told you earlier that I will expound about the health system in terms of its resilience; you know many are times that health system is able to quickly or at least be able too work within the existing health system but for a resilient health system it should be having the efficiencies to mobilize and to be able to respond but also remain in a state that it is able to continue to deliver essential health services, so if you look at what you need, which are our health workers. If you have an emergency and allow health workers to be able to respond to that emergency or outbreak in whatever duration it takes but for it to work, every other service like HIV services stop. So, if you put all your human resources to responding to an Ebola emergency services then your system is not resilient. And you are unable to continue to provide and to supply other services that are needed then your system is not resilient. Its characteristics should be that it is able to rise and respond to an emergency at the same time continue to provide adequate services that are needed and at the same time be able to come out that shock and proceed normatively in the regular and routine services. So, I have used the example of human resource but the same applies to the finances, if you have a system that pulls all its resources and puts them all to emergency or outbreak but leave a big gap in the provision of routine services then that is not a characteristic of a resilient health system. It should be able to pull the resources to emergency or outbreak but remain with adequate resources to continue to run your routine services and also after the end of this outbreak or emergency to be able to continue to also provide your routine services, So, these are some of the few examples that I can pick out of what should be the characteristics of a resilient health system.

**I:** Thank so much doctor, in regard to the Ugandan context I know you have shared some of those characteristics, could you throw more light on how the structure, the culture and other features of the Ugandan health system set it apart from the other health systems when it comes to resilience

**R:** Yeah, number one (1), I just want to say that I repeat that our community is very unique. I have been part of the response in DRC, I was part of the response in West Africa and I can say that the communities were not as, at least then they were not as responsive, they were not as appreciative, they were not as collaborative as I find in the Ugandan community during emergencies and outbreak so this is one thing that I would say in the Ugandan context that set apart the response. And…

**I:** I think I have lost you, hello doctor

**R:** Yes, sorry, sorry I think I got a problem with my network. I was saying the community as a component of the health system, the Ugandan one is very, very unique. The second one that I wanted to raise the uniqueness is on the leadership and coordination, I think right from the president whom we have seen taking the lead in mobilizing the community and mobilizing the entire population around emergencies and outbreaks is not the same in other countries, where the top leader becomes an integral and pertinent part of the response and that is very unique in Uganda.

The same we can talk of in terms of our leadership; I have also been in responses where minster for health is not very much at the forefront but in Uganda we have seen the uniqueness that in outbreaks for example during the recent outbreak in Mbale, Kampala and also the one in Mubende, we had the minister paying regular visits to the hotspots just to encourage health workers, to motivate them. So, coordination and leadership in Uganda is very unique in that context

We also have around the drugs and supplies, the national medical stores which I can say is the key institution in Africa that is really above question and it is able to mobilize drugs and supplies to different parts of the country within a very short period of time because of their robustness and uniqueness and agility and their resources in terms of their vehicles and all that is also unique to Uganda. So, those are the three main examples that I would bring out about the uniqueness of Uganda resilient health system, over to you

**I:** Thank you so much, maybe before we proceed you did mention that the Ugandan community is very unique but then you didn’t stress or I missed out on the point that makes it so unique. What are some of those things that make it so unique; the Ugandan community from other communities in other countries?

**R:** The Ugandan community believes in the health systems; believes in the messaging and the actions that they need to do when you talk about other countries when people are given messaging, I will just use an example of Ebola which we have had very many times, you don’t need to carry out practices you know that we do carry out on bodies traditional practices that we do carry out on bodies and all those other assemblies during burial you know such things. The other communities don’t appreciate that and don’t follow the guidance of the health workers and the health system and that definitely brings a big challenge. You know as an example of the West African outbreak that what took that outbreak long was because of the lack of collaboration by the community regarding what needed to be done. I have seen the same in DRC, their population even though they have been exposed to many outbreaks of Ebola they don’t appreciate and respond to the messaging of the health system and the health workers give them on what their role is in preventing, their role in containing, their role in being able to, respond to the outbreak and manage the outbreak, they don’t usually respond the way the Ugandan population does, thank you

**I:** Thank you so much doctors, still on the role of the community in health systems resilience, earlier on you did mention community participation and you have just explained more on community adhering to SOPs that is given out by the ministry. Are there any other roles the community plays towards the resilience health systems in Uganda, if any to build on what you had mentioned prior?

**R**: Well, I mean I think I have mentioned them but just to repeat myself, the communities number one (2) are our first source of information, because when you look at community-based disease surveillance we can have diseases being hidden in the community and they are never picked because they don’t necessarily come to the health facilities. So, once you have a community-based disease surveillance another type of community way of information picking you can always rely on that because they will tell you that people are still in the community and they haven’t gone to the health facilities but they are presenting with symptoms silently which we can pick out and later confirm it could be some very dangerous pathogens, already existing

Number two (2) we have them as a very good action arm of the response as I have mentioned, when messages come out about how to contain the response they are major players in terms of what to do.

They are also a good source of sharing and giving out the information around what the disease entails and contains so that people can also start learning more about that so they become a part of the system in terms of educating and ensuring that the information the health workers have for example we usually have the community case definitions which we give out and then the community starts spreading them around to their families and before you know you have an entire population or community that has an awareness about the signs and the symptoms of the disease and all that. Yeah, jut to reiterate some of that, over.

**I:** Yes, thank you so much doctor, earlier on you mentioned some of the key challenges to health systems resilience and I mean still if you have more. What challenges or obstacles affect the heaths systems ability to withstand and recover from the infectious disease shocks

**R:** Yeah, I have mentioned them for you in particular and I think financing is one at the very top, we have our health work force that is also becoming another big gap because when it comes to the quality; we have may have the numbers but the quality is quite poor or at times depending on how they have been exposed to disease outbreaks, I think in this last outbreak we saw that we missed out what we call the index case because I think he went to three or four facilities and he was not picked and that was a gap regarding the knowledge but also the quality. So, that is some of the challenges. We definitely have a challenge like I mentioned around stocks and the supplies of drugs and what is needed. Honestly these are some of the gaps.

I think on the community aspect, there is also an issue that is coming around some misinformation especially regarding vaccination as I mentioned during COVID that made the community very suspicious around some of the messaging and so slightly needing to have additional intentional interventions to keep the community on our side so that I future we don’t have them doubting very serious messaging that the government is sending out. Over to you

**I:** Yes, doctor, hello

**R:** Yeah, I gave input and said over to you

**I:** Thank you so much how do you these challenges can be overcome at both national and local levels?

**R**: Yeah, the challenges can be overcome by being intentional for example in resource mobilization, we need to be strategic in regards to how do we resource mobilize in the current context, I feel that we need to bring on board more of the private sector, we saw that during COVID that the private sector is very much interested in being a part of a resilient system because at the end of the day emergencies or all outbreaks will affect them. So, we need to expand our resource mobilization and resource capability beyond the traditional government support and partner support and we start looking at private and other non-state actor’s involvement in resource mobilization.

We also need to look at continuous engagement with the community even in times when there is no outbreak to answer questions around the issues they raised several times and are not answered. When we don’t answer their issues and we just speak above their heads regarding what we know and what we are confident about then their suspicion remains and when the time comes they may not be collaborative so we have to continuously engage the communities, not to just speak at them or over them but to hear what their concerns are and how to address them and then have them confidently back with us.

**I:** Yes, doctor

**R:** We definitely have to look at how revitalized intensive trainings also can be undertaken. So, those are some of the key ways in which we can try to address the gaps. Over to you XXXX

**I:** Thank you so much, doctor, there are a number of stakeholders that we work with within the health system; talk about the government, international community, Civil Society and Private sector. How can these partners work together to address most of the major challenges and what kind of partnerships and collaborations do you think are necessary to address most of the challenges effectively?

**R:** Yeah, I think one we need is to strengthen what we call private public partnerships (PPPs) because as I have mentioned we need to have the private sector more engaged and fully participatory in our health activities so formal engagement of the private sector is very key and I think what we can do differently is that public sector or the leadership and coordination should reach out to the private sector. They will not come by themselves, they need to be reached out for them to come on board.

I think we need to relook at our financing system and financing structures, I know for example that there have been financing dialogues, finance policy shifts and policy papers and these should move from just papers to actual implementation because we do have a very, very big gap that is scary around the financing for the health sector which should obviously be addressed sooner than later. And then as I mentioned earlier we need to bring on board the media houses, and other media interventions to kick off further engagement of communities because I believe that it is better if the private sector again would take the lead in trying to re-engage the community definitely with the support of the technical personnel from the Ministry and other partners to come on board in addressing some of the concerns of the community.

And then you talked about how the partners and all the other stakeholders can be engaged; the partners, the local development partners, we have groups that are specific to areas of concern like the global health security group, so all these should be reactivated because I know that some of them are not functional but these should be reactivated by government so that all of them are working, all of them are talking together and all of them sharing information for action on how best to change the situation, Over to you

**I:** Okay, thank you so much doctor, so lets talk about some of the recommendations what strategies or initiatives would you propose to strengthen the health system and improve resilience to infectious diseases shocks?

**R:** Well, some of the strategies I would recommend or ways that I would recommend is the strengthening of the PPPs, the public private partnerships, yeah, they exist in theory but they need to be reactivated, re-engaged and be fully operationalized. We can no longer do this, only depending on the government and partners, we now need a broad engagement with the private sector in all its forms whether it is private business, whether it is private health or whatever. All these must come around the same table, health doesn’t have boundaries, it doesn’t have shades, it doesn’t have denominations, health affects every one if we got an outbreak in Kampala, it doesn’t know whether this one works for Makerere, works for the private sector or works for WHO; we are all affected equally so we need to start sending out some of these key messages. So, I recommend re-engagement, re focusing and intentional operationalization of the PPP which is the public private partnerships,

I also strongly recommend that the financial, the financing groups that are already in place should be reenergized to come up with decisive recommendations around how best to fund the health system. I think we do a lot of talking and this talking comes up with very flowered and good outcomes but I think moving this to the next stage of actual implementation should be the focus of our next agendas around financing and sustainable financing. I know I was part of the team and group that participated in the sustainable financing dialogue in Munyonyo two weeks ago, I pray that and many others, we would move away from just talking to really putting the key recommendations into action and it will be disaster waiting to happen if we don’t fill the void in the resilient health system

The third one is to recommend ongoing engagements with the communities to listen to them and answer them not to talk to them, not to talk over them but intentionally engagements with the communities that listen to their concerns, that listens to their doubts and fears and answers them as appropriately, thanks and over to you XXXX.

**I:** Thanks, so much XXXXXX, let’s talk about empowerment of the VHTs, doctor how can VHTs be better supported or empowered to develop a more resilient health system in Uganda?

**R**: Yeah, I think the ministry structure is now in place, its quote and quote it is supposed to be operational, I think what needs to be done now is to make sure we don’t support it in an adhoc way. I feel that we need to support it in a comprehensive manner where all the stakeholders can channel that support through one entity whether it is through Ministry of Health so that we have a fair distribution and a fair support and a fair engagement throughout the country one because I think the gap that we are seeing with the VHTs is the lack of uniformity in their knowledge, their abilities, their skills and their facilitation and that is not going to help us where we are having strong VHTs and weak VHTs. So, I feel that the VHTs are already established, they are already in place, we need to approach them in a holistic way, to support them in a holistic way and facilitate them in a holistic way. I think that will help us move the VHTs which is a very, very fundamental and crucial pillar in resilient systems to move forward and to be able to fairly able in all parts of the country to work in a resilient health system, thank you. Over to you XXXX

**I:** Thank so much doctor, still on VHTs are there particular trainings, resources or policy changes that would improve on the resilience of the VHTs?

**R**: Well, there are resources but like I have mentioned at times resources are geared towards specific areas for example I know that the Korean NGO, KoFIH had some resources which they had focused towards the Masaka, Buganda area that was substantial they did help with giving facilitation to the VHTs, in terms of bicycles and all that. I know that there was also an additional support that went towards the East which I remember well but again like I said what we need now is not to continue doing it in bits and pieces; whether it sits at the government level and all the resources are channeled or it sits somewhere else and the resources are actually channeled there, we need to look at the uniformity across the VHT strengthening and support

**I:**Ooh XXX, can you hear me I lost you at a certain point

**R:** Yes, I can hear you

**I:** I lost you in the last sentence

**R:** I mentioned that we need to have a holistic way of supporting the VHTs and you has asked me some of the identified gaps and I said that we have patchy support of the NGOs I know for a fact that some NGOs like KOFI, Korea were supporting the VHTs in some parts of the country but that, that is not adequate we need to see how to support them holistically, or provide some uniformity in their support if we are giving them bicycles then lets know that all the VHTs in Uganda have bicycles. If we are giving them tools that they need to capture information whether they are kits or anything, we know that the entire in Uganda have those kits and not to say that the ones in the East have. So, that way of looking at the entire VHT structure across needs to be emphasized and actioned. Over to you.

**I:** Thank you so much doctor, now let’s talk about the different models of the health system strengthening, are there any successful models in other countries or other contexts that you think could be adapted to the Ugandan context to enhance health systems resilience?

**R:** think that the models that have been used in other countries is exactly what Uganda is using, so am not privy to a unique model that Uganda could pick up but may be that is something that we focus our intention to do but from where I sit it is one and the same model. Over to you, XXXX

**I:** Silence

**R:** Have I lost XXXX, can the others hear me?

**I:** Yes, we can hear you doctor, you did mention, you had earlier mentioned that you don’t see a any model but you recommend the current model. Why do you think that the current model is the one that is effective?

**R:** I didn’t say that I recommend model, I said that from where I sit and the exposure I have had with the countries is that they are using the same model as we are using. So, I didn’t necessarily say that the model we are using s superior or better than the others, I said that where I sit and I may not be advantaged to or gone into areas that have other models but the one that Uganda is using is the one that…

**I:** Ooh, hello

**R:** Yes, I can hear you

**I:** I lost you from the point where you were saying that the one that Uganda is using is the one that, I lost you from there

**R:** Okay, I said that I am not necessarily recommending the one of Uganda but what I know that given my exposure which is limited is that other countries are using the same model, over to you.

**I:** Okay, thanks so much doctor and this bring us to the end of this interview, maybe could you be having any other issue which you want to bring to our attention that we have not discussed about

**R:** No, not an issue, I just wanted to hear again the title of this PhD or the topic for the PhD; what is the topic ad what are the objectives

**I:** Yes, thank you so much And I think XXXXXX is on, XXXXX would you want to take up that?

**I_2_:** Yes, yes, thank you XXXX and thank you so, so much XXXXXX for giving us the wonderful insights, I understand that you have been in this space for some time and surely a lot of what you have given is very, very useful. So, the title of this, uh I just want to et something here like so, the title of our PhD, my PhD is “Health Systems Resilience in the Context of Infectious Disease outbreaks; Exploring Concepts and Experiences, Frameworks and Tools in Uganda” So, that is the title. And we had four objectives and our first objective is to explore and really that is what we are working on right now; To explore the understanding of health systems from, it has two parts; the first part is to explore it from a very wide angle in the African context; for that we conducted the systematic review and then from the Ugandan context where we re doing key informant interviews and like I mentioned earlier we will also reach out to regions and districts but also from the implementers themselves. Then uh uh, our second (2) objective is to critically address the IHR and DHIS tool for their appropriateness to measure resilience to disease outbreak in Uganda and based on the results adapt on WHO health system indicators and contextualize it to be used in Uganda.

Then our objective number three (3) is to assess the resilience of the health systems in Kasandha and Mubende districts during response to Ebola viral disease outbreak in 2022. So, this is going to be a retrospective application of the contextualized tool and the key here is that we are going to use the tool that we will develop, and the two that is contextualized from the WHO and the priori tool and apply it to Kasadha and Mubende based on what they did in 2022 and figure out whether they had elements of resilience and if so to what extent, we are measuring. And then our fourth (4) objective is to identify the enablers and barriers to the implementation of the interventions to strengthen the resilience of the health system in the two districts and for that we are going to implement an implementation research and we are using the consolidated framework in the implementation research to identify the enablers and barriers. So, these are our four objectives for the PhD, over to you XXXX

**R**: Say the title again

**I_2_**: Ooh, the title is “Health Systems Resilience in the Context of Infectious Disease Outbreaks; exploring concepts, and experiences, frameworks and Tools in Uganda”

**R:** Okay

**I_2_:** Yeah, and the researcher is myself

**R:** Okay, no problem, I just wanted to know

**I_2_:** Just in case you want anything further you can ask me and then I share some of the tools that I have and my proposal I don’t mind sharing it

**R:** Okay, thank so much, thank you really for this opportunity

**I_2_:** We truly appreciate your time, and beyond his I will surely reach out to you

**R:** Okay, thanks

**I:** Thank you very much XXXXXX, XXXXXXX had her hand up

**I_2_:** XXXXXXX please go on before we can close this

**I_1_:** Sorry, to come at the end uh-uh, I was just interested in XXXXXX commenting about probably Uganda’s experience or exposure to the various outbreaks, how that could be a unique attribute that contribute to the Uganda’s health systems resilience. If you have any comments on that XXXXXX I would be happy to hear thank you and over to you

**R:** Yeah, it is a good one and honestly quote and quote uniqueness of Uganda to so many outbreaks has ended up being more of an advantage because the more you continuously respond and the successfully contained outbreaks the more you will find that you are building within your system the resilience that it needs because with the many shocks the system naturally and as a matter of defending itself develops the uniqueness’s of handling. So, the Ugandan health system has had many outbreaks I think the last Ebola one was the 8^th^ and because of that we have many people that have been a part of these responses, so when it comes to the human resources for health you are not talking from a point of inadequate who have never handled an outbreak. We have many people across the country 8that have been engaged in so many different ways.

The same with the people to mobilize resources we have people who have been part of the supply chain and therefore they are able to handle the request for the supplies and drugs very effectively and very efficiently. We also have a system as I have seen for example the national medical system that knows the challenges but also the ability to handle what they call routine and emergencies. So, during emergencies they quickly move into the emergency mode and can deploy within a short turn around time of small as 1hour requests they are able to respond.

Definitely the communities have been engaged in different outbreaks and so when you come and tell them about Ebola for example they don’t question it and say this cannot be, but they know that they have handled it before, they have been part of it before, they know the SOPs of Ebola and when you say that you don’t need to bury your person in the normal way and using the traditional and cultural means they respond to that because they have some experiences where people have unearthed bodies and exposed themselves and died in Kasandha and we know that quite a number of people that exhumed that body and touched it actually ended up dying and that was a nasty experience which people know about and wouldn’t want… (*network issues*)

**I:** Ooh doctor, we are not hearing you

**I_1_:** Doctor we can’t hear you

**End**
