# Supplementary material for: Resilience as recovery: stakeholder perspectives on Uganda's health system response to infectious disease shocks
Source: Front Health Serv. 2026 Jul 22;6:1888814. doi: 10.3389/frhs.2026.1888814 (PMC13437620; doi:10.3389/frhs.2026.1888814)
Supplement: Supplementary file 2 [file Datasheet2.zip › KII07.docx]

**TRANSCRIPTION FOR KEY INFORMANT INTERVIEW.**

**I: Once again thank you so much for accepting to be part of this interview. Before we can start kindly share your view related to the resilience of health system related to the Marburg Virus Disease outbreak in Kween District in 2017?**

**R.** Health system resilience is when the human resource is on point in terms of ensuring that we have the human resource that is available and capable of responding. So as MoH, I think the system is resilient in a number of aspects. The human resource is tested and tried, there has been outbreaks for quit sometimes and Marburg, this Marburg was not the outbreak that happened in Uganda. I think we have had about 3^rd^ Murburg outbreaks. This was the 3^rd^, and ah, based on the previous experience, the HR at Ministry’s response mechanism, it has built some level of resilience in terms of how to respond, they have people who have responded to Murburg before.

So, mobilizing the team to actually respond is kind of a conclusion in terms of who is available and who can handle this and that and there is no doubt about their competence and resilience in trying to ably respond to an outbreak like Murburg. Ah, building on all the history of Uganda’s threats that come from viral hemorrhagic fever like Ebola and Murburg, I think the system. The MoH system or health system in Uganda *[Network breaks, in audible]*

**I:** **Hello XXXXXX**

**R:** *[inadible]*

**I:** **We have lost him. *[note taker]***

**I:** **XXXXX?**

**R:** Yes XXX,

**I:** **Ahn, I lost you sometime back when you were talking about the MoH systems. I think there was an interference on your side and we were not getting what you were saying.**

**R:** I think I receive a call but, I think what I was saying is that over time, I think MoH has built not only the system but the human resource and the capabilities to be resilient to respond to any viral hemorrhagic fevers like Marburg over the years.

**I:** **Hmmnn**

**R:** Yeah

**I:** **OK. So, you have talked about resilience, resilience, So, we would like to understand. How do you understand the term health systems resilience or how would you define it in your own terms.**

**R:** Resilience is basically means the MOH having a mechanism in place to withstand and push through an emergency response against the viral hemorrhagic fevers like Marburg from the time of detection, being able to detect the virus and ensure that the appropriate teams and resources are available to respond and control the outbreak. Ahh in as quickly as possible. So, me if a system can do that, like MoH can or has, if like the MoH health system is resilient. So, resilience is withstanding the response. Standing up with the response to Marburg outbreak, detection, and control of the outbreak and even declaration that actually we are Murburg free.

**I:** **OK, so, how different will that be from emergency response?**

**R:** [Sighs] Emergency response is… is any, anytime you actually have to respond, you can respond but are you resilient enough to get to the end and you are to be resilient, you need to stand up to the end and control.

**I:** **OK**

**R:** So, Emergency response can fail, you go and fail but when you are resilient you stand and I think at the end of the day you succeed with the control of the outbreak.

**I:** **OK. Thank you very much, ah, so, in your own view what are some of the factors that contribute to the ability of Uganda’s health system to withstand and recover from the major disruptions or outbreaks?**

**R:** I think ah.. the main factor is first of all there is a lot of good will from the political good will from MoH and government basically. They kind of have a clear understanding of why they should be a quick and fully supported response when there is Ebola or Murburg outbreak.

**I:** **Hmmn**

**R:** So there is a lot of government support that goes into it and understanding that this is something that we are supposed to deal with.

The second is that, we have quite a bit of support, actually a lot of support from international organizations, partners, ah WHO, CDC. They actually come in handy to support MoH and ensure that they are on standby and have the resources to respond resiliently. There is a lot of trainings, human resource capacity that is available in of people who can actually be called on to respond is available and tested and tried. So, there is a lot of human resource that has experience to actually respond to outbreaks like these.

**I:** **Hmm**

**R:** And also, there is a mechanism. A decentralized mechanism, ah- districts, regional referrals, regional EoC, and national, so that funnel like system where National goes to regional EoC’s, goes to regional referrals and to the district up to the VHT, that system has been tested and actually is very, very dependable when you have , when it comes to things like VHM, because when you do a VHM, you need to do active search and the VHT’s are the people who actually help you to do the search. So, that system being in place is very important for you to actually run a resilient emergency response.

**I:** **Hmmnn. OK, I am most interested in following up some of the answers that you gave. Ah, looking at the VHTs, how further can you comment on the involvement of the community in the response in the health system resilience during outbreaks?**

**R:** So, part of the resilience that we are talking about for the health systems is, I think almost 70% is built by the community.

**I:** **Hmmm**

**R:** It the community that plays the biggest role in controlling any of these emergencies that come around. Having the community on your side, is so important because they the people actually who are diseased. And because they are diseased, they are actually the people who can help you control, because they will help you identify, they will help you control this. So, VHTs who are part of the community are so important, because they are the people on the ground. Ah at MoH, there is no way you can say that you are going to Village X and do detection or identification people who are sick. You have to go to the community and use the VHTs to help you, tell you where they have a problem or not, and where there is a problem, they take you to where the problem is and that is how you start. So, having them on board as part of the response mechanism is part of the big resilient system that we are having because they play a very, very important role in the community.

**I:** **Ok, you also talked about, ahm having government support from political will on your side. Comment on the leadership on the side of the MoH, regarding resilience.**

**R:** So, the MoH.. ah.. already has a mechanism in place formation of the DHF, if it is Murburg or Ebola, MoH already knows how to respond. Top management chairs meetings, they actually declare the outbreak and as soon as they declare it, they are daily meetings, if it is Murburg or Ebola, there daily meetings by top management shared by the Minister of Health every day with updates straight from the field. So, the preparations of MoH when it a VHF like Murburg or Ebola are undoubtable. As soon as they declare that there is an outbreak, the mechanism just jumps into play. The minster chairs the meetings, and they are daily meetings until the ministry feels like the OK, the emanate danger is gone and then we can now maybe re-schedule and have staggered meeting, so, that, that preparation, that mechanism for them to be place immediately when there is a problem, is basically what I am saying that there is a lot of support from MoH because they are there to coordinate and ensure that there are resources available for the response teams to be able to deal with the issues in the field.

**I:** **Thank you very much, what about any equipment that is used during such outbreaks, do we have any? Could you comment on that?**

**R:** I think the equipment’s for such outbreaks like Marburg; it is mainly laboratory.

**I:** **Ok**

**R:** Laboratories have been put in place. We have very good laboratories, right now when it is VHF we think we have like 3 or 4 Labs that can actually help confirm or test for a VHF, UVRI, CPHL. So, in terms of LAB I think we are very well equipped there is a whole sample transportation mechanism that the MoH has that runs with or without an emergency. The other big part is human resource, which is basically. All you need is human resource and having human resource with PPE. If PPE is there for Murburg, that is OK. The other part that is require for equipment’s is case management. Set up the treatment unit and be available and the last part is basically vehicles, just have vehicles moving around. So, all these things are actually in place and MoH can these in time.

**I:** **Ok, thank you very much. Ah then you also talked about us receiving support from the UN during such outbreaks. Kindly throw more light on what kind of support that you receive from the UN agencies or other partnerships.**

**R:** I think the partners provide support in all the spheres that we are dealing with, mainly resources, they provide the money, the resources. To pay the people, to procure equipment but they also support us with human resource, sometimes they send us epidemiologists, to actually help to support the system when the issues for Murburg, usually, WHO sends in some support, CDC sends support, the resources to help us with fuels, vehicles, pay people, buy PPE’s all these they chip in through their support.

**I:** **OK, ah thank you very much, so, ahm what do see as the interventions or the actions that have been taken, that have contributed to the resilience of our health system in Uganda. What are those interventions or what are those actions the country has taken to contribute to the resilience that we have now?**

**R:** For me I think it is preparedness, the ministry is always in preparedness mode, whenever there no imminent danger, they are prepared, they are preparing the human resource, prepositioning supplies, they are always training. We have a MoH full training, that Public health fellowship program that is run under the MoH is basically there to actually produce epidemiologists who are resilient and can actually support the response any time.

Ahh still the MoH also, I think it is preparedness and the trainings. Those are the things that I think MoH does as interventions before any outbreak happens, even the labs, they keep supporting the LABs by training people in the LABS. So, it is the trainings of epidemiologists and ensuring that we are in the preparedness mode, of course with our early warning systems that is in place to ensure that we can detect any of these VHFs.

**I:** **Hmmnn, OK. Thank you very much. So, now we would like you to paint for us a picture of a resilient health system, especially looking at the infectious disease outbreaks. What are some of the attributes or characteristics that make a resilient health system?**

**R:** Attributes of a resilient health system?

**I:** **Yes, or characteristics of a resilient health system when you talking about a resilient health system, what are those characteristics it should have.**

**R:** I feel like these are the things that I have talked about.

**I:** **You talked about the interventions that have been taken and you said there is preparedness and then there is the trainings. But you are talking about a resilient health system, whare those attributes it should have or what are its characteristics, is it the same?**

**R:** The human resource, it should be equipped with the human resource. The main issue about resilience and about the people. It is the people who are resilient. The people who make the system are the people who actual make the system resilient.

**I: Hmmnn.**

**R:** So, if you have the human resource that is fully trained and knowledgeable, and they have experience in responding, I don’t see how the system is not going to be resilient. Because it is the people who are running the system who are supposed to be resilient and that makes the system resilient. So, it is mainly the human resource, and that is why I am saying we are always preparing by training people, prepositioning supplies. Ahh the public health fellow, producing epidemiologists, running the surveillance system that does the detection. These are the main attributes that I think make the health system resilient. The people actually who run that system are the people who are resilient and having them trained and ready to actually respond is basically for me what I call the resilient system.

**I: OK. Still on that point of the resilient system. Any unique attributes that make the Ugandan Health system resilient?**

**R:** The uniqueness here for me is two folds. One, is experience, we have a lot of experienced people in that system who have responded to not only one Marburg outbreak, two have not responded one Ebola outbreak, four, five, six. So, those people already know. So, the main part that makes our system resilient is experience, the exposure. The exposure that our human resource has is top notch.

**I:** **OK**

**R:** They have done it, they have done it before and they will do it again, and that is basically what defines their resilience.

**I:** **Hmmn, Ok. Ahh, earlier on you had talked about the role of the community towards a resilient heath system, and you had said they are the people who are diseased, they are the people tell us where the conditions are, and they help us ah. Ah.. intervene, or, or work towards the outbreak. Now, I am just wondering if there is any other role that the community plays towards resilient health systems.**

**R:** I think the main role for me that the community can play further is ahhh, is, the [sighs] is the awareness level. For as long as the community is in sync with what you are trying to do, then your system will appear or it will remain resilient. So, they are the diseased people, they are the ones that help to detect the community has sick people and they accept that the people who have come to respond have actually come to help them. So, there is that community acceptance part for as long as you have a good will with the community, then you are going to deal with the issue quickly. So, for me that the other aspect that the community has. If they are resistant and not welcoming then however resilient that your system is, it gonna fail.

**I:** **OK, alright. Thank you very much. Ah, so despite having our resilient health system, Ahm are there any challenges or obstacles that we have faced?**

**R:** Yes, challenges are always there. As long as you have painted a good picture of everything, sometimes we are short of human resource.

**I:** **Oh! OK. Ahm,**

**R:** Yeah, it not like we are, sometimes the human resource is short, sometimes it can be short of the expertise. Sometimes, we actually have logistics issues sometimes that come. Vehicles, sometimes are not there, we have challenges with resources, payments of people, the human resource takes sometime when they are not paid on time so the motivation is a challenge. Ahh, so, ahh, however good the system is and I think I should say that our system is really good and resilient. It doesn’t come without those small, small challenges of payments, delays in Lab supplies, delays in return of Lab results, shortage of epidemiologists, so, all these basically happens in any system.

**I:** **Ok, so how have we overcome this over time.**

**R:** *[they do have to overcome them]* of course persistence to work, they keep getting support, so, the system, the challenges are solved as time goes. If it is resources, the meetings, the coordination, it is the coordination that resolves all these problems. The problems come and they are sent to the coordination team, the incidence management team, to the strategic meetings, the strategic team in MoH and these are the challenges, that they keep solving. So, the solutions come from that coordination.

**I: OK.**

**R:** The strong coordination that MoH has eventually solves the issues.

**I:** **OK. I am also looking at the different stakeholders, because you said during these outbreaks we have partnerships. So, how are these partnerships bond together outside MoH coordination this, how have they worked together to solve some of these challenges?**

**R:** The main thing is that partners are part of the response team. So they don’t have to go through MoH. Partners are part of the response team. The pillars that we have under the health management team. The partners are part of the surveillance, they are part of LAB pillars, they are part of the response, so they actually get the challenges first hand and they try to solve them even with or without the coordination of MoH. So, working with partners as part of the response, is basically what helps solve the issues better because the partners are seeing the problems there and actually come up with a solution.

**I: OK. So, ah we are now going to look at some of the recommendations, and we would like you to suggest some things that have been done. What are some of strategies ah, that you would propose to strengthen this health system and also resilience to infectious diseases outbreaks or shocks.**

**R:** Sighs

**I: Any strategies or initiatives that you would propose?**

**R:** I think continuously, training the pool of human resource that is should be available to tap into, ah incase there is any response is one of the main key factors. The other is MoH staying on alert with their alert systems and surveillance that help the system be resilient. Ah, the third one is, ah, the preparedness part, because there is absence of an outbreak, we need to be prepared to respond. So, ensuring that the teams that are supposed to respond are continuously prepared to be able to respond is very key.

And I think lastly, Is ah, is having the coordination. This incident management team, the public health emergency response and public health emergency center is very important if it can be in place and ensure that it is on top of any response, I think they manage every response very well. So, have that public emergency center available and running and strengthening the regional emergency centers, operation centers, is very key to ensuring that there is a quick turn around in terms of response and having human resource on standby.

**I:** **OK. Thank you very much. What about the VHTs, how can they be better supported or empowered to develop a more resilient health system.**

**R:** But VHTs also I think fall into the same mechanisms I have talked about in terms of preparedness.

**I: Hmnn..**

**R:** Having them prepared all the time is also part of the preparedness that we are taling about, so, having them involved, having them ah, trained continuously so that they are on standby, and they can understand how the response works is also part. So, I assume the VHTs they also part of the health system. So, whatever we are planning at the top goes up to the VHT at the bottom.

**I:** **OK, Thank you very much. I was just looking at the top being people with a certain level of education and understanding and probably the VHTs being at the low end and I thought probably we would be using different things.**

**R:** Definitely, the way you communicate with the VHT is different from the way you communicate with the IT but the message is the but just the packaging is the one that is different. That is why I said that what you do at the top you ensure that you do it at the bottom where the VHTs are, of course using different mechanisms depending on the levels.

**I:** **OK. Thank you, thank you very much. Ah, so, ahm, are there any successful models that you think would suit our context better?**

**R:** Context of what?

**I:** **Of health systems, resilient health systems**

**R:** Eh!, I don’t have any model that is successful that I can give you know.

**I:** **Hmnn**

**R:** I don’t have any model really, I know if there are nay models that can describe a resilient system, but I don’t think I have any.

**I:** **Ok. So, we have come to the end of this discussion. Is there anything that you would want to tell us about resilient health system to infectious diseases that we have not talked about.**

**R:** Ah, just one thing, for resilient system it needs to continuously be energized. It needs to continue to grow, as you might know it might ah, especially the human resource, they get old, they turn around, they leave, so you have to continuously train people to fill up positions because people leave, people die, people lose interest. *[“True”* ***Interjection by interviewer*]. So, you can, it is not, it is an active thing. It is not standing there and you say this is it. It needs to be re-energized, re-invested in for it to remain resilient.**

**I:** **Ok, when you say energized, I don’t know how you can energize maybe you could expound on that more.**

**R:** The system is a living thing.

**I:** **Yes.**

**R:** So it needs to be fed.

**I: Ahn..**

**R:** You have to keep feeding it in all aspects for it to remain resilient. If you stop feeding it, then it will lose its resilience. How does it lose its resilience? For example, in human resource, you lose some. It is what we talked about. Some migrate, some die off, so you need to keep feeding it with all aspects for it to still remain resilient.

**I: Hmmn..**

**R:** Yes.

**I:** **Thank you very much for your time, XXXX**

**R:** You are welcome.

**ENDS.**
