# Supplementary material for: Resilience as recovery: stakeholder perspectives on Uganda's health system response to infectious disease shocks
Source: Front Health Serv. 2026 Jul 22;6:1888814. doi: 10.3389/frhs.2026.1888814 (PMC13437620; doi:10.3389/frhs.2026.1888814)
Supplement: Supplementary file 2 [file Datasheet2.zip › KII08.docx]

**TRANSCRIPT FOR KEY INFORMANT INTERVIEW**

**Interviewer:** **XXXXX, you will excuse me, I will come back with a consent form. I didn't carry one, unfortunately.**

**Respondent:** Put on recording, I have not accepted to participate because the consent form is not here.

**Interviewer**: **I'll carry one for you, sir.**

**Respondent:** That's when I'll consent. All right, continue

**Interviewer**: **Okay, so, like I alluded to earlier**…

**Respondent:** What you've finished, you've finished, because I'll have a lot of questions for you, but not before you do your interview.

**Interviewer: Yes, sir. In your view, what would you describe as health system's resilience to infectious disease shocks?**

**Respondent:** Personally, I'm not a specialist in health system resilience but I also know health systems has multifaceted blocks. So my understanding of a health system is that all those five blocks must be there. And for a system to be resilient, the weakest point will determine the strength of the system. Systems has multiple points. Finance, skills, infrastructure, all the WHO building blocks, five of them. So you'll go back and fill in that part for me. All the five must be working.

**Interviewer**: **They are six actually.**

**Respondent:** Six building blocks, actually. Which are... Let me put my pen and remind you. Yeah. You must have finance, you must have human resource, you must have structure, you must have... tools, what we call supplies and utilities, we must have and what's the sixth one?

**Interviewer: Finally, service delivery itself.**

**Respondent:** What is service delivery? It's also another system also. Service delivery is a system. The people, the tools, the time, the layout, the monitoring. So, if you say we have a resilient health system, it must have all these components which can run independent of sudden challenges. That makes it resilient, meaning we can continue having vaccinations routinely, even when we have a COVID outbreak and lockdown. So extraneous factors do not interrupt the essential health services. That's when you have a resilient health system. Resilience means it can bend, it can stretch, but it will not break down.

**Interviewer**: **That's helpful. Thank you so much. So the Ugandan system... Well, I think we may not have a yes or no answer with regard to whether our system is resilient. But what do you think, according to those the health system building blocks, what are some of the factors in each of these blocks that you think have contributed to whatever level of resilience we have as a country?**

**Respondent:** Most of our systems depend on supplies and medicines. Once there's a break in that, the system does not hold at all. Because many of the things can be put in place, but the moment the supplies are not in place, the reason why that is critical is because the majority of the supplies and drugs are not manufactured locally. We depend on some other logistical supplies and some other manufacturers to get us what we need. So we outsource a lot of those things in our system. It makes it very fragile.

**Interviewer: So do we have any mitigating factors?**

**Respondent:** The government has gone for BUBU (buy Uganda, build Uganda), and now we are all saying we need to have local manufacturing in Africa, in Uganda, whatever therapeutics, diagnostics, and even vaccines as much as possible. We've also gone back and said let's promote natural products, pharmaceutical products locally being used so that we understand what's there, so that in case of disruptions...

**Interviewer**: **We can resort to the natural...**

**Respondent:** Yes, we have a fallback position.

**Interviewer**: **What about with regard to financing, are there any steps you've taken to make sure our health system is more resilient?**

**Respondent:** There's no step for resilience in financing. Finance resilience comes with economic development. So it's not a prerogative of the Ministry of Health to secure financing to make people wealthy.

**Interviewer: But what about at the health sector level? Let's say do we have any funding, any emergency funding in an event…**

**Respondent:** No, the government does not put money aside for emergency and the health, but it has a Ministry of Disaster Preparedness. So that ministry must have the ability to respond to whatever disaster, including health disasters. That would be the kind of funding we have. Financing is good and needed, but it's in tandem with the development of the country. I think where we have a challenge is that we have not needed legislation to put money aside for the known and predictable outbreaks, because the way money comes into our country is kind of saying... How do they call it? a cash economy. So whatever money is collected is what the government spends. So they cannot start putting aside money they have not yet received, because they depend on tax revenue collection to spend. And it's hard to convince them to collect this money and put it away. They always think they... I don't know. So that's one of the areas.

The other one is to have non-traditional methods of having money pooled together. Health insurance would be one of them. Community insurance could be another. But also other licenses which are paid for instance, there is road licenses. That money is for accidents and emergencies. That money should be pooled and put aside. As long as it's money for road licenses, and you know that the biggest contributor to long-standing hospitals is road traffic accidents. So that money should be put aside as unique domestic resources. They also do taxes. They call them SIM taxes for alcohol and cigarettes, very high exorbitant to prevent people from smoking. That money should have been put aside to help with health financing. Because that's the reason we collect it, to help the health because they have been smoking and they have caused all these lung diseases and pneumonias and mines and pollutions. Those would be unique if we want to have a resilient system. Unfortunately, I don't think the economy is at that level.

**Interviewer: So are you aware of any, let's say, community health insurance that you mentioned? Are you aware of any that's going on?**

**Respondent:** In Bushenyi, Nyaruguru. Another community initiative I had in Buganda is national insurance, Buganda insurance. I don't know if it has started.

**Interviewer: Buganda?**

**Respondent:** Yes. Buganda government.

**Interviewer: Ah, okay. That's new. But Bushenyi has one that's running.**

**Respondent:** That's where I've heard. And there was a community health insurance initiative we heard about in one big meeting. Unfortunately, I didn't write it down. And I think it's in western Uganda. We should read about it. Who was in that meeting? There's a big, even donor-funded to set it up. I think it was being supported by some of these multi-agencies.

**Interviewer: As a pilot?**

**Respondent:** As a pilot, but it's been going on. So they work with the bank. They also work with communities, the communities collect the money, the bank keeps the money. And when one of the people in the community falls sick, they have money to pay. And they have signed up with some non-profit hospitals to give services at a low cost for those communities. I want to remember. It's either running around Kisizi Hospital. It's a community health insurance.

**Interviewer: Now that you mentioned it, I think I heard about one that's run by a Catholic facility in Ntungamo. I don't remember the name of the facility.**

**Respondent:** There are many. I threw away the presentation. But the way it works, it was impressive.

**Interviewer: That's one that would be very helpful. What about around health information? Are there things that we are doing?**

**Respondent:** Resilience in health information? Because health information is what? It's surveillance data. So if we want to say we have a resilient health system and we are looking at the pillar for information and data, we need to be collecting data routinely and consistently and reporting on all occurrences and being able to diagnose even when there are strange happenings. So rumor correction, rumor analysis, quick response in terms of having people who can be on the ground to detect, timing cases, the Ebola outbreak, the most recent. It was a surveillance person who picked it, mortality surveillance. He said, this body cannot be released. I just read in the file. The circumstance under which this person died is strange, Let us test for Ebola. One man can save the country the whole trouble.

**Interviewer: Okay, what about issues around data sharing? Do you think it's across, let's say, I've been around, but I'm just trying to avoid my own experience coming in the way of this interview.**

**Respondent:** Then you let the other person ask. Continue.

**Interviewer: Yes, but data sharing, especially from the privates to private, private to public, public to private, do you think there's adequate data sharing to help us understand the way we are in our health system? Is it adequate enough?**

**Respondent:** Absolutely not. It's a big hindrance. For instance, in KCCA, which is part of government, they have 2,208 health facilities registered. But the reporting is up to most only 31% for the weekly and 66% for the monthly. And we even have a list of health facilities not reporting. So we already know that we've even written to them asking for this data, we are getting less than optimal, especially in Makindye. So what do they say, they make our system very fragile by not doing their work routinely, and so we can't even tell why the slump in reporting. Is it because they were using donor money? Were they having people hired by USAID to collect and report data? Because we know all partners, their number one interest was data, for good, for bad, but their main one interest. That's why they have things like Mets. That's why they have data.

**Interviewer: To focus on data.**

**Respondent:** Yes, only. A $6 million project just for data.

**Interviewer:** So this is an indictment on them because I don't know how the figures were before the interruption, before the executive orders.

**Respondent:** We shall check. It may not have been any different, when you go in all health facilities, the data unit is run by PEPFAR-USAID projects. All of them. The data informants, the clerks are all paid by donor money. Yes.

**Interviewer:** Which makes it a bit unsustainable.

**Respondent:** And then the pay rates are distorted. So because of that distortion, we can say it's one of our biggest challenges. Information, especially for what you're asking about. Can I give you some water?

**Interviewer: No, no, it is okay. Thank you so much. Now, what about leadership as a block? What do we have in that bloc that has has fostered resilience, or any if you have.**

**Respondent:** I think leadership is good. It has its gaps but it will... You know, for anything to hold, leadership must be in place. But we overhyped leadership without really facilitating it. 30% of the DHOs are in acting capacity. That's an indictment on our leadership roles. Why don't the districts want to appoint people substantially? They're waiting for their sons to graduate. Or they want weak people who can be manipulated for their own sake. So I don't know whether I say that is good or that is bad, but everything rises and falls on leadership, according to John Maxwell. Therefore, if you have bad leadership, however good the system is, they will fail it.

**Interviewer: Yes, that's true. So if you brought this down to levels, would you say at the national level we seem okay? At the regional, I don't know how things are.**

**Respondent:** So we must go back and divide our health system. Our system is not flat. We have primary health care. We have hospital care. We have community care, then we also have national leadership for policy and strategy. So at the Ministry of Health headquarters, we have policy and strategy. And then at national level, at regional level, we have specialized care, which is tertiary care for regional referrals, hospitals and district hospitals. Then we start going to lower level health facilities. All those have different people they report to. At health centre IV, they are reporting at the district, local government. Whereas they are run by Ministry of Health. But all the staff come from the local government. They report to the CAO, Health centre III, CAO. They are paid by the district. So there is a big... There is a big gap to address in terms of decentralization.

**Interviewer: So while that is a gap, can that be a blessing with some proper coordination between Ministries, do you think that would sort out the issue?**

**Respondent:** It would if we allow them to appoint a district health officer who is fully in charge. But one district health officer cannot supervise five health centres. IVs or IIIs, with all their staff, plus one district hospital. And if he was the only technical person, medical, he would not be able to see those gaps. He will see, but he won't see.

**Interviewer: Wow, okay. Then... Around health infrastructure.**

**Respondent:** We are good. Infrastructure, we are good. The numbers are continuously becoming adequate because the focus of health facilities is to make sure we provide routine vaccination and all mothers deliver in health facilities, at that level. We are not in a... We are not... Our idea is not to provide curative services over preventive services. So we are looking for at primary health, primary prevention of disease, health education, and then providing services where we want people to have professional care. So we only have to have good maternities and immunization programs with minimum curative care. Maybe for the elderly, maybe for the chronic diseases. In terms of infrastructure, that's what we want to see. The infrastructure is spread out evenly so that people can have where to deliver their babies.

**Interviewer: Okay, so there is this issue of community. Diseases originate from the communities and somehow the idea is to nip them in the bud from where they start from. And so in your view, what is the unique role of the community in giving us resilient health systems?**

**Respondent**: If the community are empowered in terms of knowledge and they know what to report, how to accept messages and information and how to use the service delivery, for instance, if you put out a vaccination program, the community should be able to come without telling us we want to kill them, why would one want to kill a community using vaccines? No. That is being so silly. Trying to do a good job with a very slow and inefficient method. And by even leaving a track record. So once people understand that nobody wants to kill you and give you a track record on how he killed you, say we are using immunization to kill us, how? You will know. And who did it? It's a very inefficient method. But because that's the level of thinking. So we need to find a way of debunking lies and promoting and not using force at all. Because the more you use force, the more people may think maybe there is a sinister. ...something behind what you want to do. Okay.

**Interviewer: Thank you so much. So now, so thinking about a resilient health system, what in your view would be the attributes, the characteristics of a resilient health system? If we say the Ugandan health system is resilient, which is because of A, B, C, D, what are those attributes?**

**Respondent:** The system is a set of things working well together, that's a system. That's a machine. Multiple parts working well in tandem. Why do I say my Ugandan health system is resilient? Ugandan health system is very resilient to disease outbreaks. Eight Ebola outbreaks, four Marburg outbreaks, multiple yellow fever outbreaks, but we respond. So being able to respond without disruption of the rest of the country is a sign of resilience. But it does not mean that a resilient system is a perfect system. It's one which can auto-run and reset its reference back to where it was. So if we are at vaccination 20% or 80%, we've got a disruption, we are able to reset back to the 80% in the shortest time. Not that a resilient system is a perfect system. No. It's one which can reset and recalibrate back cost correction in the shortest possible time. We've been vaccinating six killer diseases. We're now at 14. And despite of all the disruptions, we are able to continue.

**Interviewer: So do we somehow... How do we balance our staff? How do we... What is it that makes the health system able to do that?**

**Respondent:** I think we've... It's been growing over time. The system you see is not a one-off thing. It's not because somebody did something magical. But it's been the incremental investment over so many years. The health system starts by saying we want to reduce maternal mortality. I'm giving an example, So you start by building maternity centres to expand them. You start by equipping them. Then you train nurses and midwives. That's part of building. Then you incentify and attract analysts in the hospitals. Then you facilitate putting theatres where you've capacitated. Then after you've put theatres, then you put NICU to help the newborn. Then you come to have local maternity networks which give each other peer support. Then you bring in ambulances for quick referral. So incrementally you are building a system, but over time. So just to make sure that you reduce your maternity mortality by even a figure of six mothers, you have to put all those in place over time and in multiple places. And also making sure neonatal tetanus is handled, preeclampsia is handled, supplies are coming in time, obstetricians are in post. Just to make sure you change your figure by one. Then traditional birth attendants are discouraged, are weeded out. All the bad practices are talked about. Then that will encourage mothers to come and use the facility and then you reduce in maternal mortality. Same for infant mortality. You start by training the mothers, give them vaccination, follow them to follow the children, nutrition, communities training and tracking who needs to be given care, prevent malaria, give them nets. So you are now beginning to reduce the chances of dying for every child. And then finally you see that even the fertility rate goes down because they are able to raise the children they have had. So no need to have 14 children because you know if you have seven, seven will survive.

My grandmother who had 14, her children had seven, now for me I can have four. It's a scale down. And then my children can have two and a puppy.

**Interviewer: Interesting. And a puppy to go with it.**

**Respondent:** Yes.

**Interviewer:** So now what are some of the challenges I think that we face as a country?

**Respondent:** The overwhelming challenge is the low level of development in the country. Poverty is real. And poverty being real, people must be participating in the economy to enjoy good health. And so we can't have it both ways. Having the low participation in the economy and having good health services, the two go hand in hand. So people will have some money, they will build better houses, they will have better clean water, they will have better education, and then the economy will grow and then we shall have where to pull their money to provide them health services. Because we only tax who is available to give the resources to them. But we are not taxing you. You are not contributing to the money but you are crying for good health services. Nobody can manufacture it from nowhere. But there is no way you can say that it's not politically corrected. Everybody says, but we voted you. Yes, but you are not paying taxes. Even if you voted me, you are having nothing productive you are giving to the economy.

**Interviewer: I agree. That's a big issue.**

**Respondent:** So, the best prize for our health economy, I mean for health, is to industrialize, increase job and market and productivity in the country. 50% of our health issues will be solved. And I didn't know that until I saw people participating in wealth creation and Emyoga and how they have transformed their 2 million pigs into something which gives them cash. And you see the women now beginning to build better houses, having their floor tiled, or even screened. No jiggers. Finished. Simple, simple participation in something they can start to produce from. And you can transform a village. So if you give me 2 billion, I take to my village to be transformed.

**Interviewer: So let me take you back to the issue of, to the community. You mentioned about how the community is worried that we're going to be killed, of course misinformation, by anti-vaccines. Is there an element of trust? Do you think there are trust issues? Or... Or it goes either way. I know the community also trusts their VHTs, but when it comes to vaccination, it's a mixed bag. I don't know where the trust lies and where the mistrust lies.**

**Respondent:** Yeah. I'm married. Trust is built over time. And one stupid error can kill the trust. When the communities have been trusting people, one bad, unfortunate occurrence can cause them to doubt what they previously thought to be true. And now with social media, our mistakes are amplified ahead of our successes in whichever way. And so that's the work of the devil. I can't forget to tell you about the devil. He's a loser, he's a liar, he's a bad loser and I hate him with a passion. Record that.

**Interviewer: Yeah and so how are we going to deal with the, how do we deal with the trust?**

**Respondent:** With the trust is, I think we need to use community resource people. We need to use the local leaders. We need to empower them to give the information we want them to pass on. Because they are in more touch with the people, they live with them. Policy makers are external to the community. If I stood in Agago with my accent and told them about health services, they'll say why doesn’t he go back to his home and speak. But if I got Charles Oyo who used to work there and I sent him back home to speak the same language, to sleep in the same community and then give the message, it will be received differently. So and we have churches, we have mosques, we have traditional leaders. Those are the best people to give the message. So we should invest in empowering them. Please when you go say this, do you believe what we've said is correct? Yes, then go and say and we should be willing to fund them. If they're going to speak on our behalf, we should be able to finance their activities.

**Interviewer: Okay so a little bit, I know we haven't really spoken much about the private sector. I don't know if the private sector does have a role towards building or breaking health system resilience.**

**Respondent:** Because we said by saying our health system is built by what?

**Interviewer: By partners.**

**Respondent:** So private sector, we talked about the not for profit. They are all partners, they are all private, only that they are not there to earn money. When we think about private, we think about those who want to recoup a hundred percent of their resources. No, they're those who want to give benevolence and goodwill to the community through their skills and service and subside. So those are all private sector. They complement what government does. Can you imagine if there were no private sector, all these high-end hospitals would not be around.

**Interviewer: That's true.**

**Respondent:** And so the people who have money cannot go and line up with those who don't have money. Society has already separated them. They want ambience when they're waiting to see a doctor, they want to sit on a chair and not on a mat and have some mineral.

**Interviewer: So how can stakeholders overall work together to overcome some of the challenges that we have?**

**Respondent:** We're already working together with stakeholders as government. We have departments for partnerships and multi-sectoral collaborations, coordination. We have agreements with government of PPP, public private partnerships, investments. We already share tools. We share resources for routine immunization. We give grants to hospitals and second staff that are not-for-profit. We have partners whose role is to fund through private sector like JMS, who receives ORT to all those private sectors, but they are all working for government even if they are off-budget. So we also have grants which come basically to allow people to sell subsidized products, condoms, mosquito nets, antimalarials. We give them at free cost to people who can vend them at minimum price. Those models have not worked well, but they work where they have seen. We also want to have private health insurance. You know all these companies secure their workers. They pay insurance for them. That is providing government's resource. People who are working hard and lining up in Kawempe, they will just go to Kampala Hospital to have their baby.

**Interviewer: Use their insurance.**

**Respondent:** Any money spent in the health is a saving from government.

**Interviewer: So finally, how can VHTs be supported better to contribute to the resilience of the country?**

**Respondent:** We cannot do away with VHTs at the moment because they are the foundation of some of the work we do in the community and they are so numerous that you cannot even eliminate them. So you support by giving them a structure to motivate them and also for them to feel that they are useful because all the work they do is voluntary. They can also be given tools to facilitate what they do. And when there are opportunities elsewhere, then they can be, if there is PDM money, funding, we should give them priority, if there are scholarships for education, VHTs should be given scholarships. If their child goes to university, we should say every VHT who has a biological child at the level of getting the grades, it should be an incentive.

**Interviewer: By the way that is a nice way of pushing it.**

**Respondent**: You say VHT your child has gone to university, government scholarship.

**Interviewer: Have you tried pushing that narrative?**

**Respondent**: You've just asked me now. Nobody ever asked me.

**Interviewer: So you've just thought about it. Maybe we should take that as a strategy.**

**Respondent**: Yes, we should take it strategically and say their children are struggling, but should any one of them make it to university? Why should you give ministers children scholarships?

**Interviewer: I think that is a very wonderful way of giving back to VHTs who volunteer.**

**Respondent**: The same should be for children for veterans.

**Interviewer: That's extremely creative.**

**Respondent**: Veterans, you'll never pay them enough, but should their child make the marks, scholarship automatic.

**Interviewer: I now feel like writing an opinion piece. I'll credit you in my write up**.

**Respondent**: Do that. Because if nobody's thinking about these things, it won't happen. But can you imagine if you say, I've not had enough school fees, but maybe one of my children would make it?

**Interviewer: Wow, so Director, I think that's the end of our interview**. Thank you so much for making time for this. I know we kept missing each other.

**Respondent**: So you have taken up the interview time for another person.

**Interviewer: Oh really? I apologize on their behalf.**
